# Electroluminescent photoresists extending lithographic scaling to OLEDs

**DOI:** 10.1038/s41586-026-11042-0

**Published:** 2026-09-16

**Authors:** Shao-Wei Lo, Sunil B. Shivarudraiah, Zhan-Hong Lin, Patrick Helbling, Lifei Song, Jiachen Wang, Miguel Nouman, Fuze Jiang, Gerrit Stemmler, Donato Maria Carretta, Mei-Nung Chen, Jiayi Zhu, Sudhir Kumar, Hua Wang, Andrew J. Christofferson, Yinyin Bao, Chih-Jen Shih

**Affiliations:** 1 Institute for Chemical and Bioengineering, Department of Chemistry and Applied Biosciences, ETH Zürich, Zurich, Switzerland; 2 School of Information Technology and Electrical Engineering, ETH Zürich, Zurich, Switzerland; 3 Department of Chemical Engineering, National Taiwan University of Science and Technology, Taipei, Taiwan; 4 School of Science, STEM College, RMIT University, Melbourne, Victoria Australia; 5 Department of Chemistry, Faculty of Science, University of Helsinki, Helsinki, Finland

**Keywords:** Polymer synthesis, Organic LEDs, Surface patterning, Organic LEDs

## Abstract

The miniaturization of organic light-emitting diodes is critical for next-generation ultrahigh-resolution displays and integrated photonics^1–8^. However, traditional vacuum evaporation and inkjet printing methods are incompatible with the lithographic scaling processes that underpin silicon electronics^9–16^, hindering true monolithic integration with complementary metal–oxide–semiconductor circuits. Here we present electroluminescent photoresists synthesized via atom transfer radical polymerization that can be directly patterned by ultraviolet and electron-beam lithography. These multi-arm star polymers feature a core–shell architecture designed to embed the thermally activated delayed fluorescence emitters within a protective host shell, segregating them from the reactive photocrosslinking moieties on the surface. The site-specific isolation ensures that crosslinking reactions occur at the periphery, leaving the emissive cores intact and preserving high electroluminescence. We demonstrate robust processing orthogonality in sequential multilayer electroluminescent photoresist photolithography, enabling subdiffraction fluorescence nanopatterns with critical dimensions down to 110 nm. We validate their device performance with multicolour, ultraviolet-patterned organic light-emitting diodes exhibiting external quantum efficiencies exceeding 13%. These results not only pave the way for extending Moore’s law to organic optoelectronics but also unlock their potential for monolithic optoelectronic integration.

## Abstract (web summary)

Electroluminescent photoresists synthesized via atom transfer radical polymerization can be directly patterned by ultraviolet and electron-beam lithography, and used to fabricate monolithic, multicolour organic-light-emitting-diode micro-pixel arrays.

## Main

The monolithic integration of high-performance light sources with silicon-based complementary metal–oxide–semiconductor (CMOS) electronics is a cornerstone for next-generation optoelectronics^1^, promising to revolutionize augmented-reality microdisplays^2^, optical computing^3^ and high-speed on-chip interconnects^4,5^. Although inorganic III–V semiconductor devices have dominated research in this field, their integration remains fundamentally constrained by lattice mismatch, the requirement for high-temperature epitaxy^6,7^ and the yield-limiting complexity of heterogeneous transfer processes^8^. Furthermore, as pixel size scales into the sub-10-μm regime, inorganic emitters suffer from severe efficiency roll-off owing to non-radiative recombination at sidewall defects^17–20^. In this regard, organic semiconductors emerge as a compelling alternative^21,22^. For example, in organic light-emitting diodes (OLEDs), their amorphous emissive layers, coupled with low-temperature and lattice-free fabrication, enable direct back-end-of-line compatible processing on CMOS wafers while preserving high external quantum efficiencies (EQEs) independent of device dimensions^23^.

Despite these material virtues, a viable technical roadmap towards the monolithic integration of multicolour OLEDs remains elusive^24^. The primary bottleneck lies in the chemical fragility of organic semiconductors, which are readily degraded by the solvents and developers inherent to standard photolithography^21,25^. Consequently, current manufacturing methods, such as fine-metal-mask evaporation and inkjet printing, are fundamentally limited by their restricted spatial resolution and overlay accuracy^9,10,26^. More critically, these techniques are inherently decoupled from subsequent photolithographic processes, preventing OLED technology from aligning with the rigorous integration densities and scaling laws dictated by modern silicon circuitry.

The development of directly photolithographically patternable organic emissive layers (EMLs)—a material system we formalize here as electroluminescent photoresists (ELPRs)—was first envisioned in the early 2000s to enable the multicolour integration of solution-processed polymer OLEDs^11,12^. Initial attempts utilized *π*-conjugated polymers with oxetane side groups synthesized via random polycondensation, forming insoluble networks through acid-catalysed crosslinking. Nevertheless, these early ELPRs were rather underperforming, with EQEs typically below 2% (refs. ^11,12^). This performance gap resulted from: (1) the intrinsic limitations of early fluorescent polymers, which harvest only singlet excitons; and (2) the stochastic distribution of emissive units and residual photoacid additives, which created potent quenching sites. In the decades following these early reports, although synthetic efforts successfully incorporated the advanced OLED emitters^13,21^, such as thermally activated delayed fluorescence (TADF) molecules^14,15^, into polymer chains^16,27–30^, the development of ELPRs has remained frozen. This persistent performance gap stems from the pervasive nature of crosslinking reactions; without precise structural control, these photochemical processes inevitably introduce non-radiative decay channels or disrupt charge-transport pathways, compromising the optoelectronic performance.

**Fig. 1:**
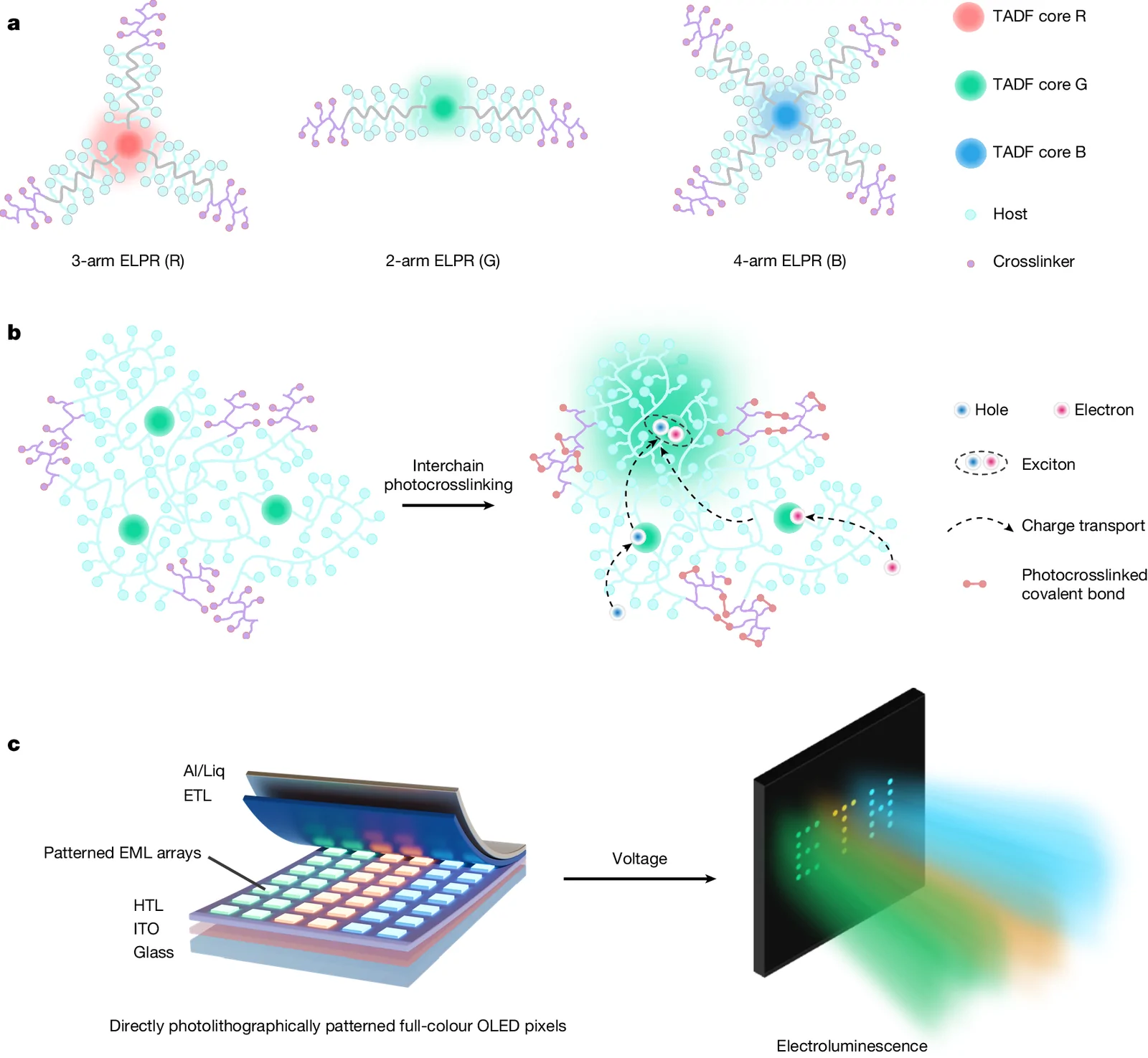
Molecular design of high-performance ELPRs enabled by controlled radical polymerization. **a**, Schematic illustrations of the multi-arm star polymers synthesized via ATRP, featuring green (G), red (R) and blue (B) TADF emitters as the central cores. Each arm comprises two sequential blocks: an internal host shell for exciton confinement and a terminal periphery for crosslinkable functionality. **b**, Left: schematic of the site-specific, core–shell compartmentalization within ELPR films. Right: upon ultraviolet or e-beam exposure, radical-mediated interchain crosslinking is spatially restricted to the terminal boundaries, effectively shielding the emissive cores and preserving the integrity of exciton recombination and charge transport. **c**, Demonstration for the monolithic integration of multicolour OLED micro-pixel arrays, realized through three consecutive, developer-orthogonal photolithographic cycles. ETL, electron-transport layer; ITO, indium tin oxide; Liq, 8-hydroxyquinolinolatolithium.

Notably, the early struggles of ELPRs coincided with the infancy of controlled radical polymerization^31,32^, which now offers an advanced synthetic toolset for tailoring molecular architecture. We hypothesized that by encapsulating individual emitters within a three-dimensional host matrix, exciton quenching induced by peripheral crosslinking could be fundamentally suppressed. Here, leveraging atom transfer radical polymerization (ATRP)^33^, we engineered a multi-arm star polymer architecture centred on a single TADF emitter. This core serves as the nucleus for star polymer arms consisting of two functionally distinct blocks: an internal host shell designed for exciton confinement and energy transfer, and a terminal crosslinkable periphery for structural definition (Fig. 1a). This site-specific, core–shell compartmentalization ensures that the radical-mediated crosslinking is restricted to the polymer boundary, effectively shielding the emissive cores from chemical and electronic perturbations (Fig. 1b).

Consequently, after ultraviolet patterning, our TADF-active ELPRs achieve a high EQE of 13.3%, representing a major leap for directly photolithographically patterned OLEDs. Furthermore, we demonstrate the robust processability and exceptional solvent orthogonality of these materials by fabricating monolithic, multicolour OLED micro-pixel arrays via three consecutive photolithographic cycles (Fig. 1c). By incorporating electron-beam (e-beam)-sensitive crosslinker moieties, we extend this capability to the nanometre regime, achieving bright emissive patterns with critical dimensions down to 110 nm—bridging the gap between microscale display technology and nanoscale integrated photonics.

## Design strategy and modular synthesis of ELPRs

**Fig. 2:**
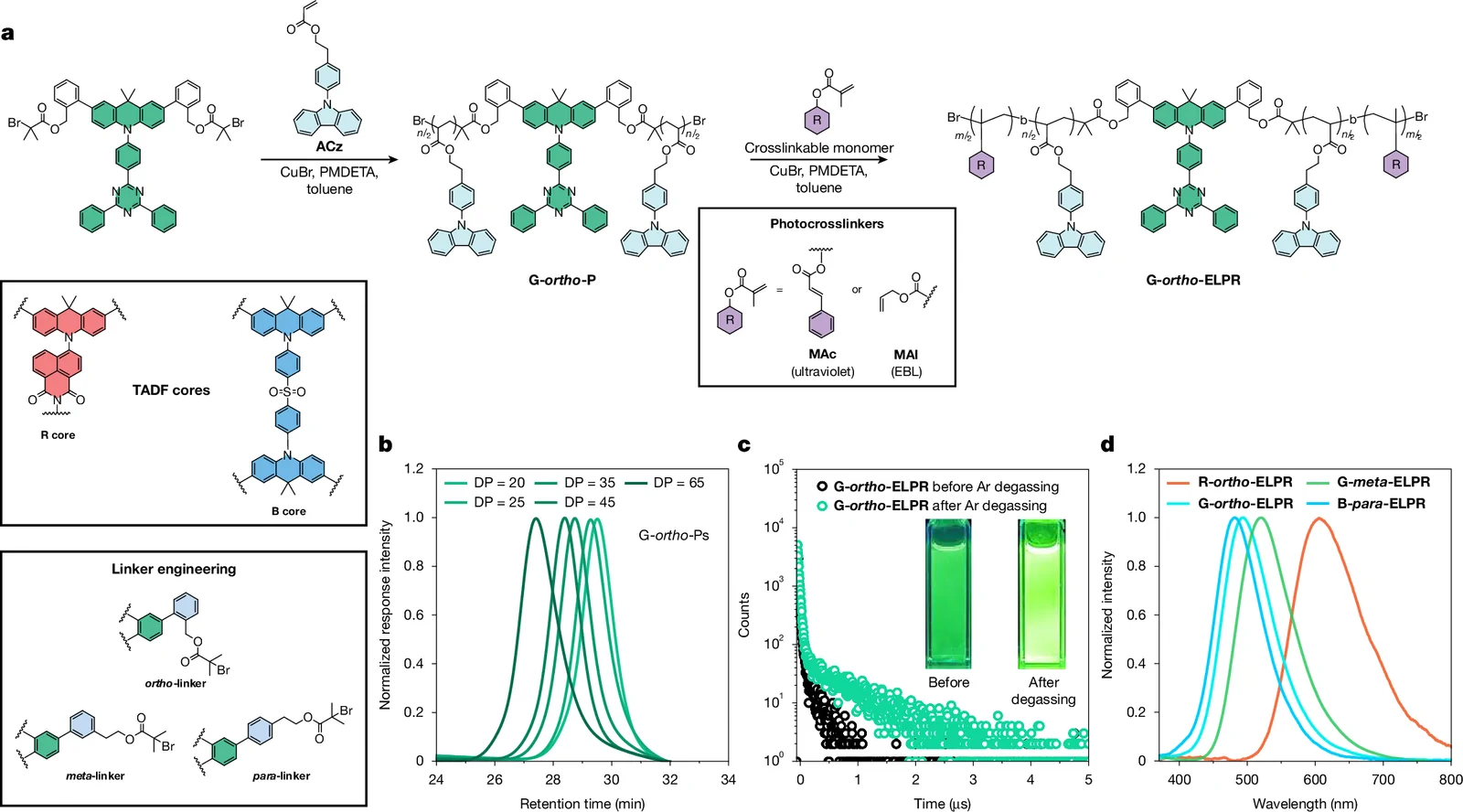
Modular ATRP synthesis and photophysical characterization of ELPRs. **a**, Synthetic route for directly patternable green-emissive ELPRs. The multicoloured TADF initiators, functionalized with *ortho*-, *meta*-, or *para*-linkers, are utilized to tune the emission chromaticity. The TADF cores are encapsulated within a host shell composed of pendent phenylcarbazole groups via the ATRP of ACz monomers as the first block. Subsequent synthesis of the second block via polymerization of either ultraviolet- or e-beam-crosslinkable monomers yields functional ELPRs. *n* and *m* denote the degrees of polymerization (DP) of the respective monomers (*n* for DP_ACz_; *m* for DP_MAc_ or DP_MAl_). **b**, Size-exclusion chromatography of self-hosted **G-*ortho*-P** precursors with varying DP_ACz_. **c**, Comparison of TRPL responses and fluorescence photographs (insets) of G-*ortho*-ELPRs in toluene before and after purging with argon (Ar). The enhanced fluorescence and increased *τ*_DF_ reveal TADF activity. **d**, Photoluminescence spectra of representative red, green and blue ELPR thin films: **R-*ortho*-ELPR** (DP_ACz_ = 42, DP_MAc_ = 9), **G-*meta*-ELPR** (DP_ACz_ = 40, DP_MAc_ = 12), **G-*ortho*-ELPR** (DP_ACz_ = 45, DP_MAc_ = 12) and **B-*para*-ELPR** (DP_ACz_ = 50, DP_MAc_ = 12). PMDETA, 1,1,4,7,7-pentamethyldiethylenetriamine. Source data

We selected three representative TADF emitters—red (R), green (G) and blue (B)—as the central cores for our ELPRs. These scaffolds share a common donor moiety, 9,9-dimethyl-9,10-dihydroacridine (DMAC), coupled with colour-specific acceptor groups (naphthalimide^34^, triazene (TRZ)^35^ and diphenyl sulfone^36^) to span the visible spectrum. The DMAC moiety was site-specifically functionalized at the 2,7-positions with 2-bromo-2-methylpropanoate initiating groups via a phenyl linker^37^, ensuring that each polymer chain grows outwards from a single emitting core via ATRP (Fig. 2a).

Utilizing this central initiation site, we first synthesized self-hosted TADF polymers, the ‘P-series’ precursors, by polymerizing host monomers with varied aromatic structures as the first block. All the synthesized molecular monomers and polymers were thoroughly characterized (Supplementary Notes 1–10). Among the designed host monomers, an *N*-phenylcarbazole derivative bearing an acrylate group (ACz; Fig. 2a) was selected throughout this study owing to its superior photophysical and electroluminescene performance^38^ (Supplementary Note 11 and Supplementary Table 1). To achieve optimal TADF activity, the emitter doping concentrations were precisely tuned between 5 wt% and 15 wt% by simply controlling the polymer chain length. A defining feature of this approach is the retention of dormant bromide chain-ends following this initial stage, enabling the subsequent re-initiation and growth of a secondary functional block. To ensure clarity, these intermediate polymers are designated with a prefix (R-, G- or B-) for the emission colour, followed by the substitution position (*ortho*-, *meta*- or *para*-), and the suffix ‘P’ to denote the pure self-hosted polymer before crosslinker attachment (Fig. 2a). All synthesized polymers exhibited well-controlled degrees of polymerization (DP) and narrow dispersities (*Đ*), owing to the efficient ATRP method (Fig. 2a, Supplementary Note 12 and Supplementary Table 2). For example, the **G-*ortho*-P** series showed a DP of 20–65 with a *Đ* of 1.16–1.25, enabling tunable doping concentration of 4.6–15.0 wt%.

Building on these precursors, a second-block synthesis was executed to incorporate a terminal crosslinkable periphery, transforming the P-series polymers into functional ELPRs. Methacrylates with cinnamoyl^39^ (MAc) or allyl^40^ (MAl) groups were used to confer ultraviolet and e-beam patternability, respectively (Fig. 2a). This modular approach allows for the independent tuning of emission characteristics and crosslinking density, and the multi-arm star architecture ensures the TADF core remains spatially isolated from reactive polymer boundaries.

Time-resolved photoluminescence (TRPL) spectroscopy was first used to evaluate the impact of molecular architecture on excitonic characteristics. The results of **G-*ortho*-ELPR** in toluene solution first confirm that the TADF activity is intrinsically preserved within the polymer framework, as evidenced by the prompt and prominent delayed fluorescence lifetimes observed under deoxygenated conditions (*τ*_PF_ = 27.5 ns and *τ*_DF_ = 1.14 µs) (Fig. 2c and Supplementary Table 3). The TADF characteristic of **G-*ortho*-ELPR** is confirmed by a high total photoluminescence quantum yield (*Φ*_tot_) of 50%, which is dominated by the delayed fluorescence quantum yield (*Φ*_DF_) of 39%. The favourable exciton dynamics are attributed to the fact that the non-radiative rate constant of the triplet state (*k*_nr_^T^ = 6.18 × 10^5^ s^−1^) is outpaced by the rapid reverse-intersystem-crossing rate (*k*_RISC_ = 3.64 × 10^6^ s^−1^) and radiative rate constant (*k*_r_^S^ = 3.93 × 10^6^ s^−1^). The photophysical and kinetic characteristics of the self-hosted TADF polymers and ELPRs are summarized in Supplementary Table 8. In the solid film, **G-*ortho*-ELPR** exhibits a delayed fluorescence lifetime of *τ*_DF_ = 0.7 µs, comparable to that of the parent TADF molecule^38^ and the polymer in solution. The delayed fluorescence also reveals strong temperature dependence, corroborating that the TADF mechanism is reserved in our ELPRs (Supplementary Note 13). The temperature-dependent TRPL responses of the thin-film sample of **G-*ortho***-**deBr** reveal that *τ*_DF_ increases with decreasing temperature from 298 K to 220 K, exhibiting typical TADF characteristics^41^ (Supplementary Fig. 76 and Supplementary Table 12).

**Extended Data Fig. 1:**
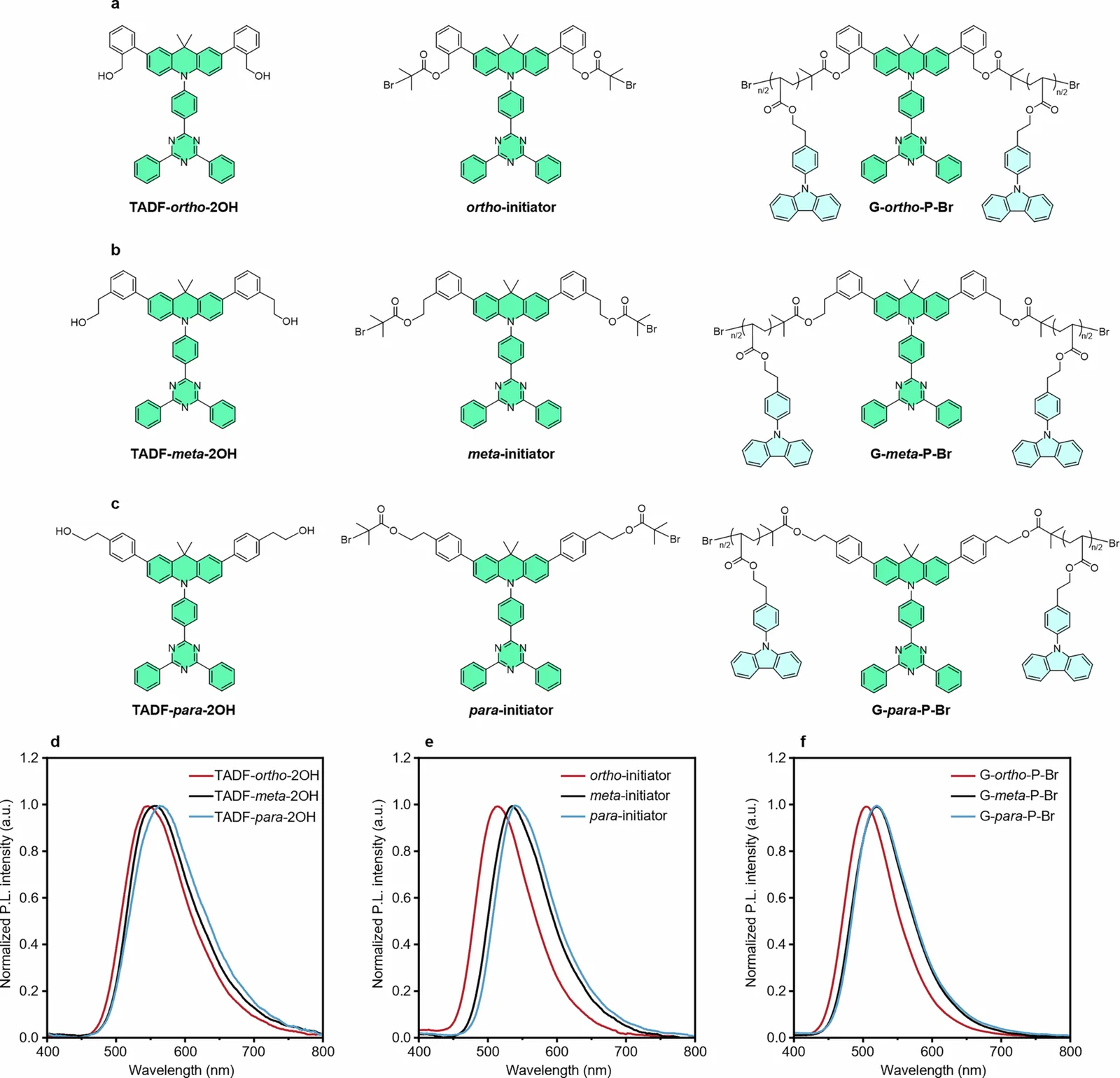
Linker engineering on TADF emitting core for fine tuning of emission wavelength of the synthesized polymers. **a**-**c**, From left to right: TADF emitting core, TADF initiator, and green-emitting self-hosted polymer functionalized with *ortho*- (**a**), *meta*- (**b**), and *para*- (**c**) spacers. The DP of polymers was kept constant for proper comparison (DP_ACz_ = 18–22). **d**-**f**, Thin-film PL spectra of TADF emitting cores (**d**), TADF initiators (**e**), and green-emitting self-hosted polymers (**f**).

Although the emission profiles are primarily dictated by the central TADF core, we found that linker engineering at the initiation site also has an important role in determining emission spectra and quantum yields. For example, the substitution position of the DMAC donor considerably influences the photophysical performance: **G-*ortho*-P** (DP_ACz_ = 20) exhibited a photoluminescence quantum yield (*Φ*_PL_) of 74% with an emission maximum at 505 nm, whereas **G-*para*-P** showed a reduced *Φ*_PL_ of 48% at 522 nm; both were measured in solid films under ambient conditions (Extended Data Fig. 1 and Supplementary Table 5). These findings reveal the enhanced steric hindrance imposed by the *ortho*-linkers^42,43^, which effectively preserves the reverse-intersystem-crossing process within the TADF core and minimizes non-radiative decay, thereby maximizing the radiative decay of excited states in the self-hosted polymers. Building on these insights, linker engineering in combination with donor–acceptor design have enabled multicolour photoluminescence emission from the synthesized ELPRs, covering nearly the full visible spectrum (Fig. 2d).

We further investigated the evolution of optoelectronic properties upon incorporating photocrosslinkers by comparing photophysical properties of the self-hosted TADF polymers (P-series) with those of their functional ELPR counterparts. Upon the growth of the second block on the periphery, a reduction in *Φ*_PL_ was observed; for example, the *Φ*_PL_ value decreased from 81% for **G-*ortho*-P** (DP_ACz_ = 45) to 40% for **G-*ortho*-ELPR** (DP_ACz_ = 45, DP_MAc_ = 12); both were measured in solid films under inert conditions (Supplementary Note 14 and Supplementary Table 6). This initial drop is probably attributed to the increased separation between emissive units and the change in local dielectric environment introduced by the methacrylate periphery, and the potential charge-transfer processes induced by the electron-donating cinnamoyl groups^37^. Notably, subsequent ultraviolet photocrosslinking did not further degrade the quantum efficiency, but instead slightly enhanced the *Φ*_PL_ to 44%. This enhancement suggests that the formation of a dense, crosslinked network rigidifies the polymer matrix, thereby suppressing non-radiative decay channels. Crucially, these findings confirm that the photochemical crosslinking process itself does not introduce additional quenching sites—a significant departure from traditional ELPR systems where radical-mediated reactions typically induce severe photoluminescence quenching^44^.

**Fig. 3:**
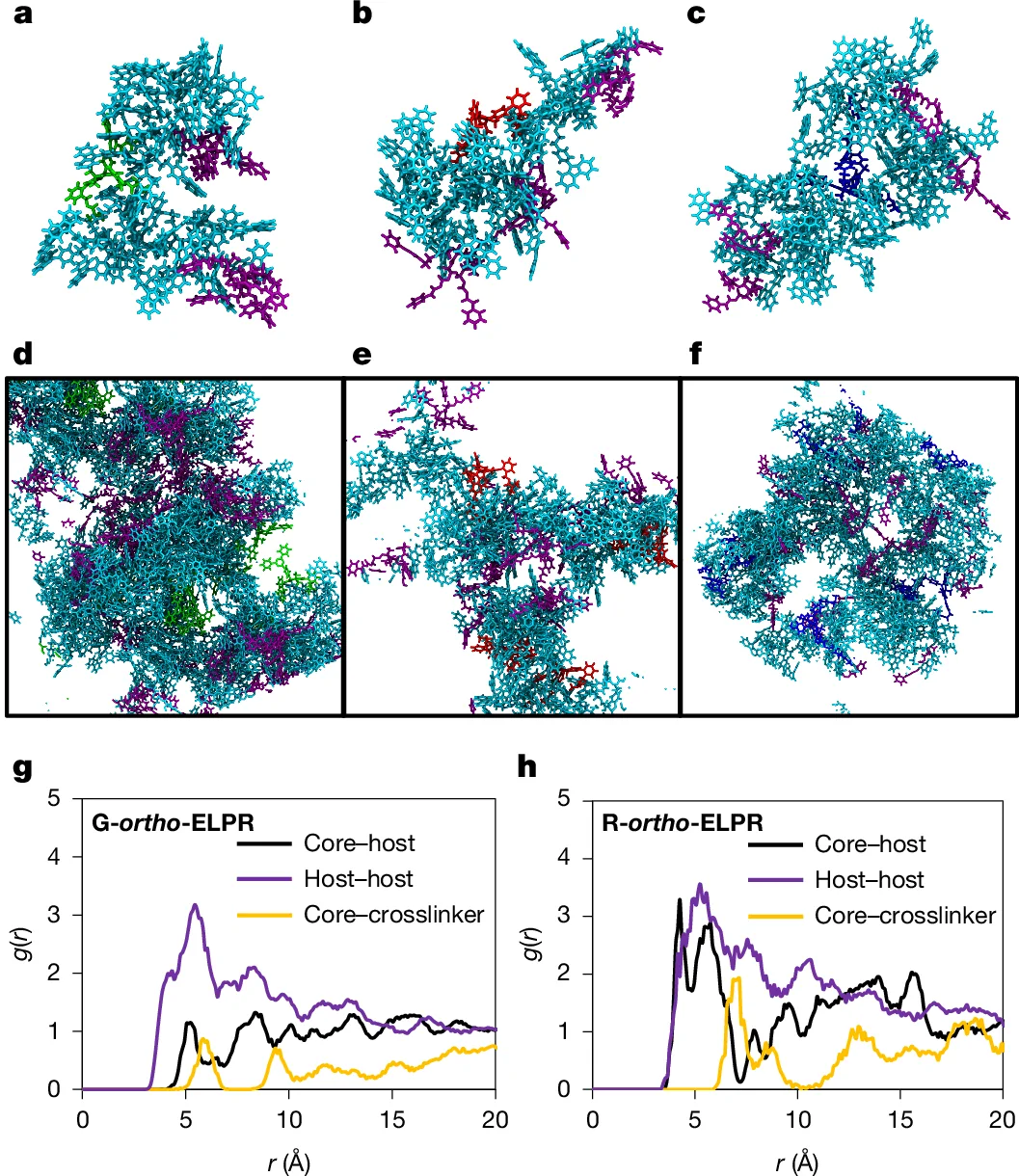
Molecular understanding of ELPR assemblies. **a**–**c**, Post-equilibrium simulation snapshots of single polymer molecules of **G-*ortho*-ELPR** (**a**), **R-*ortho*-ELPR** (**b**) and **B-*para*-ELPR** (**c**) dispersed in toluene. Green, G core; red, R core; blue, B core; cyan, host monomer; purple, crosslinker monomer. **d**–**f**, Representative post-equilibrium simulation snapshots of polymer aggregates of **G-*ortho*-ELPR** (**d**), **R-*ortho*-ELPR** (**e**) and **B-*para*-ELPR** (**f**). **g**,**h**, Extracted centre-of-mass radial distribution functions, *g*(*r*), of polymer aggregates of **G-*ortho*-ELPR** (**g**) and **R-*ortho*-ELPR** (**h**), quantifying the densities of (1) host monomers surrounding a core (core–host), (2) host monomers surrounding a host monomer (host–host) and (3) crosslinker monomers surrounding a core (core–crosslinker). Source data

To better understand the ability of the host monomer units to shell the emitter cores, classical molecular dynamics simulations were performed. Before film formation, single polymers solvated in toluene exhibited behaviour where the host monomer units would tend to aggregate, exposing the emitter cores to toluene, but largely shielding them from the crosslinker monomer units (Fig. 3a–c). As the toluene is evaporated to form films, the emitter cores largely remain shielded by the host monomer units, and the crosslinker monomer units of adjacent polymers are brought into contact where they can crosslink efficiently (Fig. 3d–f).

Centre-of-mass radial distribution functions were extracted to quantify the ability of the host shell monomers to shield the TADF emitters from the photocrosslinking moieties (Fig. 3g,h). For the green emitter, as revealed in Fig. 3g, each emitter was surrounded by 3.8 host monomers on average at a centre-of-mass distance with a maximum at 8.4 Å. Although the majority of photocrosslinking moieties were spatially distant from the emitter core, with a first peak maximum at 9.5 Å, a small number of emitter cores (12.5%) were in direct contact (centre-of-mass peak at 5.9 Å) with a single photocrosslinking moiety. For the photocrosslinking moieties themselves, each was in close contact (centre-of-mass peak maximum of 5.5 Å) with 0.8 other photocrosslinking moieties on average, rising to 2.7 on average within a radial distance of 10 Å. Similar results were found for the red and blue emitters. For example, for the red emitter, each emitter had 2 host monomers in close contact (centre-of-mass peak at 5.8 Å), and 4.2 within 10 Å. Approximately 25% had a photocrosslinking moiety in close contact (centre-of-mass peak at 7.2 Å). The photocrosslinking moieties were in close contact (centre-of-mass peak at 5.3 Å) with 0.6 other crosslinking moieties, rising to 1.8 at 10 Å.

In general, these results demonstrate that the crosslinker moieties are spatially separated from the emissive cores, and that the photocrosslinking moieties have a high probability of interacting with photocrosslinking moieties in adjacent polymer chains. Owing to the initial self-aggregation of the host monomers in toluene, there is a small probability that the emissive cores can remain solvent exposed and come into contact with photocrosslinking moieties; however, the results suggest that this occurs for only a minority of emissive cores.

## High-resolution and multicolour patterning

**Fig. 4:**
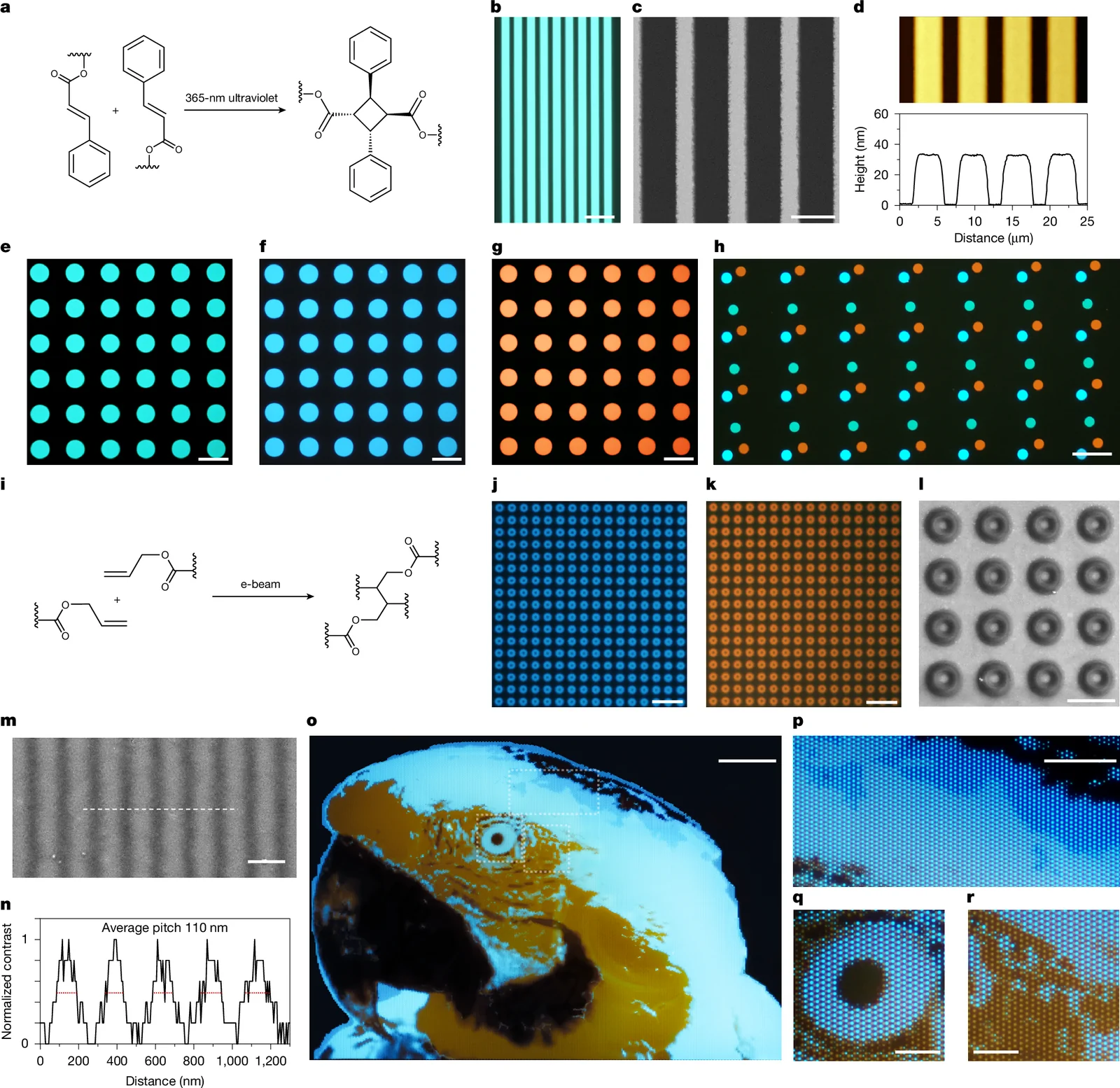
Demonstration of multicolour patterning of ELPRs via ultraviolet photolithography and EBL. **a**, Interchain photocrosslinking between two pendent cinnamoyl units triggered by 365-nm ultraviolet light. **b**–**d**, Fluorescence (**b**), SEM (**c**) and AFM (**d**) micrographs of ultraviolet-patterned lines of **G-*ortho***-**ELPR** (DP_ACz_ = 45, DP_MAc_ = 12), with L/S pitches of 4/2 μm. **e**–**h**, Fluorescence micrographs of ultraviolet photolithographic **G-*ortho***-**ELPR** (**e**), **B-*para***-**ELPR** (**f**; DP_ACz_ = 50, DP_MAc_ = 12), **R-*ortho*-ELPR** (**g**; DP_ACz_ = 42, DP_MAc_ = 9) and multicolour RGB (**h**) micropatterns fabricated on silicon substrates. **i**, Interchain crosslinking between two vinyl groups triggered by e-beam exposure. **j**–**l**, Fluorescence micrographs of nanodoughnut patterns composed of e-beam **G-*ortho*-ELPR** (**j**; DP_ACz_ = 35, DP_MAl_ = 30) and **R-*ortho***-**ELPR** (**k**; DP_ACz_ = 23, DP_MAl_ = 20), and SEM image (**l**) of the former. **m**,**n**, SEM image (**m**) and the extracted greyscale intensity along the white dashed line (**n**) for a thin layer of e-beam patterned **G-*ortho***-**ELPR** (DP_ACz_ = 35, DP_MAl_ = 30), showing L/S pitches of 110/160 nm. **o**–**r**, Fluorescence micrographs of multicolour ‘macaw parrot’ image, where individual nanodisk pixel diameters range from 150 nm to 600 nm. This pattern was fabricated by two consecutive cycles of EBL. Scale bars, 5 μm (**b**,**c**,**j**,**k**), 50 μm (**e**–**g**,**o**), 200 μm (**h**), 2 μm (**l**), 300 nm (**m**), 20 μm (**p**), 10 μm (**q**,**r**). Source data

We first examined the photopatternability of our synthesized ELPRs using ultraviolet photolithography. The ELPRs loaded with an optimized number of ultraviolet photocrosslinker units, cinnamoyl groups, were processed via negative-tone direct photolithography. The ELPR solutions in toluene were spin-coated onto silicon substrates to form solid films and thermally annealed to remove the solvent traces. These films were selectively exposed to 365-nm ultraviolet light through a photomask in vacuum contact mode. The ultraviolet-triggered [2 + 2] cycloaddition of neighbouring cinnamoyl groups (Fig. 4a) occurs exclusively within the exposed regions. Subsequent development with toluene effectively dissolved the unexposed areas, yielding high-fidelity negative-tone patterns with bright photoluminescence. We systematically examined the resulting patterns by fluorescence microscopy, scanning electron microscopy (SEM), and atomic force microscopy (AFM). For example, with **G-*ortho*-ELPR** (DP_ACz_ = 45, DP_MAc_ = 12), we successfully pushed the resolution limit to line/space (L/S) pitches of 4/2 μm with a precise film thickness of 36 nm (Fig. 4b–d). Beyond single-colour resolution, the multicolour patterns were nicely integrated with three sequential photolithographic processes, demonstrating the robust solvent orthogonality of our ELPR platform (Fig. 4e–h).

To further push the resolution beyond the diffraction limit of optical lithography, we synthesized e-beam-sensitive ELPRs incorporating allyl moieties (Fig. 4i). By leveraging the ultrashort de Broglie wavelength of accelerated electrons, we achieved unprecedented spatial control. Representative e-beam-patternable ELPRs, such as **G-*ortho*-ELPR** (DP_ACz_ = 35, DP_MAl_ = 30) and **R-*ortho*-ELPR** (DP_ACz_ = 23, DP_MAl_ = 20), were successfully patterned into nanodoughnuts with 300-nm features via direct electron-beam lithography (EBL; Fig. 4j–l). By further lowering the thickness of the spin-coated film of **G-*ortho*-ELPR**, we extended the resolution to L/S pitches of 110/160 nm (Fig. 4m,n).

**Extended Data Fig. 2:**
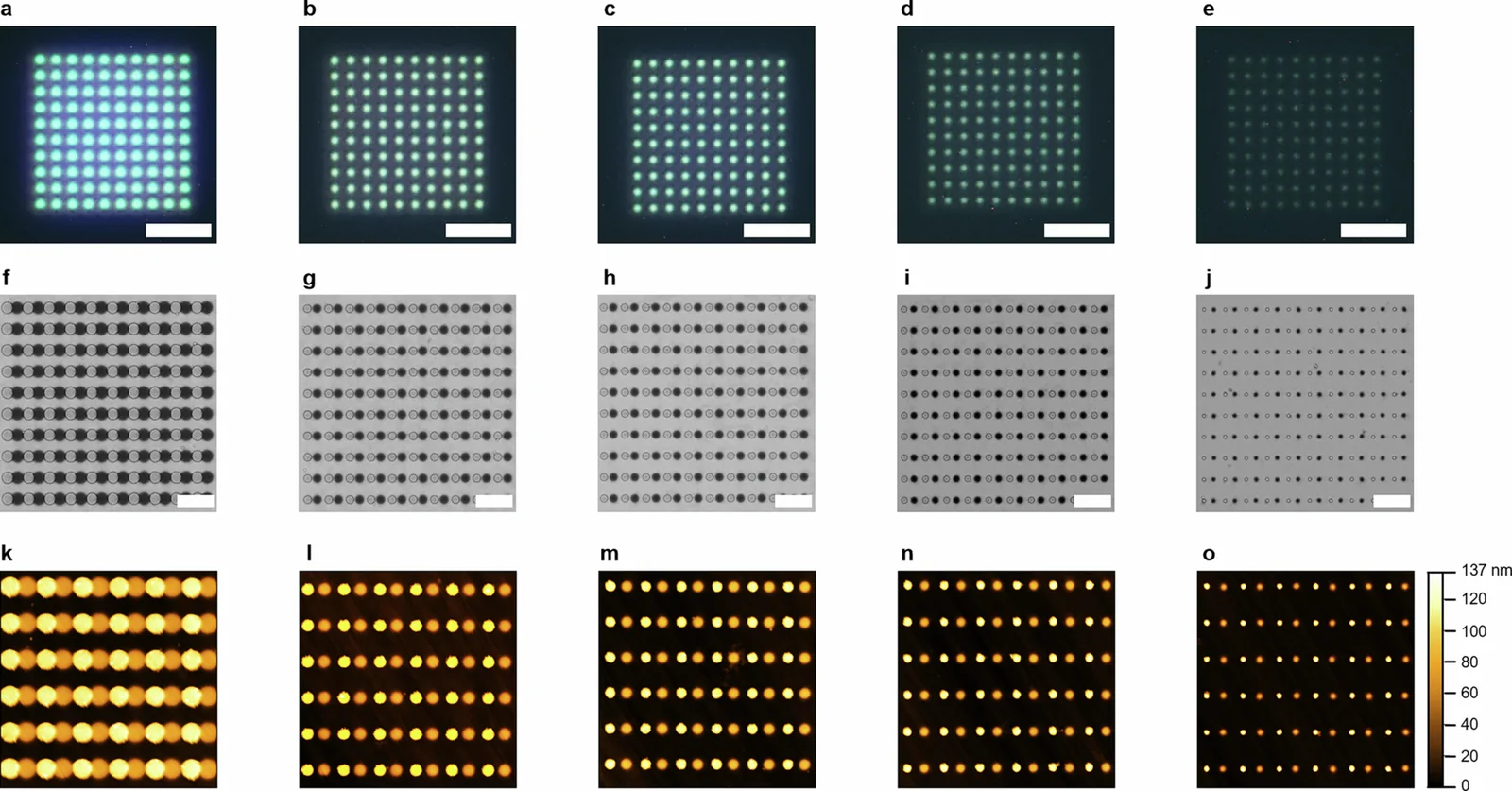
Demonstration of integrating G-*ortho*-ELPR (direct E-beam lithography) with nanoscale aluminum discs (E-beam lift-off). **a-e**, Fluorescent images; **f-j**, SEM images; **k-o**, AFM images of well-aligned nano-patterns composed of aluminum and **G-*ortho*-ELPR** nano-discs, where aluminum nano-discs were fabricated through conventional E-beam lift-off process and **G-*ortho*-ELPR** nano-discs were fabricated by direct E-beam lithography. Diameters of nano-discs are (b) 590 nm, (c) 380 nm, (d) 350 nm, (e) 230 nm, and (f) 140 nm. Scale bars of (*a-e*), 5 μm; Scale bars of (*f-j*), 2 μm.

The ELPR platform demonstrates exceptional robustness, enabling seamless integration with consecutive EBL processes and conventional lift-off processes of other functional materials. Crucially, these patterns remain intact even after prolonged exposure to aggressive chemical environments, for instance, warm acetone solution. This is exemplified in Extended Data Fig. 2, which showcases periodic nanodisk arrays composed of **G-*ortho*-ELPR** and aluminium, with precisely controlled diameters and pitches ranging from 600 nm down to 150 nm. Despite the high-energy electron exposure and subsequent solvent immersion, these nanopatterns retain intense photoluminescence with high contrast and fidelity. This validates that our single-emitter core–shell architecture effectively shields the TADF cores from both e-beam-induced degradation and chemical attack during post-processing. As a key demonstration of this nanoscale integration, we fabricated a multicoloured ‘macaw parrot’ micrograph via two consecutive EBL cycles (Fig. 4o–r). With individual pixel dimensions down to 200 nm, this represents, to our knowledge, the highest-resolution multicolour fluorescent image realized through direct lithographic patterning, enabled by the robust solvent orthogonality that facilitates high-density, multi-step integration without cross-contamination.

## Electroluminescence performance and integration

**Extended Data Fig. 3:**
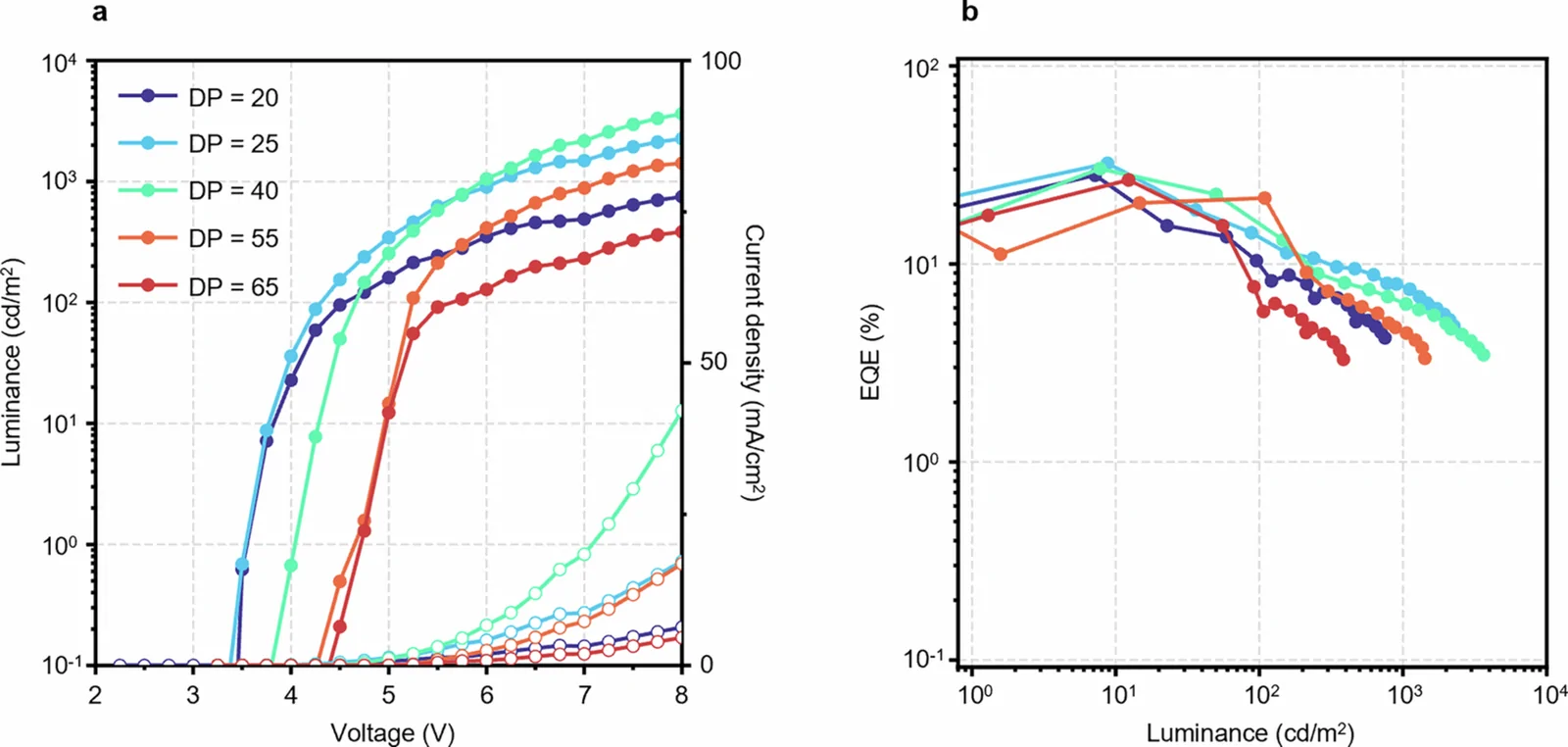
OLED performance of green-emitting self-hosted polymers G-*ortho*-P-deBr with different DP_ACz_. **a**, Luminance and current density values as a function of voltage for **G-*ortho*-P-deBr** with different DPs (DP_ACz_ = 20, 25, 45, 55, 65). **b**, EQE versus luminance characteristics. The emitter doping concentration can be controlled by changing the feeding ratio of ACz to TADF initiator for ATRP. In this case, the **G-*ortho*-P-deBr** with DP = 45 exhibited the highest max. EQE (30%).

**Extended Data Fig. 4:**
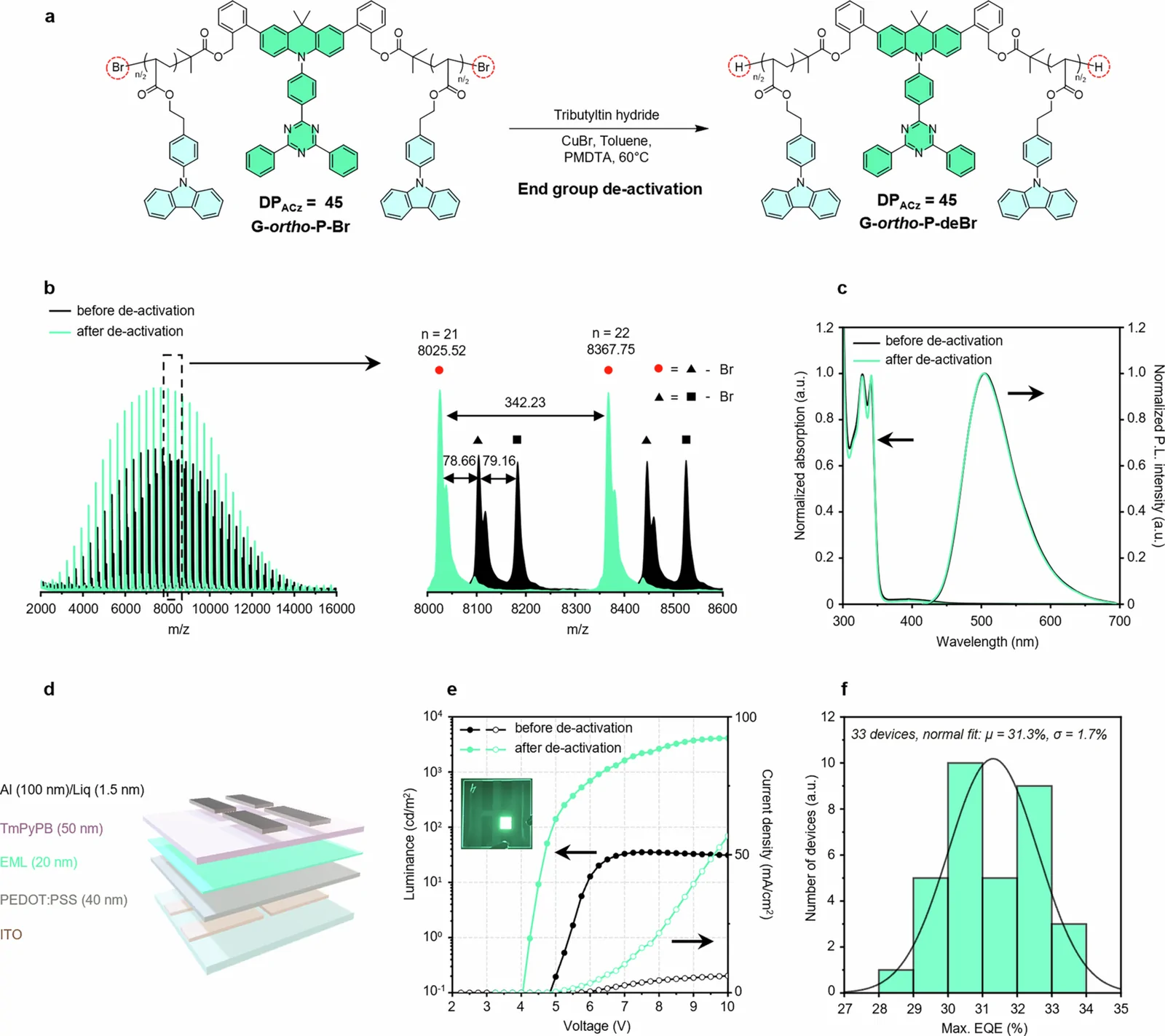
EL enhancement of green-emitting self-hosted TADF polymer after end-group deactivation. **a**, Debromination of **G-*ortho*-P** is shown as a model reaction. **b**, MALDI-TOF mass spectra for **G-*ortho*-P-Br** and **G-*ortho*-P-deBr**. The enlarged mass spectra tracks whether the Br end groups are removed. **c**, Normalized UV-vis absorption and PL spectra of **G-*ortho*-P-Br** and **G-*ortho*-P-deBr** thin films. **d**, OLED device architecture with either **G-*ortho*-P-Br** or **G-*ortho*-P-deBr** as EML. e, Comparison of luminance and current density as a function of voltage for polymer OLEDs before and after end-group deactivation. Inset: EL photograph of OLED device using **G-*ortho*-P-deBr** as EML. f, Histograms of max. EQE for the OLED devices using **G-*ortho*-P-deBr** as EML.

Before evaluating the electroluminescence of the ELPRs, we first investigated and optimized the device performance of their self-hosted TADF precursors (the P-series; Extended Data Fig. 3). These polymers initially possess dormant bromide end groups, a remnant of the ATRP process. We hypothesized that these reactive moieties may be activated under the high electric fields present during OLED operation, potentially acting as deleterious charge traps or quenching sites. To validate our hypothesis, we have deactivated the end groups by debrominating all the self-hosted TADF polymers, and matrix-assisted laser desorption/ionization time-of-flight (MALDI-TOF) mass spectroscopy was used to confirm the successful reaction (Extended Data Fig. 4b and Supplementary Note 15). For instance, the green self-hosted polymer before (**G-*ortho*-P-Br**) and after (**G-*ortho*-P-deBr**) debromination showed distinct characteristic peaks; **G-*ortho*-P-Br** exhibited periodic mass peaks corresponding to dormant bromide end groups, whereas **G-*ortho*-P-deBr** did not. Both **G-*ortho*-P-Br** and **G-*ortho*-P-deBr** preserved the characteristic charge-transfer absorption peak in ultraviolet–visible spectra, indicating that the emitter core remained intact before and after debromination (Extended Data Fig. 4c). Furthermore, electrochemical analysis revealed that the highest occupied molecular orbital of **G-*ortho*-P** is consistent with the parent TADF emitter unit, DMAC-TRZ (Supplementary Note 16 and Table 7). **G-*ortho*-P-deBr** exhibits robust thermal stability, with a decomposition temperature (*T*_d_, defined as a temperature account for 5% weight loss of material) of 350 °C. This high thermal robustness ensures reliable and uniform film formation during spin-coating and subsequent processing by preserving polymer integrity and mitigating thermally induced morphological instability^45^ (Supplementary Note 17). Interestingly, after debromination, **G-*ortho*-P-deBr** exhibited a shorter *τ*_DF_ of 1.26 μs, compared with 1.60 μs for **G-*ortho*-P-Br** in thin-film TRPL measurements (Supplementary Note 13 and Table 3).

**Fig. 5:**
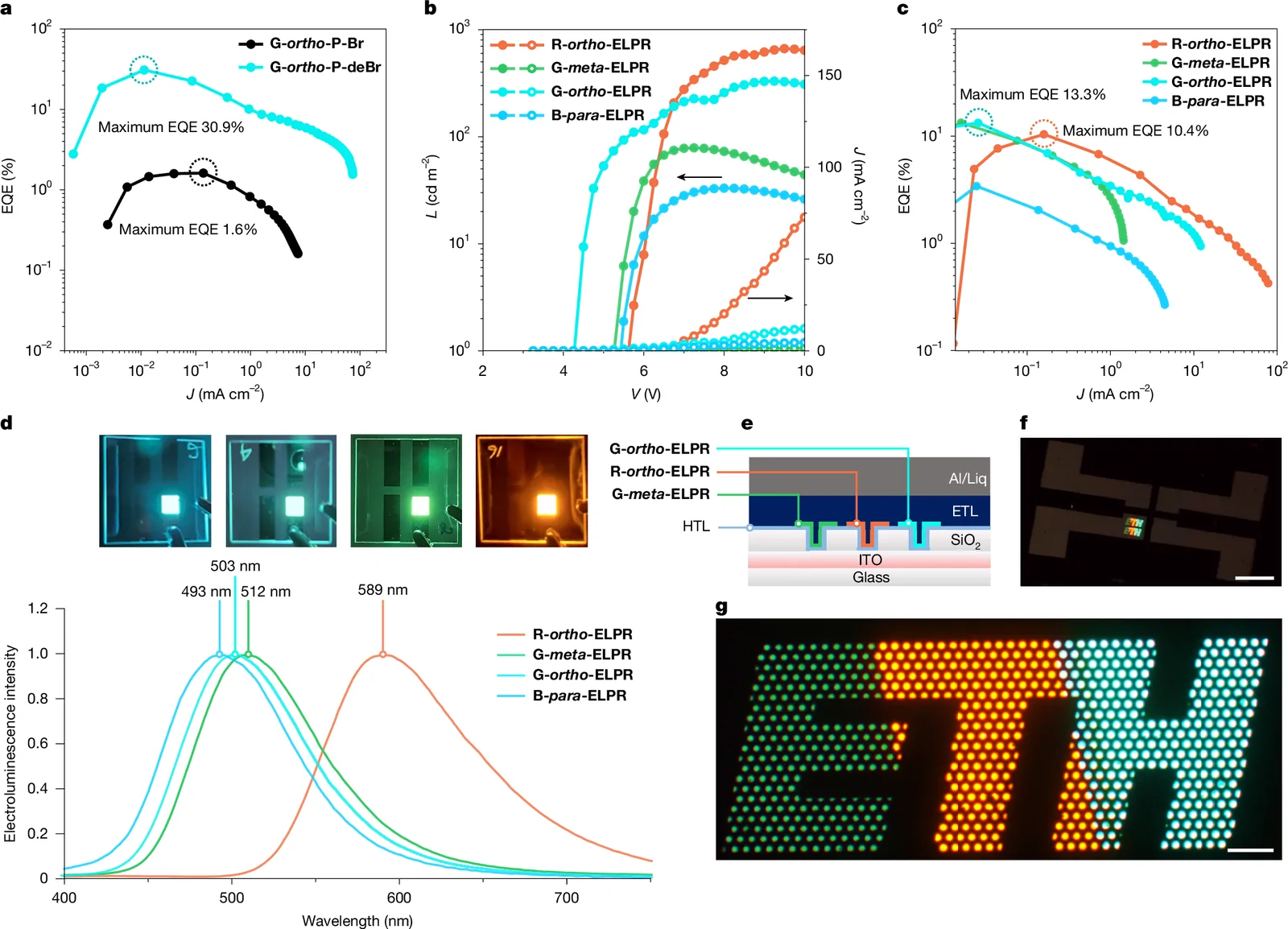
ELPR OLED performance and multicolour integration. **a**, Comparison of OLED characteristics for self-hosted polymers (P-series) before (**G-*ortho*-P-Br**) and after (**G-*ortho*-P-deBr**) end-group deactivation (DP_ACz_ = 40). Maximum EQE values markedly increased from 1.6% to 30.3%. **b**,**c**, OLED characteristics, including luminance (*L*) and current density (*J*) as a function of voltage (**b**), and EQE as a function of *L* (**c**) for various ELPRs after photocrosslinking and end-group deactivation. **d**, Electroluminescence spectra for OLEDs made by various ELPRs measured at 7 V, revealing that the emission peak wavelength can be finely tuned by linker engineering, with *ortho*-linkers inducing blueshift. Inset photographs from left to right: **B-*para*-ELPR** (DP_ACz_ = 50, DP_MAc_ = 12), **G-*ortho*-ELPR** (DP_ACz_ = 45, DP_MAc_ = 12), **G-*meta*-ELPR** (DP_ACz _= 40, DP_MAc_ = 12) and **R-*ortho*-ELPR** (DP_ACz_ = 42, DP_MAc_ = 9). **e**, Schematic diagram illustrating the device architecture of multicolour OLED pixels, where multicolour ELPR EMLs were photo-patterned through three consecutive cycles of direct photolithography. **f**,**g**, Electroluminescence photograph (**f**) and micrograph (**g**) of a working multicolour micro-OLED device taken at 6 V. The diameter of individual circular pixels is 30 µm. Scale bars, 4 mm (**f**), 200 μm (**g**). Source data

**Extended Data Fig. 5:**
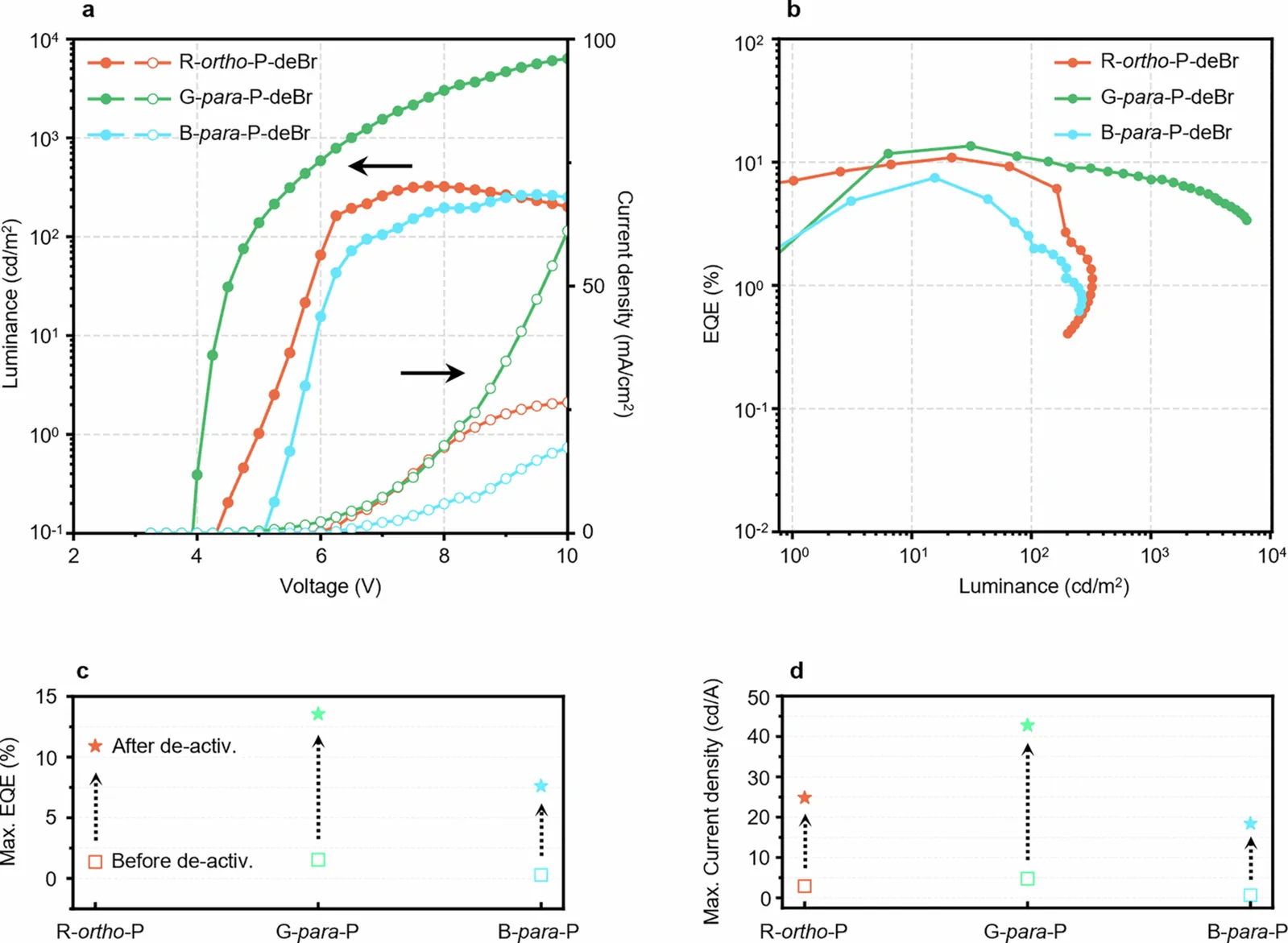
OLED performance of various self-hosted TADF polymers before and after end-group deactivation. **a**,**b**, Luminance and current density as a function of voltage (**a**) and EQE as a function of luminance (**b**) for the self-hosted TADF polymers after debromination. **c**,**d**, Comparison of maximum EQE (**c**) and current density (**d**) for polymers before and after end-group deactivation. DPs of ACz monomers for **R-*ortho*-P-deBr**, **G-*para*-P-deBr**, and **B-*para*-P-deBr** are 42, 20, and 50 respectively.

Finally, we compared the OLED performance of **G-*ortho*-P-Br** and **G-*ortho*-P-deBr**. Strikingly, deactivation led to an 18-fold and 17-fold enhancement in maximum EQEs and current efficiencies, surging from 1.6% to over 30.3% and from 4.25 cd A^−1^ to 75.44 cd A^−1^, respectively (Fig. 5a and Supplementary Fig. 18). Notably, the EQE of the solution-processed polymer OLED based on **G-*ortho*-P-deBr** is slightly higher than that for the control TADF OLEDs fabricated via vacuum evaporation of parent molecules (26.5%)^35^, indicating the critical role of covalent molecular design of single-emitter TADF polymers. This strategy is also effective for self-hosted TADF precursors with red- and blue-emitting cores. The OLED performance of all self-hosted TADF precursors, including EQE and luminance, were markedly enhanced after the end-group deactivation (Extended Data Fig. 5). We believe that this approach could serve as a universal strategy for developing highly efficient electroluminescent polymers via controlled radical polymerization.

Leveraging this critical insight, we characterized the fully functionalized ELPRs in a standard OLED architecture, where the emissive layer was ultraviolet-crosslinked before the deposition of the electron-transport layer (Fig. 5b). Notably, **G-*ortho*-ELPR** (DP_ACz_ = 45; DP_MAc_ = 12) achieved a peak EQE of 13.3% (Fig. 5b,c). This represents a transformative advancement for directly photolithographically patternable organic emitters, which have been historically limited to EQEs below 2% (refs. ^21,22^). Furthermore, through linker engineering at the TADF core, we demonstrated nearly multicolour tunability across the visible spectrum while maintaining robust electroluminescence performance (Fig. 5d).

To showcase the potential for high-density integration, we fabricated a miniaturized multicolour OLED array featuring 30-µm pixels. The device architecture utilized a common indium-tin-oxide anode with pixels defined by a photolithographically patterned silicon dioxide (SiO_2_) passivation layer, which also served as an interlayer dielectric (Fig. 5e). Exploiting the precise photopatternability and solvent orthogonality of our ELPRs, we sequentially deposited blue, green and red pixels to form the letters ‘ETH’ through three consecutive lithographic cycles. Upon completion with common electron-transport and cathode layers, the multicolour array exhibited uniform and bright electroluminescence at 6 V (Fig. 5f,g). This demonstration of monolithic, micrometre-scale multicolour integration unlocks the scalability of our ELPR platform for next-generation microdisplays and on-chip photonic integration.

We further examined the operational lifetimes (LT_50_; time required for the luminance to decay to 50% of its initial value) of our devices (Supplementary Note 19). Devices using poly(3,4-ethylenedioxythiophene):poly(styrenesulfonate) (PEDOT:PSS) showed pronounced degradation, probably owing to its acidic nature interacting with the EML during ultraviolet crosslinking^46,47^. Conversely, using poly[bis(4-phenyl)(2,4,6-trimethylphenyl)amine] (PTAA) as the sole hole-transporting layer (HTL) significantly improved robustness. Although absolute lifetimes are currently limited by trace-metal impurities, we demonstrated that halving the residual copper content via intensive dialysis and optimizing the transport layer architectures extended the LT_50_ of **G-*ortho*-P-deBr** devices to 172 min at a constant current density of 2.4 mA cm^−2^ (Supplementary Fig. 63). A parallel stability enhancement (LT_50_ of 146 min) was observed for the crosslinked **G-*ortho*-ELPR** system. We anticipate that device stability can be further improved by minimizing copper residues among the ELPRs through regenerated^48^ or organocatalysed^49^ ATRP synthesis or through multiple dialysis cycles.

Although these findings highlight that the extrinsic factors, including HTLs and metal impurities, significantly limit current device operational stability, the potential contributions of intrinsic degradation mechanisms, such as those related to the dual-functional polymer architecture and crosslinkable groups, remain to be independently evaluated. In addition, it should be noted that a distinction must be made between peak high-current operation and continuous stable operation. Although our measurements demonstrate that the devices can sustain transient current densities well over 100 mA cm^−2^, continuous long-term operation is currently restricted to 10 mA cm^−2^ or below to mitigate excessive Joule heating and prevent premature electrical breakdown.

## Conclusion and outlook

We have developed a molecular design strategy that elegantly fuses efficient electroluminescence with photoresists—a class of materials traditionally regarded as insulating and non-functional. Despite the conceptual leap, a performance gap remains between the directly patterned ELPRs and state-of-the-art vacuum-evaporated OLEDs, particularly regarding operational lifetimes. However, the modular nature of our controlled polymerization-based star polymer architecture provides a clear and robust strategic roadmap for future optimization. Our findings establish ‘functional photoresists’ as a universal platform to synergize molecular design with nanofabrication. By encoding lithographic capability directly into functional backbones with advanced macromolecular control, this strategy could address the ‘compatibility–resolution’ bottleneck that has long hindered organic electronics. Envisioning Moore’s law extended to the organic regime, the functional-photoresist platform enables monolithic integration of ultrahigh-density organic integrated photonics and circuits.

## Methods

### End-group deactivation of TADF polymers

The bromo end groups of ATRP-synthesized TADF polymers were removed via hydrogenation. In a typical procedure, **G-*ortho*-P** (1.0 equiv.) and copper(I) bromide (CuBr; 2 equiv.) were charged into a Schlenk flask and evacuated for 20 min, followed by 3 vacuum–argon cycles. Next, 1,1,4,7,7-pentamethyldiethylenetriamine (PMDETA; 2.0 equiv.), degassed via argon sparging for 10 min, was injected into the flask, and the resulting mixture was stirred at room temperature for 30 min under an argon atmosphere. Tributyltin hydride (2.0 equiv.) was then injected into the Schlenk flask under argon. The flask was placed in an oil bath at 60 °C for 2 h with stirring. After a specific time, the Schlenk flask was cooled to room temperature to stop the reaction. Then the viscous mixture was dissolved in tetrahydrofuran, filtered through a column filled with activated basic Al_2_O_3_, precipitated once in hexane and twice in methanol, and then dried under vacuum overnight. Final solid powders were obtained as the product and ready for further use after characterization with NMR, size-exclusion chromatography and MALDI-TOF.

### Block copolymerization affording ELPRs

ELPRs were synthesized via ATRP using the self-hosted precursor polymers as macroinitiators. The crosslinker loading was modulated by varying the stoichiometric feed ratio of the crosslinkable monomer (MAc or MAl) to the macroinitiator. In a representative synthesis of **G-*ortho*-ELPR**, **G-*ortho*-P** (1.0 equiv.) and CuBr (1.5 equiv.) were charged into a flame-dried Schlenk flask and subjected to 3 vacuum–argon cycles. Separately, a solution of MAc monomer and PMDETA (1.5 equiv.) in toluene was degassed via argon sparging for 20 min. The degassed monomer/ligand solution was then transferred to the Schlenk flask via syringe under argon. The polymerization was conducted at 60 °C and subsequently stopped by cooling the flask to room temperature. The resulting viscous mixture was diluted with tetrahydrofuran and filtered through a column filled with activated basic Al_2_O_3_ to remove the residue copper ions. The final block copolymer was isolated by precipitation once in hexane and twice in methanol, followed by drying under vacuum overnight to yield solid powders of **G-*ortho*-ELPR**.

### Direct photolithography

In a typical direct photo-patterning process, a certain amount of ELPR polymers were dissolved in toluene and filtered through a 0.22-µm PTFE membrane, affording 10–25 mg ml^−1^ precursor solution. Then this ELPR precursor can be spin-coated either on silicon wafer for photo-pattern demonstration or on top of the HTL layer for OLED device fabrication. The thickness of spin-coated ELPR films can be tuned by changing spin-coating speed and/or the precursor concentration of the ELPR. The mask contact aligner exposure was performed on an ABM ultraviolet mask aligner through a 4-inch quartz photomask. The 405–365 nm exposure mirror was installed on an ABM aligner that allows i-line exposure. The typical dose range used for ELPR patterning is 6,000–8,500 mJ cm^−2^. The development was performed by rinsing the substrate with toluene to remove the un-crosslinked regions.

### Direct e-beam lithography

The film deposition of ELPR polymers for direct EBL is the same process as that of direct ultraviolet photolithography. ELPR solution concentration can be varied between 10 mg ml^−1^ to 25 mg ml^−1^ if thickness tuning is needed. The e-beam exposure was performed on EBPG5200, Raith with a fixed 100 keV voltage from the Binnig and Rohrer Nanotechnology Center (BRNC) at the IBM research centre in Zurich. The optimal dose for each single-coloured ELPR is different, but typically ranges from 100 μC cm^−2^ to 500 μC cm^−2^. The development was performed by rinsing the substrate with toluene to remove the un-crosslinked regions. The aluminium nanodisks were patterned with a conventional poly(methyl methacrylate) and methacrylic acid (PMMA/MMA) bilayered e-beam lift-off process.

### OLED fabrication

For pursuing the best OLED performance, all the synthesized polymers used as the EML underwent end-group deactivation before device integration. Indium-tin-oxide-coated glass substrates were cleaned and treated with oxygen plasma. For bulk devices, a PEDOT:PSS blend (Clevios AI 4083/CH 8000 mix) was spin-coated (4,000 rpm, 50 s) with adjustable relative ratio to a thickness range of 40–80 nm and annealed at 150 °C for 15 min on a laminar flow bench. Subsequently, the devices were transferred to a nitrogen-filled glovebox to deposit an optional HTL and polymer EML via spin-coating. For photo-patterned devices, a PTAA layer (5 mg ml^−1^ in toluene) was spin-coated (4,000 rpm, 50 s) and annealed at 120 °C for 10 min. To understand the role of the hole-injection layer and the HTLs on the operational stability of the OLED devices, we used a hole-injection material, nickel oxide (NiO_*x*_), and two other hole-transport materials, *N*4,*N*4′-di(naphthalen-1-yl)-*N*4,*N*4′-bis(4-vinylphenyl)biphenyl-4,4′-diamine (v-NPB) and poly(9-vinylcarbazole (PVK). Subsequently, a 20-nm TADF polymer EML was spin-coated (2,000 rpm, 50 s). In the case of ELPRs, the EML was crosslinked via 365-nm ultraviolet exposure. For the multicolour patterning device (ETH logo), each ELPR layer was ultraviolet-irradiated through a photomask selectively and developed in toluene; this process was repeated for additional colours to achieve pixelated EMLs. Finally, the substrates were transferred into a physical vapour deposition chamber, and a 50-nm 1,3,5-tri(*m*-pyridin-3-ylphenyl)benzene (TmPyPB) or 1,3,5-tris(1-phenyl-1H-benzimidazol-2-yl)benzene (TPBi) electron-transport layer, a 1.5-nm 8-hydroxyquinolinolatolithium interface layer, and a 100-nm aluminium cathode were sequentially deposited via thermal evaporation through a shadow mask at a vacuum level of 7.5 × 10^−8^ torr.

### Device encapsulation

Following the thermal deposition of the aluminium electrode, the fabricated devices were immediately transferred without atmospheric exposure into an integrated, dry-nitrogen-filled glovebox (O_2_ < 0.1 ppm, H_2_O < 0.1 ppm) for hermetic encapsulation. The devices were sealed using a chemically etched aluminosilicate cavity cover glass integrated with a pre-conditioned desiccant label to scavenge residual moisture. Am ultraviolet-curable NOA 61 epoxy resin sealant was precisely dispensed along the perimeter of the cover glass. The encapsulation architecture was then pressed securely against the device substrate and cured under ultraviolet radiation (*λ* = 365 nm) for 30 s. A pixel-aligned shadow mask was utilized during exposure to completely shield the active polymer emissive area from ultraviolet-induced degradation.

### Device operational lifetime optimization

We have further examined the operational lifetimes (LT_50_; time required for the luminance to decay to 50% of its initial value) of our polymeric OLED devices incorporating spin-coated **G-*ortho*-P-deBr** (P-series) and **G-*ortho*-ELPR** (ultraviolet-crosslinked) EMLs deposited on different underlying HTLs, namely, PEDOT:PSS, PEDOT:PSS/PTAA, and PTAA (Supplementary Note 19). Devices with the EML deposited directly on PEDOT:PSS showed the lowest LT_50_, where the LT_50_ of **G-*ortho*-ELPR** was reduced by approximately 50% compared with the LT_50_ of **G-*ortho*-P-deBr**. This pronounced degradation is attributed to the acidic PSS component of PEDOT:PSS layer^46,47^, which is likely to chemically interact with the EML during ultraviolet crosslinking. Notably, devices using PTAA as the sole HTL showed decent robustness, exhibiting a nominal approximately 4% decrease in LT_50_ for the crosslinked ELPR compared with the parent P-series counterpart.

Although the absolute lifetime remains limited at this stage, with the best-performing crosslinked **G-*ortho*-ELPR** devices reaching an LT_50_ of approximately 70 min at a constant driving current density of 1 mA cm^−2^ (Supplementary Fig. 53), this is probably associated with trace impurities in polymers and potential weak chemical bonds. We anticipate that device stability can be further improved by (1) using alternative controlled polymerization methods and (2) systematically optimizing the hole- and electron-transporting layer materials and architectures. By halving the residual copper content via intensive dialysis (Supplementary Fig. 62 and Supplementary Table 9) and replacing the baseline PTAA/TmPyPB transport layers with crosslinkable v-NPB and TPBi, respectively, the optimized **G-*ortho*-P-deBr** device showed an extended LT_50_ of 172 min at a constant current density of 2.4 mA cm^−2^ (Supplementary Fig. 63). This represents a 135% lifetime improvement over the control device, notably achieved under a 2.4-fold higher operating current. Furthermore, under elevated driving currents of 5.0 mA cm^−2^ and 10 mA cm^−2^, the devices recorded solid LT_50_ values of 108 min and 30 min, respectively (Supplementary Figs. 63 and 65). A parallel stability enhancement was observed for the sister system, where the dialysed **G-*ortho*-ELPR** device achieved an LT_50_ of 146 min (Supplementary Fig. 63d).

We observed a reduction of *Φ*_PL_ upon the integration of crosslinkable moieties. This reduction, coupled with limitations in EML conductivity and peak luminance, highlights the inherent complexity of balancing photochemical functionality with high-performance optoelectronics. The observed quenching suggests that the local dielectric environment or potential charge transfer induced by the crosslinker periphery still poses challenges to the radiative efficiency of the core. In addition, more detailed study is required to elucidate the effects of ATRP residues to the device operational stability, including the optimization of device architecture for long-term stress.

### Molecular dynamics simulations

The polymer systems were constructed for molecular dynamics simulations using PolyConstruct^50^, with CHARMM CGenFF force-field parameters generated using the online tool (www.cgenff.com)^51^. Each polymer system consisted of crosslinker monomer units (purple) with Br-atom termini, host monomer units (cyan) and initiator emitter cores (green, red and blue). The green polymer comprised the initiator with 2 polymer branches of 20 cyan monomers and 5 purple monomers each. The red polymer comprised the initiator with 3 polymer branches; 2 of 13 cyan and 3 purple monomers each, and one of 14 cyan and 4 purple monomers. The blue polymer comprised the initiator with 4 polymer branches 15 cyan and 2 purple monomers each. Initially, individual polymers were solvated in 100 × 100 × 100 Å^3^ boxes of toluene constructed using PACKMOL^52^, and after at least 20 ns of equilibration, multiple polymer systems of the same type were packed into a single box of the same dimensions, and the toluene was sequentially evaporated in a manner similar to our previous work^53^. All molecular dynamics simulations were performed using the GROMACS 2024.1 in the isothermal–isobaric (NPT) ensemble with the v-rescale thermostat and c-rescale barostat to maintain temperature at 298.15 K and pressure at 1 bar^54^. Pairwise electrostatic interactions were cut off at 12 Å, with a particle mesh Ewald treatment of long-range interactions, and van der Waals interactions were reduced to 0 between 10 Å and 12 Å using a switching function. Covalent bonds involving hydrogen were constrained using the LINCS algorithm. A time step of 2 fs was used, with simulation snapshots saved every 10 ps. Radial distribution functions and corresponding integrals were calculated using VMD 1.9.3^55^.

## Online content

Any methods, additional references, Nature Portfolio reporting summaries, source data, extended data, supplementary information, acknowledgements, peer review information; details of author contributions and competing interests; and statements of data and code availability are available at https://doi.org/10.1038/s41586-026-11042-0.

## Supplementary information

Supplementary Information Supplementary Notes 1–27, Tables 1–12 and References.

Peer Review File

## Source data

Source Data Figs. 2–5.

## Data Availability

The data that support the findings are available upon request; please contact C.-J.S. Source data are provided with this paper.
