# Supplementary material for: Electroluminescent photoresists extending lithographic scaling to OLEDs
Source: Nature. 2026 Sep 16;658(8134):78–85. doi: 10.1038/s41586-026-11042-0 (PMC13626961; doi:10.1038/s41586-026-11042-0)
Supplement: Supplementary file 1 [file 41586_2026_11042_MOESM1_ESM.pdf]

nature portfolio

https://doi.org/10.1038/s41586-026-11042-0

Supplementary information

Electroluminescent photoresists extending lithographic scaling to OLEDs

In the format provided by the authors and unedited

Supplementary Information for

Electroluminescent Photoresists Extending Lithographic Scaling to OLEDs

Shao-Wei Lo,[1] Sunil B. Shivarudraiah,[1] Zhan-Hong Lin,[1] Patrick Helbling,[1] Lifei Song,[1] Jiachen Wang,[1] Miguel Nouman,[1] Fuze Jiang,[2] Gerrit Stemmler,[1] Donato Maria Carretta,[1] Mei-Nung Chen,[1,3] Jiayi Zhu,[1] Sudhir Kumar,[1] Hua Wang,[2] Andrew J. Christofferson,[4] Yinyin Bao,[1,5*] and Chih-Jen Shih[1*]

[1] Institute for Chemical and Bioengineering, ETH Zürich, 8093 Zürich, Switzerland

[2] School of Information Technology and Electrical Engineering, ETH Zürich, Zürich, Switzerland

[3] Department of Chemical Engineering, National Taiwan University of Science and Technology, Taipei, 10607, Taiwan

[4] School of Science, STEM College, RMIT University, Melbourne, VIC 3001, Australia

[5] Department of Chemistry, Faculty of Science, University of Helsinki, 00014 Helsinki, Finland

[*]All correspondence should be addressed to Y.B. (yinyin.bao@helsinki.fi) and C.J.S (chih-jen.shih@chem.ethz.ch)

Table of Contents

Supplementary Note 1. | Chemicals and general information 3

Supplementary Note 2. | Synthetic route of green-emitting TADF initiator for ATRP 7

Supplementary Note 3 | Synthetic route of red-emitting TADF initiator for ATRP 14

Supplementary Note 4 | Synthetic route of blue-TADF-based initiator for ATRP 26

Supplementary Note 5 | Synthetic route of AcryCz host monomer for ATRP 33

Supplementary Note 6 | Synthetic route of cinnamyl photocrosslinkable monomer for ATRP 37

Supplementary Note 7 | NMR characterization of green-, red-, and blue-emitting self-hosted TADF polymers with different degree of polymerization (DP)...39

Supplementary Note 8 | NMR characterization of the green-, red-, and blue-emitting self-hosted TADF polymers before and after end group de-activation 42

Supplementary Note 9 | NMR characterization of the green-, red-, and blue-emitting ELPR polymers before and after end group de-activation..45

Supplementary Note 10 | NMR characterization of the green-, red-, and blue-emitting E-beam patternable polymers before and after end group de-activation 48

Supplementary Note 11 | Host monomer engineering for self-hosted G-*ortho*-Ps.................51

Supplementary Table 1 | Summary of OLED performance for host engineering 52

Supplementary Note 12 | Size-Exclusion Chromatography (SEC) curves of selected TADF polymers...53

Supplementary Table 2 | SEC data of selected TADF polymers. 54

Supplementary Note 13 | Lifetime decay curves 55

Supplementary Table 3 | Exciton lifetimes of selected TADF polymers in toluene solution with/without argon degassing 61

Supplementary Table 4 | Exciton lifetimes of selected TADF polymer thin films coated on quartz substrates measured at room temperature 62

Supplementary Table 5 | Linker engineering for finely tuning emission properties of the green-emitting TADF polymers. 63

Supplementary Note 14 | Absolute thin-film PLQYs histograms of self-hosted TADF polymers, ELPRs before and after UV crosslinking measured under nitrogen atmosphere. .64

Supplementary Table 6 | Summary of photophysical data of selected polymer thin films......65

Supplementary Note 15 | Mass data analysis of selected TADF polymers 66

Supplementary Note 16 | Electrochemical characterization 71

Supplementary Table 7 | Electrochemical characterization data of G-*ortho*-P before and after end group de-activation...71

Supplementary Note 17 | Thermal stability characterization of G-*ortho*-P-deBr 72

Supplementary Note 18 | OLED current efficiency enhancement of green-emitting self-hosted TADF polymer after end-group deactivation. 73

Supplementary Note 19 | Operational stability performance of green-emitting self-hosted TADF polymer and ELPR with different device architecture...74

Supplementary Note 20 | Extraction of photophysical kinetic parameters for green-emitting TADF polymers 75

Supplementary Table 8 | Photophysical characteristics and rate constants of self-hosted TADF polymers measured in 0.1 mM toluene solution....76

Supplementary Note 21 | Reliability and reproducibility characterization 77

Supplementary Note 22 | Microscopic characterization 81

Supplementary Note 23 | Quantification of copper residue within TADF polymer inherited from ATRP synthesis....84

Supplementary Table 9 | Quantification of copper residue concentration in the G-*ortho*-P-deBr polymer sample before and after dialysis. 85

Supplementary Note 24 | Optimized operational stability lifetime by dialysis treatment and device optimization....86

Supplementary Note 25 | OLED devices optimized for high brightness and high current density....87

Supplementary Note 26 | Nyquist plots & frequency response characterization....91

Supplementary Table 10 | Summary of the fitting parameters of the Nyquist plots extracted from the equivalent circuit model. 93

Supplementary Note 27 | Applicability of end group de-activation for red/blue emitting polymers....95

Supplementary Table 11 | Quantitative comparison of pixelation strategies for emissive display technologies, including inorganic QD-LEDs, III-V micro-LEDs, perovskite LEDs, and ELPR OLEDs. 98

Supplementary Table 12 |. Temperature dependent TRPL lifetimes and PL intensity of green-emitting self-hosted TADF polymer after (G-*ortho*-P-deBr) end group de-activation with DP_{ACz} = 45 in thin fim. 101

References....102

Supplementary Note 1. | Chemicals and general information

1,10-Phenanthroline (98%, Apollo Scientific Ltd), 4-Bromo-1,8-naphthalic anhydride (96%, Apollo Scientific Ltd), 4-Aminophenethyl alcohol (97%, Thermo Scientific - Acros Organics), tert-Butyldimethylchlorosilane (TBDMS-Cl, 97%, Fluorochem Ltd), Imidazole (>99%, Sigma-Aldrich), 9,9-Dimethyl-9,10-dihydroacridine (DMAC, 98%, BLDpharm), N-Bromosuccinimide (NBS, 99%, Sigma-Aldrich), Cinnamoyl chloride (97%, BLDpharm), 2-Hydroxyethyl methacrylate (>99%, contains <50 ppm monomethyl ether hydroquinone as inhibitor, Sigma-Aldrich), 4-Methoxyphenol (98%, BLDpharm), Triethylamine anhydrous (99%, Fluorochem Ltd), 2-(4-Bromophenyl)-4,6-diphenyl-1,3,5-triazine (Br-TRZ, 98%, BLDpharm), 4-(4,4,5,5-Tetramethyl-1,3,2-dioxaborolan-2-yl)benzeneethanol (*para*-spacer, 95%, BLDpharm), 2-(3-(4,4,5,5-Tetramethyl-1,3,2-dioxaborolan-2-yl)phenyl)ethanol (*meta*-spacer, 97%, BLDpharm), (2-(Hydroxymethyl)phenyl)boronic acid (*ortho*-spacer, 98%, BLDpharm), 9-(4-Bromophenyl)-9H-carbazole (98%, BLDpharm), Acryloyl chloride (> 97%, Merck Millipore), Sodium hydride (95%, Sigma-Aldrich), 4,4'-Difluorobiphenyl sulphone (98%, Apollo Scientific Ltd), Tetrakis(triphenylphosphine)palladium(0) (99.9%, Thermo Scientific - Acros Organics), Tetrabutylammonium fluoride solution (TBAF, 1.0M in THF, Sigma-Aldrich), 2-Bromo-2-methylpropanoyl bromide (98%, Sigma-Aldrich), Dibutyltin oxide (98%, Thermo Scientific - Acros Organics), *Tert*-Butyldimethylsilyl chloride (98%, Fluorochem Ltd), Carbazole (Cz, 96%, Thermo Scientific - Acros Organics), 4-Bromophenethyl alcohol (99%, Sigma-Aldrich), 1,1,4,7,7-pentamethyldiethylenetriamine (PMDETA, 99+%, Thermo Scientific - Acros Organics), Allyl methacrylate (>97.0%, Tokyo Chemical Industry), Palladium(II) acetate (>99.9%, trace metals basis, Sigma-Aldrich), Sodium *tert*-butoxide (NaOtBu, >98%, Tokyo Chemical Industry), Tri-tert-butylphosphonium tetrafluoroborate ($t Bu_3P \cdot HBF_4$, 97%, Fluorochem Ltd), Copper(I) iodide (CuI, 98%, Sigma-Aldrich), Copper(I) bromide (CuBr, 98%, Sigma-Aldrich), Anhydrous Sodium sulfate (> 99%, Fisher Scientific), Potassium carbonate (K_2CO_3, 99%, Sigma-Aldrich), Toluene (99.8%, Fisher Scientific), Anhydrous Toluene (99.8%, Fisher Scientific), Ethanol (EtOH, >99.8%, Sigma-Aldrich), Methanol (MeOH, >99.9%, HPLC gradient grade, Sigma-Aldrich), N,N-Dimethylformamide (DMF, >99%, Sigma-Aldrich), Anhydrous DMF (99.8%, Fisher Scientific), Dichloromethane (DCM, 99.8%, Fisher Scientific), Anhydrous DCM (99.8%, Fisher Scientific), Tetrahydrofuran (THF, >99.5%, , Sigma-Aldrich), Anhydrous THF (99.5%, Fisher Scientific), Dichloromethane-d_2 (DCM-d_2, 99.5%, Eurisotop), Chloroform-*d* ($CDCl_3$, >99% deuterated, Sigma-Aldrich), Dimethyl sulfoxide-d6 (DMSO-d6, 99.9% deuterated, Eurisotop)

All chemicals above were listed in order of organic chemicals, salts, and solvents. All chemicals were used as received without further purification.

Measurement

Proton nuclear magnetic resonance (^{1}H NMR, 300 MHz) and carbon nuclear magnetic resonance (^{13}C NMR, 76 MHz) spectra were recorded on a spectrometer (Bruker, USA) at room temperature.

UV-visible absorption spectra of the polymers were measured using Jasco V670 spectrophotometer. The scanning rate was 200 nm/min. PL characterizations of the liquid samples were carried out using a Hamamatsu Quantaurus QY absolute PL quantum yield spectrometer (C11347-11) equipped with a 150 W xenon lamp and a 3.3 inch integrating sphere, which is coated with highly reflective Spectralon. The PL spectra were measured at an excitation wavelength of 320 nm. The liquid samples were diluted accordingly with toluene.

The thin film samples were spin-coated by preparing 20 mg/mL concentration solution in toluene with 2000 rpm on 1x1 cm quartz substrate.

Time-resolved photoluminescence (TRPL) spectra of the ELPR thin films and solutions were measured using a Hamamatsu Quantaurus-Tau Fluorescence Lifetime Spectrometer (C11367-31), which is equipped with a photon counting measurement system. The solution samples were excited using a 340 nm pulsed emission with a repetition rate of 5 kHz. The concentration of polymer solutions in toluene was 0.5 mM. The instrument response function (IRF) was measured using a LUDOX® AS-30 colloidal silica suspension (30 wt. % suspension in H_2O, Aldrich) diluted with water.

In order to carry out temperature dependent TRPL and PL intensity measurements for thin film, an Oxford Optistat DN cryostat was integrated with the Quantaurus-Tau (C11367) Fluorescence lifetime spectrometer. This spectrometer used time-correlated single photon counting (TCSPC) to measure TRPL lifetime. The thin film samples were spin-coated by preparing 20 mg/mL concentration solution in toluene with 2000 rpm on 1x1 cm quartz substrate. The thin film samples were excited using a picosecond pulsed laser (PLP-10) with an excitation wavelength of 406 nm, a repetition rate of 10 kHz, 10000 counts, and a FWHM of ~0.4 ns. All the TRPL traces were fitted with a bi-exponential function.

To isolate and quantify the prompt and delayed emission components, the photoluminescence quantum yields (PLQYs) and time-resolved photoluminescence (TRPL) spectra of the TADF polymers were recorded in 0.1 mM dilute toluene solutions, utilizing oxygen bubbling to determine the prompt PLQY (Φ_{PF}).[14] The efficient quenching of triplet states (T_1) by oxygen ensures that the emission occurs exclusively via the prompt singlet pathway.

The total PLQY (Φ_{tot}) is measured after argon degassing the TADF polymer solution for 30 minutes to entirely remove dissolved oxygen, allowing both singlet and triplet pathways to contribute for PL emission. This yielded a Φ_{tot} of TADF polymer in the toluene solution.

The delayed PLQY (Φ_{DF}), in **Supplementary Table 8**, is calculated directly by subtracting the Φ_{PF} from Φ_{tot}.

$$\Phi_{DF} = \Phi_{tot} - \Phi_{PF}$$

This photophysical assignment is explicitly corroborated by the TRPL decay spectra provided in **Supplementary Fig. 54**. In the oxygen-bubbled toluene solution, the triplet excited states are fully quenched, resulting in a clean, monoexponential prompt decay profile devoid of any delayed features.[3] In stark contrast, the argon-degassed solution exhibits a pronounced long-lived delayed emission component with a prompt component. Both prompt and delayed exciton lifetimes (τ_{PF} and τ_{DF}) were estimated by fitting the TRPL decay curves with an Arrhenius biexponential function. This clearly confirms that the triplet excited states are preserved under inert conditions and successfully undergo thermally activated reverse intersystem crossing (RISC) back to the singlet manifold ($T_1 \rightarrow S_1$) to generate delayed fluorescence. Note that the fast RISC rate (k_{RISC}) in the TADF polymers is directly associated with fast reverse intersystem crossing that directly associated with intersystem crossing rate (k_{ISC}).

By extracting the τ_{PF}, τ_{DF}, k_P and k_P from these TRPL curves (**Supplementary Fig. 54**) alongside the PLQY data, we evaluated the key kinetic rate constant of the self-hosted TADF polymers and ELPRs. We then derived the key kinetic characteristics, such as radiative decay rate constant (k_r^s), reverse intersystem crossing rate constant (k_{RISC}), intersystem crossing rate constant (k_{ISC}), and non-radiative radiative rate constant from triplet state (k_{nr}^T) using a well-established TADF kinetic model reported by Adachi group.[15,16] All photophysical characteristics, and derived rate constants are summarized in **Table 8**.

Thermogravimetric analysis (TGA) performed using Netzsch-Gerätebau GmbH - STA 449 C Jupiter Thermo-microbalance TGA analyzer. The weight loss of TADF polymer emitter was recorded between 70 and 600 °C with a constant heating rate of 10 °C min^{-1} under inert atmosphere as nitrogen flow with a flow rate of 10 ml min^{-1}. All samples were preheated before performing the TGA analysis to avoid any moisture content and solvent traces effect on weight loss.

Atomic force microscopy (AFM) images were carried out on NX20 (Park Systems) in non-contact mode.

SEM (Scios 2 DualBeam, Thermo Fisher Scientific) images were taken to characterize the morphology of the ELPR or ER patterns.

Fluorescent images were taken by using Zeiss optical microscope installed with 365 nm LED light source to excite the emission from polymers in reflective mode. High-magnification objectives (20X, 50X, 100X) are used to resolve features down to the diffraction limit.

Size-exclusion chromatography (SEC) measurements were carried out on a system from Shimadzu (Kyoto, Japan) including a CBM-20A system controller, an SIL-20A HT auto 14 sampler, a guard column (50 × 7.5 mm, beadsize: 10.0 µm) followed by three KF-805L columns (300 × 8 mm, bead size: 10 µm, pore size maximum: 5000 Å), an SPD-20A ultraviolet detector, and a RID-20A refractive-index detector. Measurements were conducted at 40 °C using a CTO20A oven.

Matrix-assisted laser desorption/ionization-time of flight (MALDI-TOF) mass spectrometry was performed using Bruker's UltraFlex II system, with DCTB as the matrix (10.0 eq.) and silver trifluoroacetate as the cationization agent (1.0 eq.). The samples were prepared by dissolving in THF (1.0 eq.).

Electrospray ionization mass spectrometry (ESI-MS) experiments were conducted using an Agilent 6230 time-of-flight liquid chromatography/MS system using positive mode.

The cyclic voltammograms (CVs) of polymers were recorded with a Vionic potentiostat, powered by Intello, from Metrohm Autolab. All measurements were conducted on nitrogen degassed 0.05 mM solutions of polymers in dichloromethane (DCM) containing 0.1 M of tetra

-n-butylammonium hexafluorophosphate (nBu_4NPF_6) as supporting electrolyte at room temperature. A three-electrode system was used, with glassy carbon as a working electrode, and platinum wires as a counter electrode as well as reference electrode, in a glass electrochemical cell. A ferrocenium/ferrocene (Fe/Fe^+) couple was used, as the external reference and a correction factor was used, as determined by the CV of ferrocene in DCM using same reference electrode. The CVs of polymers were recorded using linear scan with a scan rate of 0.1 V s^{-1}, after each scan, the solutions were purged with argon for 30 minutes.

The J-V-L characteristics and EL spectra of the OLED devices were measured using a an absolute η_{ext} measurement system (C9920-12; Hamamatsu Photonics) consisting a calibrated 3.3 inches reflective Spectralon coated integrating sphere (A10094), a highly sensitive CCD Spectrometer PMA-12 Photonic multichannel analyzer (C10027-01), a fiber probe, a computer-controlled Keithley 2450 source meter unit, and data analyzer EL efficiency measurement software package (U6039-06). The EL performance of our devices was also verified using Photo Research PR-655 SpectraScan spectroradiometer coupled with a Keithley 2400 source measurement unit. The operational lifetime (LT_{50}) of the encapsulated devices were evaluated at room temperature under ambient relative humidity conditions. Long-term stability characterization was executed using a commercial, 16 parallel stressing channels in 4 different chambers, Litos LED Lifetime System (Fluxim AG) equipped with calibrated silicon

photodiodes to track luminance decay. The devices were operated in a constant direct-current (DC) driving mode at varied current densities of 1 mA cm^{-2}, 2.4 mA cm^{-2}, 5 mA cm^{-2}, and 10 mA cm^{-2}.

Supplementary Note 2. | Synthetic route of green-emitting TADF initiator for ATRP

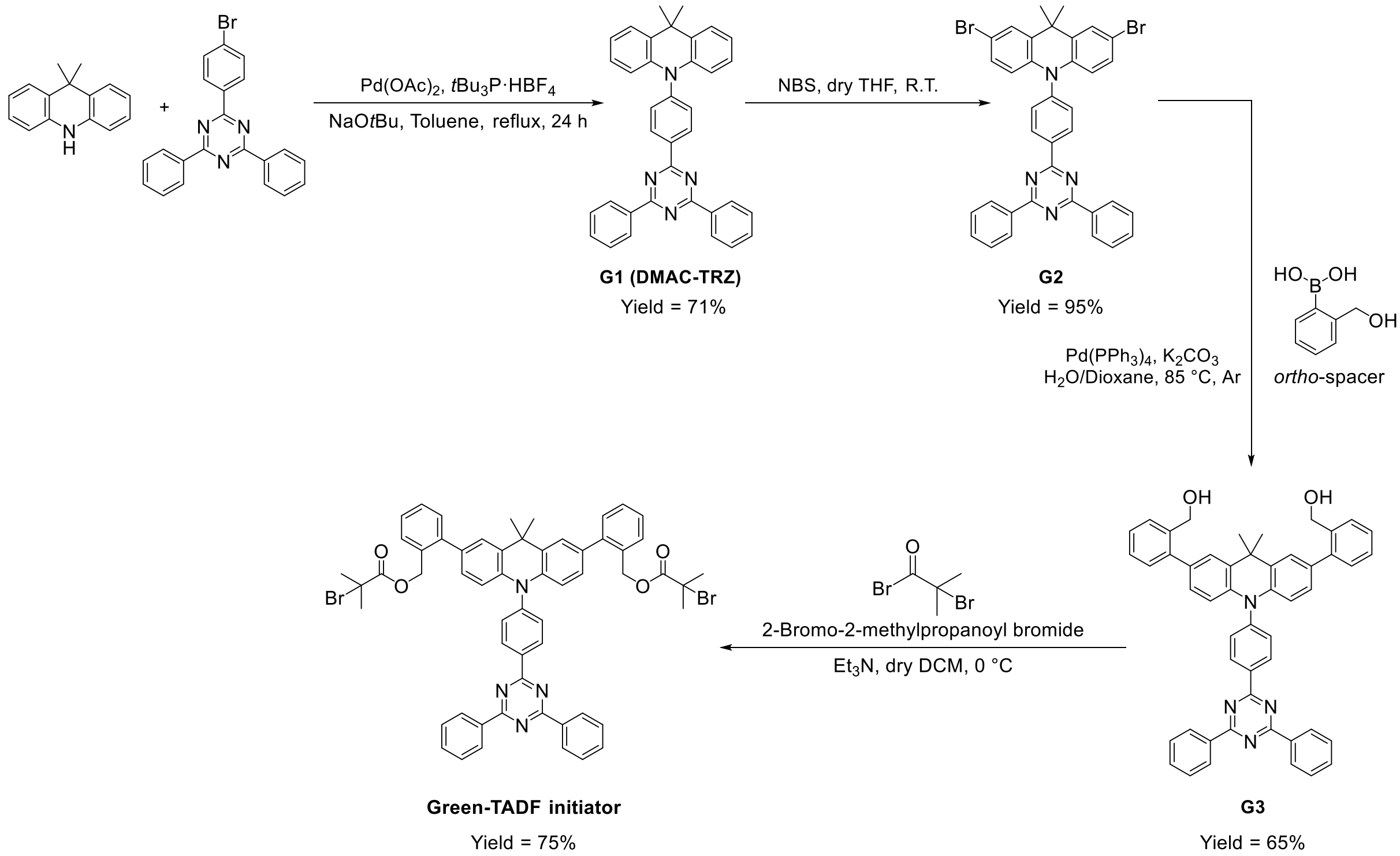

Supplementary Fig. 1: Synthetic route of green-emitting TADF initiator.

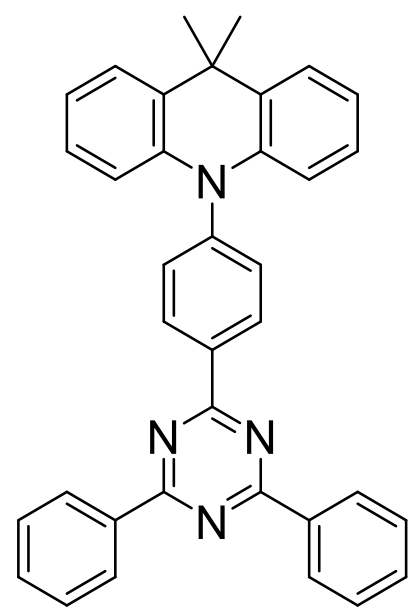

Synthesis of **G1**

As shown in **Supplementary Fig. 1**, **G1** was synthesized based on the reported method.[1] A 100 mL Schlenk flask was charged with a stirring bar, 2-(4-bromophenyl)-4,6-diphenyl-1,3,5-triazine (1.00 g, 2.58 mmol, 1.0 eq.), DAMC (0.593 g, 2.83 mmol, 1.1 eq.), $Pd(OAc)_2$ (0.011 g, 0.05 mmol, 0.02 eq.), NaO*t*Bu (0.297 g, 3.09 mmol, 1.2 eq.) and $t$$Bu_3P{\cdot}HBF_4$ (0.044 g, 0.15 mmol, 0.06 eq.). After drying the solids for 30 minutes under vacuum, the flask was flushed three times with argon and charged with anhydrous toluene (30 mL) degassed with argon previously. The reaction mixture was heated to 115 °C and refluxed overnight. After cooling down to room temperature, the residue was diluted in DCM (50 mL) and washed with water (3 x 10 mL) and brine (30 mL), dried over anhydrous Na_2SO_4 and evaporated to dryness. The product was purified by column chromatography on silica gel eluting with a 4:1 mixture of hexane and DCM to produce pale green solids (0.95 g, 1.84 mmol, 71%). ^{1}H NMR (300 MHz, $CDCl_3$) δ 9.13 – 8.99 (m, 2H), 8.91 – 8.75 (m, 4H), 7.72 – 7.54 (m, 8H), 7.54 – 7.44 (m, 2H), 7.07 – 6.88 (m, 4H), 6.45 – 6.34 (m, 2H), 1.74 (s, 6H). ^{13}C NMR (76 MHz, $CDCl_3$) δ 171.88,

171.11, 145.37, 140.64, 136.11, 132.72, 131.62, 131.54, 130.27, 129.05, 128.75, 126.48, 125.38, 120.88, 114.23, 77.25, 36.08, 31.34. See **Supplementary Fig. 2.**

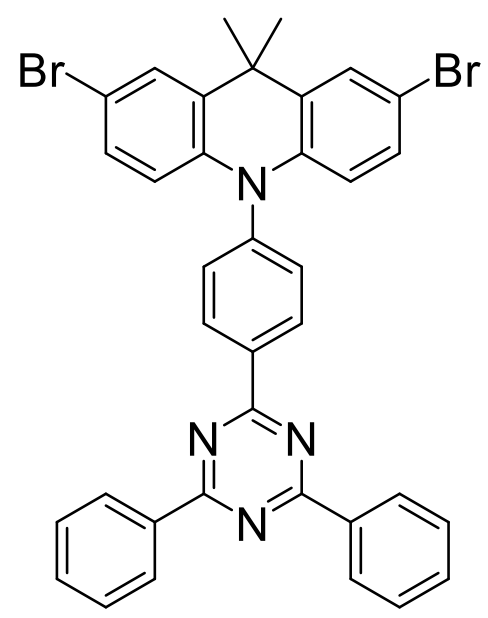

Synthesis of **G2**

A 250 mL round bottomed flask was charged with **G1** (1.20 g, 2.32 mmol, 1 eq.) and anhydrous THF (40 mL). NBS (0.868 g, 4.88 mmol, 2.1 eq.) in anhydrous THF (15 mL) was added dropwise to the solution under 0 °C ice bath. The mixture was stirred overnight at room temperature. The residue was diluted in DCM (20 mL) and washed with brine (2 x 20 mL), dried over anhydrous Na_2SO_4 and evaporated to dryness. The crude solids were dissolved in THF and precipitated in hexane, and collected through vacuum filtration. The product was further recrystallized in THF/MeOH to afford pale solids (1.5 g, 2.22 mmol, 95%). ^{1}H NMR (300 MHz, CD_2Cl_2) δ 9.04 (d, J = 8.4 Hz, 2H), 8.85 – 8.78 (m, 4H), 7.70 – 7.60 (m, 6H), 7.58 – 7.51 (m, 4H), 7.10 (dd, J = 8.8, 2.3 Hz, 2H), 6.28 (d, J = 8.8 Hz, 2H), 1.67 (d, J = 1.8 Hz, 6H). ^{13}C NMR (76 MHz, CD_2Cl_2) δ 171.90, 170.94, 144.36, 139.57, 136.67, 136.04, 132.78, 131.98, 131.75, 131.17, 129.32, 128.93, 128.75, 128.17, 115.99, 113.43, 36.33, 30.73. See **Supplementary Fig. 3.**

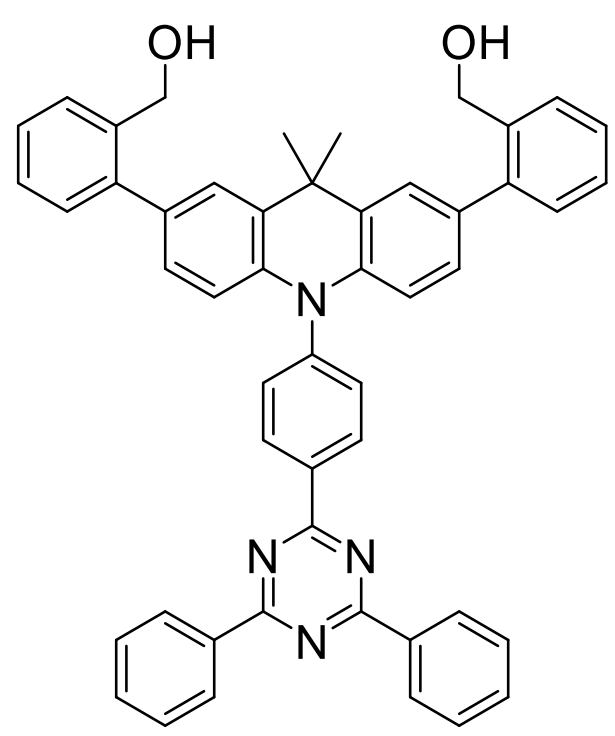

Synthesis of **G3**

A 100 mL schlenk flask equipped with a stirring bar was charged with **G2** (1.20 g, 1.78 mmol, 1 eq.), (2-(Hydroxymethyl)phenyl)boronic acid (*ortho*-spacer, 0.765 g, 5 mmol, 2.8 equiv.), K_2CO_3 (1.72 g, 12.45 mmol, 7 equiv.) and $Pd(PPh_3)_4$ (92 mg, 0.11 mmol, 0.06 eq.). After drying under vacuum for 30 minutes, the flask with solids was flushed three times with argon. The solids were dissolved in mixture of dioxane (40 mL) and water (10 mL) degassed by argon bubbling. The reaction mixture was heated to 85 °C and reacted for 36 hours. After cooling down to room temperature, the residue was dissolved in DCM (50 mL) and washed with water (3 x 10 mL) and brine (2 x 20 mL), dried over anhydrous Na_2SO_4 and evaporated to dryness. The product was purified by column chromatography on silica gel eluting with a mixture of

DCM and MeOH (from v:v = 1:0 to 100:1) to produce a yellowish solid (0.849 g, 1.16 mmol, 65%). ^{1}H NMR (300 MHz, $CDCl_3$) δ 9.16 – 9.02 (m, 2H), 8.89 – 8.77 (m, 4H), 7.69 – 7.53 (m, 12H), 7.35 (tdd, *J* = 9.5, 4.3, 3.2 Hz, 6H), 7.04 (dd, *J* = 8.4, 2.0 Hz, 2H), 6.46 (d, *J* = 8.4 Hz, 2H), 4.68 (s, 4H), 1.78 (s, 6H), 1.61 (s, 2H). ^{13}C NMR (76 MHz, $CDCl_3$) δ 171.92, 145.00, 141.42, 139.76, 138.16, 136.45, 136.08, 133.13, 132.75, 131.75, 131.56, 130.12, 129.88, 129.06, 128.75, 128.64, 127.79, 127.35, 127.28, 126.64, 114.06, 77.23, 63.46, 36.27, 31.69. See **Supplementary Fig. 4.**

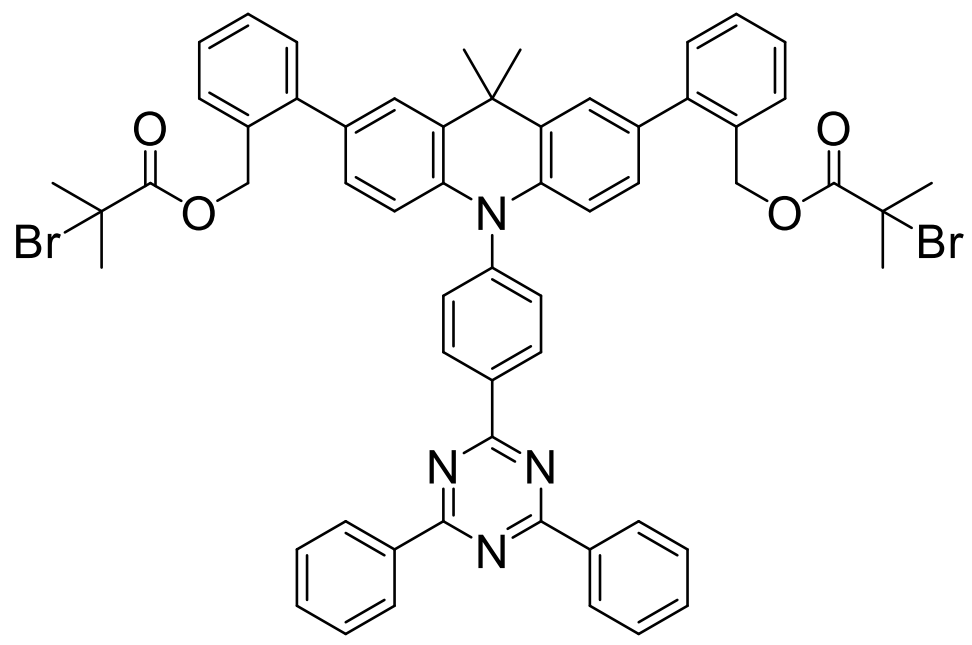

Synthesis of **green-TADF initiator**

A 100 ml flask with a stirring bar was charged with **G3** (0.60 g, 0.82 mmol, 1 eq.), triethylamine (Et_3N, 460 µL, 3.29 mmol, 4 eq.) and anhydrous DCM (15 mL) under N_2. 2-bromoisobutyryl bromide (230 µL, 1.81 mmol, 2.2 eq.) was added dropwise to the solution under 0 °C ice bath. The mixture was stirred overnight at room temperature. The residue was dissolved in DCM (30 mL), washed with water (2 x 15 mL) and brine (30 mL), dried over anhydrous Na_2SO_4 and evaporated to dryness. The product was purified by column chromatography on silica gel eluting with a 1:1 mixture of hexane and DCM to produce yellow solids (0.632 g, 0.616 mmol, 75%). ^{1}H NMR (300 MHz, $CDCl_3$) δ 9.08 (d, *J* = 8.2 Hz, 2H), 8.90 – 8.78 (m, 4H), 7.71 – 7.57 (m, 8H), 7.57 – 7.46 (m, 4H), 7.45 – 7.32 (m, 6H), 7.02 (dd, *J* = 8.5, 2.0 Hz, 2H), 6.43 (d, *J* = 8.5 Hz, 2H), 5.22 (s, 4H), 1.92 (s, 12H), 1.79 (s, 6H). ^{13}C NMR (76 MHz, $CDCl_3$) δ 171.93, 171.48, 142.26, 139.80, 136.08, 132.83, 132.74, 131.78, 131.55, 130.24, 129.95, 129.45, 129.06, 128.76, 128.48, 127.39, 127.25, 126.52, 114.13, 66.09, 55.75, 36.28, 31.89, 30.85. ESI-MS: m/z of adduct $(M+Na)^+$, calcd. for $C_{58}H_{50}Br_2N_4O_4$, 1047.2091; found, 1047.2087. See **Supplementary Fig. 5.**

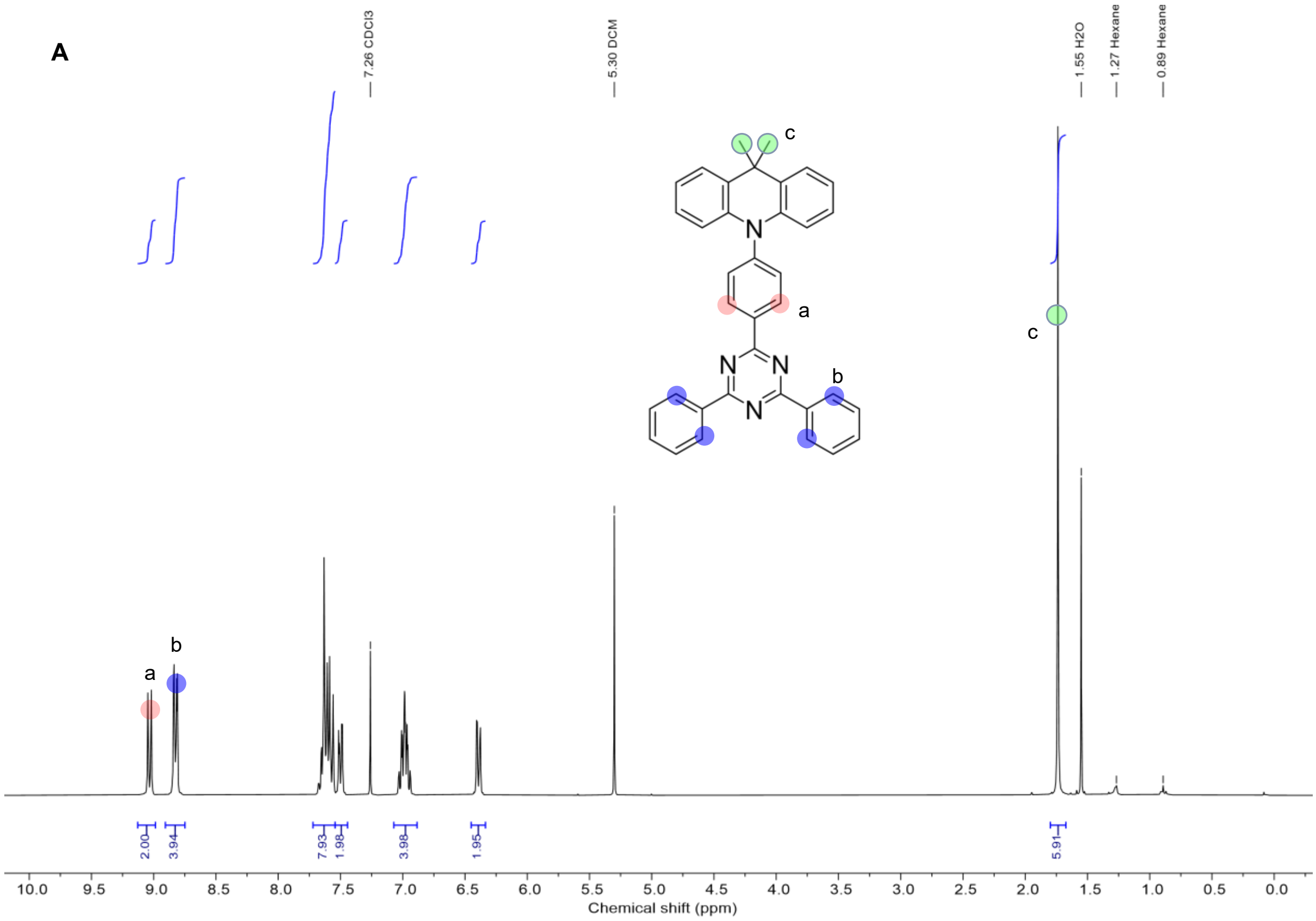

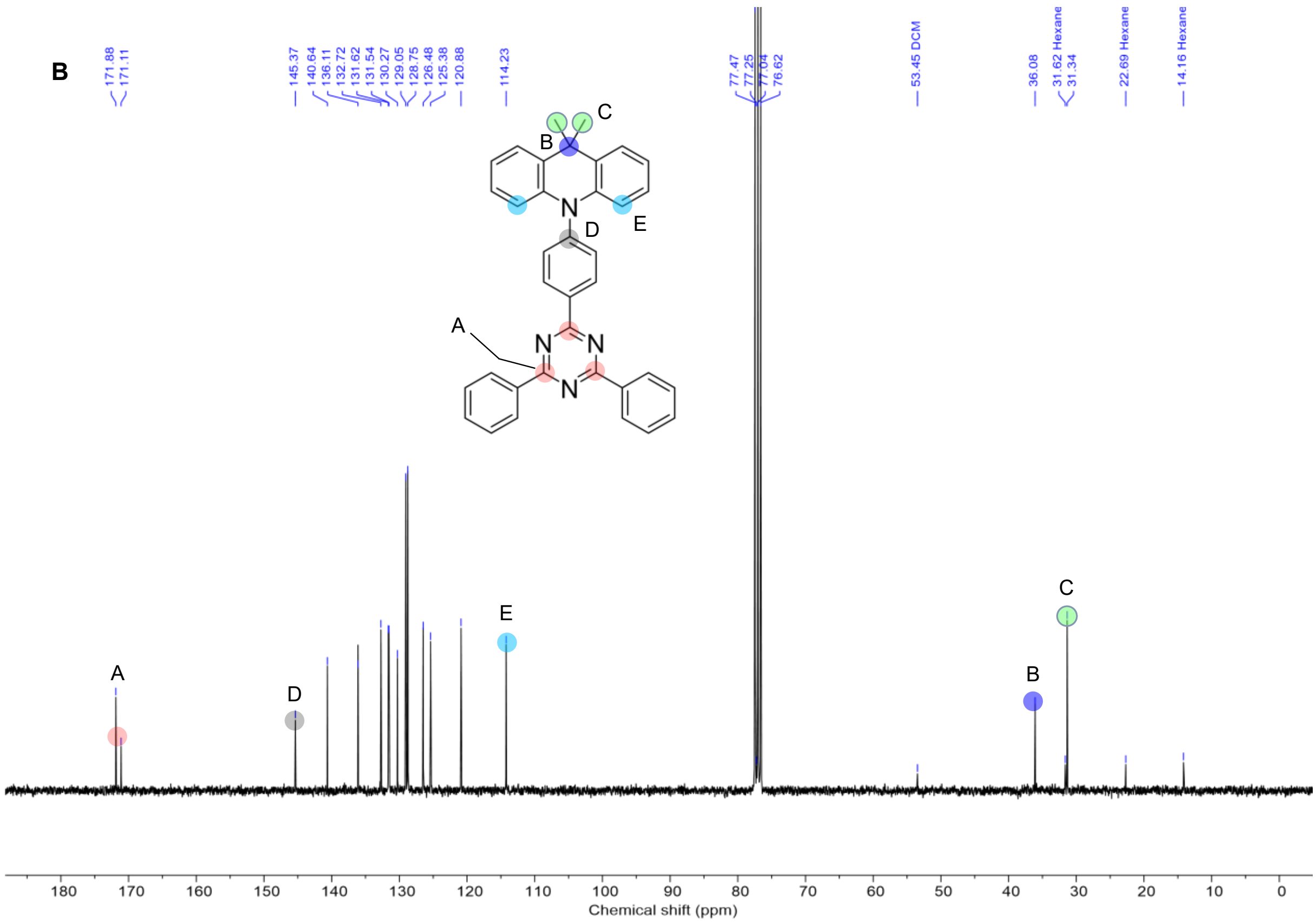

Supplementary Fig. 2: Structural characterization of G1 (DMAC-TRZ). (**A**) ^{1}H NMR and (**B**) ^{13}C NMR spectrum in $CDCl_3$-*d*.

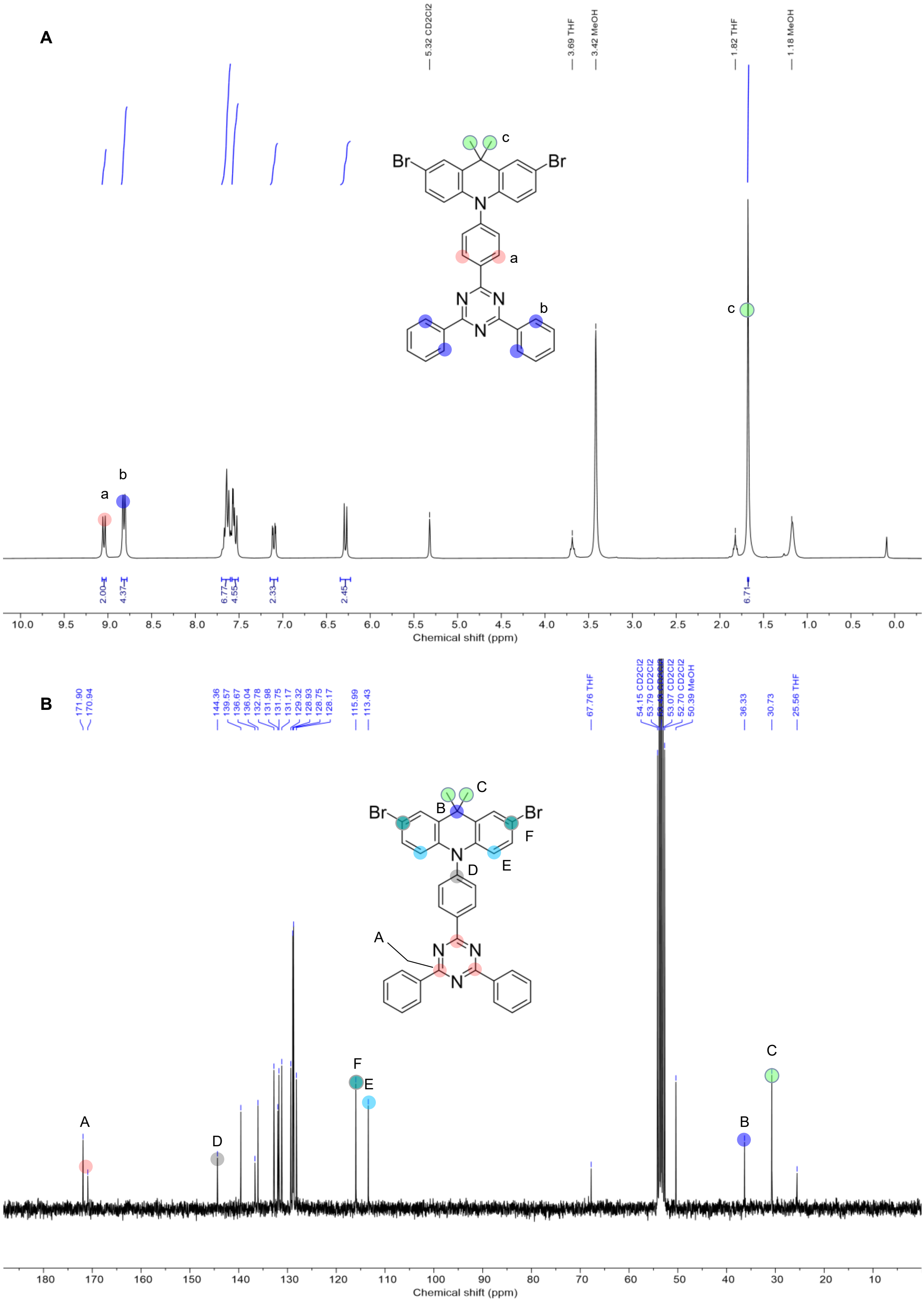

Supplementary Fig. 3: Structural characterization of G2. (**A**) ^{1}H NMR and (**B**) ^{13}C NMR spectrum in CD_2Cl_2-*d_2*.

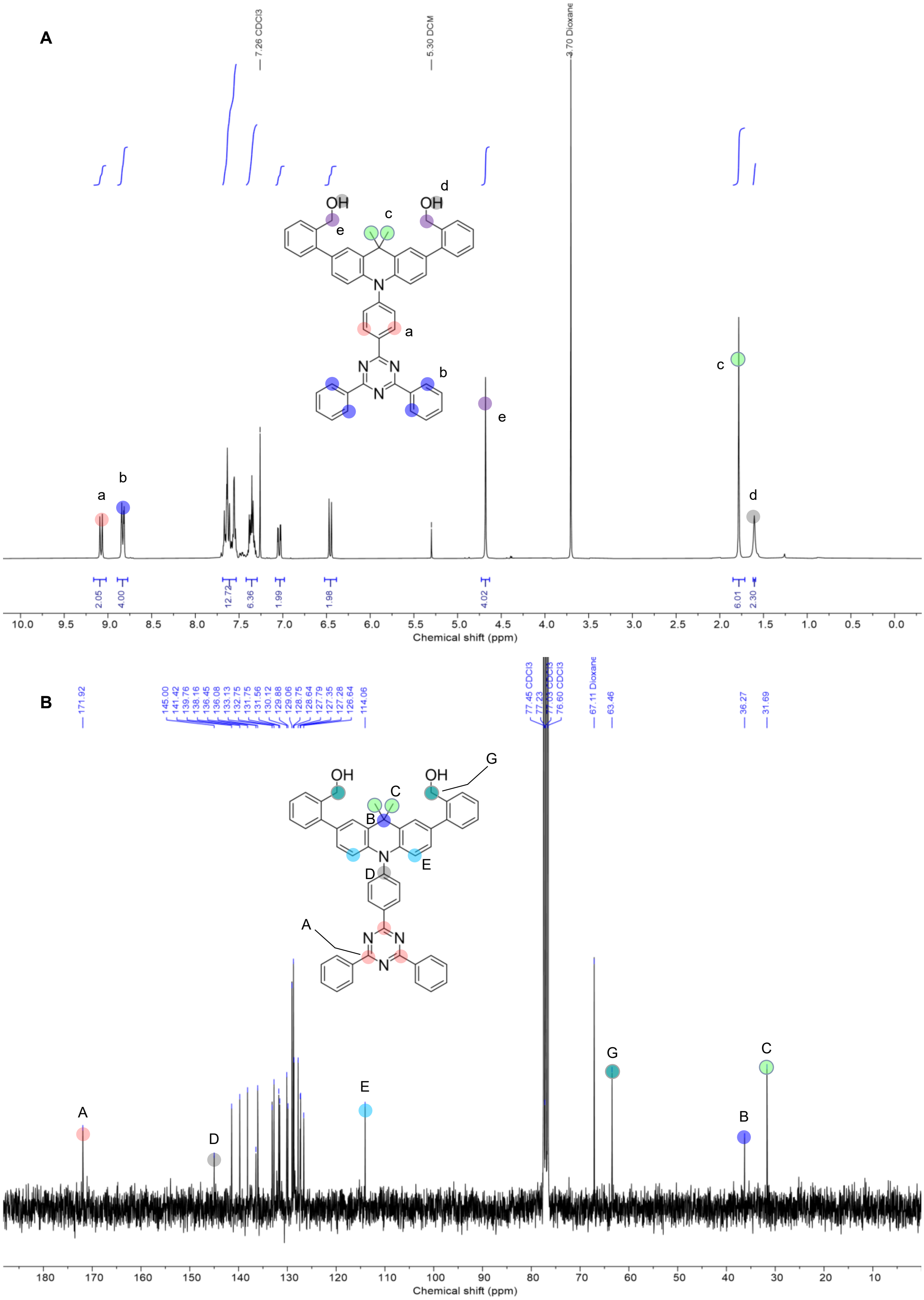

Supplementary Fig. 4: Structural characterization of G3. (**A**) ^{1}H NMR and (**B**) ^{13}C NMR spectrum in $CDCl_3$-*d*.

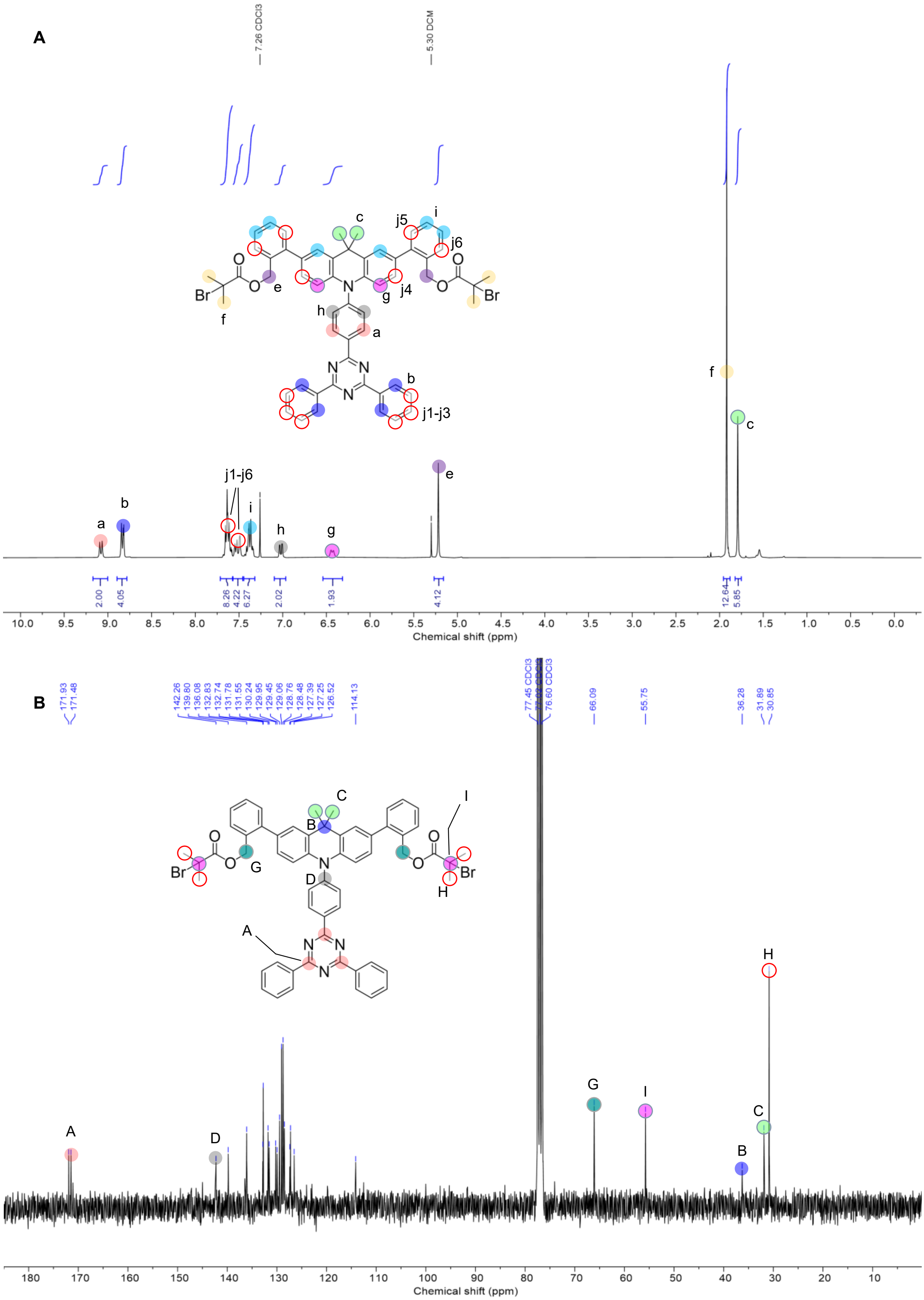

Supplementary Fig. 5: Structural characterization of green-TADF initiator. (**A**) ^{1}H NMR and (**B**) ^{13}C NMR spectrum in $CDCl_3$-*d*.

Supplementary Note 3 | Synthetic route of red-emitting TADF initiator for ATRP

Please see **Supplementary Fig. 6.**

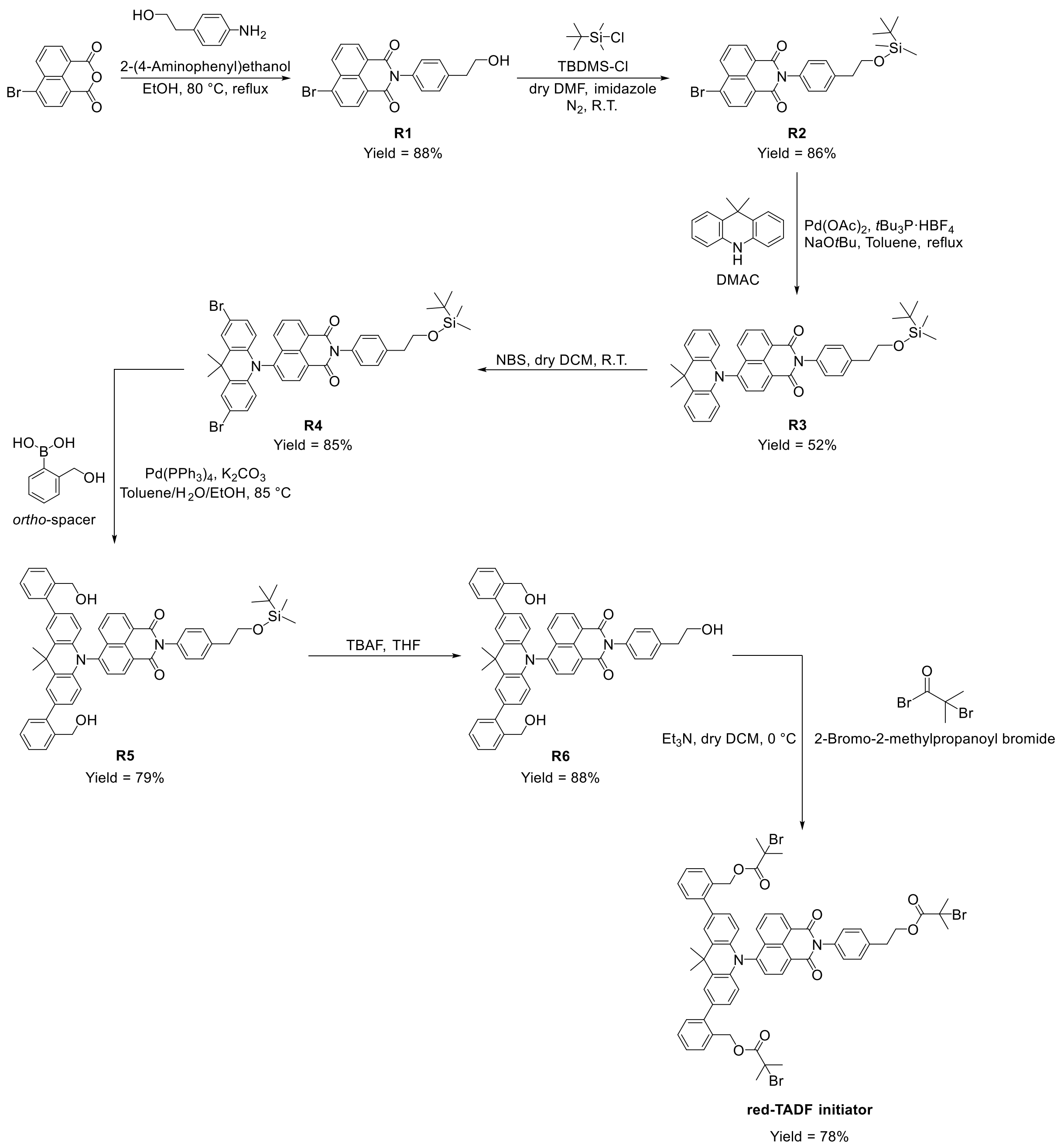

Supplementary Fig. 6: Synthetic route of red-emitting TADF initiator.

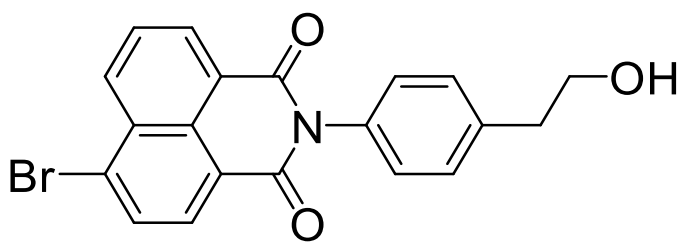

Synthesis of **R1**

A 250 mL flask with a stirring bar was charged with 4-bromo-1,8-naphthalic anhydride (5.54 g, 20 mmol, 1 eq.) and 2-(4-aminophenyl)ethanol (8.23 g, 60 mmol, 3 eq.). The solids were dissolved in 120 mL EtOH, and the solution was heated to 80 °C and refluxed overnight. After cooling down to room temperature, the crude mixture was poured into water. The precipitates were collected by filtration, and washed with EtOH. Yellow brownish powders were obtained as product after drying under vacuum overnight (6.97 g, 17.59 mmol, 88%). ^{1}H NMR (300 MHz, DMSO) δ 8.59 – 8.56 (m, 1H), 8.56 – 8.53 (m, 1H), 8.32 (d, *J* = 7.9 Hz, 1H), 8.22 (d, *J* = 7.9 Hz, 1H), 8.06 – 7.96 (m, 1H), 7.37 (d, *J* = 8.3 Hz, 2H), 7.27 (d, *J* = 8.3 Hz, 2H), 4.74 (t, *J* = 5.1 Hz, 1H), 3.70 (q, *J* = 6.7 Hz, 2H), 2.82 (t, *J* = 6.9 Hz, 2H). ^{13}C NMR (76 MHz, DMSO) δ 163.71, 163.65, 140.25, 134.00, 133.17, 132.08, 131.85, 131.44, 130.38, 129.83, 129.63, 129.31, 129.20, 129.10, 123.83, 123.05, 62.45, 39.13. See **Supplementary Fig. 7.**

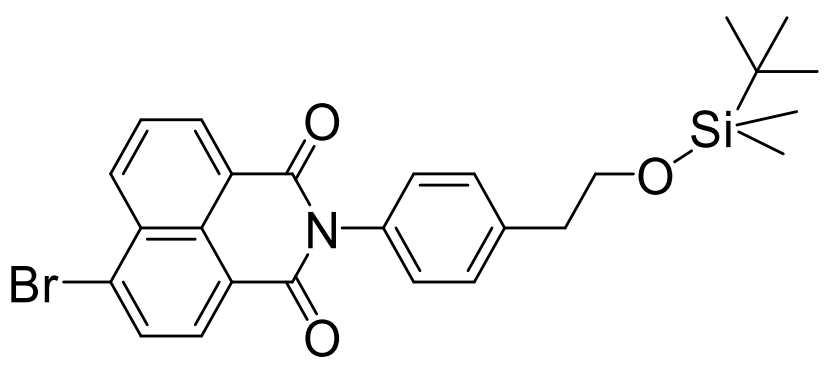

Synthesis of **R2**

A 250 mL flask with a stirring bar was charged with **R1** (5.151 g, 13 mmol, 1 eq.), imidazole (1.770 g, 26 mmol, 2 eq.), and anhydrous DMF (50 mL) under N_2. The mixture was stirred for 20 minutes at room temperature. Then TBDMS-Cl (2.939 g, 19.5 mmol, 1.5 eq.) was added dropwise to the solution and the mixture was kept stirring overnight under N_2. The crude slurry was filtered under reduced pressure, and washed with water. Grey solids were obtained as product after drying under vacuum overnight (5.711 g, 11.18 mmol, 86%). ^{1}H NMR (300 MHz, $CDCl_3$) δ 8.70 (dd, *J* = 7.3, 1.1 Hz, 1H), 8.63 (dd, *J* = 8.5, 1.1 Hz, 1H), 8.46 (d, *J* = 7.9 Hz, 1H), 8.08 (d, *J* = 7.9 Hz, 1H), 7.88 (dd, *J* = 8.5, 7.3 Hz, 1H), 7.39 (d, *J* = 8.0 Hz, 2H), 7.25 – 7.18 (m, 2H), 3.88 (t, *J* = 7.1 Hz, 2H), 2.92 (t, *J* = 7.1 Hz, 2H), 0.91 (s, 9H), 0.04 (s, 6H). ^{13}C NMR (76 MHz, $CDCl_3$) δ 163.86, 163.83, 139.94, 133.61, 133.13, 132.45, 131.60, 131.22, 130.83, 130.63, 130.19, 129.39, 128.26, 128.19, 123.32, 122.44, 64.36, 39.38, 25.99, 18.39, -5.34. See **Supplementary Fig. 8.**

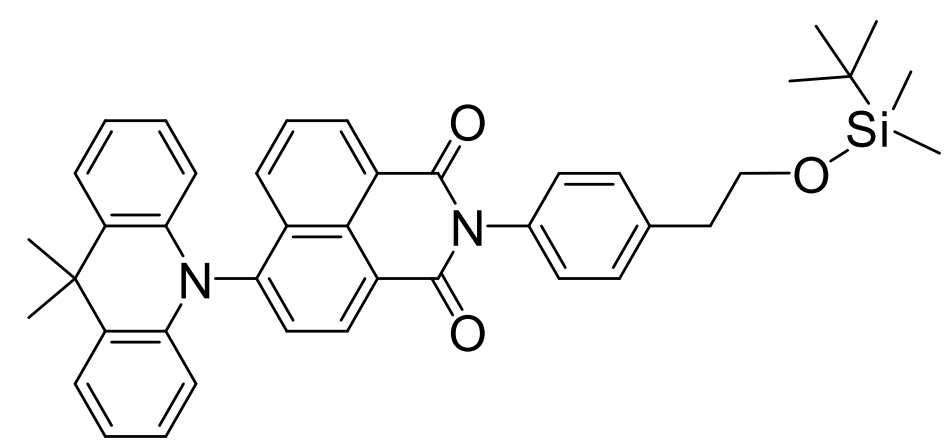

Synthesis of **R3**

R3 was synthesized based on the reported method.[2] A 100 mL schlenk flask with a stirring bar was charged with **R2** (0.766 g, 1.5 mmol, 1 eq.), DMAC (0.345 g, 1.65 mmol, 1.1 eq.), $Pd(OAc)_2$

(0.0067 g, 0.03 mmol, 0.02 eq.), NaO*t*Bu (0.173 g, 1.8 mmol, 1.2 eq.), and *t*$Bu_3P·HBF_4$ (0.026 g, 0.09 mmol, 0.06 eq.). After drying under vacuum for 30 minutes, the flask with solids was flushed three times with argon and charged with dry toluene (30 mL). The reaction mixture was heated to 115 °C and reacted overnight. After cooling down to room temperature, the residue was dissolved in DCM (50 mL) and washed with water (3 x 10 mL) and brine (30 mL), dried over anhydrous Na_2SO_4 and evaporated to dryness. The product was purified by column chromatography on silica gel eluting with a 1:3 mixture of hexane and DCM to produce orange solids (0.5 g, 0.783 mmol, 52.2%). ^{1}H NMR (300 MHz, CD_2Cl_2) δ 8.83 (d, *J* = 7.7 Hz, 1H), 8.64 (dd, *J* = 7.2, 1.2 Hz, 1H), 8.07 (dd, *J* = 8.4, 1.2 Hz, 1H), 7.85 (d, *J* = 7.8 Hz, 1H), 7.69 (dd, *J* = 8.5, 7.3 Hz, 1H), 7.56 (dd, *J* = 7.8, 1.6 Hz, 2H), 7.48 – 7.41 (m, 2H), 7.31 – 7.24 (m, 2H), 6.96 (td, *J* = 7.4, 1.3 Hz, 2H), 6.91 – 6.83 (m, 2H), 5.99 (dd, *J* = 8.1, 1.3 Hz, 2H), 3.93 (t, *J* = 6.9 Hz, 2H), 2.95 (t, *J* = 6.9 Hz, 2H), 1.81 (d, *J* = 22.6 Hz, 6H), 0.93 (d, *J* = 0.8 Hz, 9H), 0.08 (d, *J* = 0.8 Hz, 6H). ^{13}C NMR (76 MHz, CD_2Cl_2) δ 164.03, 163.74, 144.32, 140.17, 140.13, 133.70, 132.67, 131.94, 130.90, 130.83, 130.70, 130.19, 130.03, 130.00, 128.46, 127.99, 126.64, 126.02, 124.02, 123.25, 121.20, 113.96, 64.23, 53.99, 39.27, 36.00, 32.53, 25.73, 18.23, -5.65. See **Supplementary Fig. 9.**

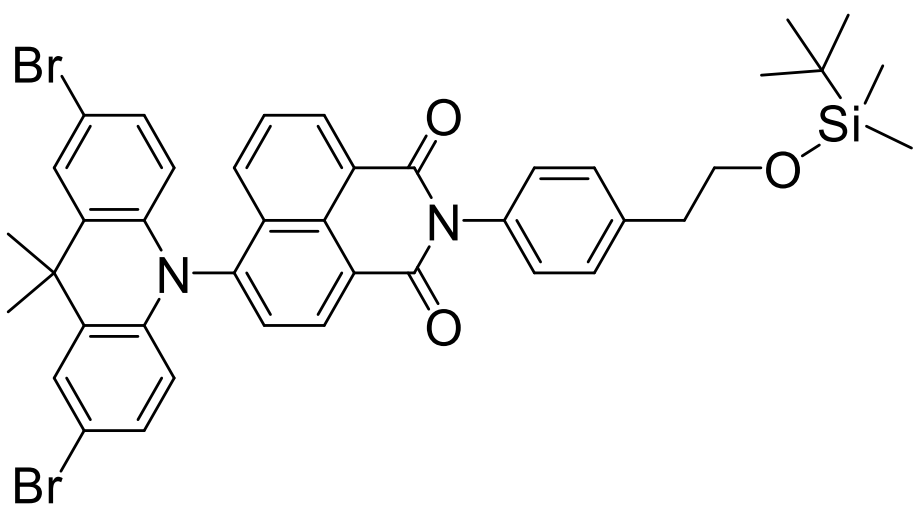

Synthesis of **R4**

A solution of N-bromosuccinimide (NBS, 0.352 g, 1.98 mmol, 2.2 eq.) in THF (5 mL) was added dropwisely to the solution of **R3** (0.575 g, 0.90 mmol, 1 eq.) in DCM (20 mL) in a round flask under 0 °C ice bath. The mixture was stirred at room temperature overnight. The residue was dissolved in DCM (50 mL) and washed with water (3 x 10 mL) and brine (2 x 15 mL), dried over anhydrous Na_2SO_4 and evaporated to dryness. The product was purified by column chromatography on silica gel eluting with DCM (0.610 g, 0.76 mmol, 85%). ^{1}H NMR (300 MHz, CD_2Cl_2) δ 8.82 (d, *J* = 7.7 Hz, 1H), 8.66 (dd, *J* = 7.3, 1.2 Hz, 1H), 7.98 (dd, *J* = 8.5, 1.2 Hz, 1H), 7.83 (d, *J* = 7.7 Hz, 1H), 7.71 (dd, *J* = 8.5, 7.2 Hz, 1H), 7.64 (d, *J* = 2.3 Hz, 2H), 7.48 – 7.42 (m, 2H), 7.30 – 7.24 (m, 2H), 6.99 (dd, *J* = 8.8, 2.3 Hz, 2H), 5.91 (d, *J* = 8.8 Hz, 2H), 3.94 (t, *J* = 6.9 Hz, 2H), 2.95 (t, *J* = 6.9 Hz, 2H), 1.79 (d, *J* = 28.4 Hz, 6H), 0.93 (s, 9H), 0.08 (s, 6H). ^{13}C NMR (76 MHz, CD_2Cl_2) δ 163.89, 163.58, 143.04, 140.26, 139.02, 133.58, 132.60, 132.13, 131.74, 130.70, 130.58, 130.23, 130.06, 129.71, 129.64, 128.85, 128.42, 128.32, 124.13, 123.73, 115.85, 113.91, 64.21, 39.27, 36.39, 32.37, 31.51, 25.74, 18.24, 13.89, -5.63. See **Supplementary Fig. 10.**

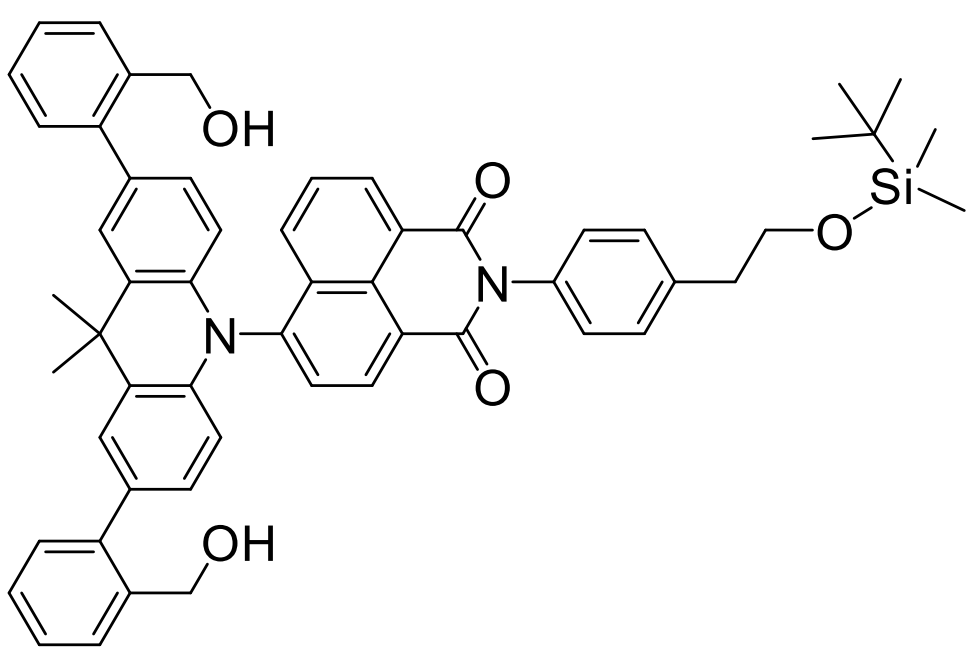

Synthesis of **R5**

A 100 mL schlenk flask with a stirring bar, was charged with **R4** (0.518 g, 0.65 mmol, 1 eq.), (2-(Hydroxymethyl)phenyl)boronic acid (*ortho*-spacer, 0.237 g, 1.56 mmol, 2.4 eq.), $Pd(PPh_3)_4$ (0.075 g, 0.065 mmol, 0.1 eq.), and K_2CO_3 (1.80 g, 13 mmol, 20 eq.). After drying under vacuum for 30 minutes, the flask with solids was flushed three times with argon. Degassed mixture of toluene (14 mL), water (7 mL), and EtOH (3.5 mL) was added to the flask. The mixture was heated to 85 °C and reacted for 36 hours. After cooling down to room temperature, the residue was diluted in DCM (50 mL) and washed with water (3 x 10 mL) and brine (30 mL), dried over anhydrous Na_2SO_4 and evaporated to dryness. The product was purified by column chromatography on silica gel eluting with a mixture of DCM and MeOH (from v:v = 1:0 to 100:3) to produce orange-red solids (0.44 g, 0.517 mmol, 79%). ^{1}H NMR (300 MHz, CD_2Cl_2) δ 8.87 (d, J = 7.7 Hz, 1H), 8.68 (dd, J = 7.3, 1.2 Hz, 1H), 8.22 (dd, J = 8.4, 1.2 Hz, 1H), 7.93 (d, J = 7.8 Hz, 1H), 7.80 – 7.73 (m, 1H), 7.65 (d, J = 2.1 Hz, 2H), 7.53 (dd, J = 5.9, 3.1 Hz, 2H), 7.45 (d, J = 7.9 Hz, 2H), 7.37 – 7.26 (m, 8H), 6.94 (dd, J = 8.5, 2.1 Hz, 2H), 6.09 (d, J = 8.5 Hz, 2H), 4.62 (d, J = 5.6 Hz, 4H), 3.94 (t, J = 6.9 Hz, 2H), 2.95 (t, J = 6.9 Hz, 2H), 1.88 (d, J = 14.3 Hz, 6H), 1.78 (t, J = 5.7 Hz, 2H), 0.94 (s, 9H), 0.08 (s, 6H). ^{13}C NMR (76 MHz, CD_2Cl_2) δ 164.02, 144.10, 141.18, 140.20, 139.24, 138.35, 133.55, 132.71, 132.06, 130.86, 130.04, 129.90, 129.75, 128.66, 128.46, 127.64, 127.49, 127.38, 127.29, 123.41, 113.90, 64.22, 63.12, 39.27, 36.25, 32.80, 25.73, 18.23, -5.65. See **Supplementary Fig. 11.**

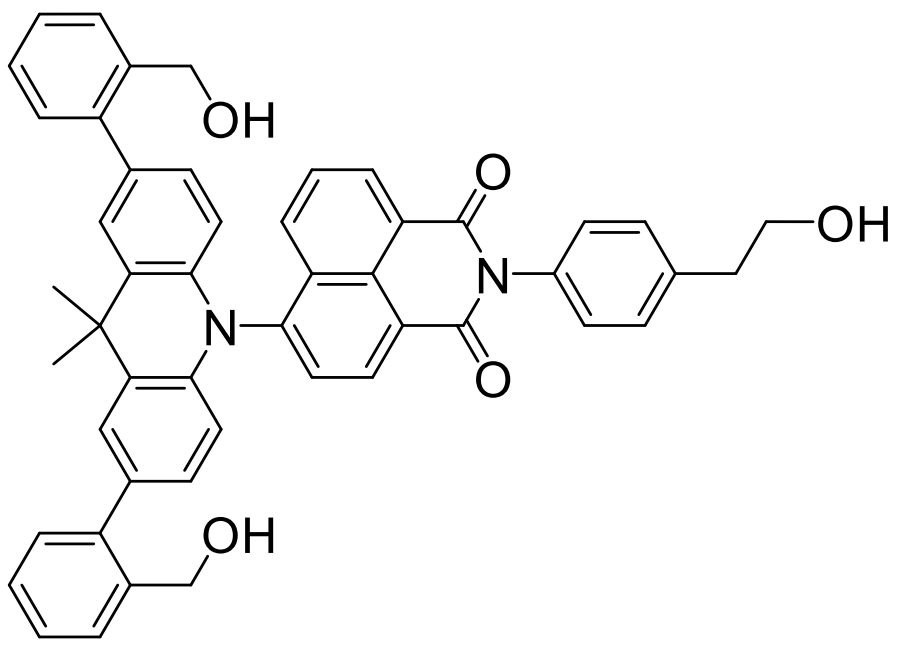

Synthesis of **R6**

A 100 ml flask with a stirring bar was charged with **R5** (0.409 g, 0.48 mmol, 1 eq.) and THF (20 mL). A clear red solution was obtained, and then TBAF solution (1M in THF, 1.01 mL, 1.01 mmol, 2.1 eq.) was added dropwise into the flask. The reaction was conducted at room temperature for six hours. The residue was dissolved in DCM (30 mL), washed with water (3 x 10 mL) and brine (30 mL), dried over anhydrous Na_2SO_4 and evaporated to dryness. The product was purified by column chromatography on silica gel eluting with a mixture of DCM and MeOH (from v:v = 1:0 to 100:3) to produce dark-red solids (0.313 g, 0.424 mmol, 88%). ^{1}H NMR (300 MHz, CD_2Cl_2) δ 8.84 (d, J = 7.7 Hz, 1H), 8.65 (d, J = 7.3 Hz, 1H), 8.20 (d, J = 8.5 Hz, 1H), 7.91 (d, J = 7.8 Hz, 1H), 7.74 (t, J = 7.9 Hz, 1H), 7.63 (d, J = 2.1 Hz, 2H), 7.54 – 7.48 (m, 2H), 7.45 (d, J = 7.9 Hz, 2H), 7.36 – 7.24 (m, 8H), 6.90 (dd, J = 8.5, 2.1 Hz, 2H), 6.06 (d, J = 8.5 Hz, 2H), 4.60 (d, J = 5.4 Hz, 4H), 3.91 (q, J = 6.2 Hz, 2H), 2.95 (t, J = 6.5 Hz, 2H), 1.90 (d, J = 6.3 Hz, 6H), 1.84 (s, 2H), 1.62 (s, 1H). ^{13}C NMR (76 MHz, CD_2Cl_2) δ 164.08, 163.77, 144.18, 141.15, 139.92, 139.23, 138.35, 133.82, 133.57, 132.79, 132.13, 130.89, 130.83, 130.72, 130.24, 130.03, 129.89, 129.75, 128.69, 128.66, 128.21, 127.62, 127.49, 127.40, 127.29, 124.00, 123.32, 113.89, 63.33, 63.08, 38.95, 36.25, 32.78, 32.63. See **Supplementary Fig. 12.**

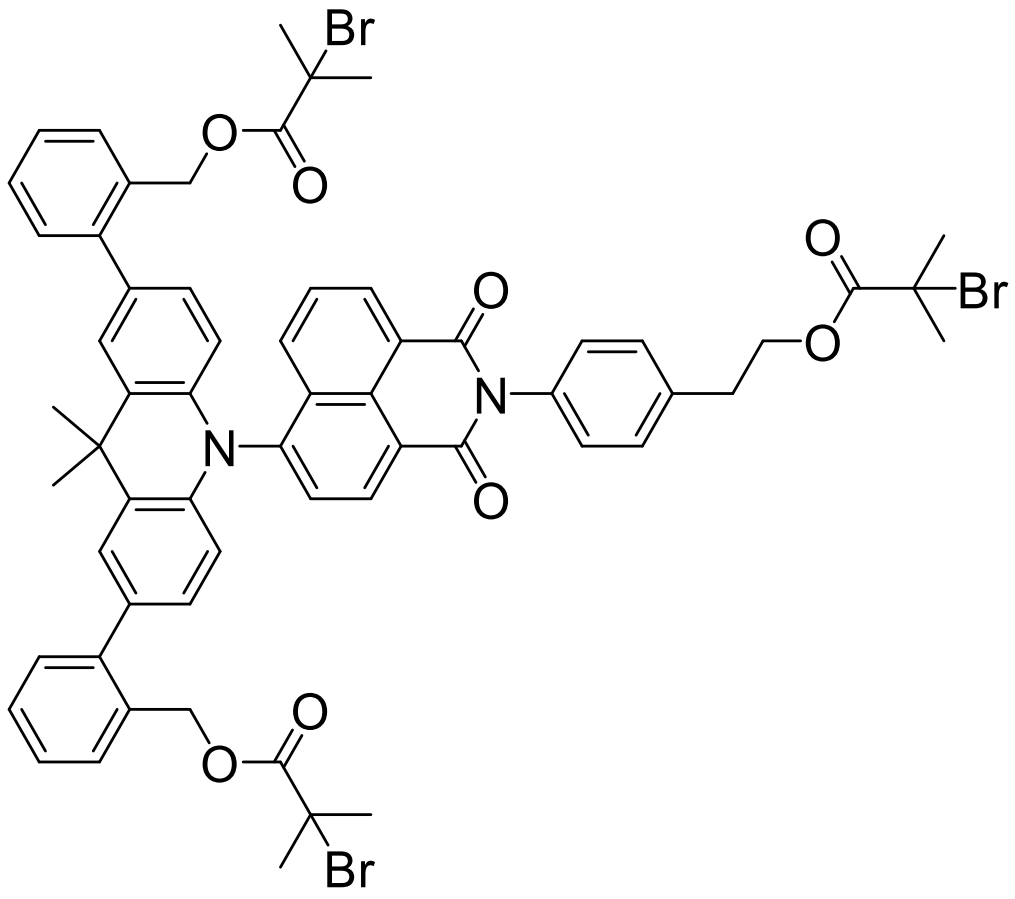

Synthesis of **red-TADF initiator**

A 100 ml flask with a stirring bar was charged with **R6** (0.287 g, 0.39 mmol, 1 eq.), triethylamine (Et_3N, 326 µL, 2.34 mmol, 6 eq.) and anhydrous DCM (15 mL) under N_2. 2-bromoisobutyryl bromide (289 µL, 2.34 mmol, 6 eq.) was added dropwise to the solution under 0 °C ice bath. The mixture was stirred overnight at room temperature. The residue was dissolved in DCM (30 mL), washed with water (3 x 15 mL) and brine (30 mL), dried over anhydrous Na_2SO_4 and evaporated to dryness. The product was purified by column chromatography on silica gel eluting with a mixture of hexane and DCM (from v:v = 1:2 to 1:4) to produce red solids (0.36 g, 0.304 mmol, 78%). ^{1}H NMR (300 MHz, CD_2Cl_2) δ 8.87 (d, J = 7.7 Hz, 1H), 8.74 – 8.65 (m, 1H), 8.27 – 8.18 (m, 1H), 7.93 (d, J = 7.7 Hz, 1H), 7.81 – 7.74 (m, 1H), 7.58 (d, J = 2.1 Hz, 2H), 7.52 (td, J = 6.6, 2.0 Hz, 4H), 7.36 (ddd, J = 15.2, 7.4, 2.3 Hz, 8H), 6.93 (dd, J = 8.4, 2.1 Hz, 2H), 6.09 (d, J = 8.4 Hz, 2H), 5.19 (s, 4H), 4.50 (t, J = 6.8 Hz, 2H), 3.14 (t, J = 6.8 Hz, 2H), 1.99 – 1.84 (m, 24H). ^{13}C NMR (76 MHz, CD_2Cl_2) δ 171.46, 171.23, 163.98, 163.68, 144.05, 142.06, 139.33, 138.52, 134.19, 133.27, 132.77, 132.12, 130.85, 130.79, 130.72, 130.16, 129.95, 129.88, 129.58, 128.83, 128.52, 128.26, 127.60, 127.30, 127.17, 124.03, 123.41, 114.03, 66.18, 66.01, 56.19, 56.05, 36.28, 34.53, 33.01, 32.73, 30.62. ESI-MS: m/z of adduct $(M+Na)^+$, calcd. For $C_{61}H_{55}Br_3N_2O_8$, 1203.1401; found, 1203.1401. See **Supplementary Fig. 13.**

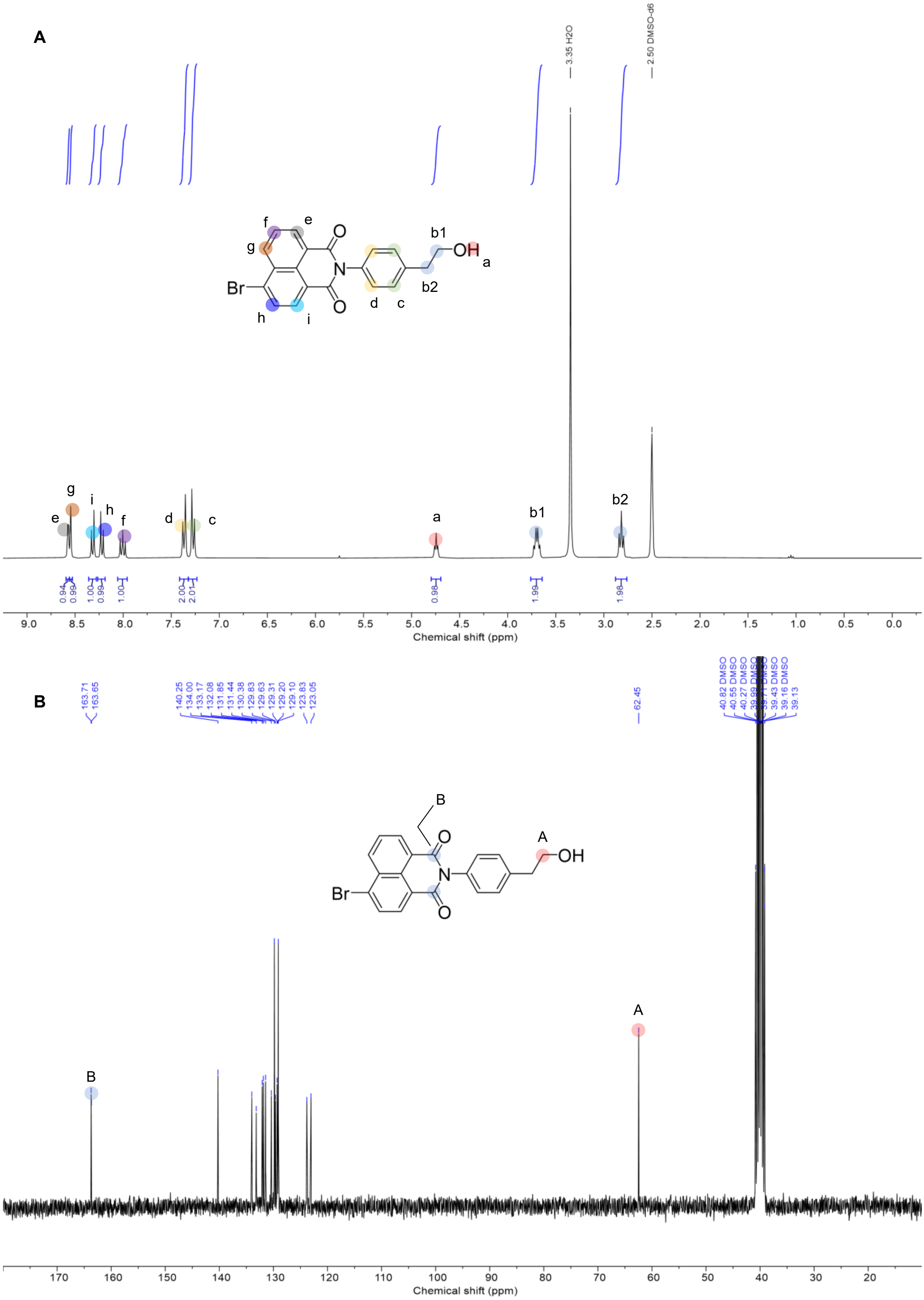

Supplementary Fig. 7: Structural characterization of R1. (**A**) ^{1}H NMR and (**B**) ^{13}C NMR spectrum in DMSO-d_6.

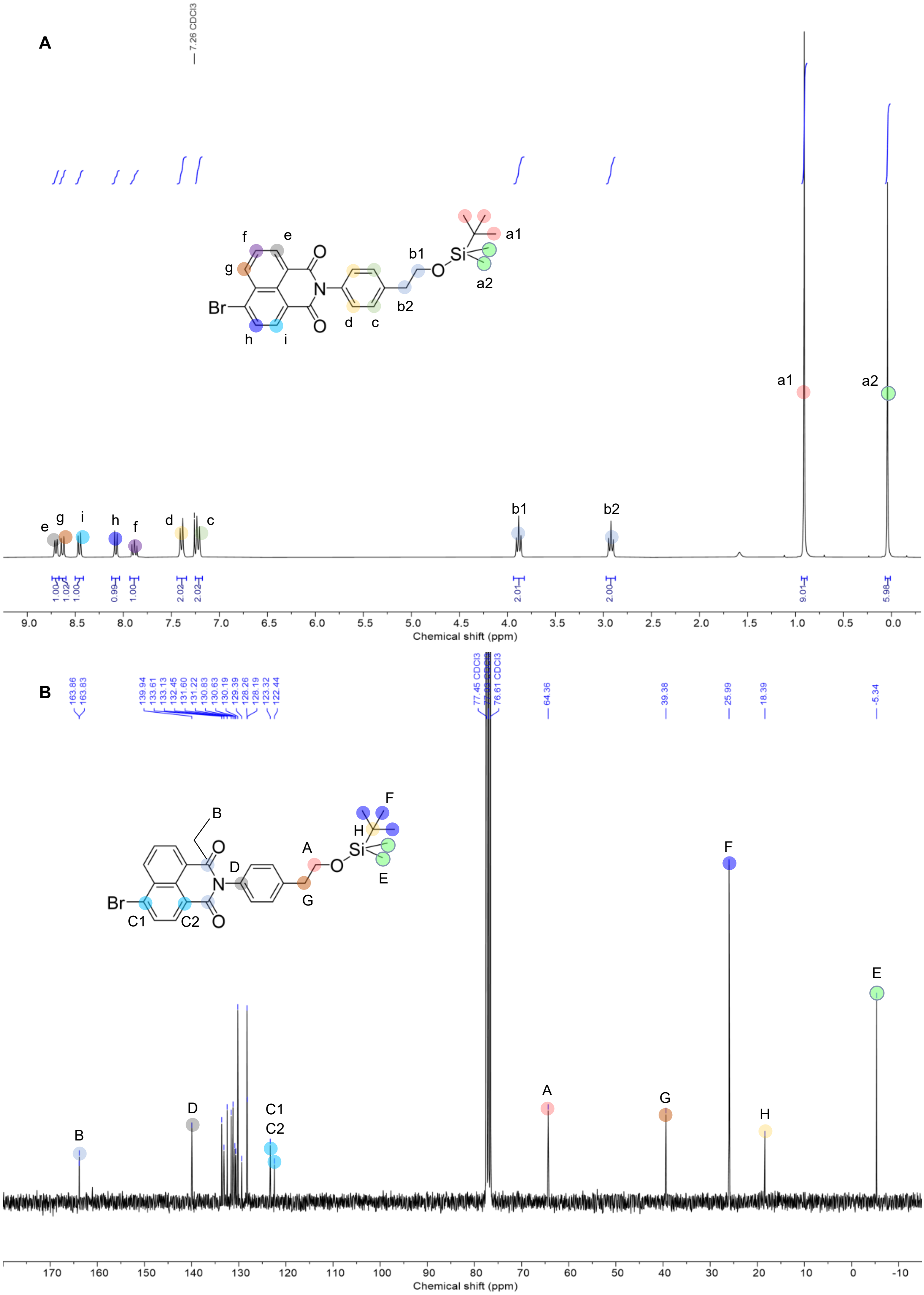

Supplementary Fig. 8: Structural characterization of R2. (**A**) ^{1}H NMR and (**B**) ^{13}C NMR spectrum in $CDCl_3$-*d*.

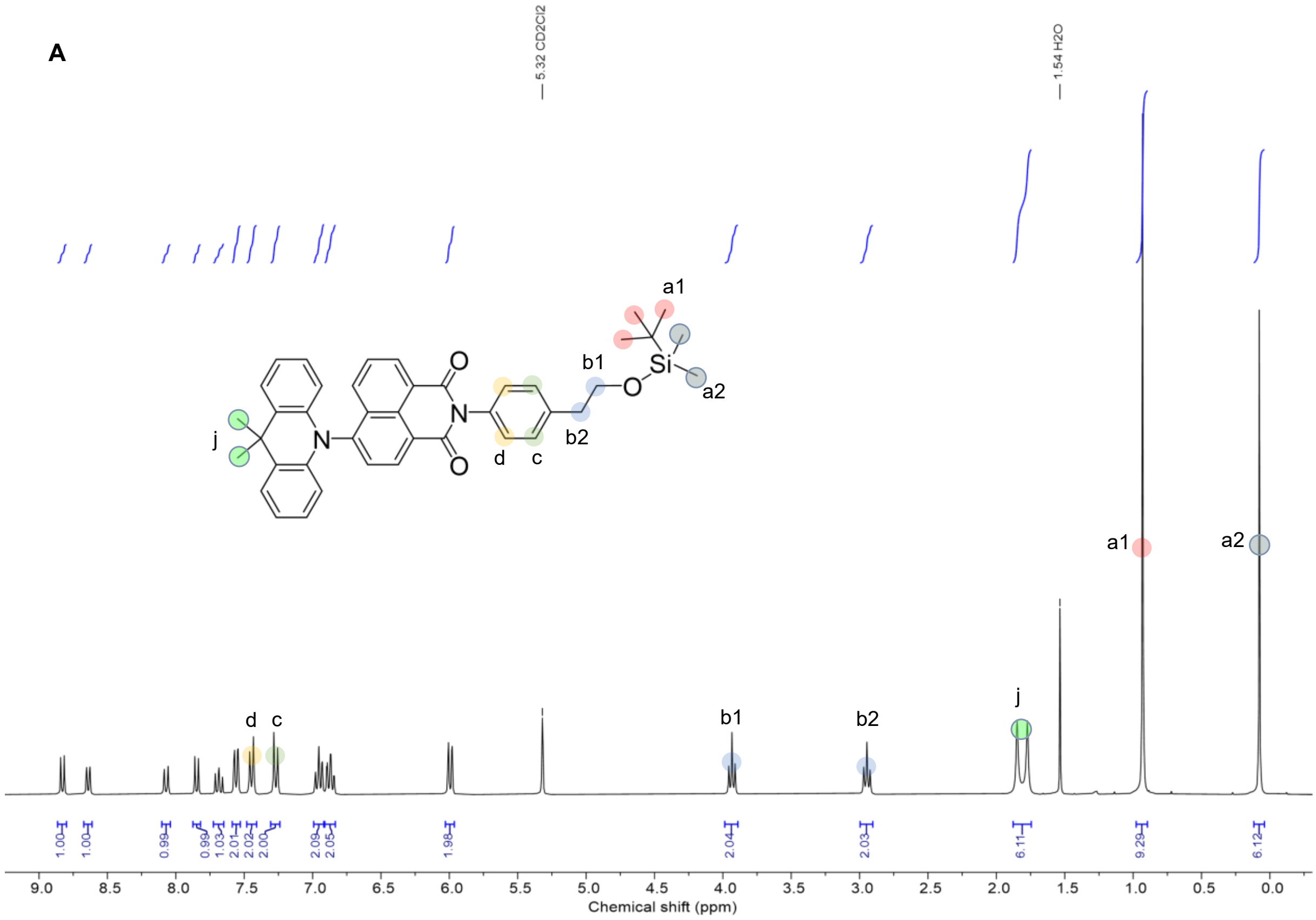

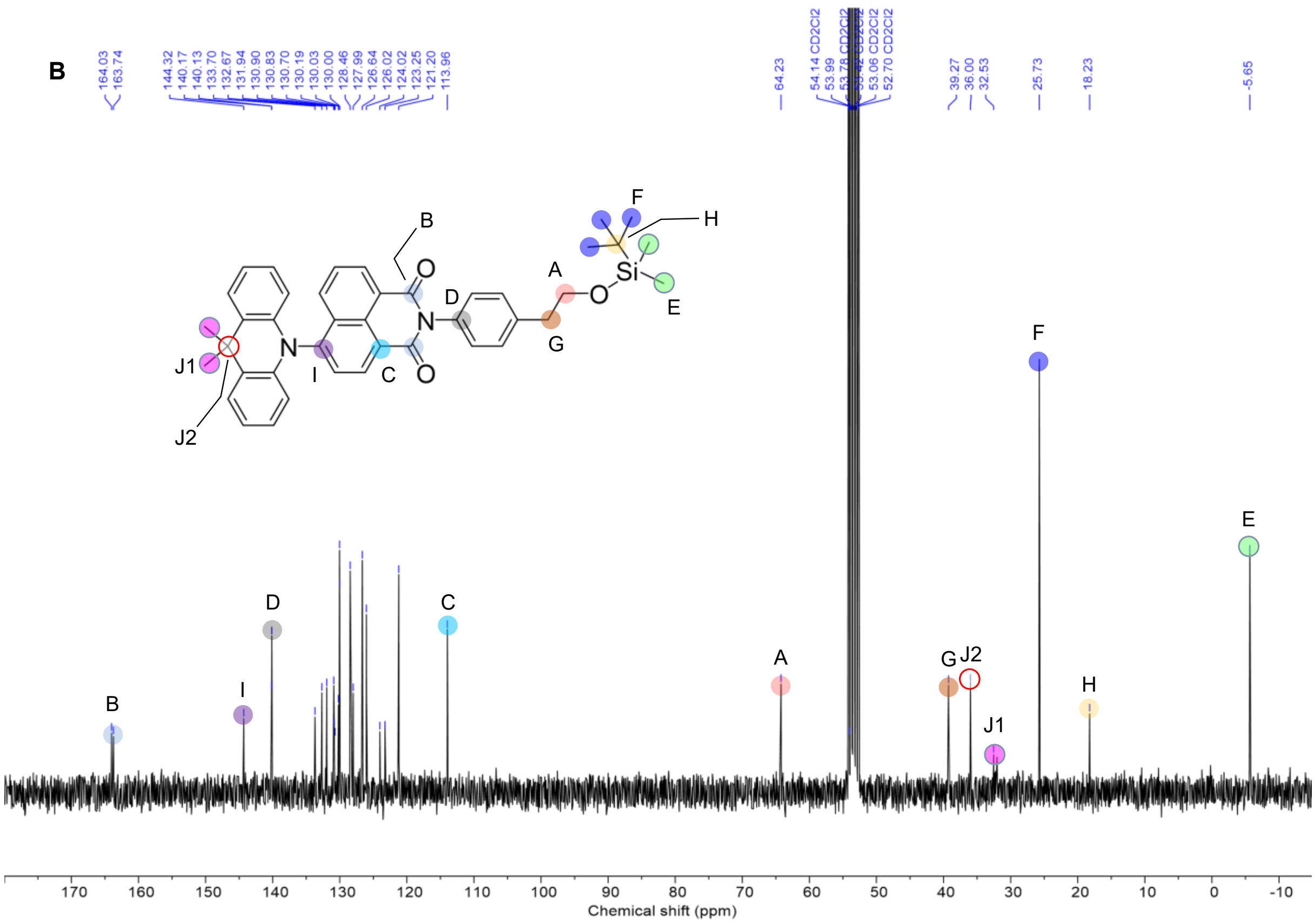

Supplementary Fig. 9: Structural characterization of R3. (**A**) ^{1}H NMR and (**B**) ^{13}C NMR spectrum in CD_2Cl_2-d_2.

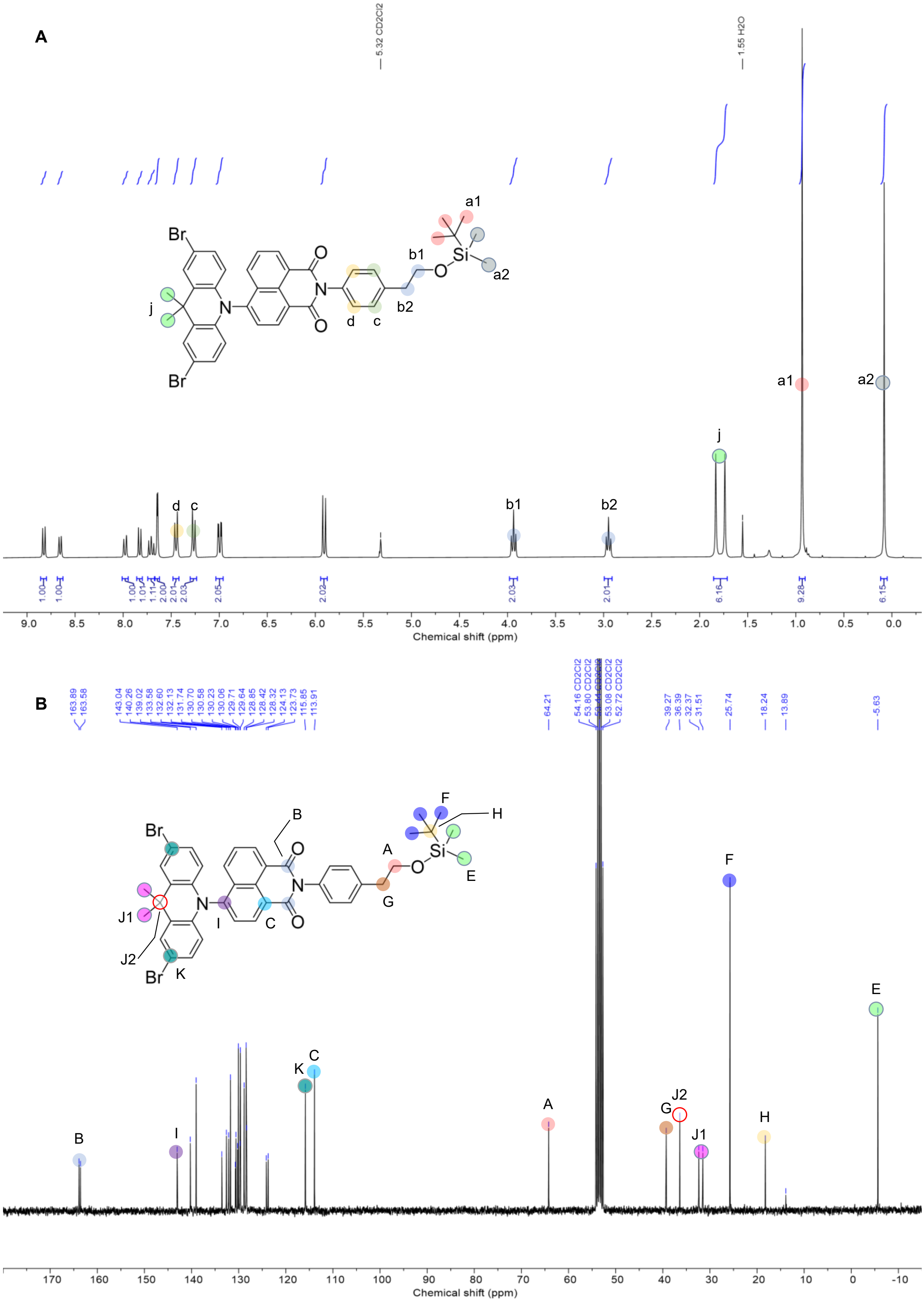

Supplementary Fig. 10: Structural characterization of R4. (**A**) ^{1}H NMR and (**B**) ^{13}C NMR spectrum in CD_2Cl_2-d_2.

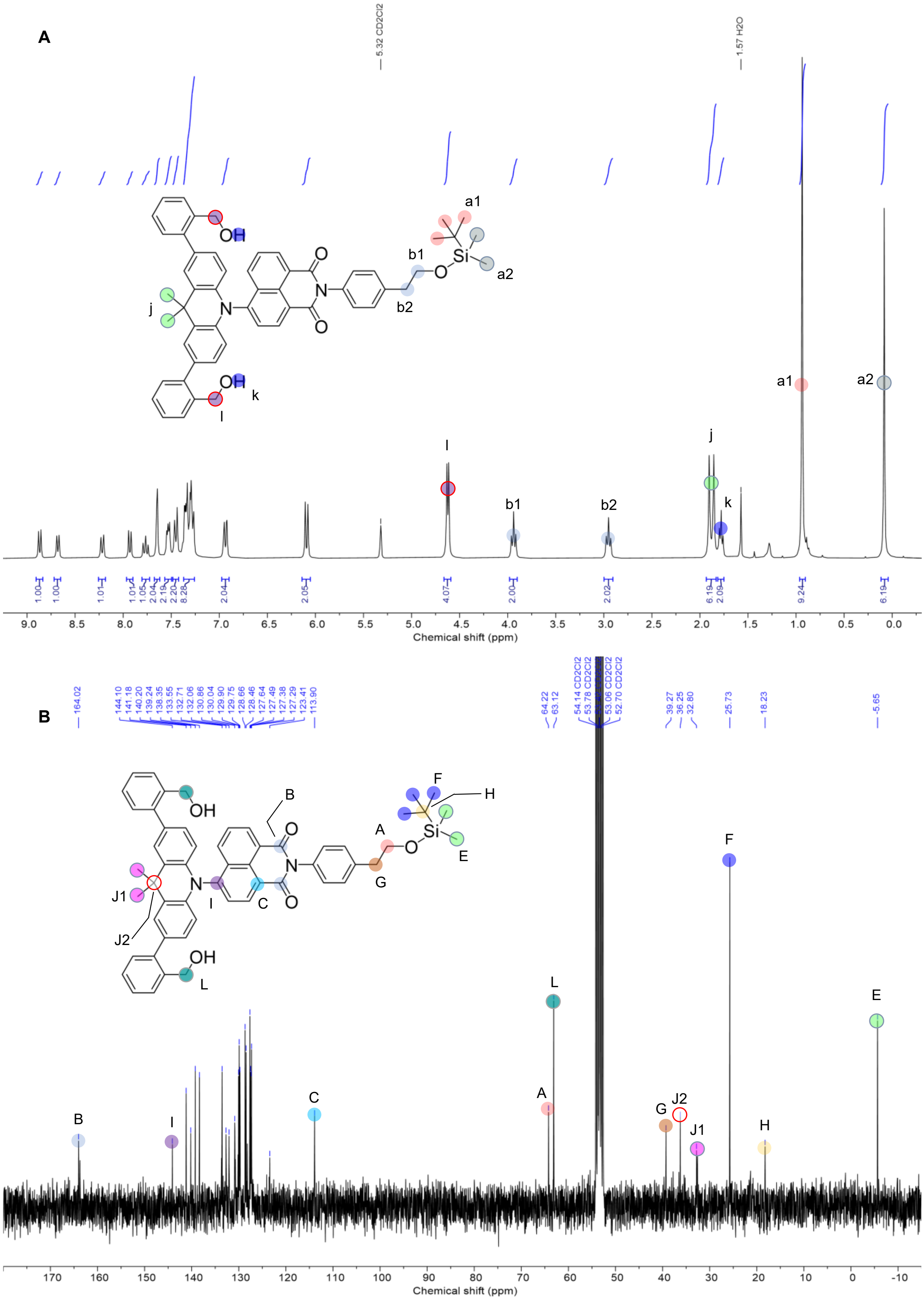

Supplementary Fig. 11: Structural characterization of R5. (**A**) ^{1}H NMR and (**B**) ^{13}C NMR spectrum in CD_2Cl_2-d_2.

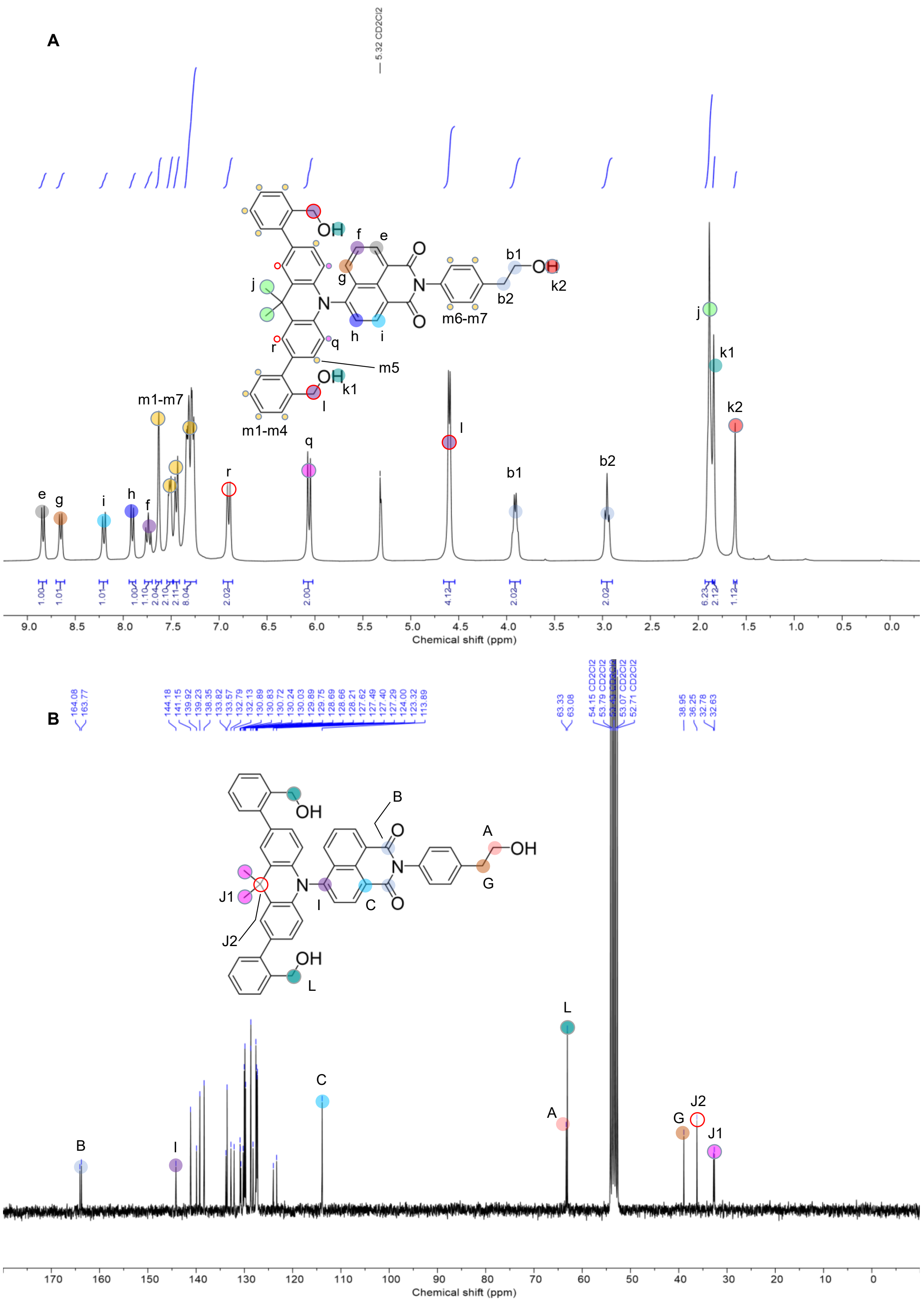

Supplementary Fig. 12: Structural characterization of R6. (**A**) ^{1}H NMR and (**B**) ^{13}C NMR spectrum in CD_2Cl_2-d_2.

A

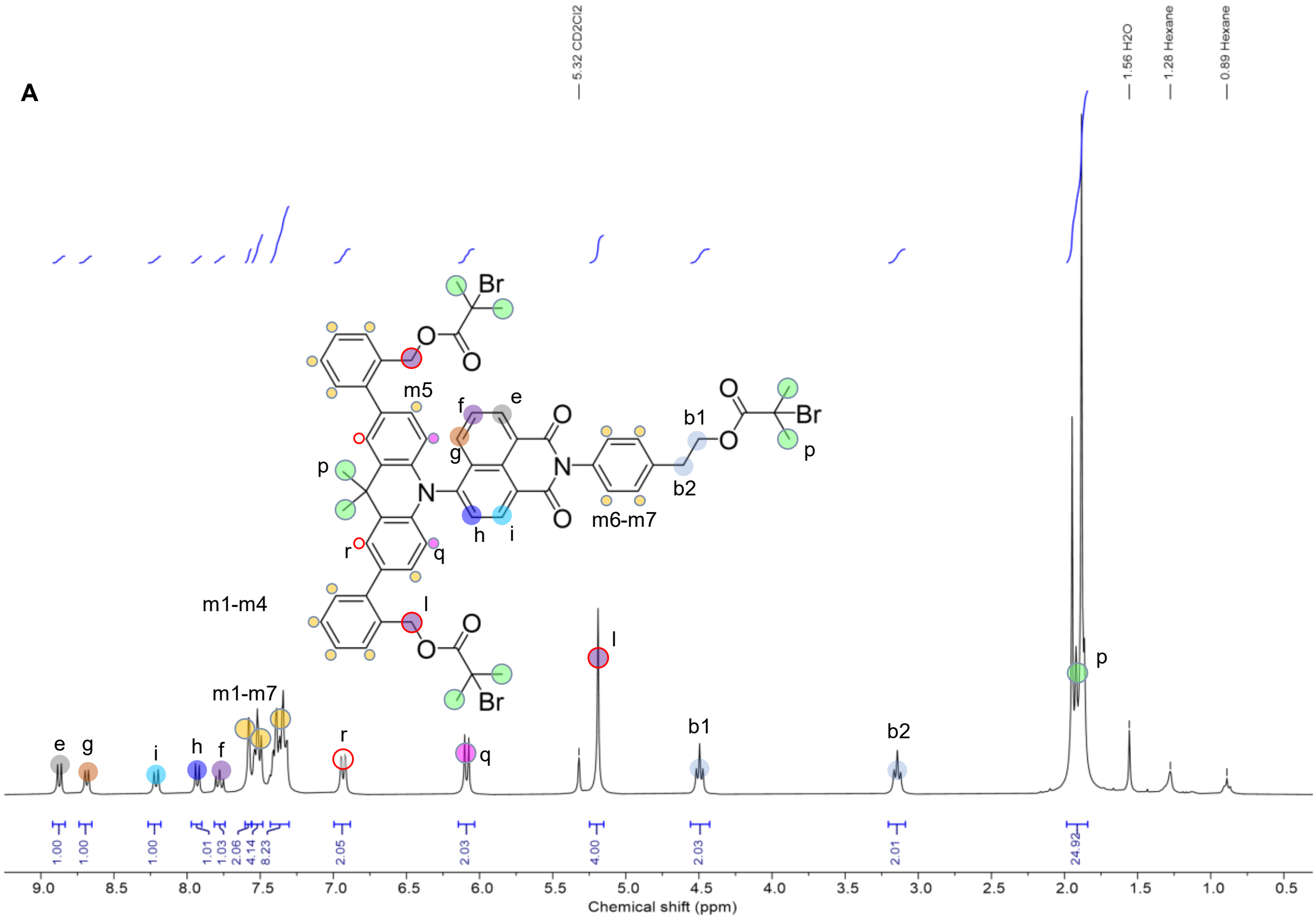

B

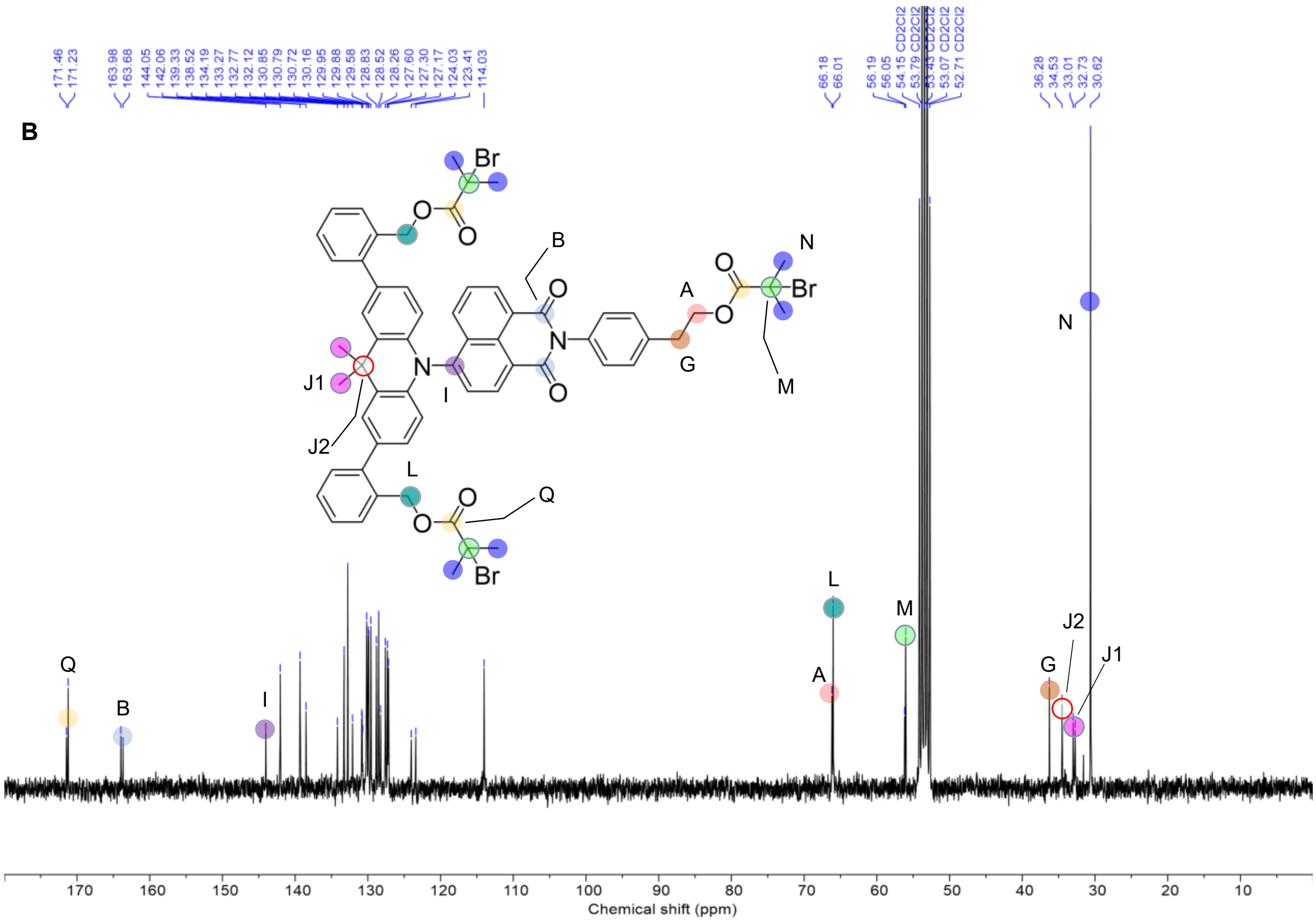

Supplementary Fig. 13: Structural characterization of red-TADF initiator. (**A**) ^{1}H NMR and (**B**) ^{13}C NMR spectrum in CD_2Cl_2-d_2.

Supplementary Note 4 | Synthetic route of blue-TADF-based initiator for ATRP

Please see **Supplementary Fig. 14.**

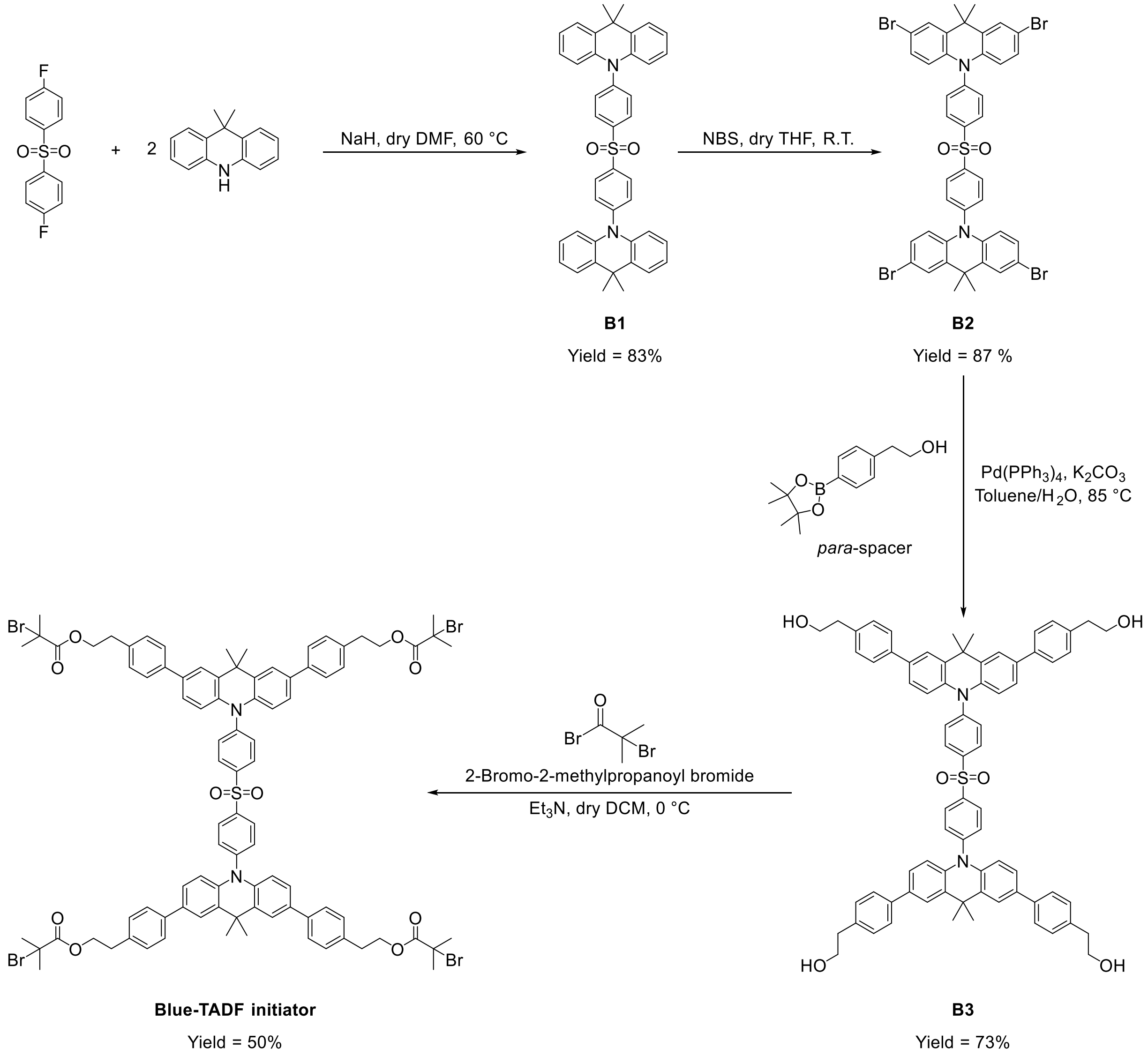

Supplementary Fig. 14: Synthetic route of blue-TADF initiator.

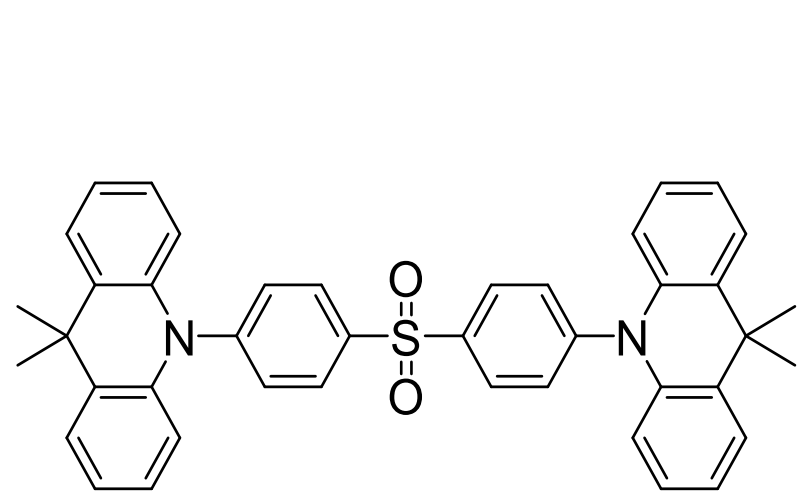

Synthesis of **B1 (DMAC-DPS)**

A 100 mL two-necked flask with a stirring bar, was charged with sodium hydride (0.609 g, 25.37 mmol, 6 eq.) and anhydrous DMF (15 mL), under argon atmosphere. Then DMAC (1.754 g, 8,38 mmol, 2.2 eq.) was added to the solution under 0°C. After the dissolution is completed, a solution of 4,4'-difluorobiphenyl sulphone (0.968 g, 3.81 mmol, 1 eq.) in anhydrous DMF (10 mL) was added dropwise under 0°C. Thereafter, the mixture was heated to 60°C and stirred

overnight. After cooling down to room temperature, water (30 mL) was poured into the mixture for quenching. The crude was extracted with DCM (50 mL), washed with brine (2 x 30 mL), dried over anhydrous Na_2SO_4 and evaporated to dryness. The crude residue was precipitated from a THF/MeOH mixture. The resulting solid was collected via filtration, washed with MeOH, and dried to afford the product (1.99 g, 3.14 mmol, 83%). ^{1}H NMR (300 MHz, $CDCl_3$) δ 8.29 – 8.19 (m, 4H), 7.62 – 7.54 (m, 4H), 7.52 – 7.44 (m, 4H), 7.11 – 6.93 (m, 8H), 6.42 – 6.28 (m, 4H), 1.67 (s, 12H). ^{13}C NMR (76 MHz, $CDCl_3$) δ 146.85, 140.25, 139.92, 131.99, 130.59, 130.53, 126.46, 125.41, 121.84, 115.23, 77.24, 36.28, 30.70. See **Supplementary Fig. 15.**

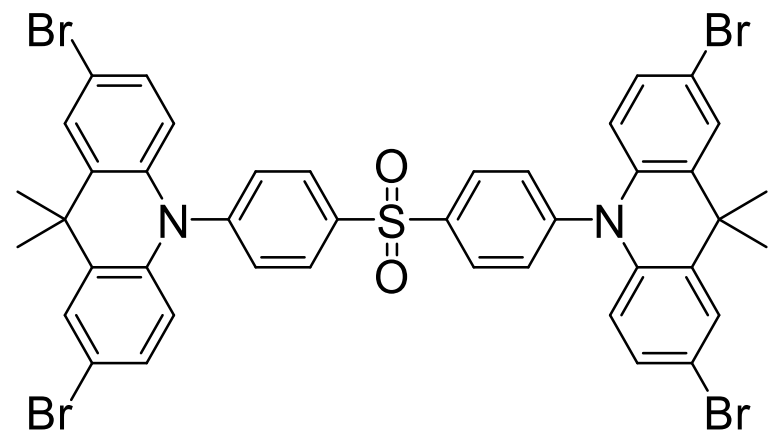

Synthesis of **B2**

A 250 mL round bottomed flask with a stirring bar, was charged with **B1** (2 g, 3.16 mmol, 1 eq.) and anhydrous THF (15 mL). A solution of NBS (2.69 g, 15.2 mmol, 4.8 eq.) in anhydrous THF (15 mL) was added dropwise to the mixture under 0 °C ice bath. The mixture was stirred under room temperature overnight. The residue was dissolved in DCM (30 mL) with water (2 x 15 mL), washed with brine (2 x 20 mL), dried over anhydrous Na_2SO_4 and evaporated to dryness. The crude residue was precipitated from a THF/MeOH mixture. The resulting solid was collected via filtration, washed with MeOH, and dried to afford the brownish pale solid product (2.6 g, 2.74 mmol, 87%). ^{1}H NMR (300 MHz, CD_2Cl_2) δ 8.33 – 8.26 (m, 4H), 7.60 – 7.50 (m, 8H), 7.09 (dd, J = 8.8, 2.3 Hz, 4H), 6.15 (d, J = 8.8 Hz, 4H), 1.64 (s, 12H). ^{13}C NMR (76 MHz, CD_2Cl_2) δ 145.67, 141.13, 139.16, 132.63, 131.77, 130.97, 129.31, 128.35, 116.14, 114.08, 36.38, 30.52. See **Supplementary Fig. 16.**

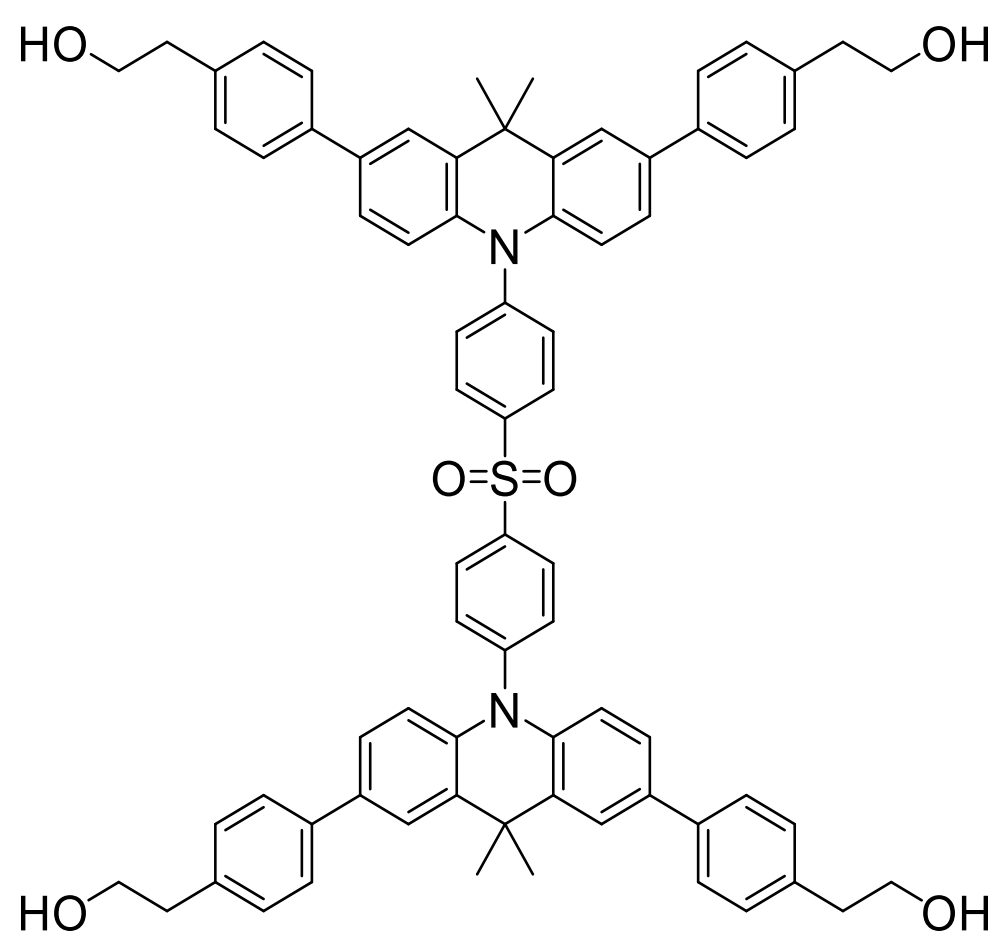

Synthesis of **B3**

A 100 mL schlenk flask equipped with a stirring bar, was charged with **B2** (0.75 g, 0.79 mmol, 1 eq.), 4-(4,4,5,5-Tetramethyl-1,3,2-dioxaborolan-2-yl)benzeneethanol (*para*-spacer, 0.94 g, 3.16 mmol, 4.8 eq.), K_2CO_3 (2.10 g, 15.8 mmol, 20 eq.), and $Pd(PPh_3)_4$ (0.073 g, 0.063 mmol, 0.08 eq.). After drying under vacuum for 30 minutes, the flask with solids was flushed three

times with argon. Degassed mixture of dioxane (40 mL) and water (10 mL) with argon bubbling was added to the flask. The mixture was heated to 85 °C and reacted for 36 hours. After cooling down to room temperature, the residue was diluted in DCM (30 mL), washed with water (2 x 20 mL) and brine (2 x 20 mL), dried over anhydrous Na_2SO_4 and evaporated to dryness. The crude solids were dissolved in THF and filtered through a short Al_2O_3 column. The crude residue was precipitated from a THF/MeOH mixture. The resulting solid was collected via filtration, washed with MeOH, and dried to afford the product (0.65 g, 0.58 mmol, 73%). ^{1}H NMR (300 MHz, DMSO) δ 8.39 (d, *J* = 8.5 Hz, 4H), 7.84 – 7.70 (m, 8H), 7.59 – 7.44 (m, 8H), 7.34 – 7.18 (m, 12H), 6.29 (d, *J* = 8.5 Hz, 4H), 4.67 (s, 4H), 3.64 – 3.58 (m, 8H), 2.78 – 2.70 (m, 8H), 1.76 (s, 12H). ^{13}C NMR (76 MHz, DMSO) δ 140.50, 139.24, 138.57, 138.19, 133.91, 131.94, 131.73, 131.25, 129.88, 129.37, 128.68, 126.44, 125.32, 124.45, 115.56, 62.69, 36.59, 31.91. See **Supplementary Fig. 17.**

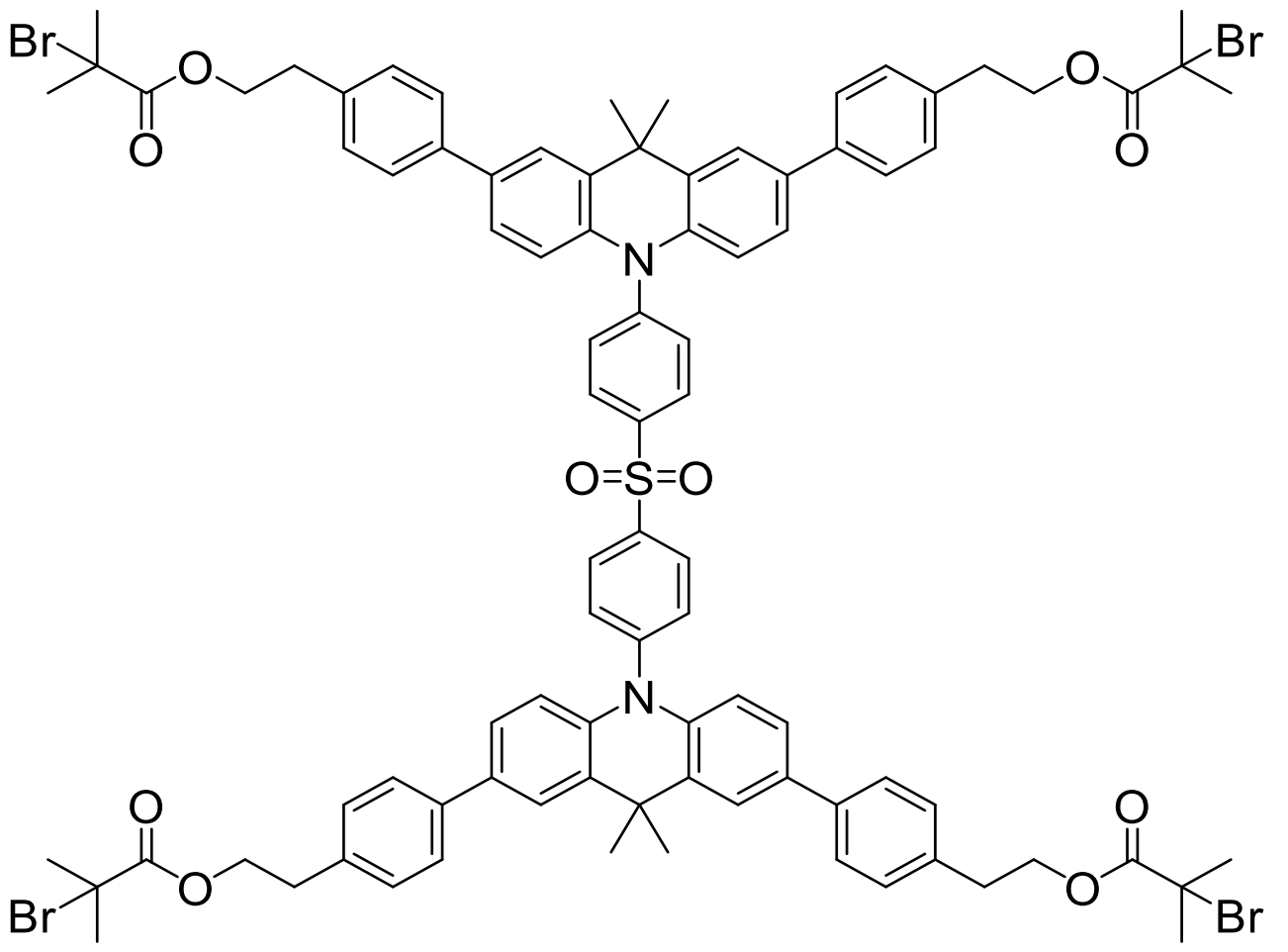

Synthesis of **blue-TADF initiator**

A 100 ml flask was equipped with a stirring bar, was charged with **B3** (0.2 g, 0.179 mmol, 1 eq.), triethylamine (Et_3N, 199 µL, 1.44 mmol, 8 eq.) and anhydrous DCM (10 mL) under N_2. 2-bromoisobutyryl bromide (106 µL, 0.86 mmol, 4.8 eq.) in anhydrous DCM (2.5 mL) was added dropwise to the solution under 0 °C ice bath. The mixture was stirred overnight at room temperature. The residue was dissolved in DCM (10 mL), washed with water (2 x 10 mL) and brine (15 mL), dried over anhydrous Na_2SO_4 and evaporated to dryness. The product was purified by column chromatography on silica gel eluting with a 1:1 mixture of hexane and DCM to produce solids (0.153 g, 0.089 mmol, 50%). ^{1}H NMR (300 MHz, $CDCl_3$) δ 8.36 – 8.29 (m, 4H), 7.73 – 7.61 (m, 8H), 7.53 – 7.47 (m, 8H), 7.34 – 7.26 (m, 10H), 7.24 (d, *J* = 2.1 Hz, 2H), 6.40 (d, *J* = 8.5 Hz, 4H), 4.41 (t, *J* = 7.0 Hz, 8H), 3.03 (t, *J* = 7.0 Hz, 8H), 1.91 (s, 24H), 1.80 (s, 12H). ^{13}C NMR (76 MHz, $CDCl_3$) δ 171.64, 146.63, 140.37, 139.39, 136.08, 134.41, 131.77, 131.19, 130.73, 129.49, 126.74, 125.15, 124.35, 115.30, 77.23, 66.45, 55.82, 36.56, 34.48, 31.32, 30.80. ESI-MS: m/z of adduct $(M+Na)^+$, calcd. For $C_{90}H_{88}Br_4N_2O_{10}S$, 1709.3702; found, 1708.9460. See **Supplementary Fig. 18.**

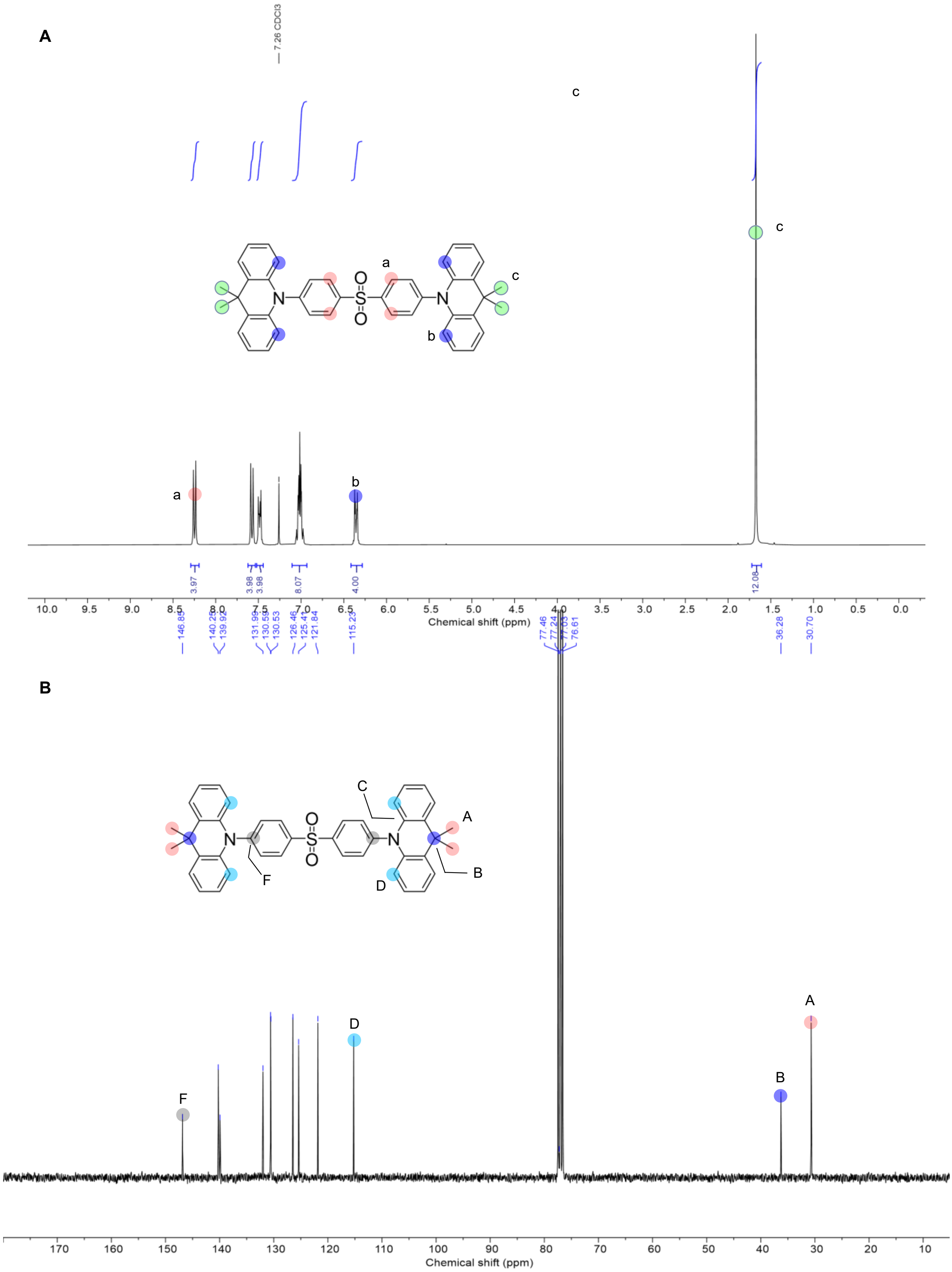

Supplementary Fig. 15: Structural characterization of B1 (DMAC-DPS). (**A**) ^{1}H NMR and (**B**) ^{13}C NMR spectrum in $CDCl_3$-*d*.

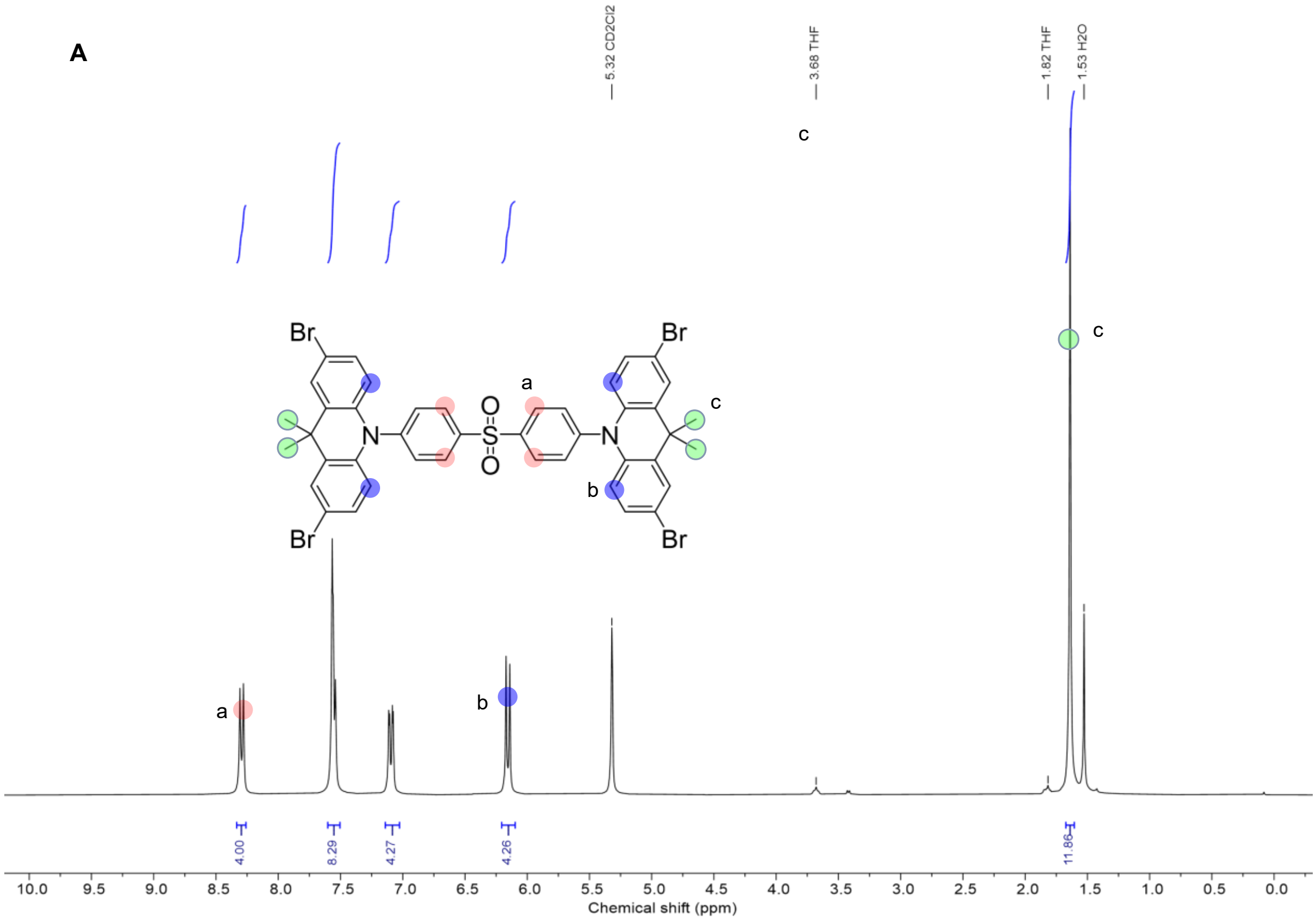

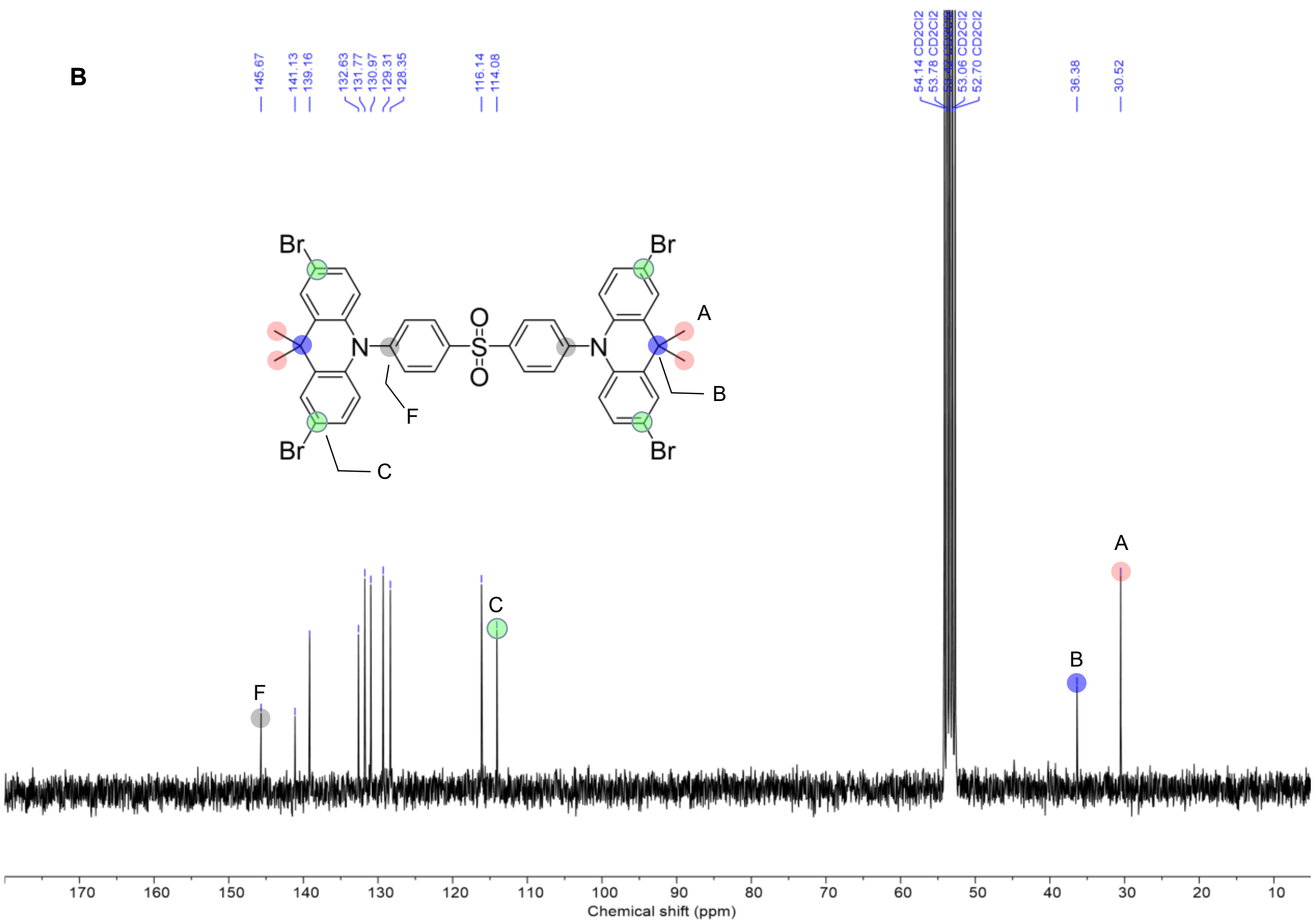

Supplementary Fig. 16: Structural characterization of B2. (**A**) ^{1}H NMR and (**B**) ^{13}C NMR spectrum in CD_2Cl_2-d_2.

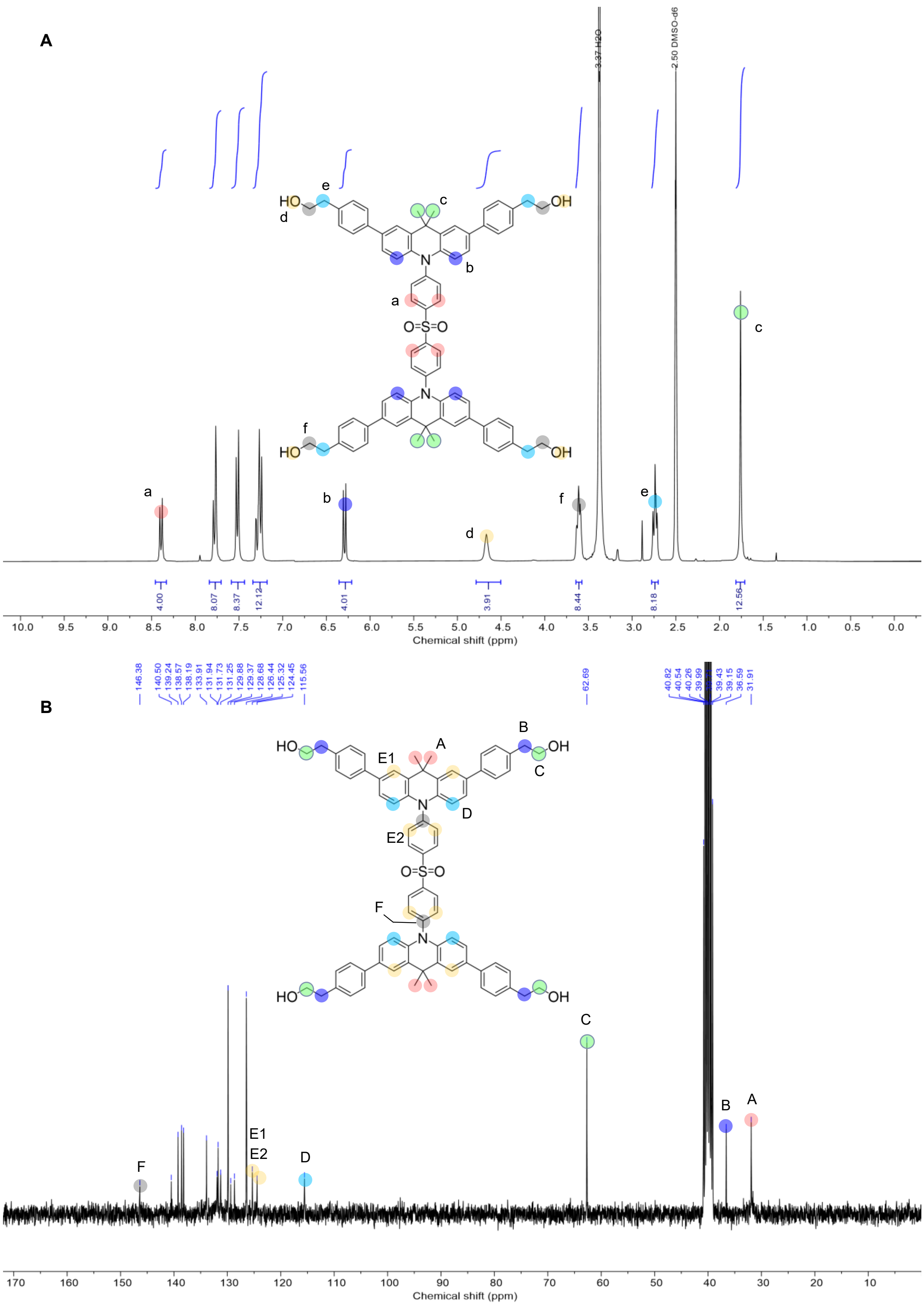

Supplementary Fig. 17: Structural characterization of B3. (**A**) ^{1}H NMR and (**B**) ^{13}C NMR spectrum in DMSO-d_6.

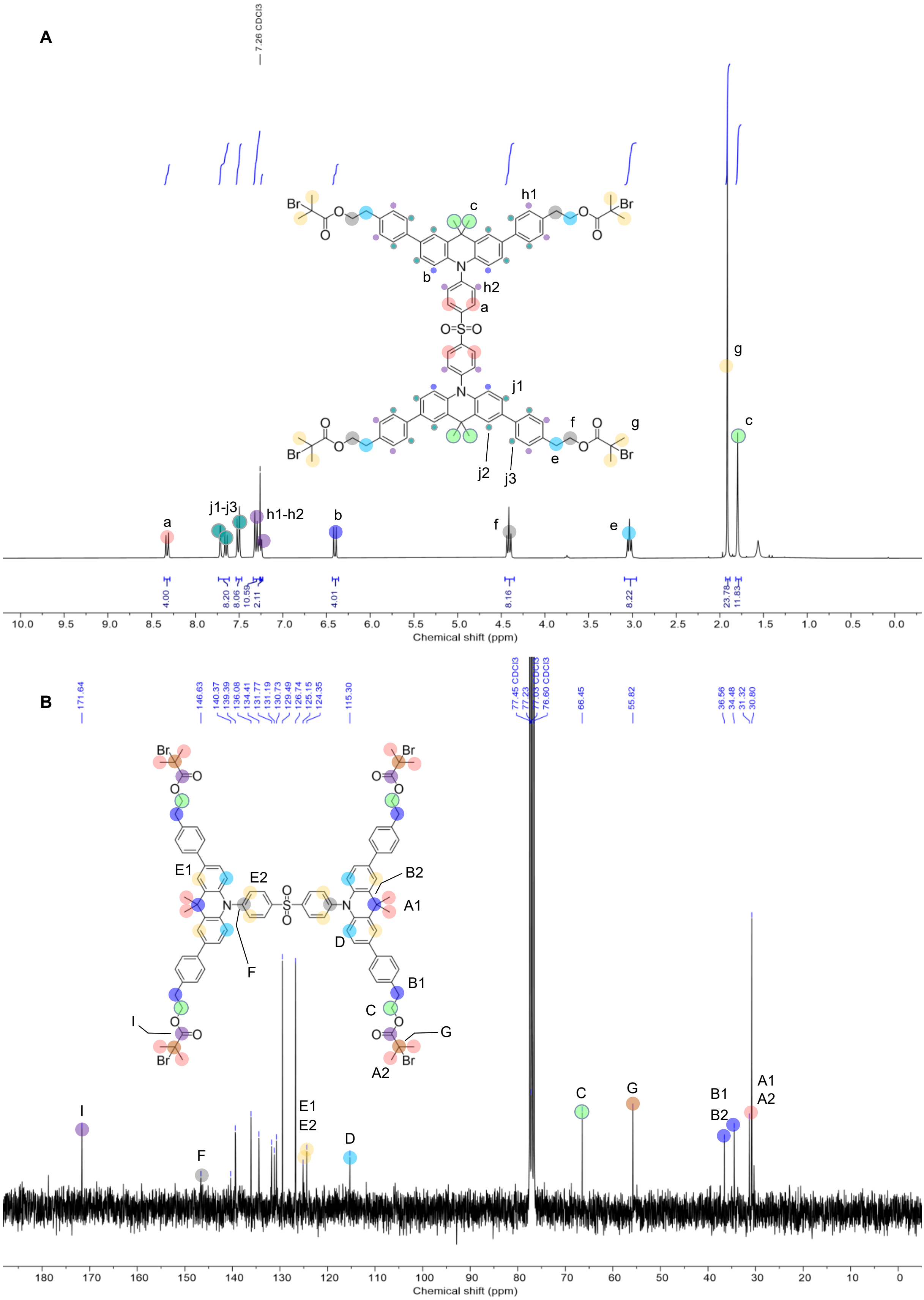

Supplementary Fig. 18: Structural characterization of blue-TADF initiator. (**A**) ^{1}H NMR and (**B**) ^{13}C NMR spectrum in $CDCl_3$-*d*.

Supplementary Note 5 | Synthetic route of AcryCz host monomer for ATRP

Please see **Supplementary Fig. 19.**

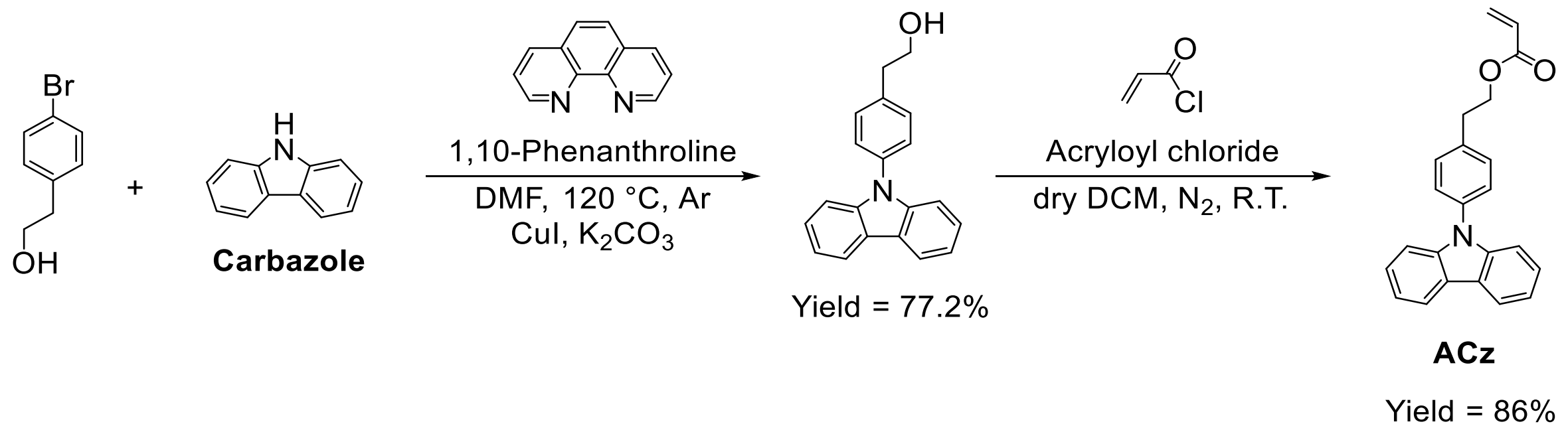

Supplementary Fig. 19: Synthetic route of AcryCz host monomer.

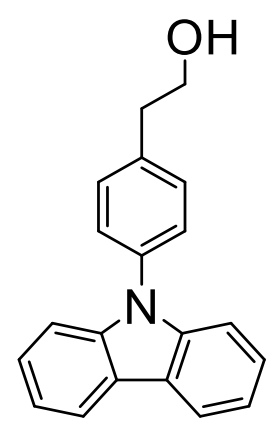

Synthesis of **2-(4-(9H-carbazol-9-yl)phenyl)ethan-1-ol**[3]

A 250 mL two-necked flask equipped with a stirring bar, was charged with carbazole (2.49 g, 14.9 mmol, 1.5 eq.), K_2CO_3 (2.12 g, 29.8 mmol, 3 eq.), CuI (2.84 g, 14.9 mmol, 1.6 eq.) and 1,10-Phenanthroline (0.287 g, 1.59 mmol, 0.16 eq.). After drying under vacuum for 30 minutes, the flask with solids was flushed three times with argon. DMF (40 mL) was then added to the flask. An orange suspension was formed and 4-bromophenylethyl alcohol (1.99 g, 9.95 mmol, 1 eq.) was further added. The reaction mixture was heated to 120 °C for 24 hours, with a reflux condenser. After cooling down to room temperature, the residue was dissolved in DCM (100 mL), washed with water (3 x 10 mL), dried over anhydrous Na_2SO_4 and evaporated to dryness. The product was purified by column chromatography on silica gel eluting with a 10:1 mixture of DCM and MeOH to produce a white solid (2.27 g, 7.89 mmol, 77.2%). ^{1}H NMR (300 MHz, $CDCl_3$) δ 8.16 (dt, J = 7.8, 1.0 Hz, 2H), 7.52 (d, J = 8.4 Hz, 2H), 7.47 (d, J = 8.5 Hz, 2H), 7.44 – 7.37 (m, 4H), 7.29 (dt, J = 8.0, 4.1 Hz, 2H), 3.99 (q, J = 6.3 Hz, 2H), 3.01 (t, J = 6.5 Hz, 2H), 1.53 (t, J = 5.8 Hz, 1H). ^{13}C NMR (76 MHz, $CDCl_3$) δ 140.94, 130.47, 127.23, 125.91, 123.33, 120.31, 119.87, 109.80, 77.26 (d, J = 32.0 Hz), 76.62, 63.59, 38.89. See **Supplementary Fig. 20.**

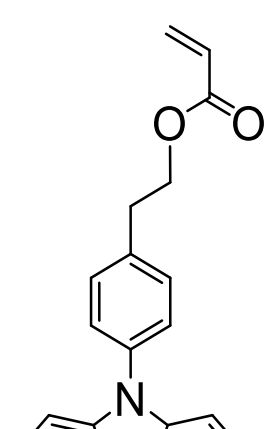

Synthesis of **ACz monomer**

A 100 mL round flask equipped with a stirring bar, was charged with 2-(4-(9H-carbazol-9-yl)phenyl)ethan-1-ol (1.802 g, 6.26 mmol, 1 eq.) and dried under high vacuum for 30 min. Then, dry DCM (40 mL) were added to the flask, followed by Et_3N (1.75 mL, 12.5 mmol, 2 eq.). Then acryloyl chloride (0.7 mL, 7.5 mmol, 1.2 eq.) was added to the solution dropwise under N_2. The solution was stirred at room temperature overnight. The mixture was diluted in DCM (30 mL), washed with water (3 x 10 mL) and brine (50 mL), dried over anhydrous Na_2SO_4 and evaporated to give a crude. The product was purified by column chromatography on silica gel eluting with a 1:1 mixture of hexane and DCM to produce a white solid. The product was recrystallized in MeOH to afford as white crystals (1.84 g, 5.38 mmol, 86%). ^{1}H NMR (300 MHz, $CDCl_3$) δ 8.15 (dt, *J* = 7.7, 1.0 Hz, 2H), 7.56 – 7.45 (m, 4H), 7.45 – 7.37 (m, 4H), 7.36 – 7.23 (m, 2H), 6.45 (dd, *J* = 17.3, 1.5 Hz, 1H), 6.18 (dd, *J* = 17.3, 10.4 Hz, 1H), 5.87 (dd, *J* = 10.4, 1.5 Hz, 1H), 4.49 (t, *J* = 7.0 Hz, 2H), 3.13 (t, *J* = 7.0 Hz, 2H). ^{13}C NMR (76 MHz, $CDCl_3$) δ 140.94, 130.96, 130.38, 128.43, 127.21, 125.92, 123.34, 120.30, 119.88, 109.77, 77.25 (d, *J* = 31.9 Hz), 76.62, 64.83, 34.83. ESI-MS: m/z of adduct $(M+H)^+$, calcd. For $C_{23}H_{19}NO_2$, 342.1489; found, 342.1481. See **Supplementary Fig. 21.**

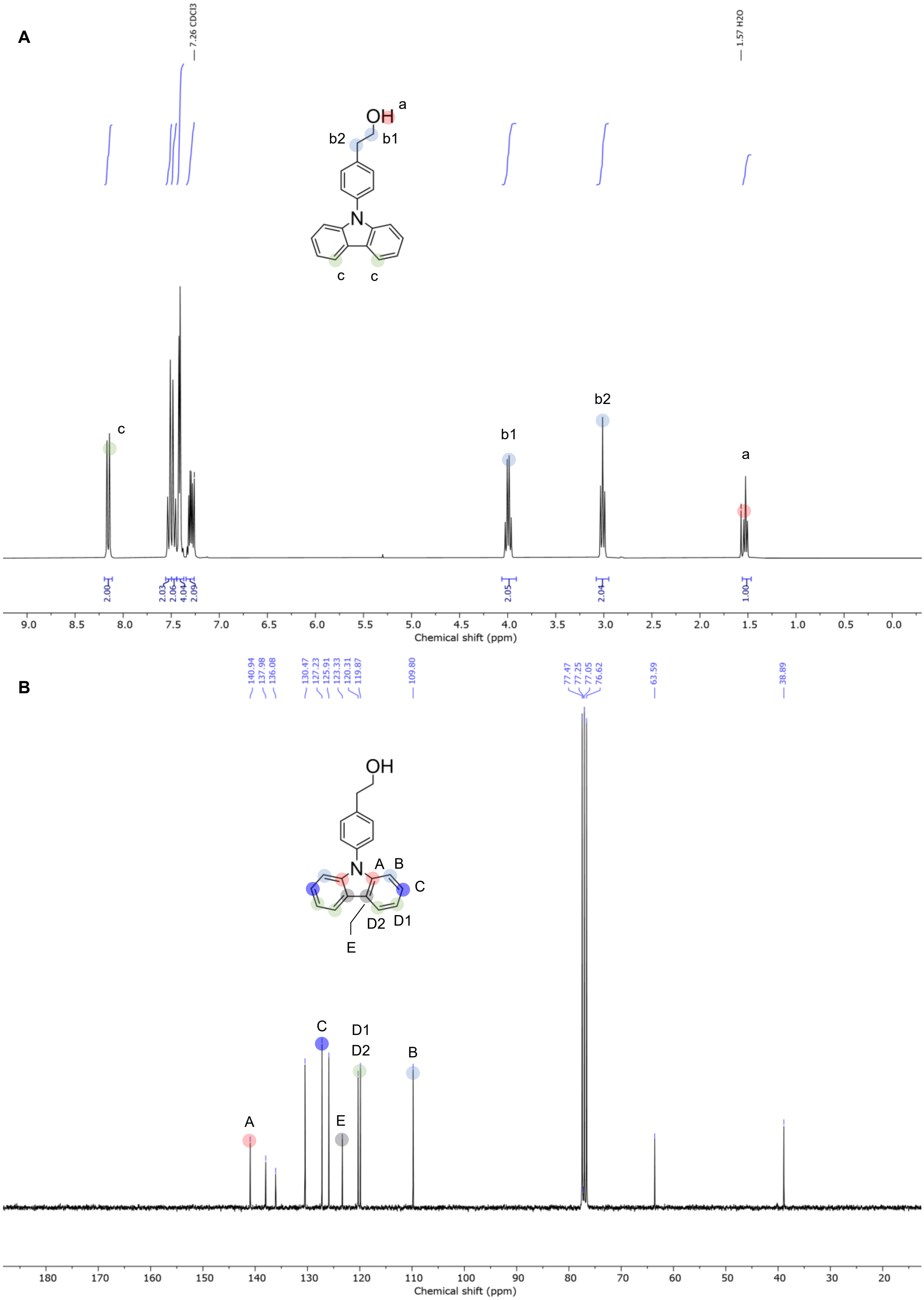

Supplementary Fig. 20: Structural characterization of 2-(4-(9H-carbazol-9-yl)phenyl)ethan-1-ol. (**A**) ^{1}H NMR and (**B**) ^{13}C NMR spectrum in $CDCl_3$-*d*.

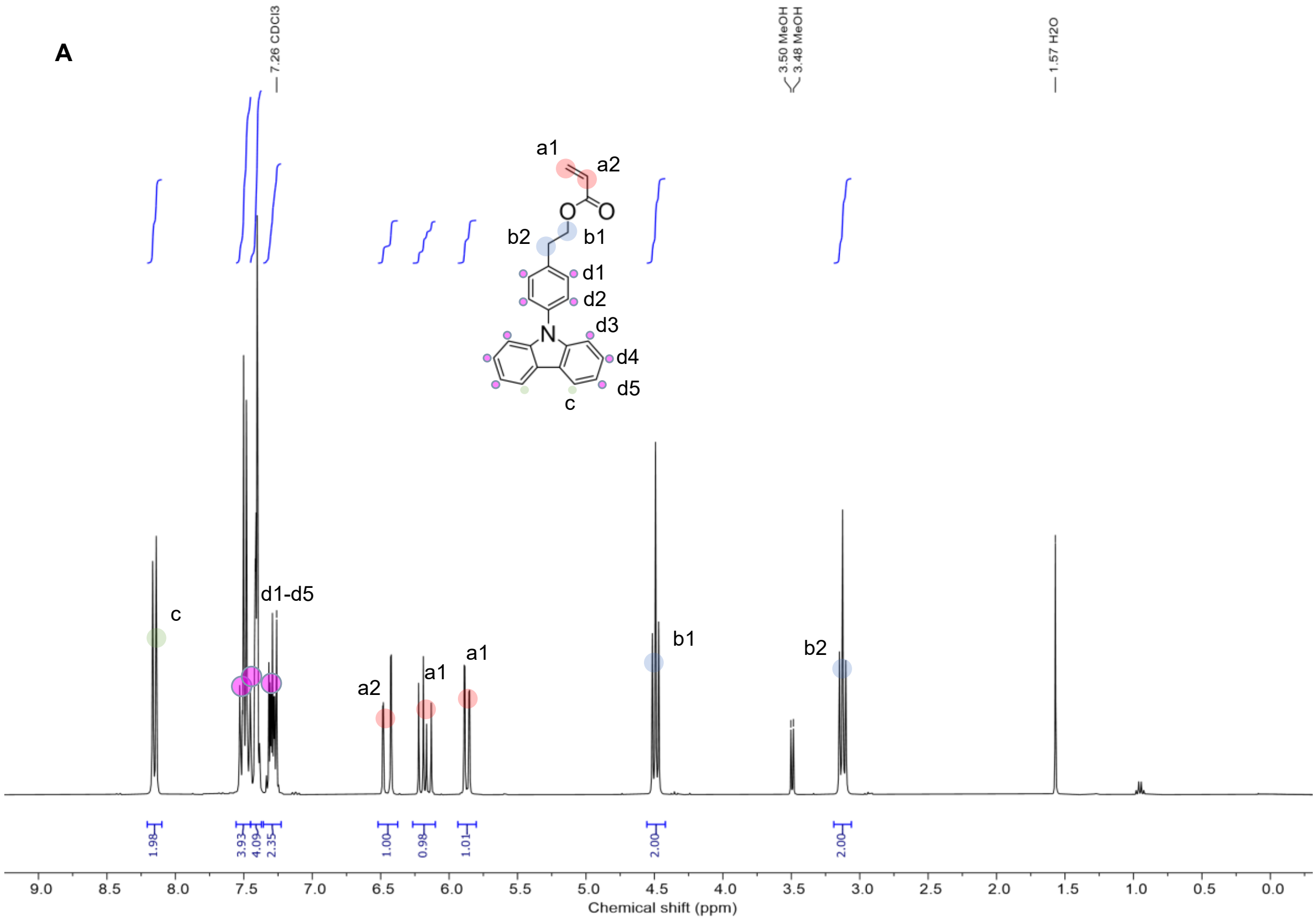

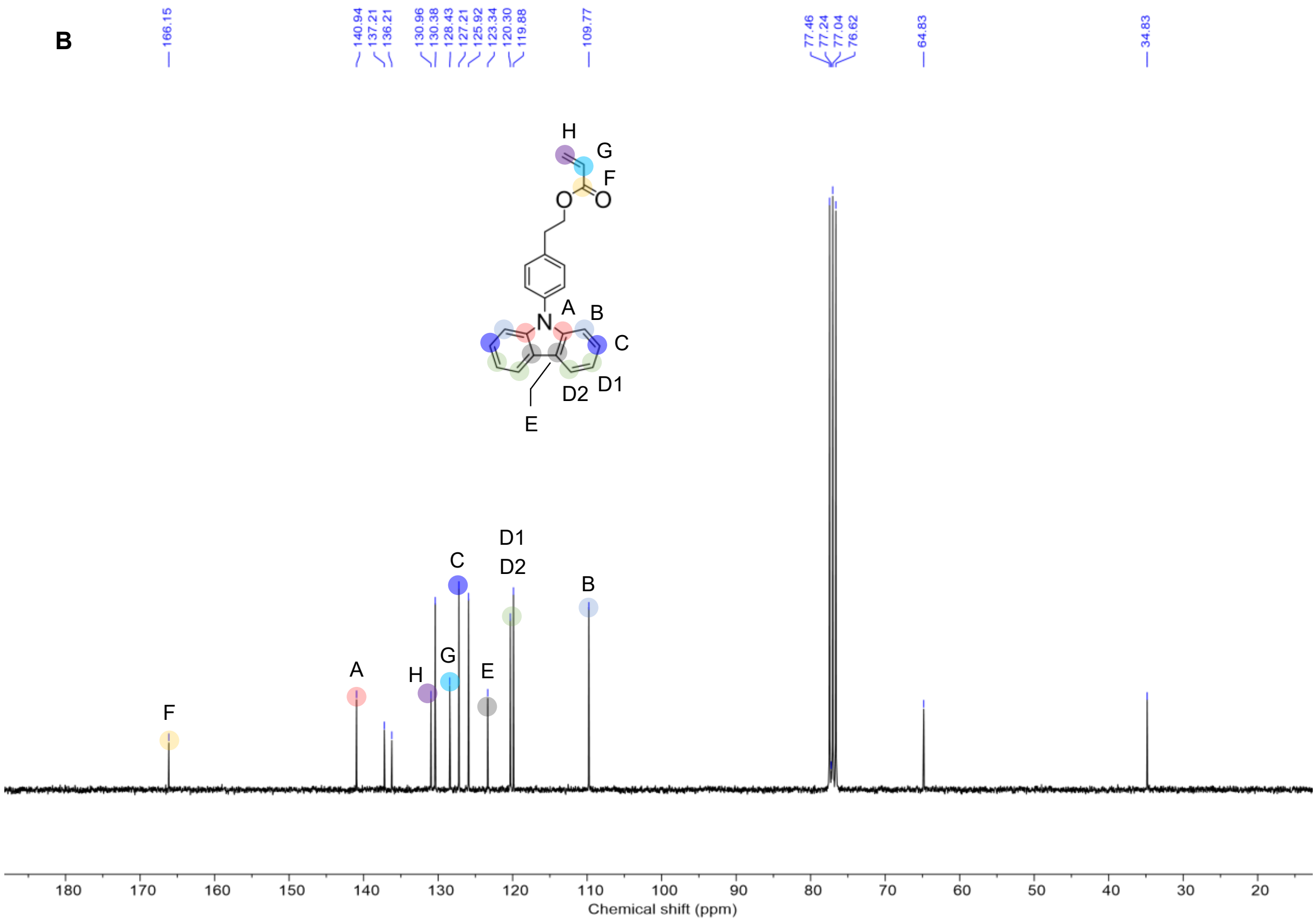

Supplementary Fig. 21: Structural characterization of ACz monomer. (**A**) ^{1}H NMR and (**B**) ^{13}C NMR spectrum in $CDCl_3$-*d*.

Supplementary Note 6 | Synthetic route of cinnamyl photocrosslinkable monomer for ATRP

Please see **Supplementary Fig. 22.**

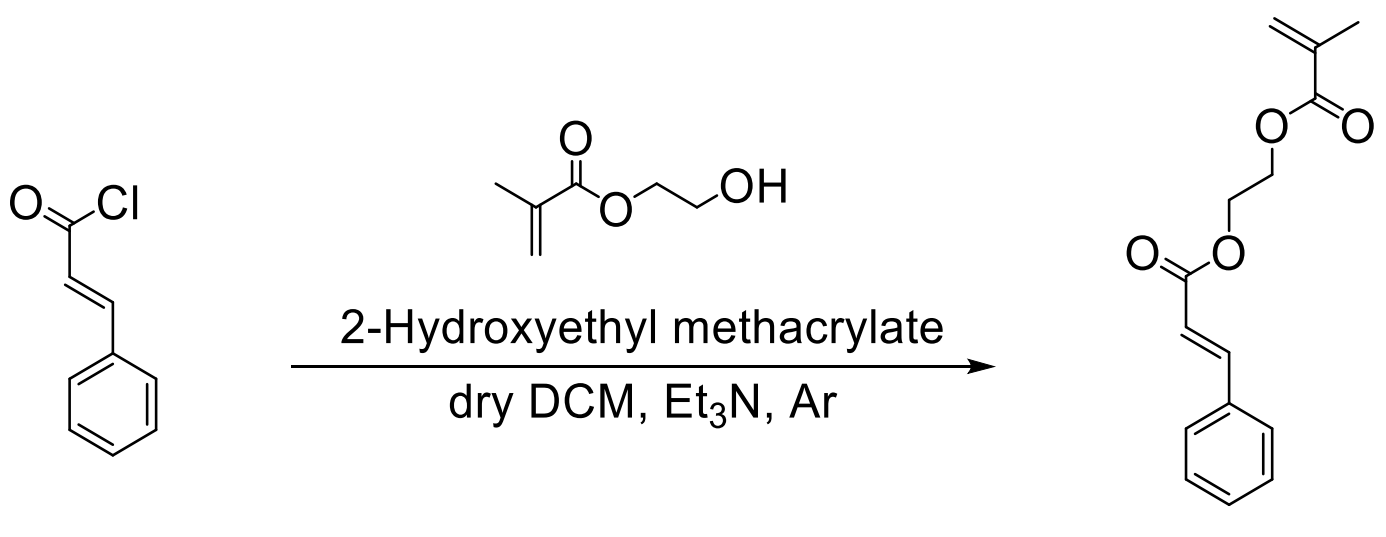

Methacrylate cinnamyl photocrosslinkable monomer (MAc)

Yield = 49%

Supplementary Fig. 22: Synthetic route of Methacrylate cinnamyl photocrosslinkable monomer (MAc).

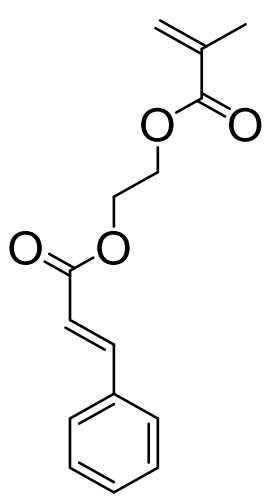

Synthesis of **MAc**

A dry 100 mL round flask equipped with a stirring bar, was charged with cinnamoyl chloride (3 g, 18 mmol, 1 eq.), dry DCM (15 mL), and Et_3N (2.76 mL, 19.81 mmol, 1.1 eq.) under N_2. 2-hydroxyethyl methacrylate (2.343 g, 18.01 mmol, 1 eq.) was added slowly dropwise to the above solution by needle. The solution was stirred at room temperature overnight. The mixture was washed with brine (3 x 20 mL), dried over anhydrous Na_2SO_4 and evaporated to give a crude liquid?. The product was purified by column chromatography on silica gel eluting with a 1:4 mixture of hexane and DCM to produce a yellowish oil. (2.32 g, 8.91 mmol, 49%) Additionally, 4-methoxyphenol (2 mg, 0.016 mmol, 0.1 wt%) in hexane (185 µL) were added to the product oil as the radical inhibitor to prevent premature crosslinking. ^{1}H NMR (300 MHz, DMSO) δ 7.76 – 7.62 (m, 3H), 7.47 – 7.35 (m, 3H), 6.66 (d, *J* = 16.0 Hz, 1H), 6.04 (s, 1H), 5.68 (s, 1H), 4.49 – 4.31 (m, 4H), 1.87 (s, 3H). ^{13}C NMR (76 MHz, DMSO) δ 166.88, 166.55, 145.45, 136.09, 134.38, 131.03, 129.38, 128.88, 126.55, 118.09, 63.01, 62.43, 40.81, 40.53, 40.26, 39.98, 39.70, 39.42, 39.14, 18.38. ESI-MS: m/z of adduct $(M+Na)^+$, calcd. For $C_{15}H_{16}O_4$, 283.0941; found, 283.0938. See **Supplementary Fig. 23.**

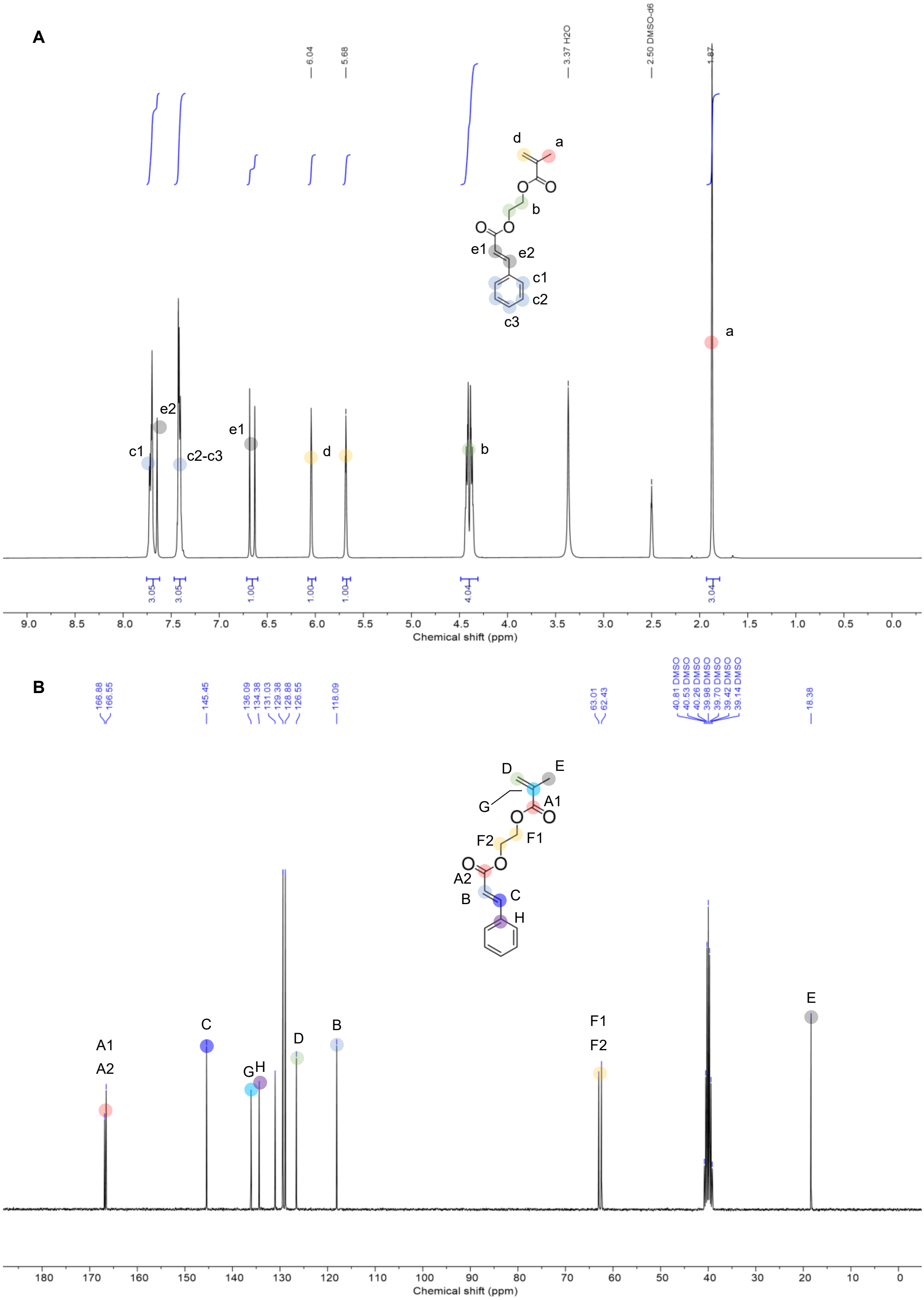

Supplementary Fig. 23: Structural characterization of MAc monomer. (**A**) ^{1}H NMR and (**B**) ^{13}C NMR spectrum in DMSO-d_6.

Supplementary Note 7 | NMR characterization of green-, red-, and blue-emitting self-hosted TADF polymers with different degree of polymerization (DP)

Please see **Supplementary Figs. 24, 25, and 26**.

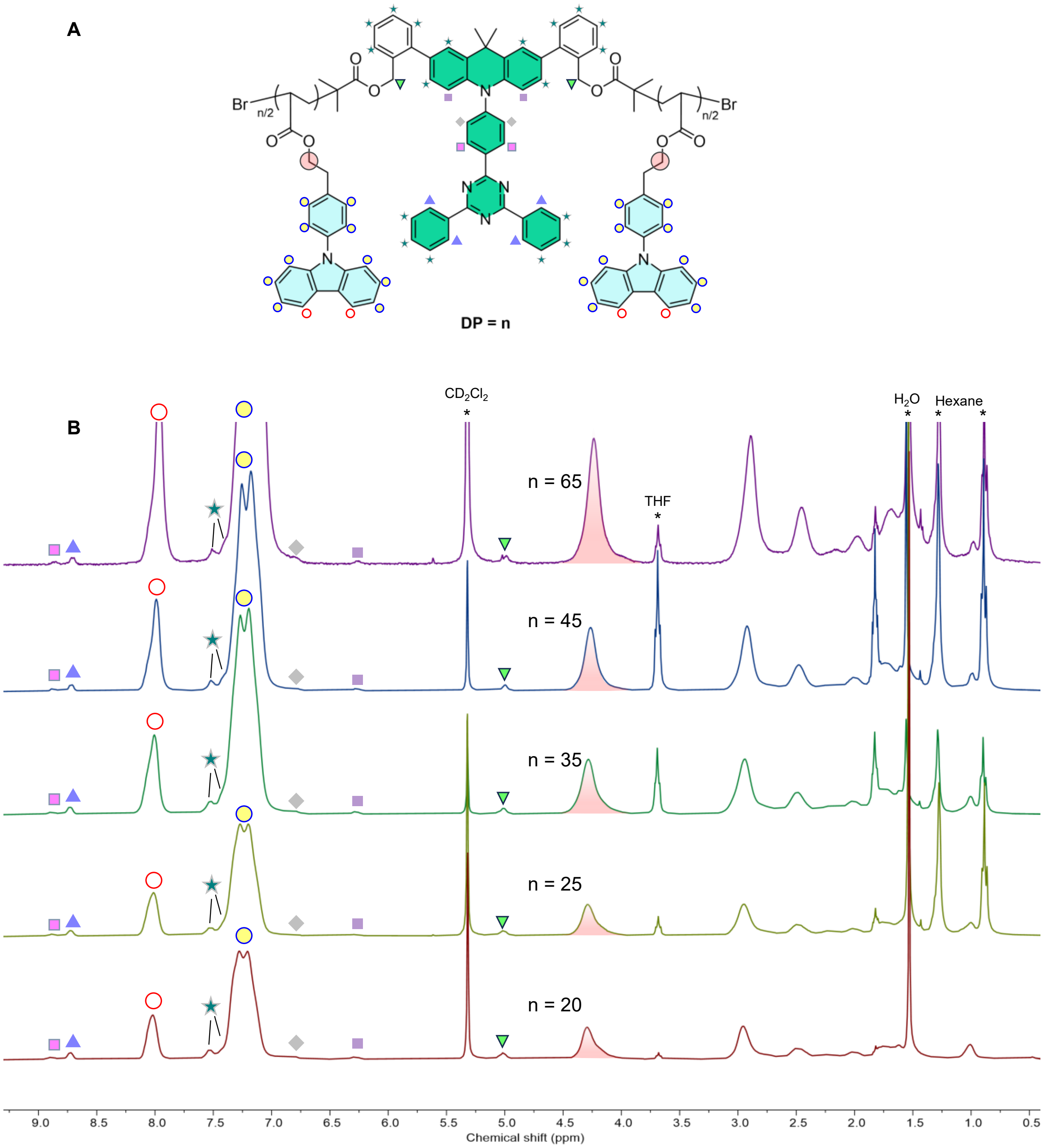

Supplementary Fig. 24: DP quantification of green-emitting self-hosted TADF polymers. (**A**) Chemical structure of the green-emitting self-hosted TADF polymers (**G-*ortho*-Ps**) with proton peaks marked for DP_{ACz} quantification (**B**) Stacked ^{1}H NMR spectra with different DP_{ACz} in CD_2Cl_2-d_2.

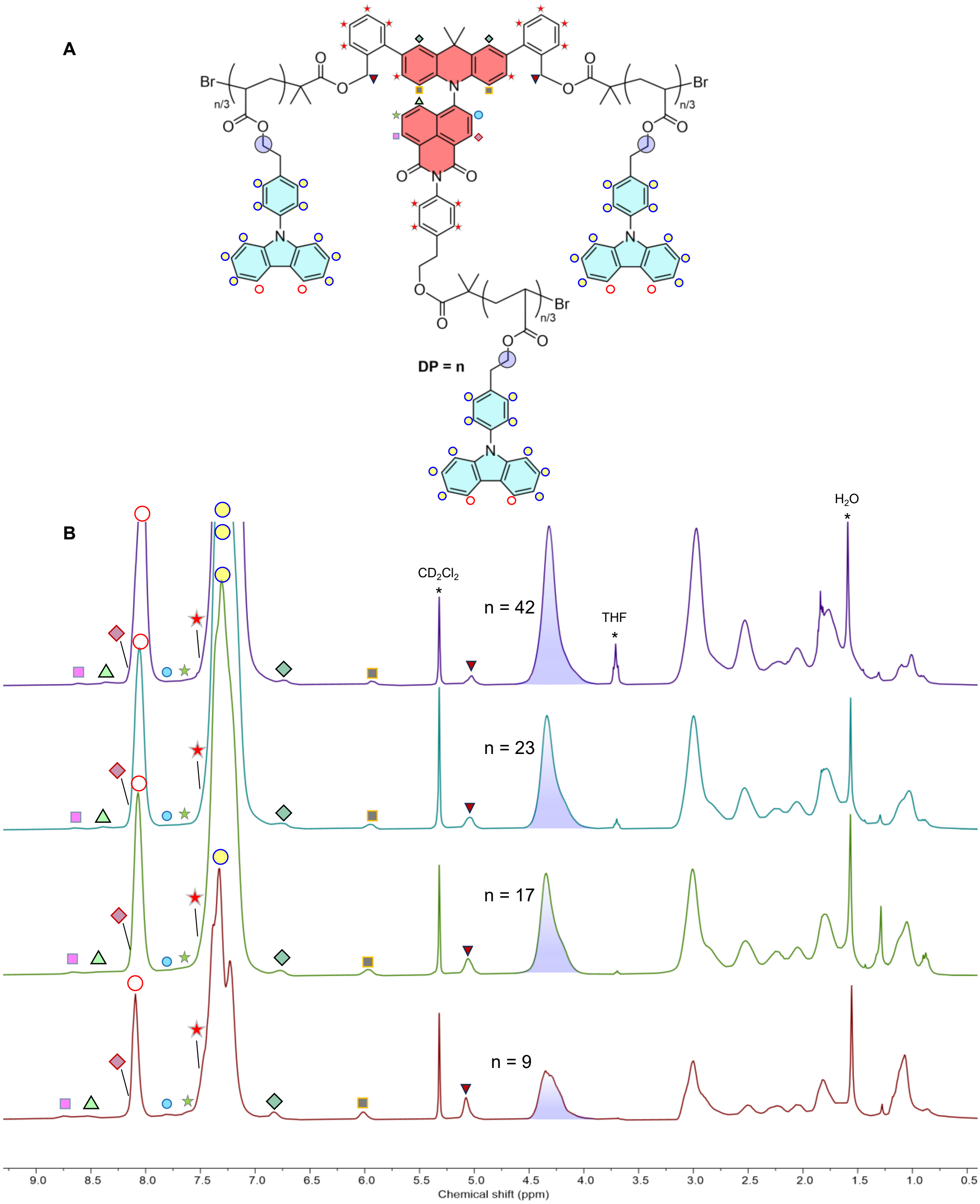

Supplementary Fig. 25: DP quantification of red-emitting self-hosted TADF polymers. (**A**) Chemical structure of the red-emitting self-hosted TADF polymers (**R-*ortho*-Ps**) with the proton peaks marked for DP_{ACz} quantification (**B**) Stacked ^{1}H NMR spectra with different DP_{ACz} in CD_2Cl_2-d_2.

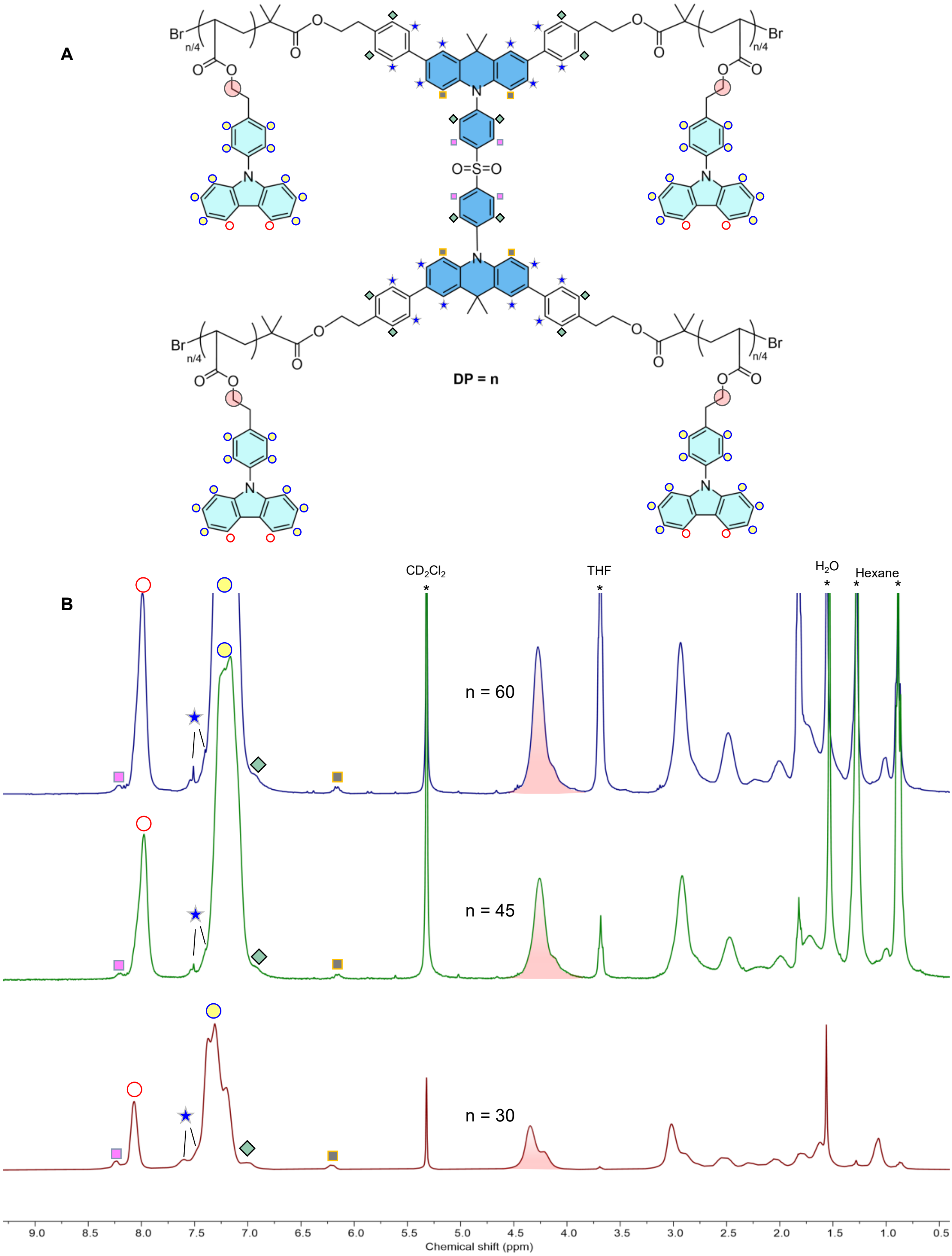

Supplementary Fig. 26: DP quantification of blue-emitting self-hosted TADF polymers. (**A**) Chemical structure of the blue-emitting self-hosted TADF polymers (**B-*para*-Ps**) with the proton peaks marked for DP_{ACz} quantification (**B**) Stacked ^1H NMR spectra with different DP_{ACz} in CD_2Cl_2-d_2.

Supplementary Note 8 | NMR characterization of the green-, red-, and blue-emitting self-hosted TADF polymers before and after end group de-activation

Please see **Supplementary Fig. 27, 28 , and 29.**

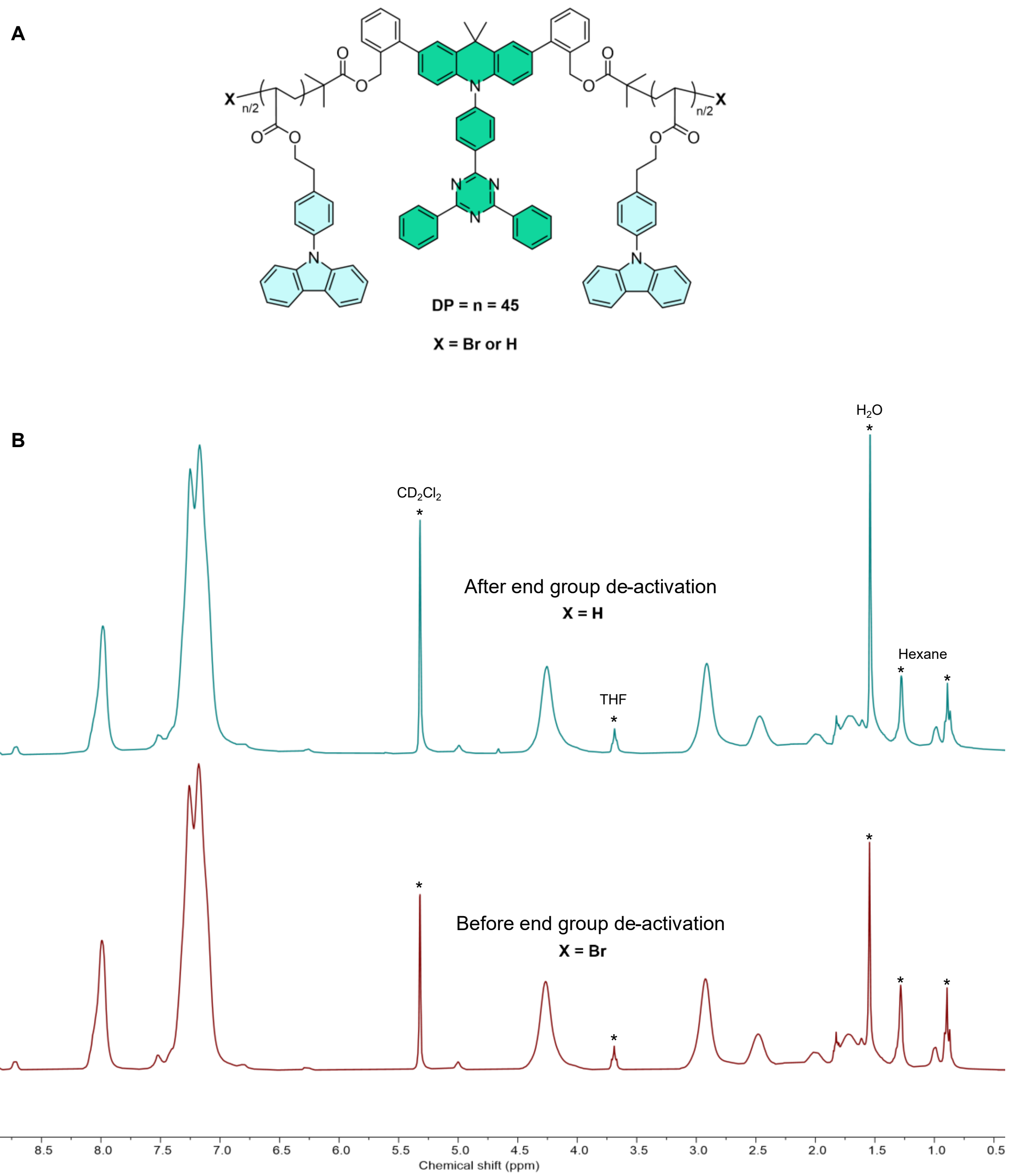

Supplementary Fig. 27: ^{1}H NMR spectra comparison of the selected green-emitting self-hosted TADF polymers before and after end group de-activation. (**A**) Chemical structure of the green-emitting self-hosted TADF polymers before (**G-*ortho*-P-Br**) and after (**G-*ortho*-P-deBr**) end group de-activation (**B**) Stacked ^{1}H NMR spectra in CD_2Cl_2-d_2.

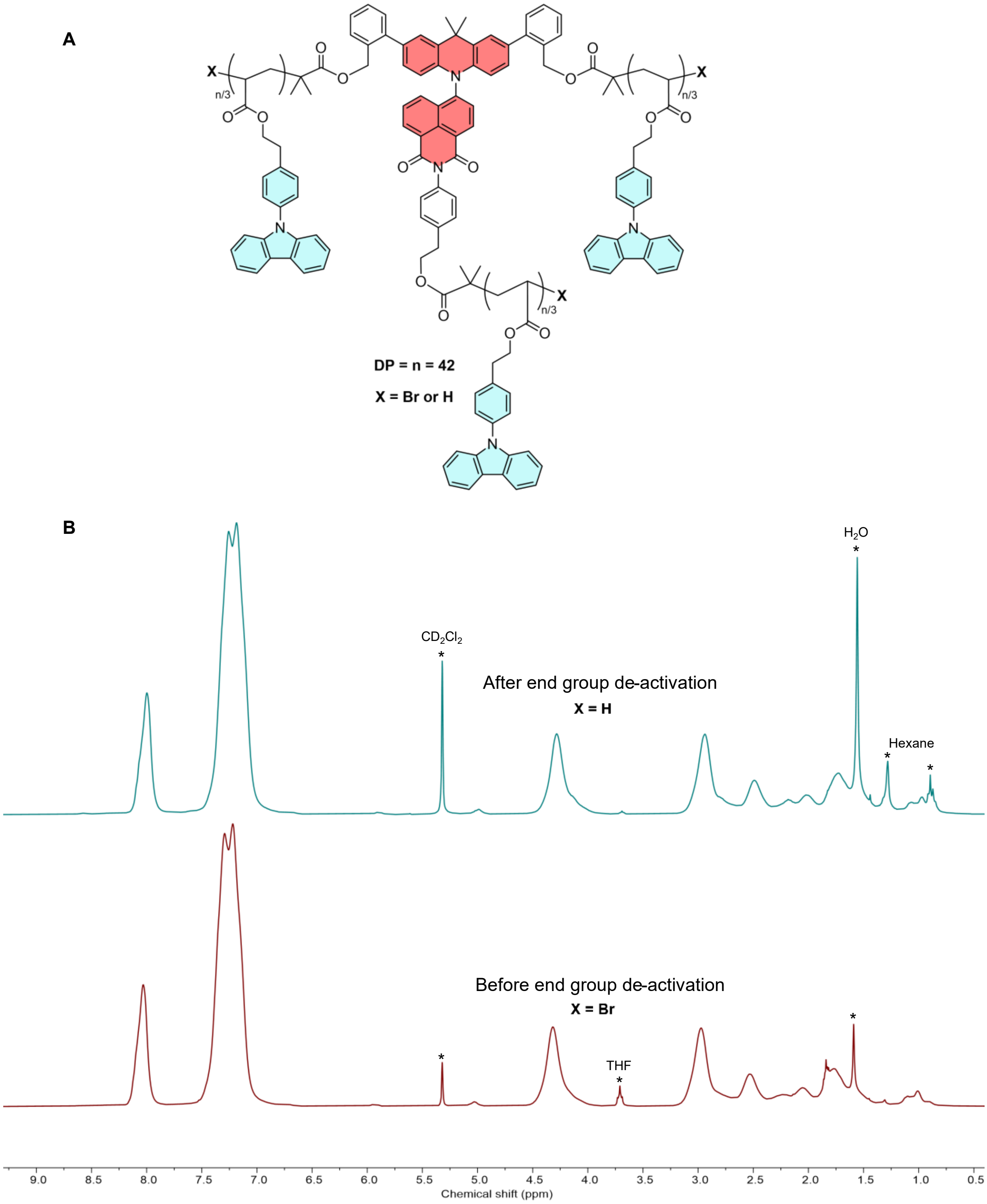

Supplementary Fig. 28: ^{1}H NMR spectra comparison of the selected red-emitting self-hosted TADF polymers before and after end group de-activation. (**A**) Chemical structure of the red-emitting self-hosted TADF polymers before (**R-*ortho*-P-Br**) and after (**R-*ortho*-P-deBr**) end group de-activation (**B**) Stacked ^{1}H NMR spectra in CD_2Cl_2-d_2.

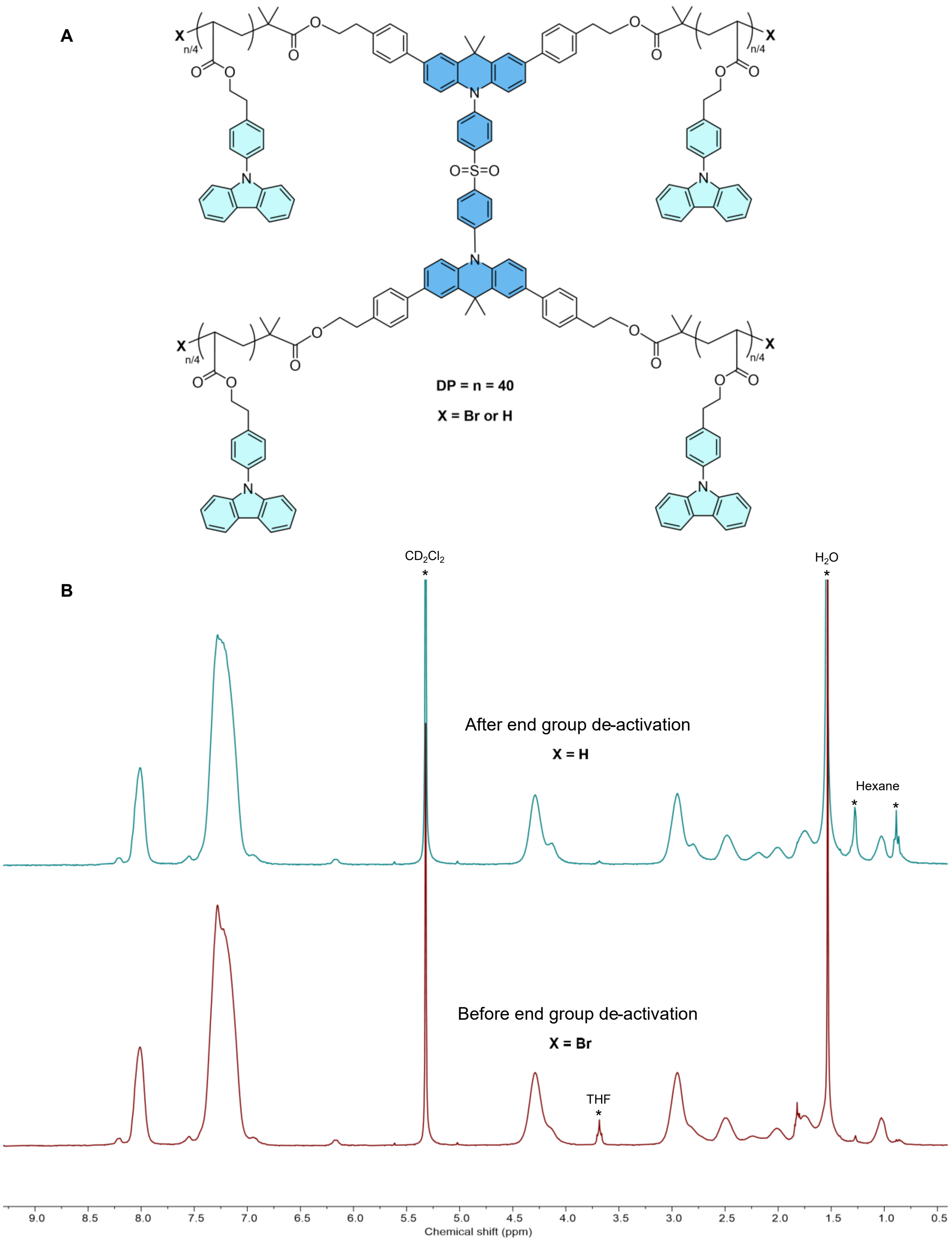

Supplementary Fig. 29: ^{1}H NMR spectra comparison of the selected blue-emitting self-hosted TADF polymers before and after end group de-activation. (**A**) Chemical structure of the blue-emitting self-hosted TADF polymers before (**B-*para*-P-Br**) and after (**B-*para*-P-deBr**) end group de-activation (**B**) Stacked ^{1}H NMR spectra in CD_2Cl_2-d_2.

Supplementary Note 9 | NMR characterization of the green-, red-, and blue-emitting ELPR polymers before and after end group de-activation

Please see **Supplementary Fig. 30, 31, and 32.**

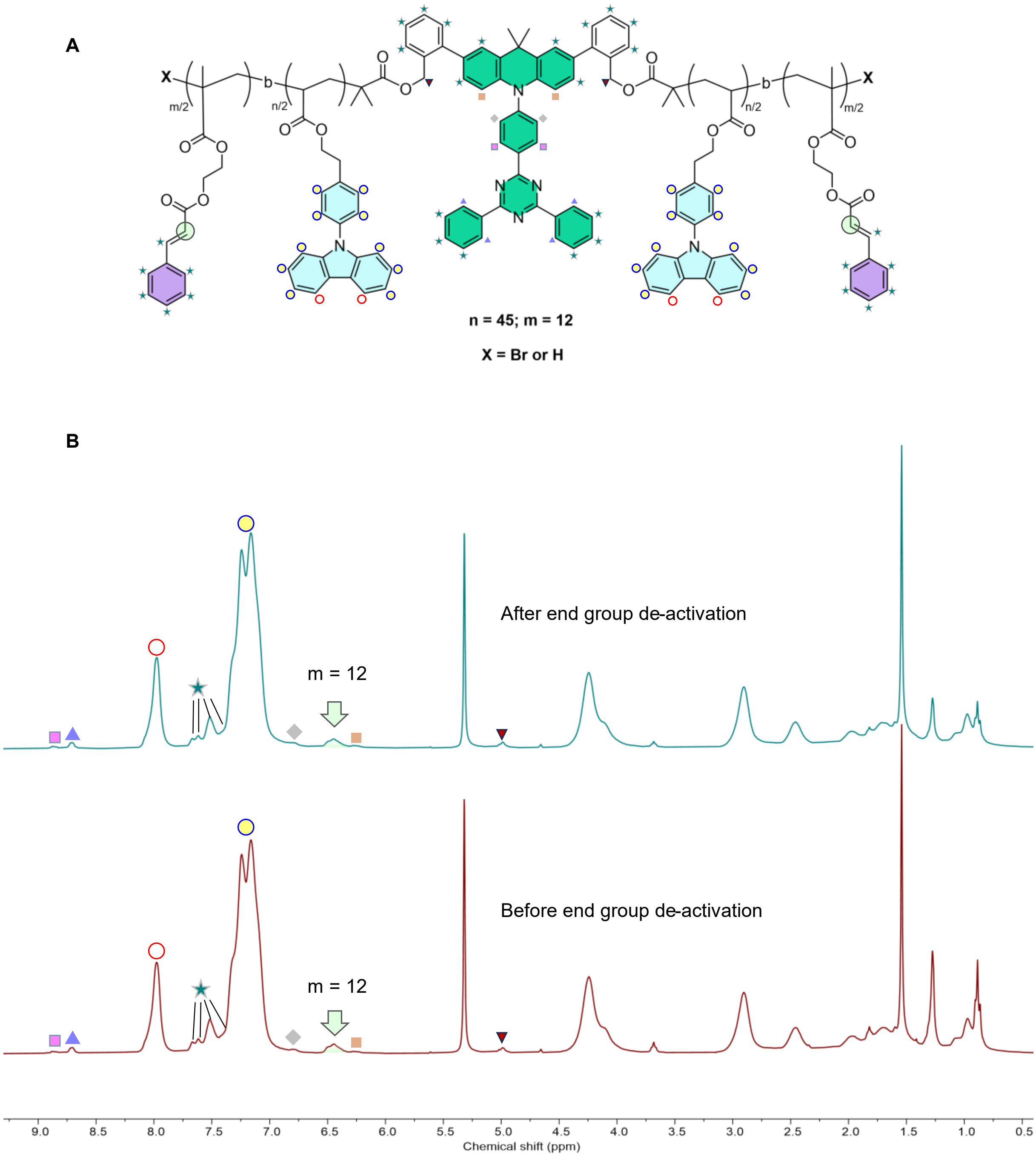

Supplementary Fig. 30: ^{1}H NMR spectra of the selected green-emitting ELPR polymers before/after end group de-activation. (**A**) Chemical structure of the selected green-emitting ELPR polymers before (**G-*ortho*-ELPR-Br**) and after (**G-*ortho*-ELPR-deBr**) end group de-activation with the proton peaks marked for DP_{MAc} quantification (**B**) Stacked ^{1}H NMR spectra in CD_2Cl_2-d_2.

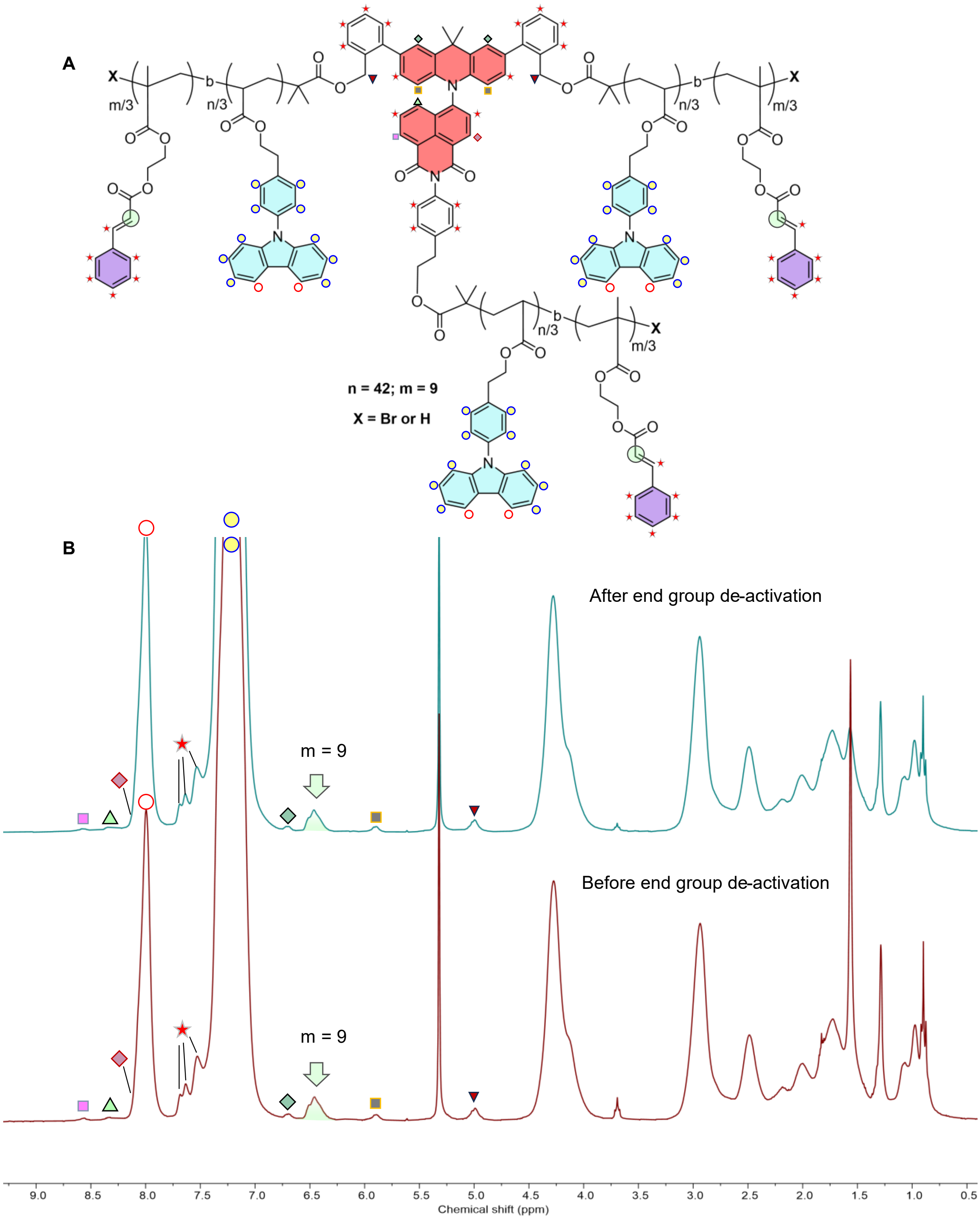

Supplementary Fig. 31: ^{1}H NMR spectra of the selected red-emitting ELPR polymers before and after end group de-activation. (**A**) Chemical structure of the selected red-emitting ELPR polymers before (**R-*ortho*-ELPR-Br**) and after (**R-*ortho*-ELPR-deBr**) end group de-activation with the proton peaks marked for DP_{MAc} quantification (**B**) Stacked ^{1}H NMR spectra in CD_2Cl_2-d_2.

A

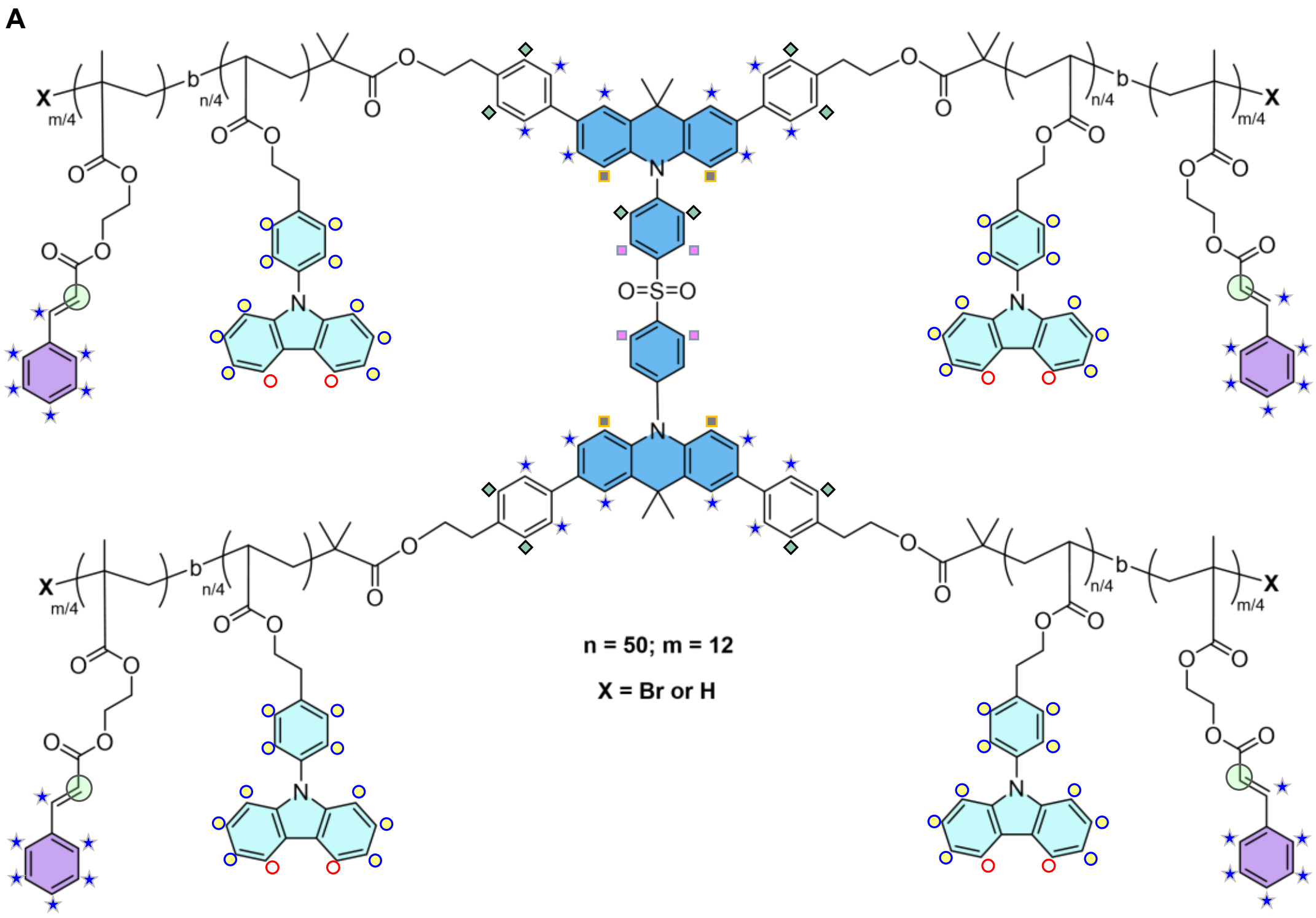

B

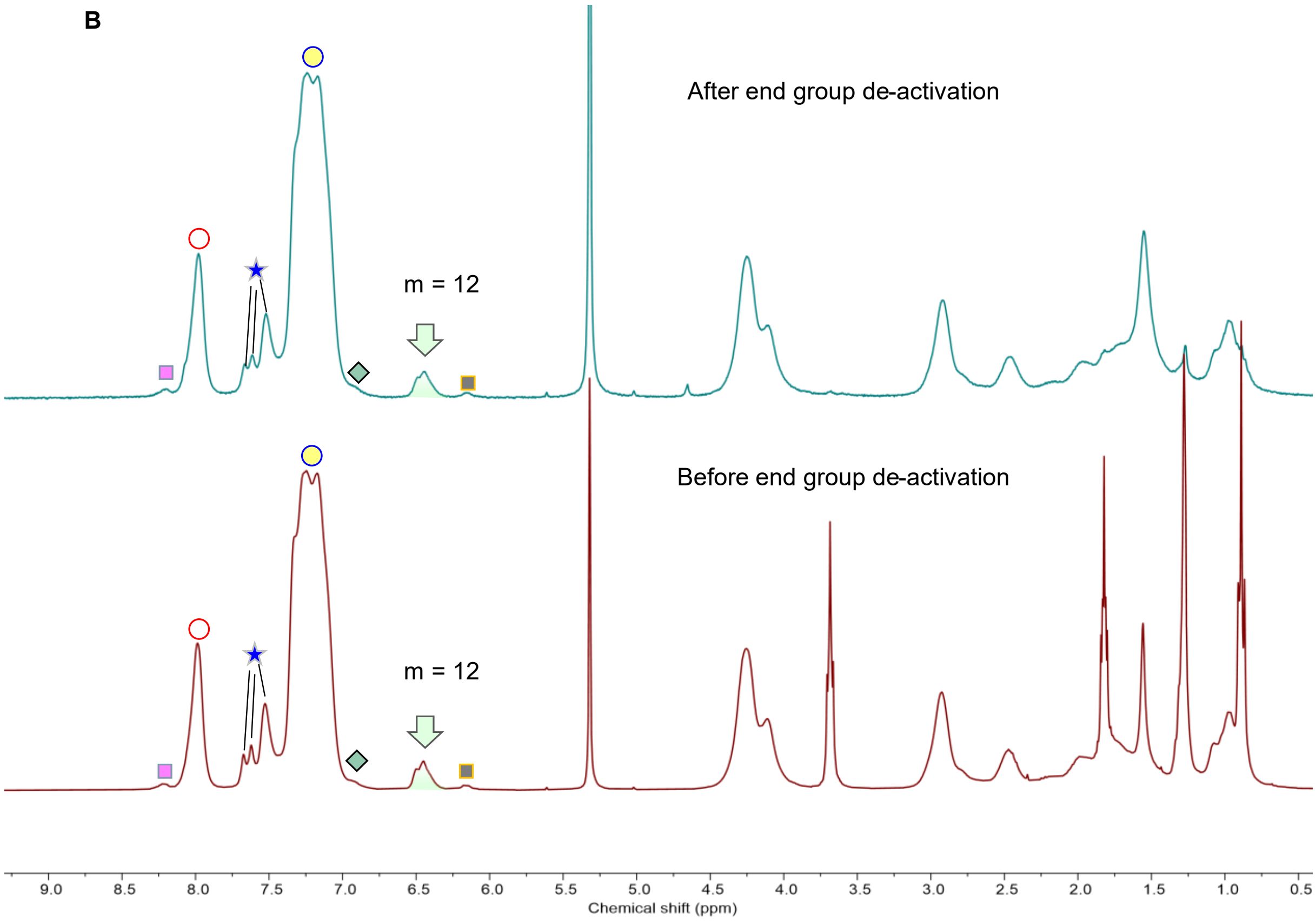

Supplementary Fig. 32: ^{1}H NMR spectra of the selected blue-emitting ELPR polymers before and after end group de-activation. (**A**) Chemical structure of the selected blue-emitting ELPR polymers before (**B-*para*-ELPR-Br**) and after (**B-*para*-ELPR-deBr**) end group de-activation with the proton peaks marked for DP_{MAc} quantification (**B**) Stacked ^{1}H NMR spectra in CD_2Cl_2-d_2.

Supplementary Note 10 | NMR characterization of the green-, red-, and blue-emitting E-beam patternable polymers before and after end group de-activation

Please see **Supplementary Fig.33, 35, and 35.**

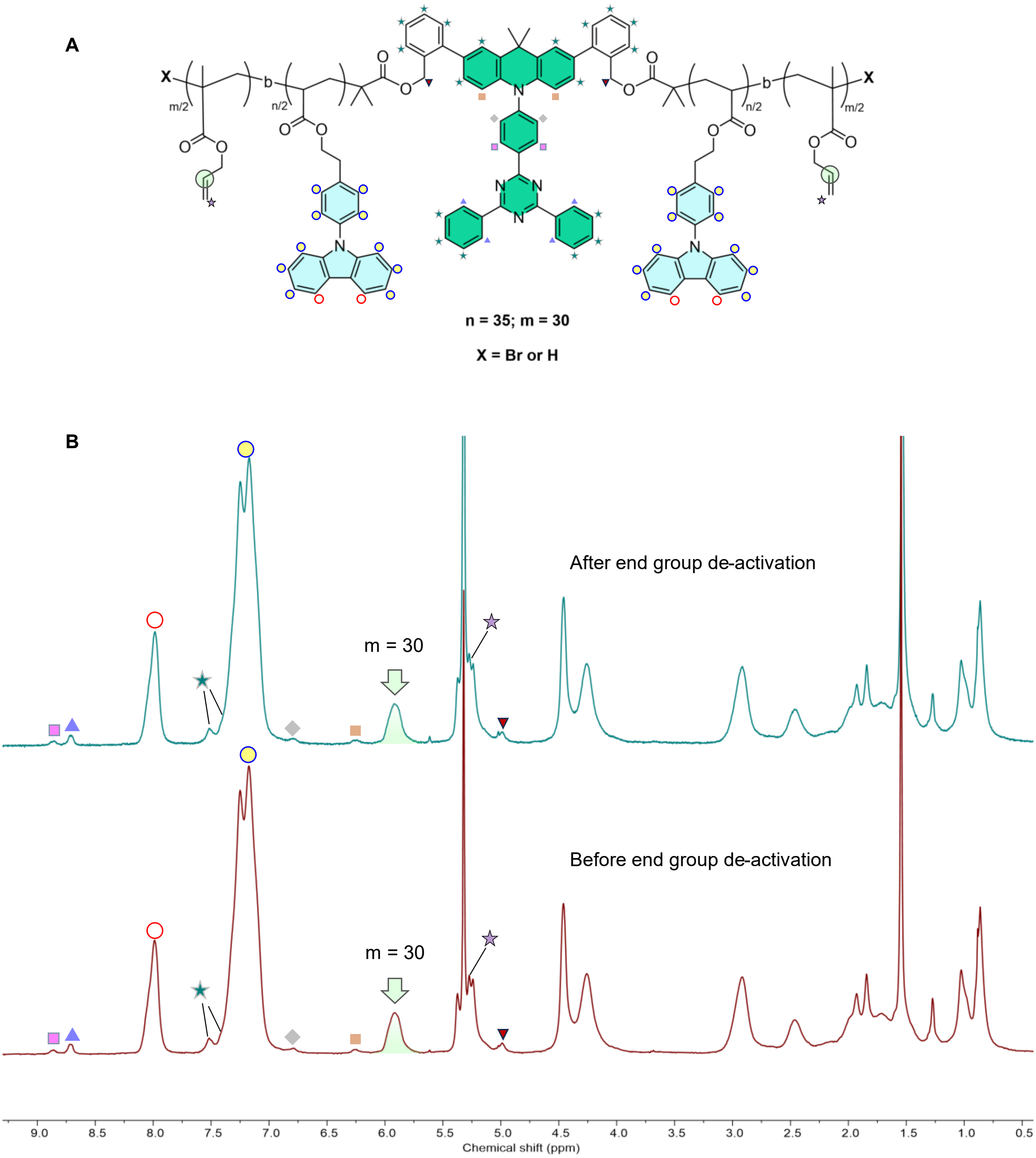

Supplementary Fig. 33: ¹H NMR spectra of the selected green-emitting E-beam patternable TADF polymers before and after end group de-activation. (**A**) Chemical structure of the selected green-emitting E-beam patternable polymers before (**G-*ortho*-ELPR-Br**) and after (**G-*ortho*-ELPR-deBr**) end group de-activation with the proton peaks marked for DP_{MAI} quantification (**B**) Stacked ^{1}H NMR spectra in CD_2Cl_2-d_2.

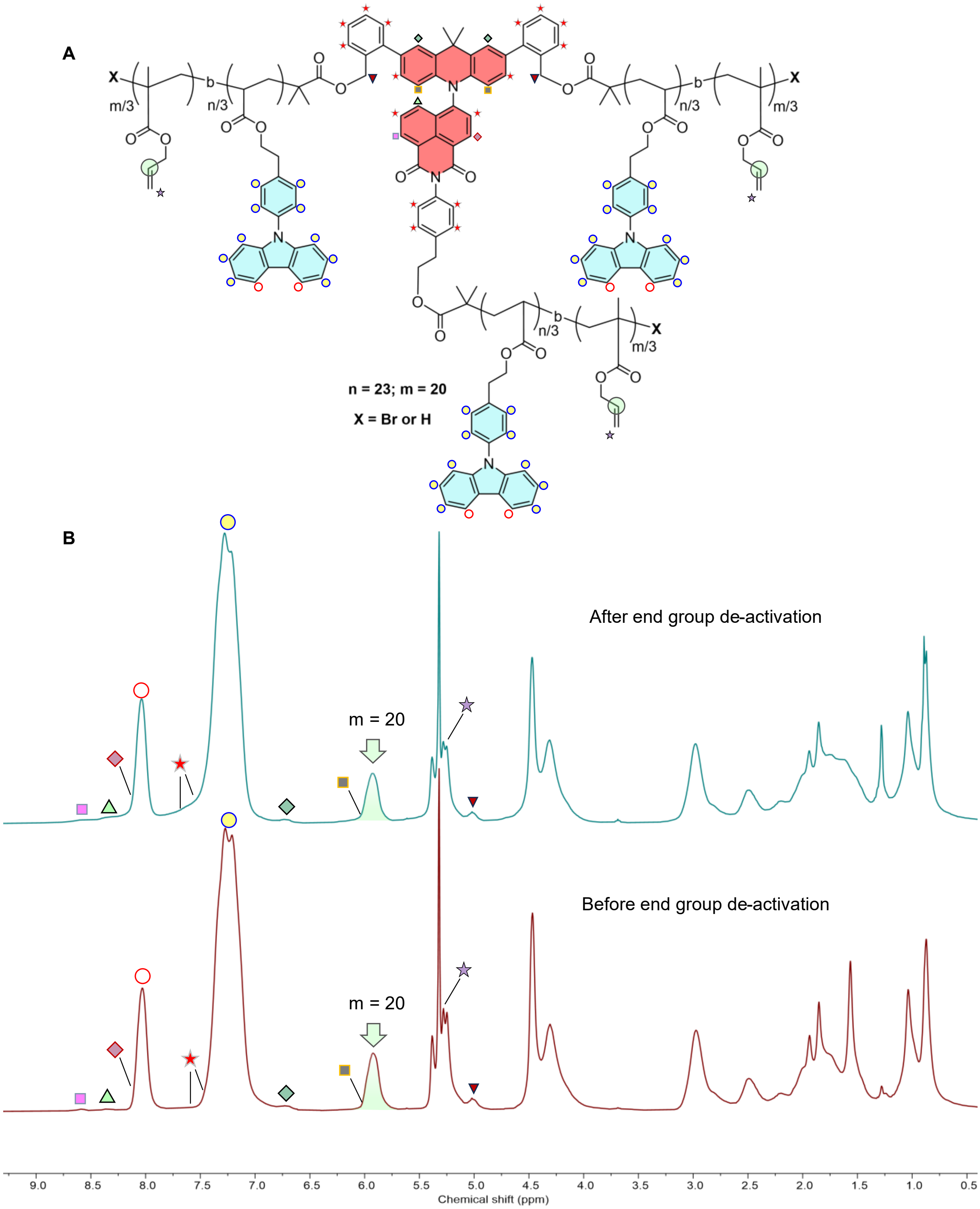

Supplementary Fig. 34: ^{1}H NMR spectra of the selected red-emitting E-beam patternable TADF polymers before and after end group de-activation. (**A**) Chemical structure of the selected red-emitting E-beam patternable polymers before (**R-*ortho*-ELPR**) and after (**R-*ortho*-ELPR**) end group de-activation with the proton peaks marked for DP_{MAI} quantification (**B**) Stacked ^{1}H NMR spectra in CD_2Cl_2-d_2.

A

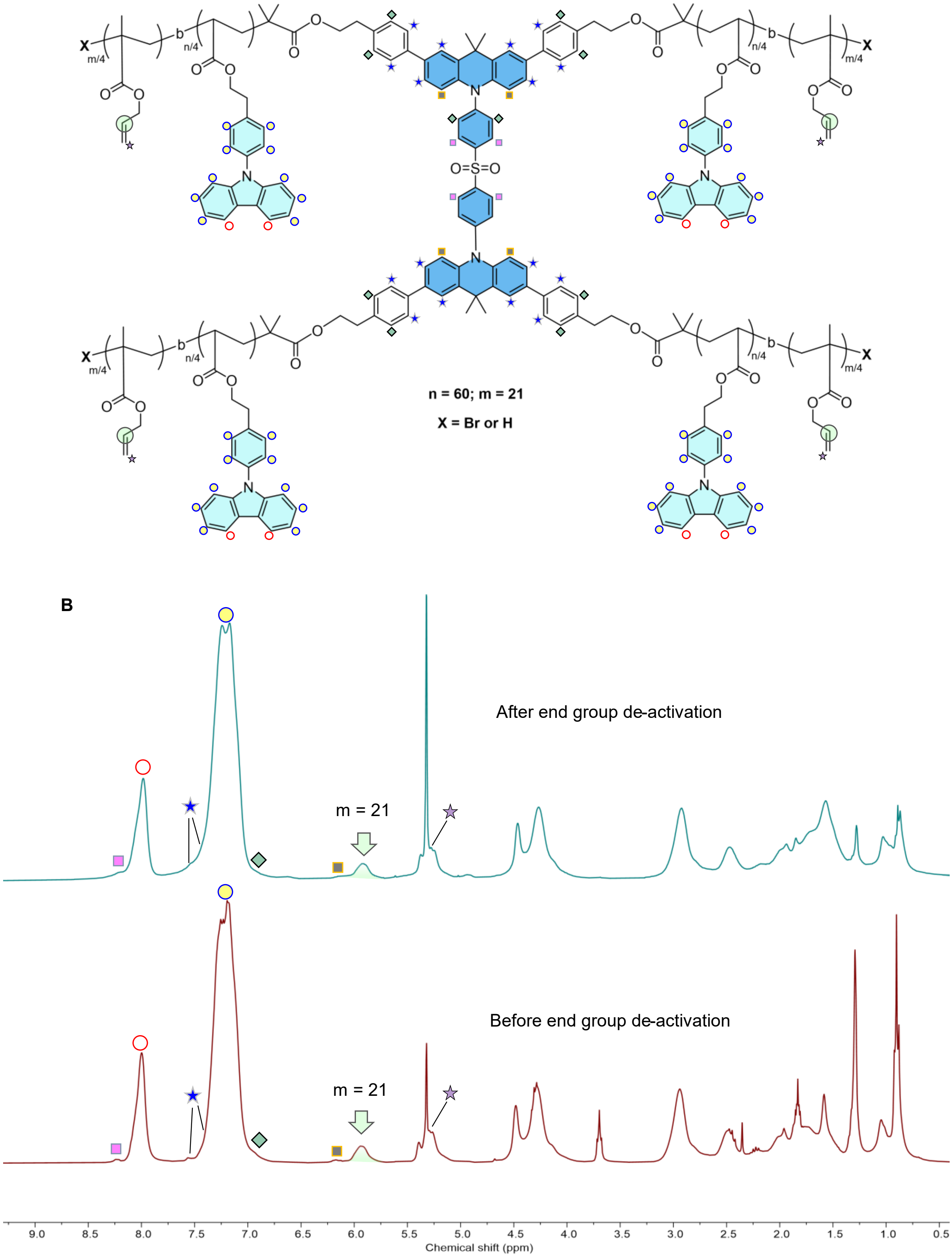

Supplementary Fig. 35: ^{1}H NMR spectra of the selected blue-emitting E-beam patternable TADF polymers before/after end group de-activation. (**A**) Chemical structure of the selected blue-emitting E-beam patternable polymers before (**B-*para*-ELPR-Br**) and after (**B-*para*-ELPR-deBr**) end group de-activation with the proton peaks marked for DP_{MAI} quantification (**B**) Stacked ^{1}H NMR spectra in CD_2Cl_2-d_2.

Supplementary Note 11 | Host monomer engineering for self-hosted G-*ortho*-Ps

Please see **Supplementary Fig. 36.**

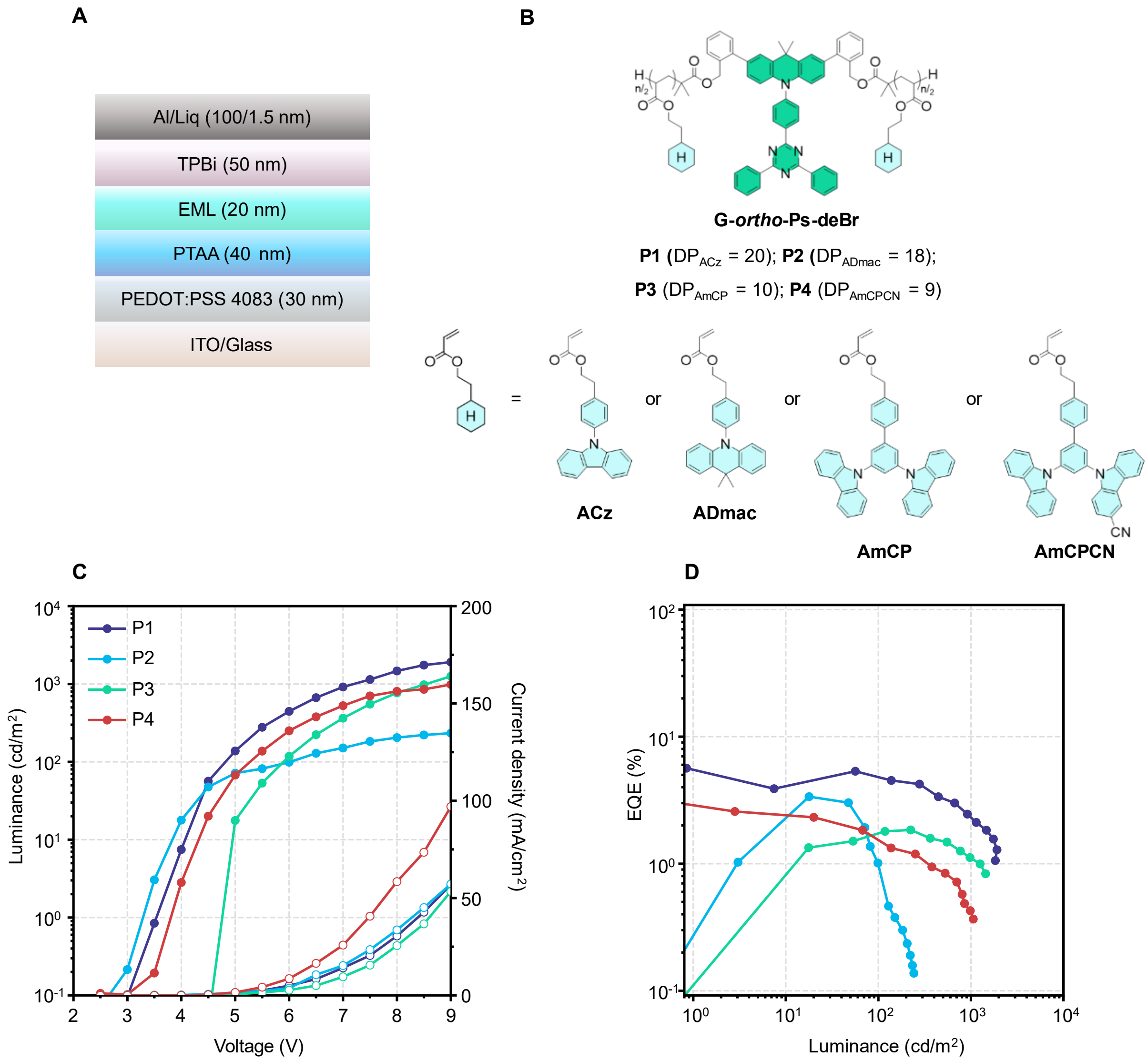

Supplementary Fig. 36: Host monomer engineering for G-*ortho*-Ps synthesized with host monomers bearing different aromatic moieties and their OLED performance. (**A**) OLED device architecture where **G-*ortho*-P** was used as EML. (**B**) Chemical structure of G-*ortho*-Ps based on 4 different host monomers, where the weight doping ratio of emitter-to-host within **G-*ortho*-Ps** was tuned to be roughly the same (13~15 wt%). (**C**) Luminance and current density versus voltage curves of 4 self-hosted polymers. (**D**) EQE versus luminance curves. The **P1** (**G-*ortho*-P** decorated with pendent ACz monomers) shows the highest performance in OLED. All the polymers were debrominated for OLED fabrication. Note the device structure is different from the set-up which is optimized in a later stage, so the EQE values are much lower.

Supplementary Table 1 | Summary of OLED performance for host engineering

	Host monomer (DP[a])	Doping ratio[b] (wt. %)	$EQE_{max., 100 cd m^{-2}}$ [c] (%)	Current density$_{max., 100 cd m^{-2}}$ [d] ($mA m^{-2}$)	Luminance$_{max.}$ ($cd m^{-2}$)
**P1**	ACz (DP_{ACz} = 20)	13	5.3, 4.9	166.2, 0.8	2590
**P2**	ADmac (DP_{ACz} = 18)	13	3.4, 0.9	131.5, 4.0	297
**P3**	AmCP (DP_{AmCP} = 10)	15	1.8, 1.7	184.9, 2.4	1919
**P4**	AmCPCN (DP_{AmCPCN} = 9)	15	1.8, 1.6	200.7, 2.8	1074

a. DP was determined by ^{1}H-NMR. *b*. Doping ratio is calculated from the weight percentage ratio of the emitting initiator to the installed host monomers. *c*. max. EQE and EQE at 100 cd m^{-2}. *d*. Max. current density and current density at 100 cd m^{-2}. Note the device structure is different from the set -up which is optimized in a later stage, so the EQEs are much lower.

Supplementary Note 12 | Size-Exclusion Chromatography (SEC) curves of selected TADF polymers

Please see **Supplementary Fig. 37.**

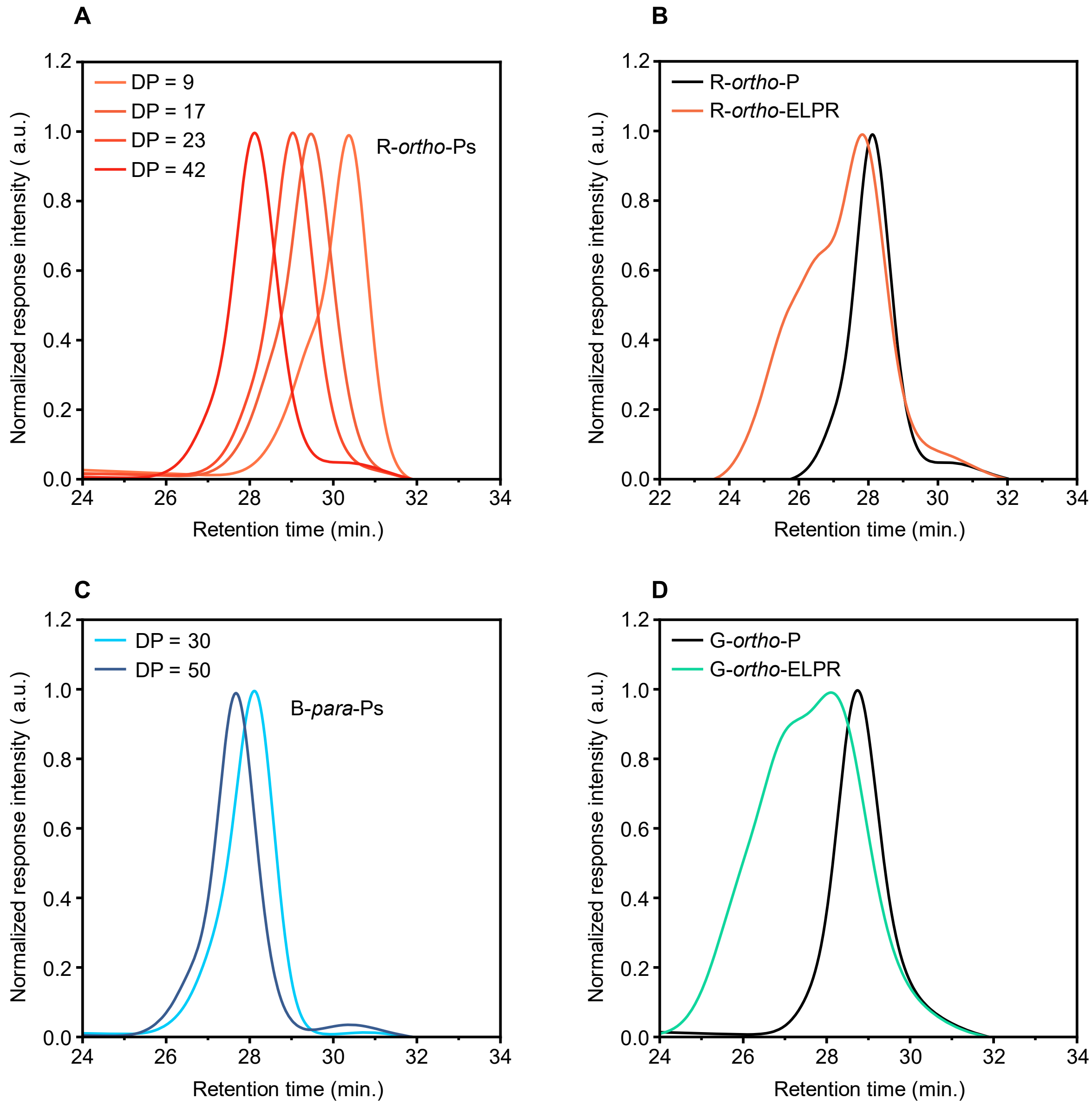

Supplementary Fig. 37: SEC curves of selected self-hosted TADF polymers and ELPRs. (**A**) SEC spectra of **R-*ortho*-Ps** with different DP_{Acz} (**B**) SEC spectra of **R-*ortho*-P** (DP_{ACz} = 42) and **R-*ortho*-ELPR** (DP_{ACz} = 42, DP_{MAc} = 9, before end group de-activation) (**C**) SEC spectra of **B-*para*-Ps** with different DP_{Acz} (**D**) SEC spectra of **G-*ortho*-P** (DP_{ACz} = 45) and **G-*ortho*-ELPR** (DP_{ACz} = 45, DP_{MAc} = 12)

Supplementary Table 2 | SEC data of selected TADF polymers.

Polymers	End group de-activation?	DP[a]	Doping ratio[b] (wt. %)	$M_{n,\ NMR}$[a] *(g mol⁻¹)*	$M_{n,\ SEC}$[c] *(g mol⁻¹)*	$M_{w,\ SEC}$[c] *(g mol⁻¹)*	*Đ*[c]
**G-*ortho*-P**	N	DP_{ACz} = 20	15.0	7855	4907	5721	1.16
	N	DP_{ACz} = 25	12.0	9562	5058	6102	1.21
	N	DP_{ACz} = 35	8.6	12976	6980	8387	1.20
	N	DP_{ACz} = 45	6.7	16390	8175	10228	1.25
	N	DP_{ACz} = 65	4.6	23218	11943	16519	1.24
**G-*ortho*-ELPR**	N	DP_{ACz} = 45, DP_{MAc} = 12	6.7	19513	13603	22422	1.65
	Y	DP_{ACz} = 45, DP_{MAc} = 12	6.7	19356	13389	22319	1.67
**R-*ortho*-P**	N	DP_{ACz} = 9	38.5	4256	3512	4176	1.19
	N	DP_{ACz} = 17	20.4	6987	5125	6385	1.24
	N	DP_{ACz} = 23	15.1	9036	6522	7779	1.19
	N	DP_{ACz} = 42	8.3	15515	9407	12387	1.22
**R-*ortho*-ELPR**	N	DP_{ACz} = 42, DP_{MAc} = 9	8.3	17739	12916	23248	1.80
	Y	DP_{ACz} = 42, DP_{MAc} = 9	8.3	17502	11679	22430	1.92
**B-*para*-P**	N	30	16.7	11955	11830	13174	1.11
	N	50	10.0	18783	14295	15636	1.09

a. DP and $M_{n,\ NMR}$ are determined by H-NMR. *b*. Doping ratio is calculated from the weight percentage ratio of the emitting initiator to the installed host monomers. *c*. $M_{n,\ SEC}$, $M_{w,\ SEC}$, and polydispersity index (*Đ)* are determined by SEC measurement with poly(methyl methacrylate) standards.

Supplementary Note 13 | Lifetime decay curves

Please see **Supplementary Figs.38-43 and Table 3-5.**

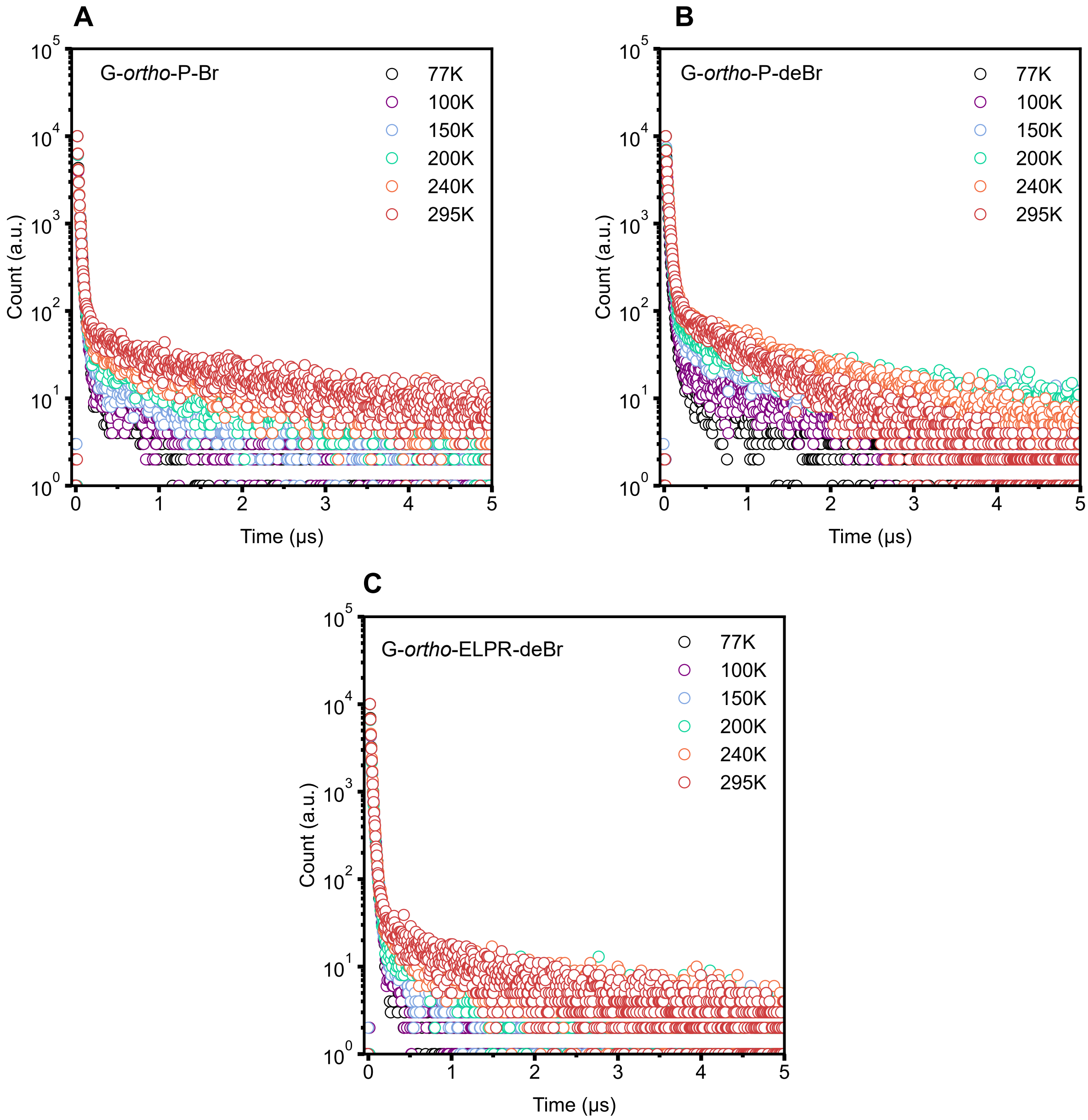

Supplementary Fig. 38: TRPL spectra of green-emitting TADF polymers coated on quartz substrates measured at various temperature from 77 to 295 K. (**A**) **G-*ortho*-P-Br** (DP_{ACz} = 45, before end-group de-activation) (**B**) **G-*ortho*-P-deBr** (DP_{ACz} = 45, after end-group de-activation) (**C**) **G-*ortho*-ELPR-deBr** (DP_{ACz} = 45, DP_{MAc} = 12)

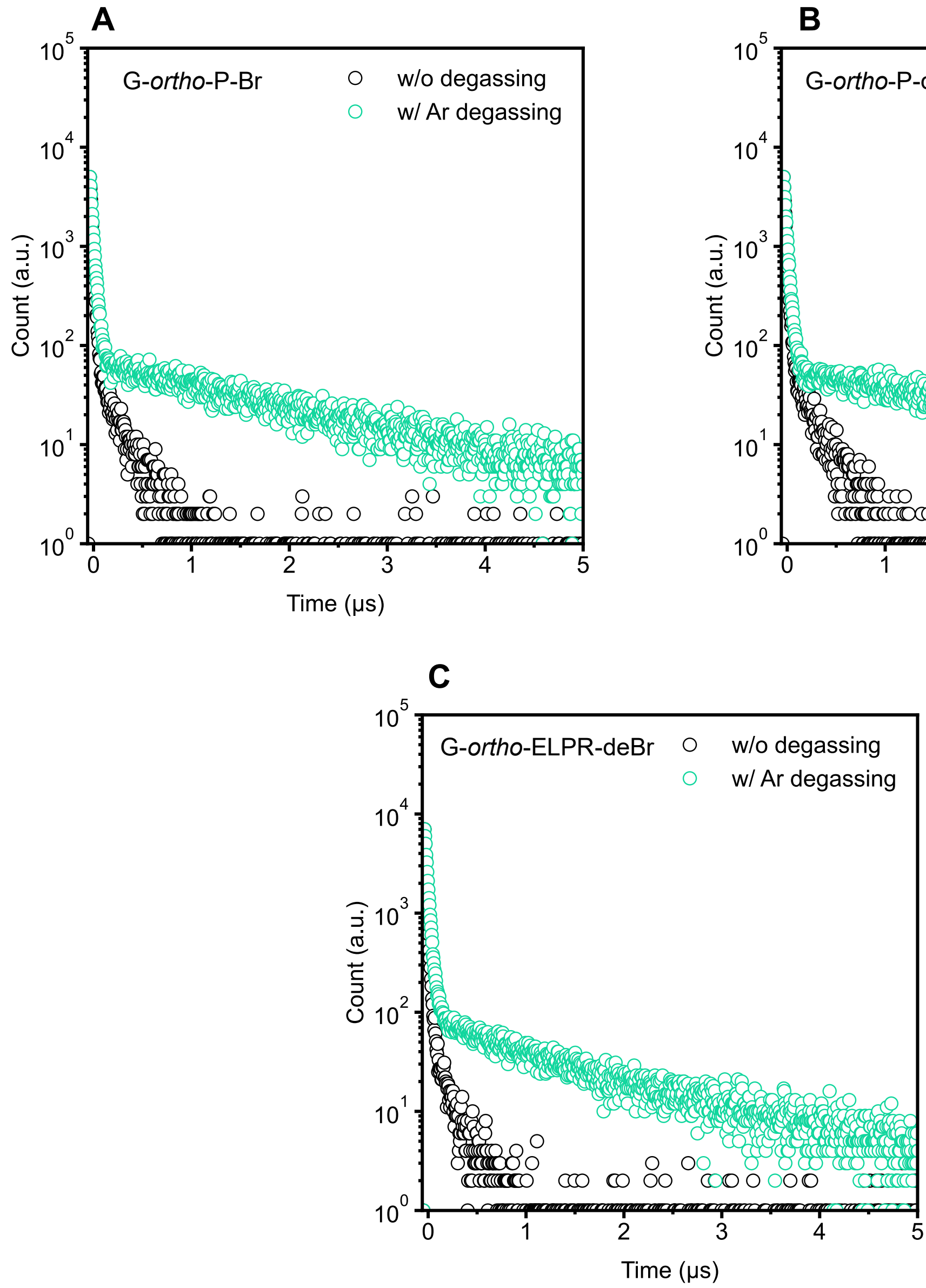

Supplementary Fig. 39: TRPL spectra of green-emitting TADF polymers dissolved in toluene measured at room temperature w/o or w/ argon degassing. (**A**) **G-*ortho*-P-Br** (DP_{ACz} = 45, before end-group de-activation) (**B**) **G-*ortho*-P-deBr** (DP_{ACz} = 45) (**C**) **G-*ortho*-ELPR-deBr** (DP_{ACz} = 45, DP_{MAc} = 12)

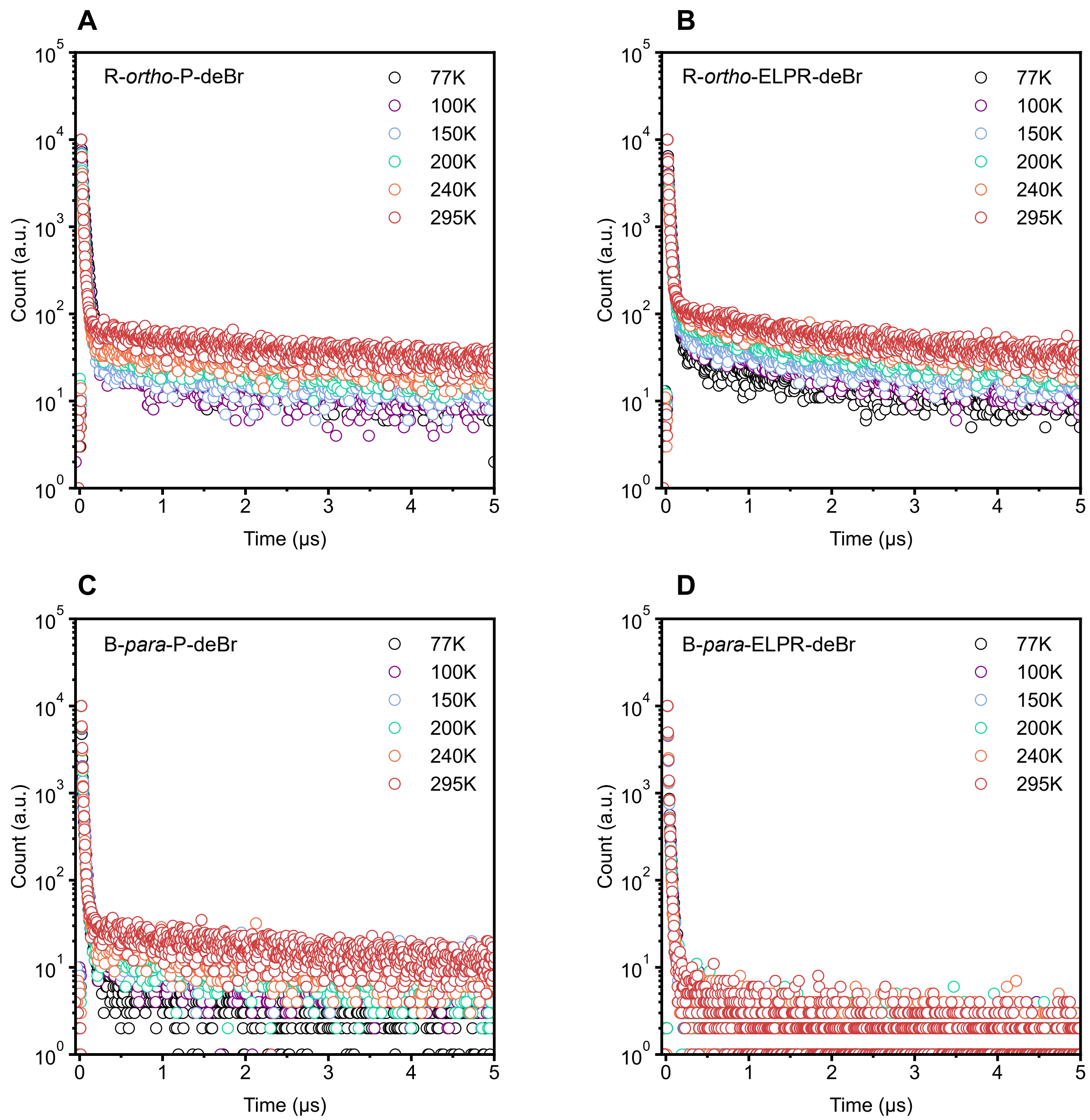

Supplementary Fig. 40: TRPL spectra of red- and blue-emitting polymers coated on quartz substrates measured at various temperature from 77 to 295 K. (**A**) **R-*ortho*-P-deBr** (DP_{ACz} = 42) (**B**) **R-*ortho*-ELPR-deBr** (DP_{ACz} = 42; DP_{MAc} = 9) (**C**) **B-*para*-P-deBr** (DP_{ACz} = 50) (**D**) **B-*para*-ELPR-deBr** (DP_{ACz} = 50; DP_{MAc} = 7,)

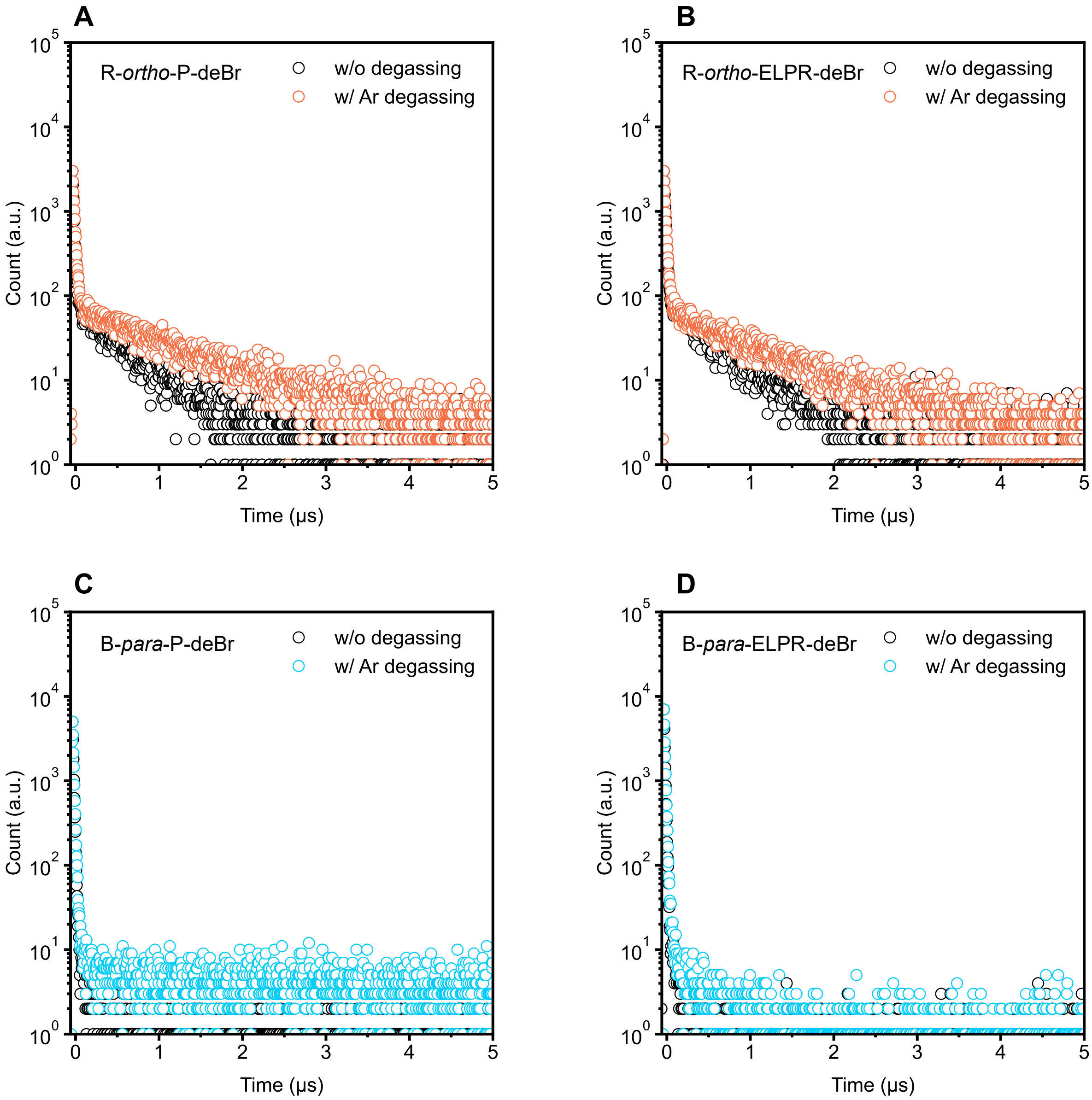

Supplementary Fig. 41: TRPL spectra of red- and blue-emitting TADF polymers dissolved in toluene measured at room temperature w/o or w/ argon degassing. (**A**) **R-*ortho*-P-deBr** (DP_{ACz} = 42, after end-group de-activation) (**B**) **R-*ortho*-ELPR-deBr** (DP_{ACz} = 42; DP_{MAc} = 9, after end group de-activation) (**C**) **B-*para*-P-deBr** (DP_{ACz} = 50, after end group de-activation) (**D**) **B-*para*-ELPR-deBr** (DP_{ACz} = 50; DP_{MAc} = 7, after end group de-activation)

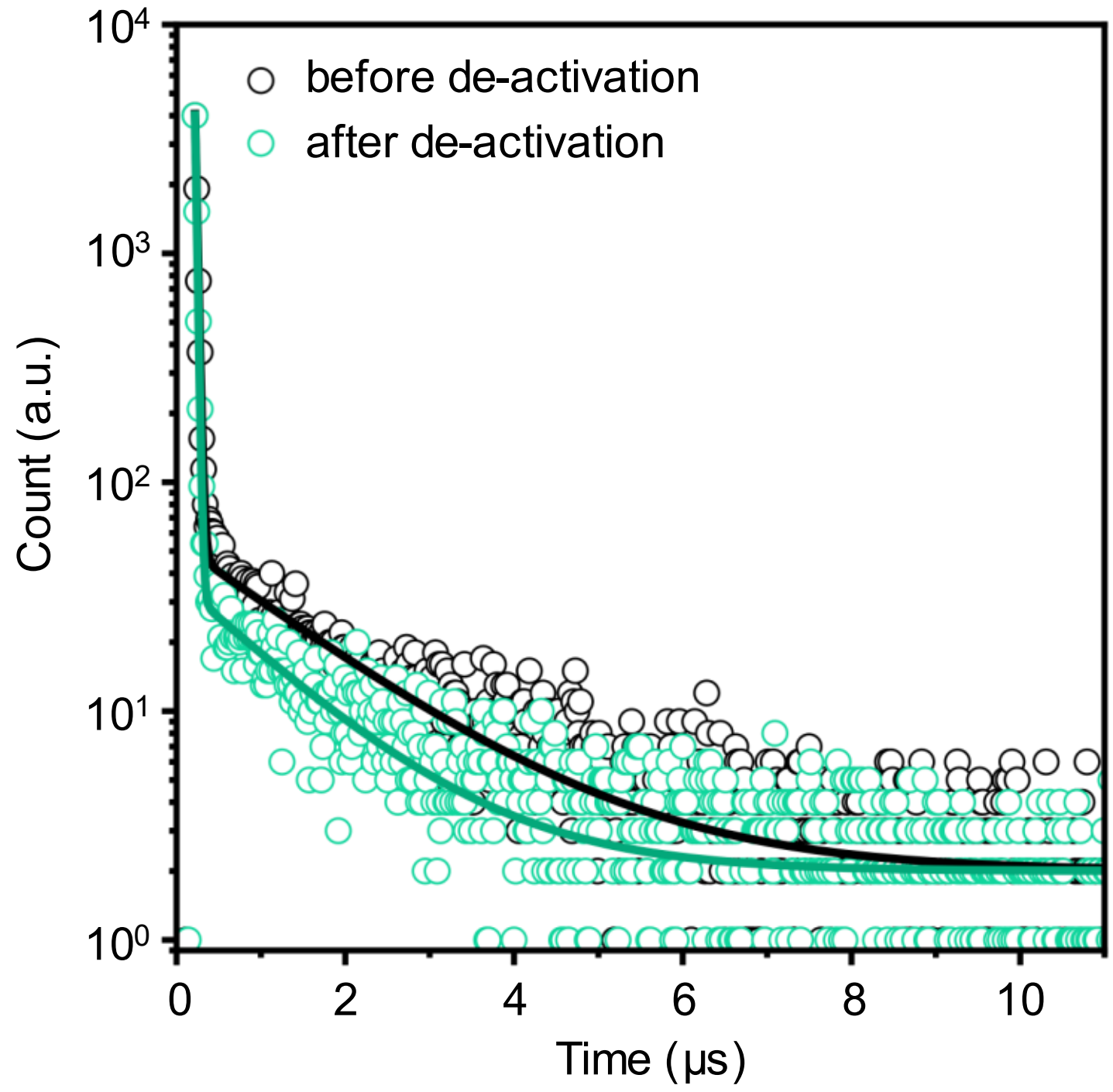

Supplementary Fig. 42: Thin film TRPL spectra of green-emitting self-hosted TADF polymer before (G-*ortho*-P-Br) and after (G-*ortho*-P-deBr) end group de-activation with DP_{ACz} = 45 measured at room temperature.

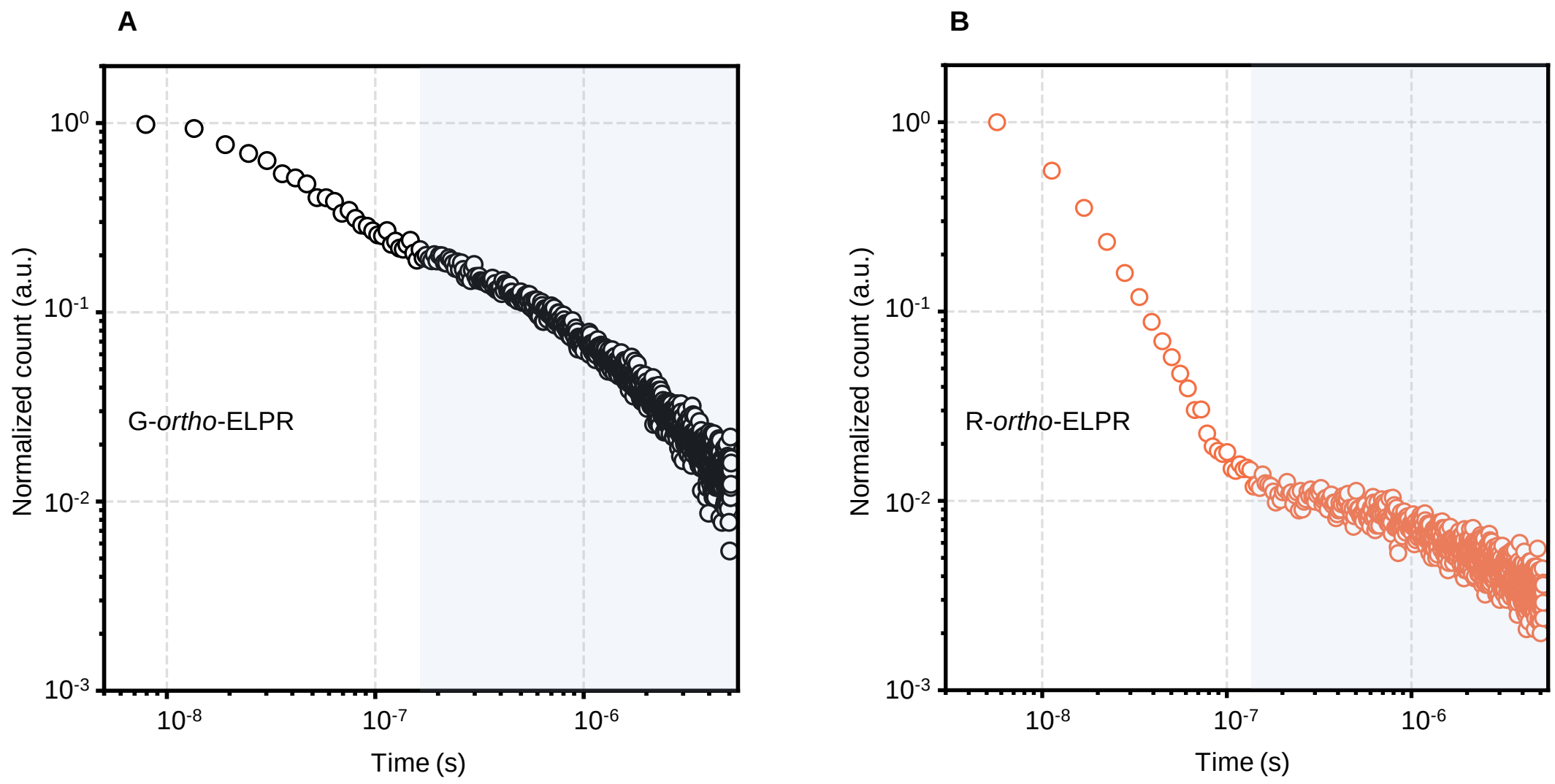

Supplementary Fig. 43: Logarithmic scale thin film TRPL spectra of G-*ortho*-ELPR (A, DP_{ACz} = 45, DP_{MAc} = 12) and R-*ortho*-ELPR (B, DP_{ACz} = 42, DP_{MAc} = 9) measured at room temperature.

Supplementary Table 3 | Exciton lifetimes of selected TADF polymers in toluene solution with/without argon degassing

Polymers (DP[a])	End group de-activation?	Argon degassing?	χ^{b} (-)	τ_{avg}^{c} (μs)	τ_{PF}^{d} (ns)	τ_{DF}^{e} (μs)
**G-*ortho*-P** (DP_{ACz} = 45)	N	N	0.979	0.029	19.1	0.181
	N	Y	1.060	0.756	27.4	1.840
	Y	N	1.142	0.392	20.4	0.184
	Y	Y	1.020	1.020	27.1	2.111
**G-*ortho*-ELPR** (DP_{ACz} = 45, DP_{MAc} = 12)	N	N	1.349	0.025	19.1	0.115
	N	Y	1.000	0.152	26.7	0.736
	Y	N	1.024	0.022	17.8	0.115
	Y	Y	1.103	0.301	27.6	1.196
**R-*ortho*-P** (DP_{ACz} = 42)	Y	N	1.179	0.143	15.8	0.535
	Y	Y	1.184	0.574	20.1	1.088
**R-*ortho*-ELPR** (DP_{ACz} = 42, DP_{MAc} = 9)	Y	N	1.117	0.147	16.8	0.532
	Y	Y	1.044	0.453	19.7	0.932
**B-*para*-P** (DP_{ACz} = 50)	Y	N	0.997	0.011	10.6	0.068
	Y	Y	1.035	0.016	13.2	0.169
**B-*para*-ELPR** (DP_{ACz} = 50, DP_{MAc} = 7)	Y	N	1.001	0.011	10.5	0.042
	Y	Y	1.007	0.013	12.4	0.057

a. DP was determined by ^{1}H-NMR. *b*. Fitting parameter χ for bi-exponential function fitting. *c*. Average lifetime. *d*. Prompt fluorescence lifetime. *e*. Delayed fluorescence lifetime. All the lifetimes (τ_{avg}, τ_{PF}, and τ_{DF}) are determined from dissolved polymer samples in toluene using a Hamamatsu Quantaurus-Tau Fluorescence Lifetime Spectrometer.

Supplementary Table 4 | Exciton lifetimes of selected TADF polymer thin films coated on quartz substrates measured at room temperature

Polymers (DP[a])	End group de-activation?	UV crosslinking?	χ^{b} (-)	$\tau_{avg}{}^{c}$ (μs)	$\tau_{PF}{}^{d}$ (ns)	$\tau_{DF}{}^{e}$ (μs)
**G-*ortho*-P** (DP$_{ACz}$ = 45)	N	-	1.031	0.419	24.128	1.605
	Y	-	0.997	0.202	19.686	1.262
**G-*ortho*-ELPR** (DP$_{ACz}$ = 45, DP$_{MAc}$ = 12)	Y	N	0.990	0.072	20.247	0.700
	Y	Y	0.995	0.065	17.061	0.600
**R-*ortho*-P** (DP$_{ACz}$ = 42)	Y	-	1.094	3.125	17.969	5.938
**R-*ortho*-ELPR** (DP$_{ACz}$ = 42, DP$_{MAc}$ = 9)	Y	N	1.077	1.562	16.112	3.877
**B-*para*-P** (DP$_{ACz}$ = 50)	Y	-	0.995	0.852	12.099	2.192
**B-*para*-ELPR** (DP$_{ACz}$ = 50, DP$_{MAc}$ = 7)	Y	N	0.937	0.011	10.442	0.089

a. DP was determined by ^{1}H-NMR. *b*. Fitting parameter χ for bi-exponential function fitting. *c*. Average lifetime. *d*. Prompt fluorescence lifetime. *e*. Delayed fluorescence lifetime. All the lifetimes (τ_{avg}, τ_{PF}, and τ_{DF}) are determined from spin-coated polymer thin film samples using a Hamamatsu Quantaurus-Tau Fluorescence Lifetime Spectrometer.

Supplementary Table 5 | Linker engineering for finely tuning emission properties of the green-emitting TADF polymers.

	Emission peak (nm)	Φ_{film}[b] (%)	FWHM (nm)
**TADF-*ortho*-2OH**	543	40	106
**TADF-*meta*-2OH**	561	39	109
**TADF-*para*-2OH**	565	21	117
***ortho*-initiator**	512	30	89
***meta*-initiator**	536	21	95
***para*-initiator**	539	22	96
**G-*ortho*-P-Br** (DP_{ACz} = 20)[a]	505	74	84
**G-*meta*-P-Br** (DP_{ACz} = 20)[a]	519	71	91
**G-*para*-P-Br** (DP_{ACz} = 20)[a]	522	48	91

a. DP was determined by ^{1}H-NMR.
b. Φ_{film} is measured on quartz substrate under atmosphere condition excited by 320 nm laser.

Supplementary Note 14 | Absolute thin-film PLQYs histograms of self-hosted TADF polymers, ELPRs before and after UV crosslinking measured under nitrogen atmosphere.

Please see **Supplementary Fig. 44 and Table 6.**

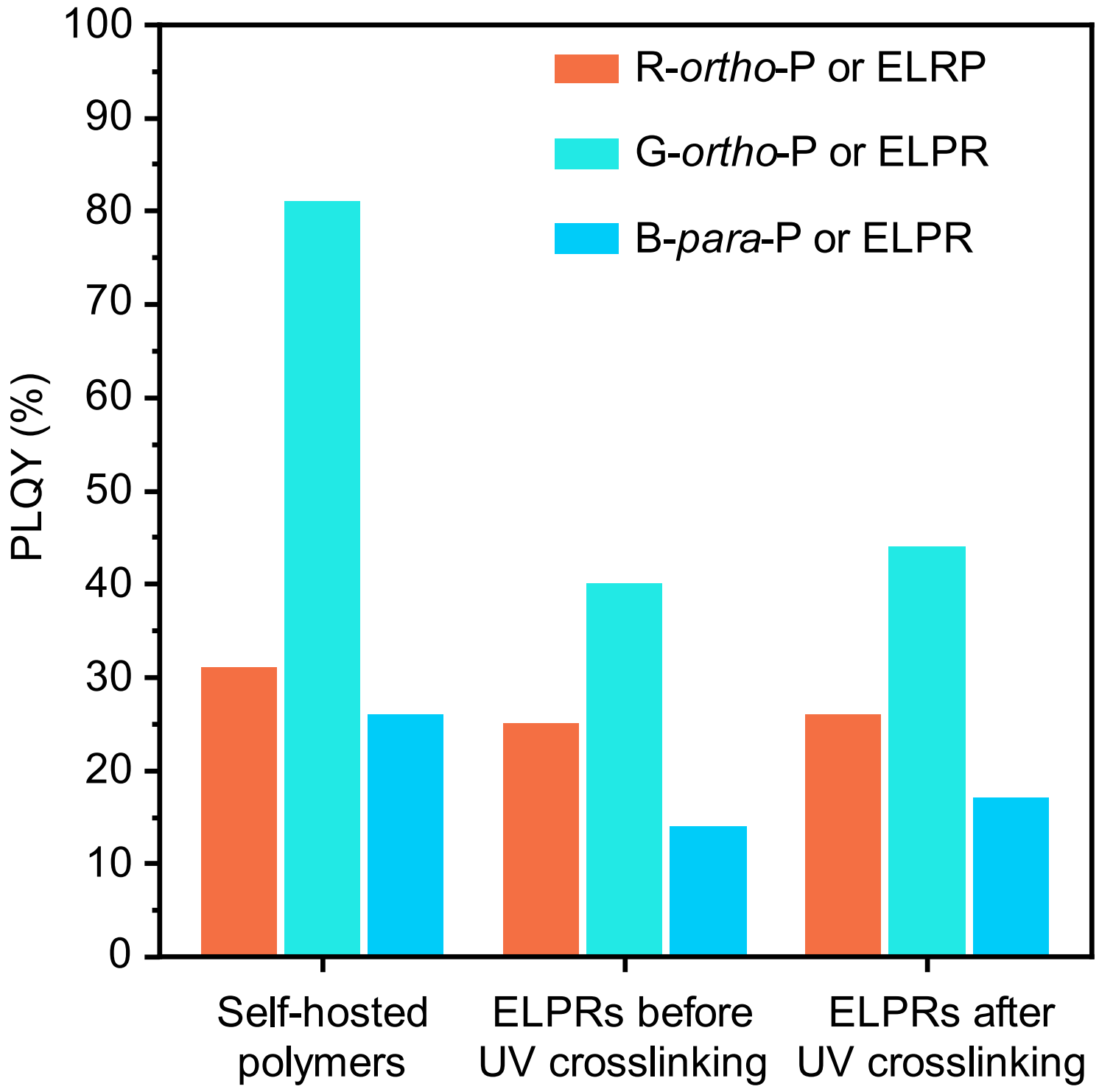

Supplementary Fig. 44: Absolute thin-film PLQYs histograms of self-hosted TADF polymers and ELPRs before and after UV crosslinking under nitrogen atmosphere. Self-hosted TADF polymers after end-group de-activation: **R-*ortho*-P** (DP_{ACz} = 42), **G-*ortho*-P** (DP_{ACz} = 45), and **B-*para*-P** (DP_{ACz} = 50); ELPRs: **R-*ortho*-ELPR** (DP_{ACz} = 42, DP_{MAc} = 9), **G-*ortho*-ELPR** (DP_{ACz} = 45, DP_{MAc} = 12), and **B-*para*-ELPR** (DP_{ACz} = 50, DP_{MAc} = 12)

Supplementary Table 6 | Summary of photophysical data of selected polymer thin films

Polymers (DP[a])	End group de-activation?[b]	Measured under air or nitrogen?	UV crosslinking?[c]	λ_{film} (nm)	Φ_{film} (%)	FWHM (nm)
**G-*ortho*-P** (DP_{ACz} = 45)	N	Air	-	500	72	87
	Y	Air	-	498	72	86
	Y	Nitrogen	-	500	81	87
**G-*ortho*-ELPR** (DP_{ACz} = 45, DP_{MAc} = 12)	N	Air	N	491	36	86
	N	Air	Y	491	38	88
	N	Nitrogen	N	490	39	86
	Y	Air	N	494	34	85
	Y	Air	Y	493	37	89
	Y	Nitrogen	N	489	38	87
	Y	Nitrogen	Y	490	44	87
**R-*ortho*-P** (DP_{ACz} = 42)	Y	Nitrogen	-	600	31	113
**R-*ortho*-ELPR** (DP_{ACz} = 42, DP_{MAc} = 9)	N	Nitrogen	N	598	24	111
	Y	Nitrogen	N	592	25	114
	Y	Nitrogen	Y	595	26	112
**B-*para*-P** (DP_{ACz} = 50)	Y	Nitrogen	-	482	30	82
**B-*para*-ELPR** (DP_{ACz} = 50, DP_{MAc} = 7)	Y	Nitrogen	N	480	14	80
	Y	Nitrogen	Y	479	17	85

a. DP was determined by ^{1}H-NMR. *b*. Y: End capping C-Br groups are debrominated. N: without end group de-activation *c*. 7000 mJ cm^{-2} of 365 nm UV is exposed to film sample for enough UV crosslinking.

Supplementary Note 15 | Mass data analysis of selected TADF polymers

Please see **Supplementary Fig. 45-49.**

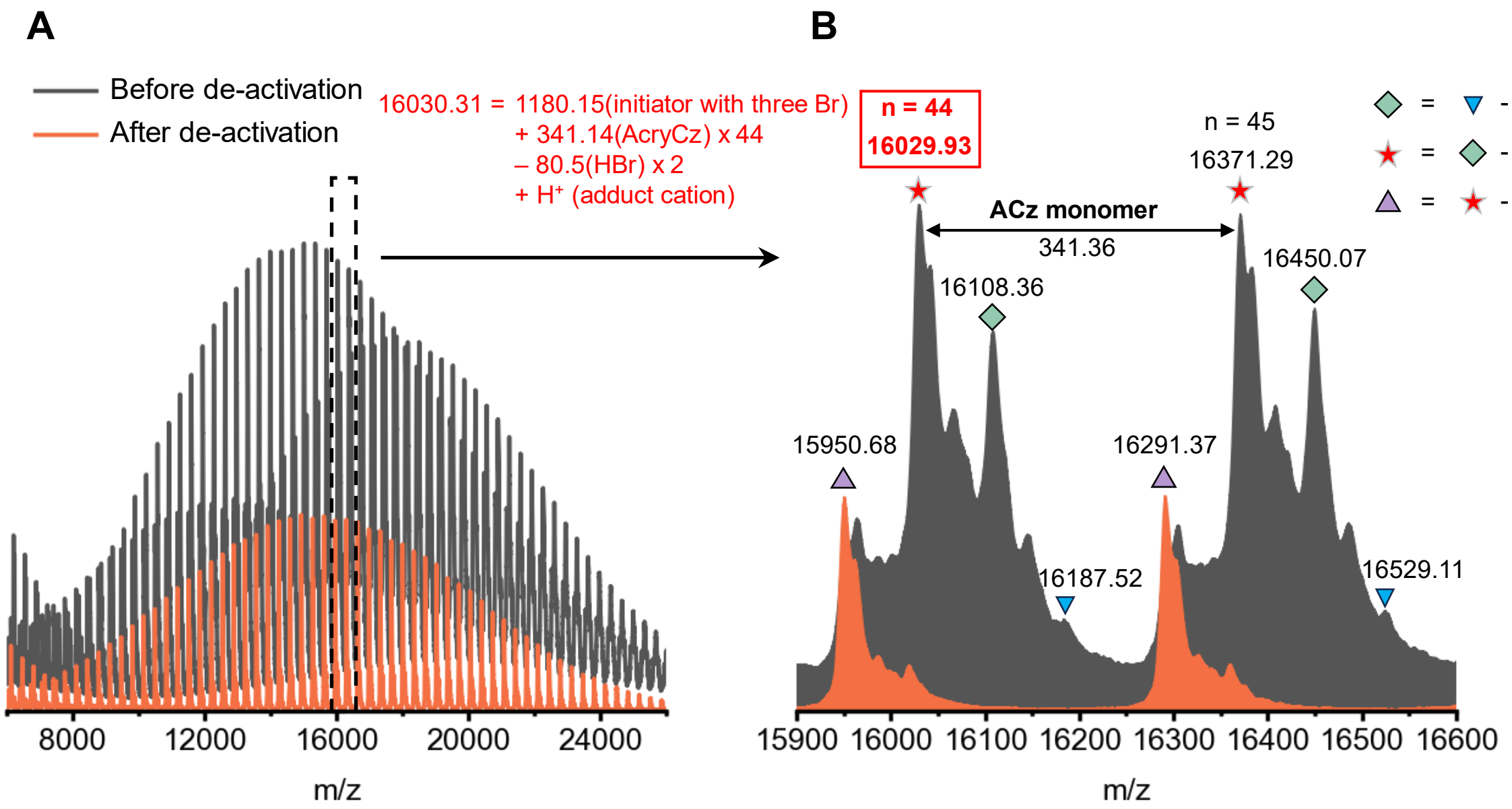

Supplementary Fig. 45: MALDI-TOF mass spectra of the selected red-emitting self-hosted TADF polymers before and after end group de-activation. (**A**) The overview MALDI-TOF mass spectra of red-emitting self-hosted polymers (DP_{ACz} = 42) before (**R-*ortho*-P-Br**) and after (**R-*ortho*-P-deBr**) end group de-activation. (**B**) The zoom-in MALDI-TOF mass spectra with the marked mass peaks. The three-armed **R-*ortho*-P-Br** bearing dormant bromides shows the debromination effect triggered by MALDI-laser, so there are four segregated peaks, where each two neighboring peaks are separated by roughly one Br atomic weight of 79.9. In contrast, the **R-*ortho*-P-deBr** doesn't have these peak patterns, proving the successful end group debromination.

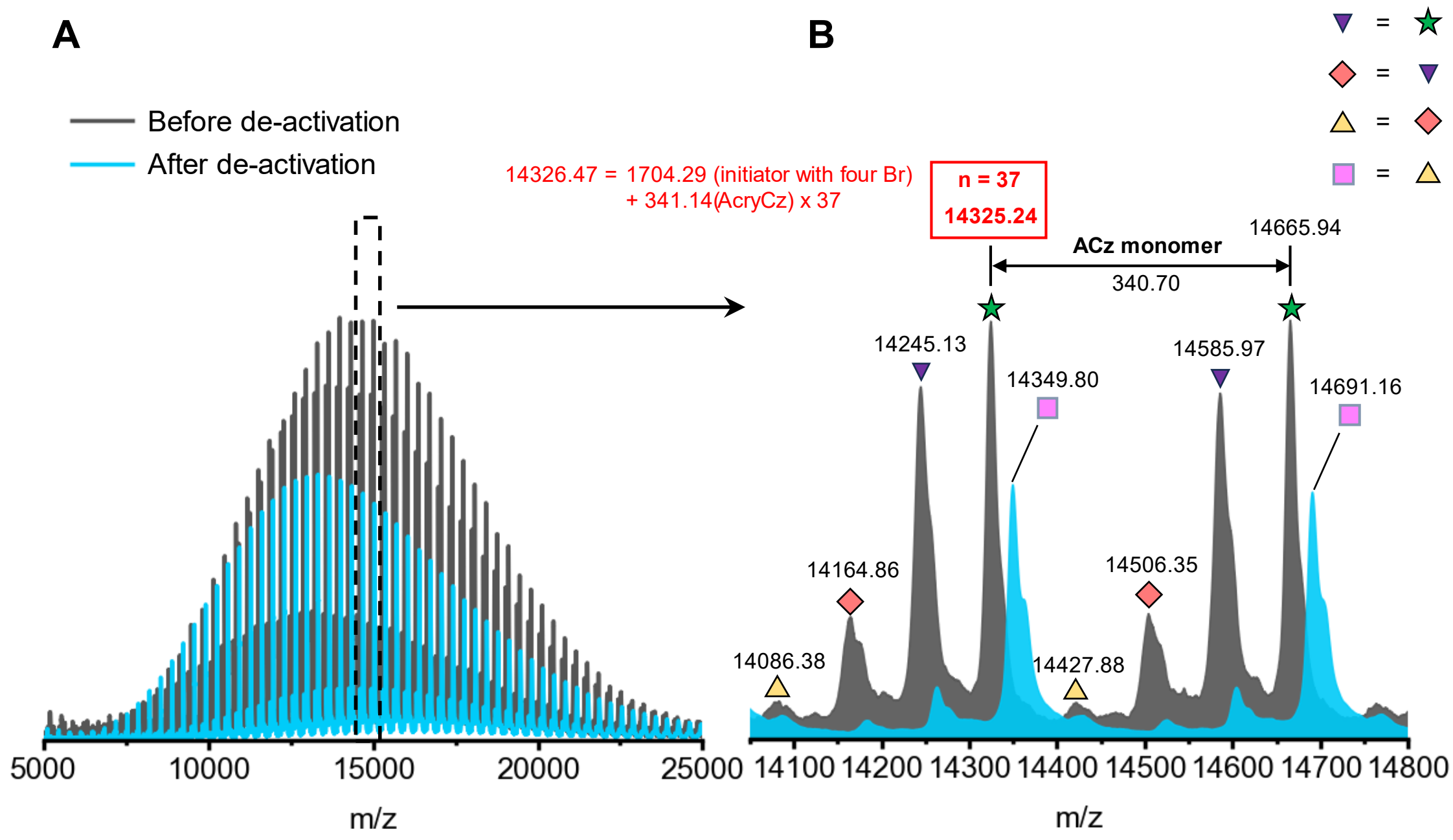

Supplementary Fig. 46: MALDI-TOF mass spectra of the selected blue-emitting self-hosted TADF polymers before and after end group de-activation. (**A**) The overview MALDI-TOF mass spectra of blue-emitting self-hosted polymers (DP_{ACz} = 60) before (**B-*para*-P-Br**) and after (**B-*para*-P-deBr**) end group de-activation. (**B**) The zoom-in MALDI-TOF mass spectra with the marked mass peaks. The four-armed **B-*para*-P-Br** bearing dormant bromides shows the debromination effect triggered by MALDI-laser, so there are five segregated peaks, where each two neighboring peaks are separated by roughly one Br atomic weight of 79.9. In contrast, the **B-*para*-P-deBr** doesn't have these peak patterns, proving the successful end group debromination.

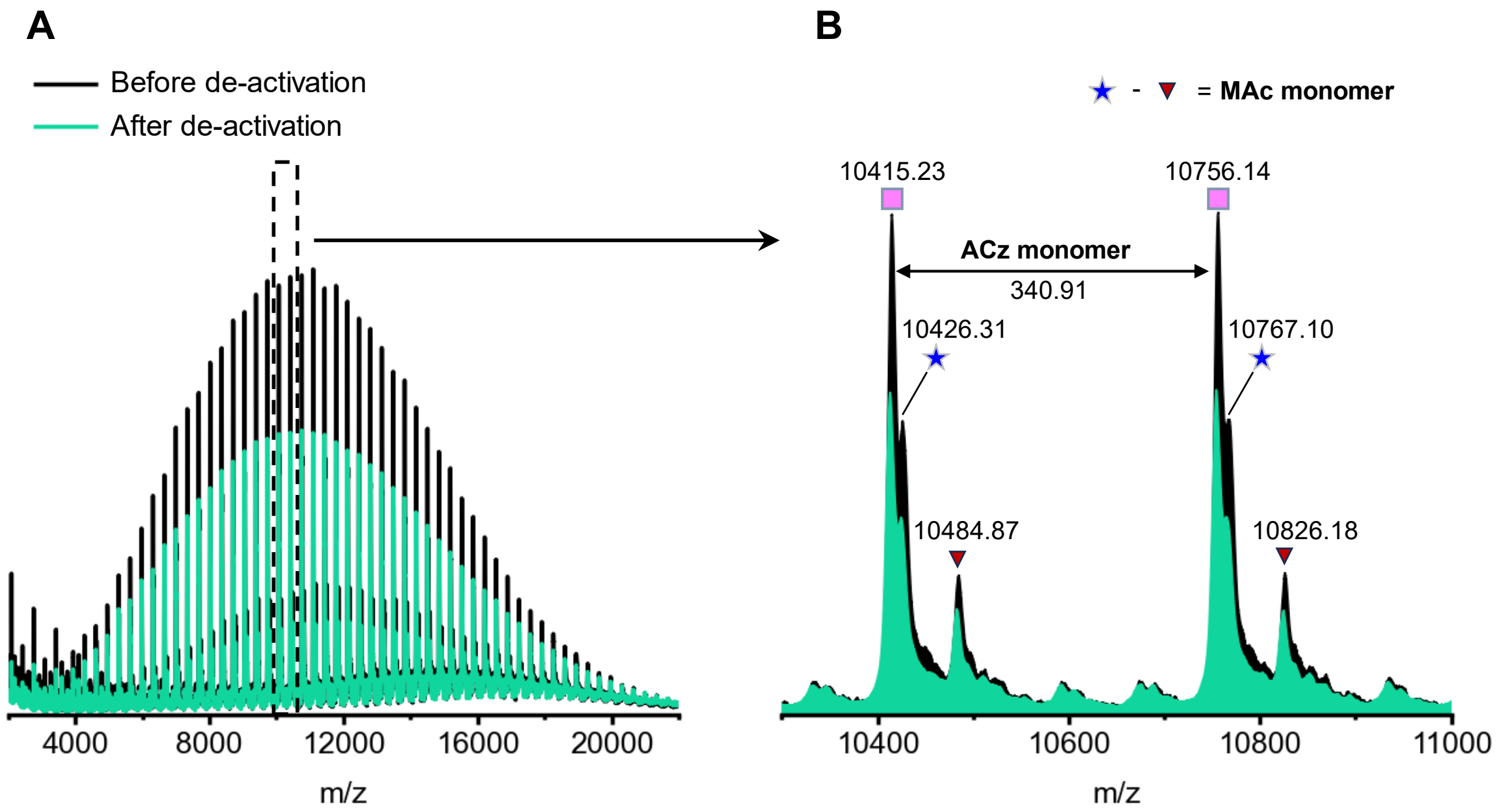

Supplementary Fig. 47: MALDI-TOF mass spectra of the selected G-*ortho*-ELPRs before and after end group de-activation. (**A**) The overview MALDI-TOF mass spectra of green-emitting ELPRs before (**G-*ortho*-ELPR-Br**) and after (**G-*ortho*-ELPR-deBr**) end group de-activation. Both of them have the same doping ratio and monomer DPs (DP_{ACz} = 45, DP_{MAc} = 12). (**B**) The zoom-in MALDI-TOF mass spectra with the marked mass peaks.

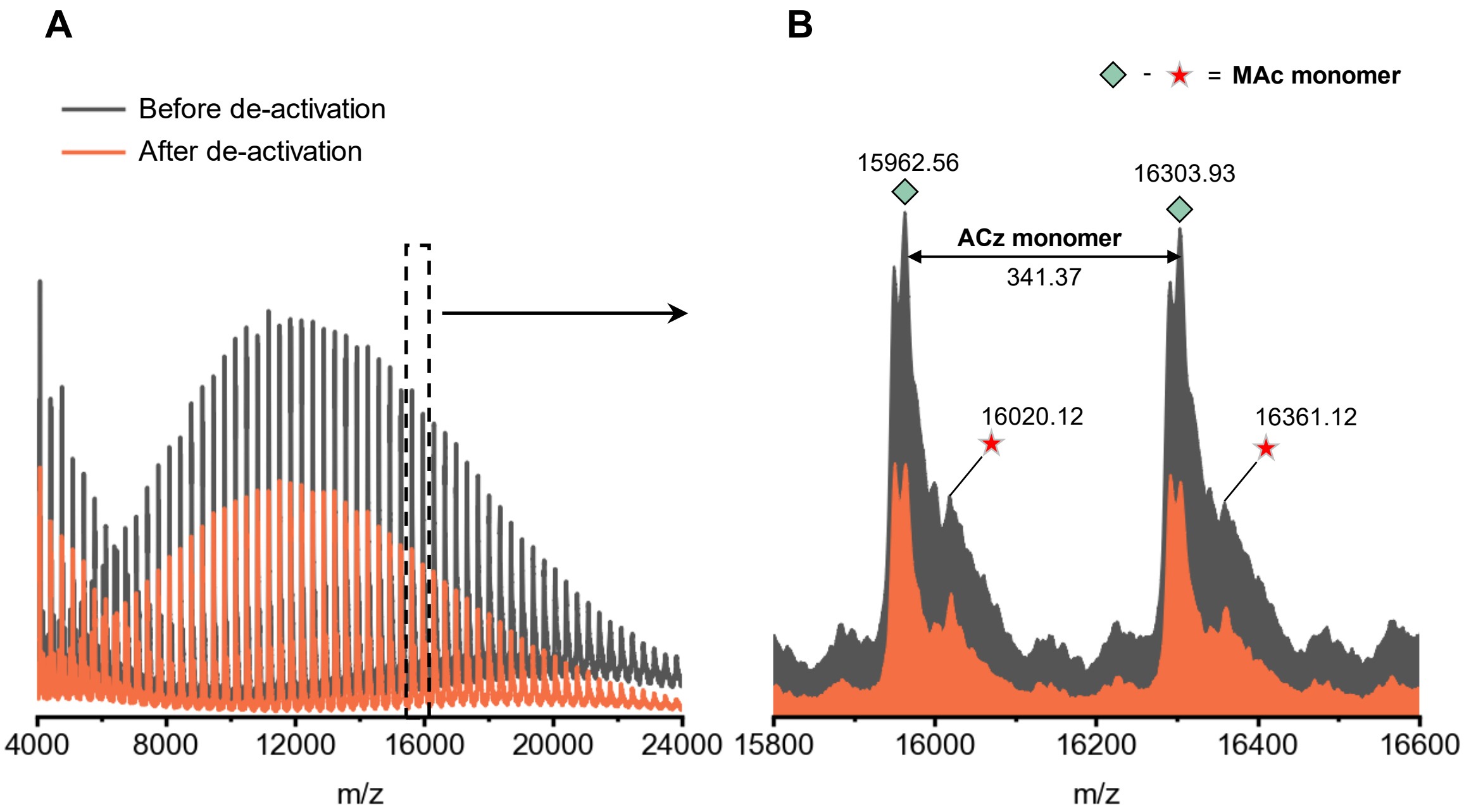

Supplementary Fig. 48: MALDI-TOF mass spectra of the selected R-*ortho*-ELPRs before and after end group de-activation. (**A**) The overview MALDI-TOF mass spectra of red-emitting ELPRs before (**R-*ortho*-ELPR-Br**) and after (**R-*ortho*-ELPRs-deBr**) end group de-activation. Both have the same doping ratio and monomer DPs (DP_{ACz} = 42, DP_{MAc} = 9). (**B**) The zoom-in MALDI-TOF mass spectra with the marked mass peaks.

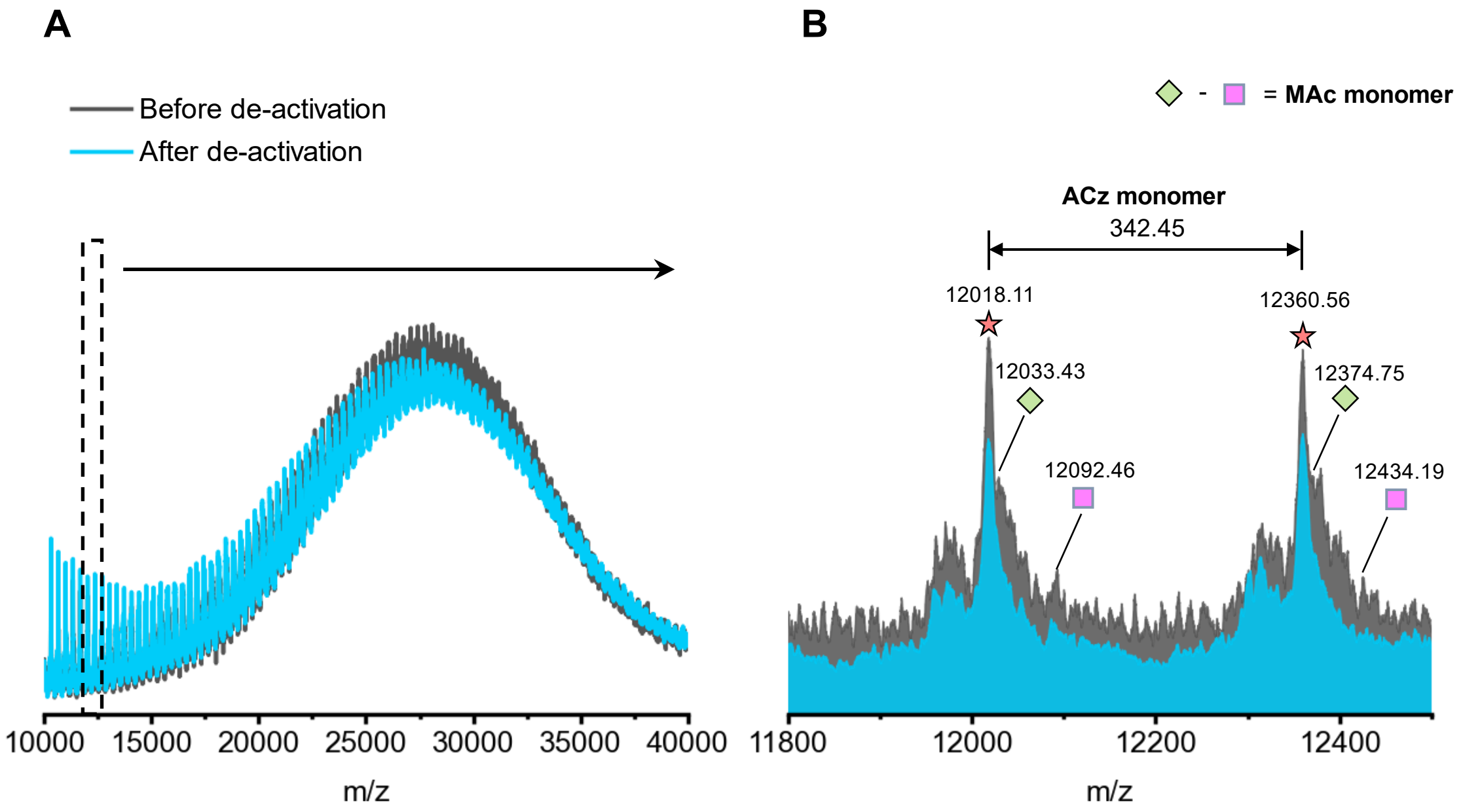

Supplementary Fig. 49: MALDI-TOF mass spectra of the selected B-*para*-ELPRs before and after end group de-activation. (**A**) The overview MALDI-TOF mass spectra of blue-emitting ELPRs before (**B-*para*-ELPR-Br**) and after (**B-*para*-ELPRs-deBr**) end group de-activation. Both have the same doping ratio and monomer DPs (DP_{ACz} = 50, DP_{MAc} = 7). (**B**) The zoom-in MALDI-TOF mass spectra with the marked mass peaks.

Supplementary Note 16 | Electrochemical characterization

Please see **Supplementary Fig.50 and Table 7.**

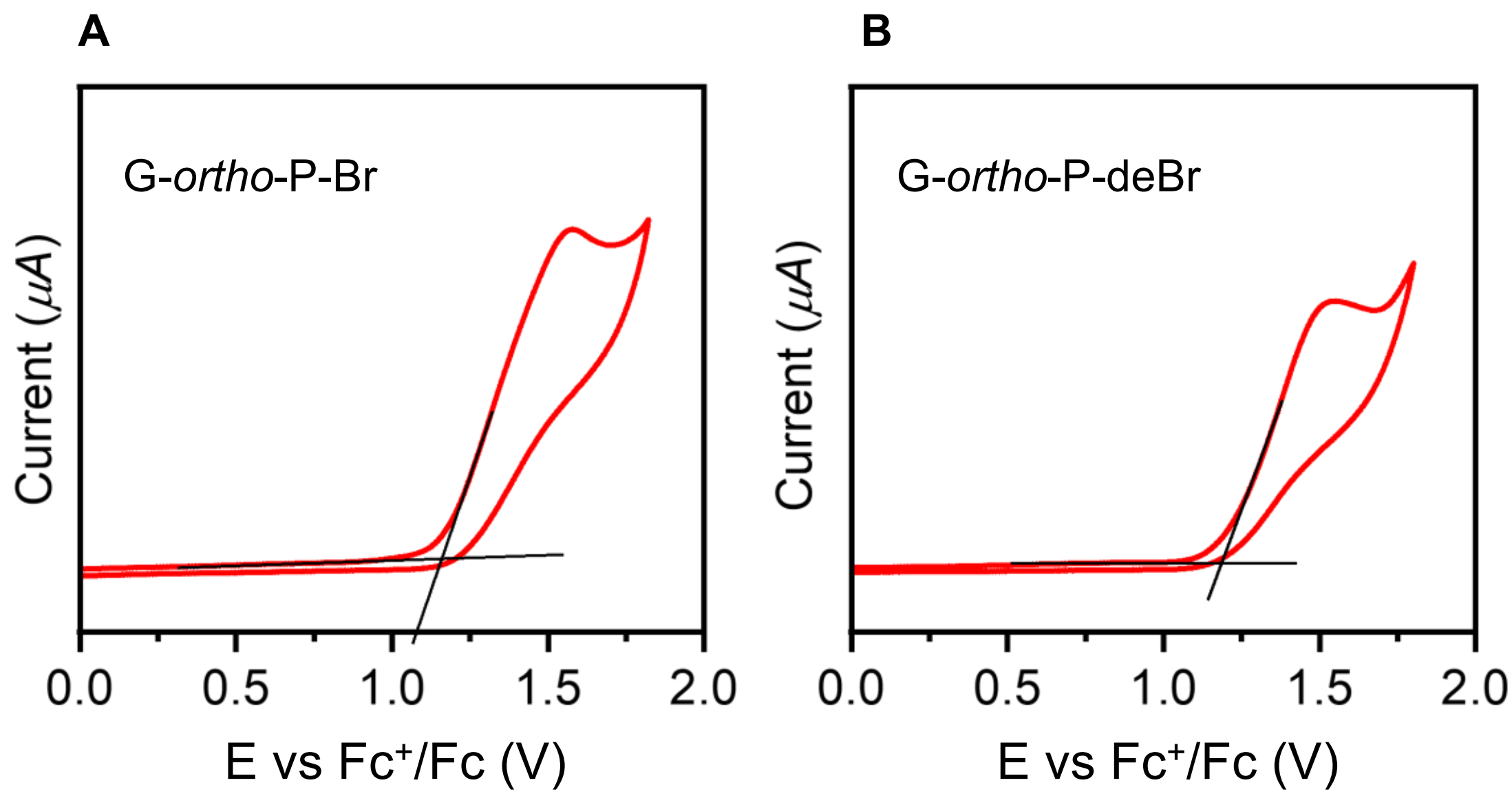

Supplementary Fig. 50: (**A**) CV characterization of **G-*ortho*-P-Br** (DP_{ACz} = 45) before end group de-activation (**B**) CV characterization of **G-*ortho*-P-deBr** (DP_{ACz} = 45) after end group de-activation

Supplementary Table 7 | Electrochemical characterization data of G-*ortho*-P before and after end group de-activation

Polymer (DP[a])	End group de-activation?	$E_{oxd}^{1/2}$ (eV)	HOMO[b] (eV)	λ_{film} (nm)	E_g[c] (eV)	LUMO[d] (eV)
**G-*ortho*-P** (DP_{ACz} = 45)	N	1.16	5.96	500	2.48	3.48
	Y	1.20	6.01	498	2.49	3.52

a. DP was determined by ^{1}H-NMR. *b*. HOMO level is calculated from the oxidation half-wave potential. *c*. E_g is calculated from $\frac{1240}{\lambda_{max}}$. *d*. LUMO level is calculated from HOMO - E_g.

Supplementary Note 17 | Thermal stability characterization of G-*ortho*-P-deBr

Please see **Supplementary Fig. 51.**

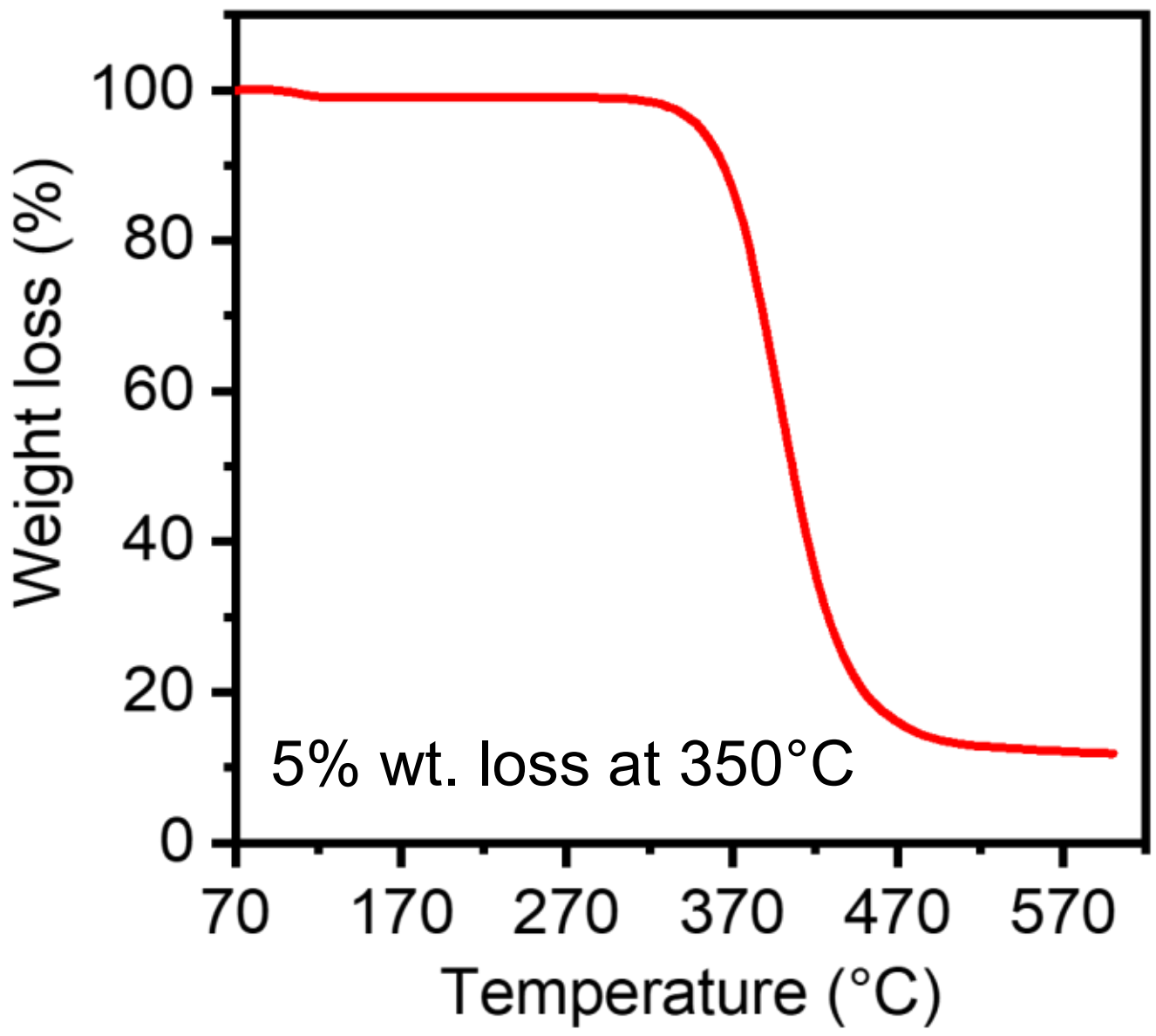

Supplementary Fig. 51: TGA analysis of G-*ortho*-P-deBr (DP_{ACz} = 45) after end group de-activation. The thermal stability of **G-*ortho*-P-deBr** was determined by the 5% weight loss at the heating temperature of 350 °C.

Supplementary Note 18 | OLED current efficiency enhancement of green-emitting self-hosted TADF polymer after end-group deactivation.

Please see **Supplementary Fig. 52.**

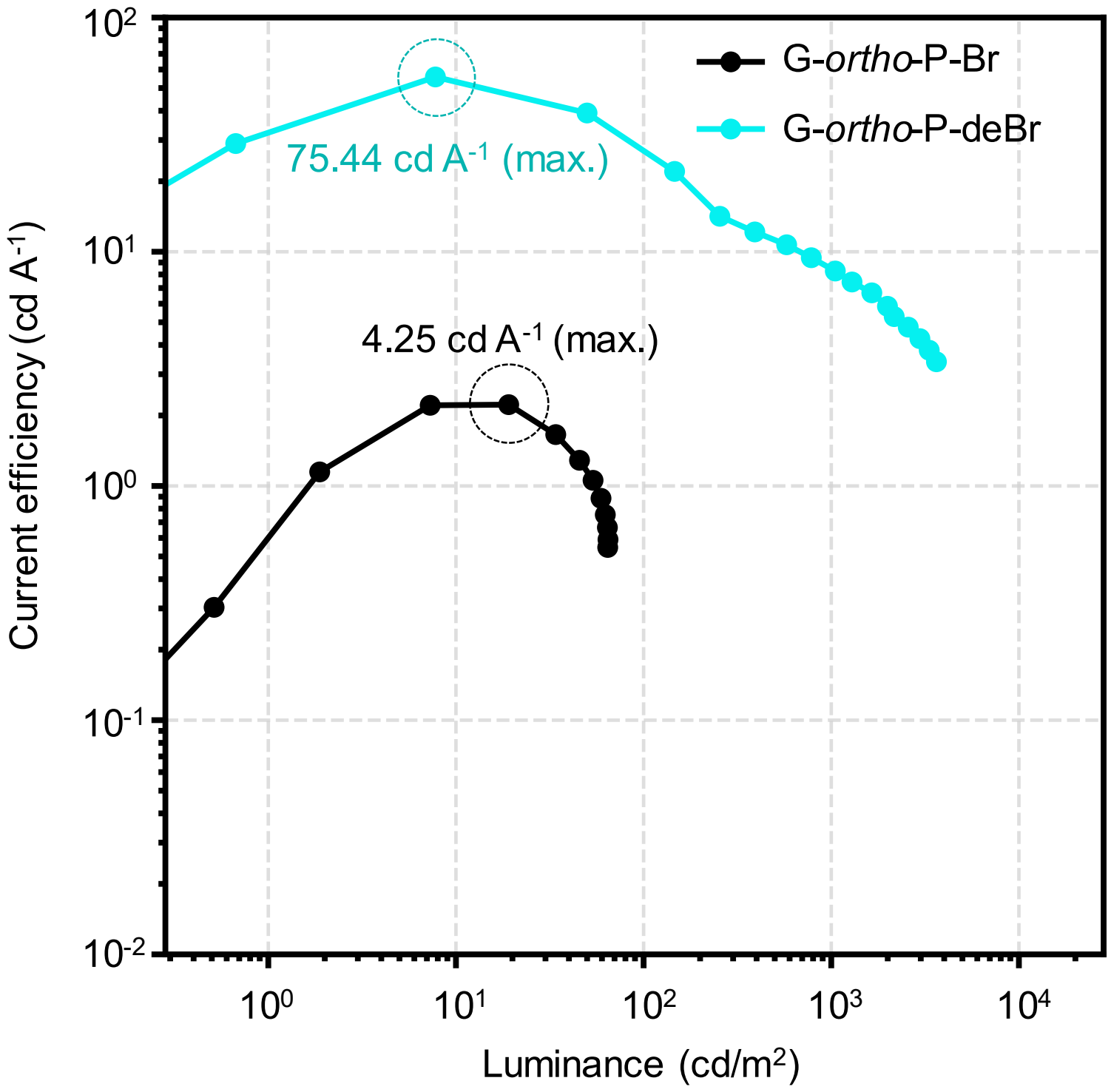

Supplementary Fig. 52: OLED current efficiency enhancement of green-emitting self-hosted TADF polymer before and after end-group deactivation. Self-hosted TADF polymer before (**G-*ortho*-P-Br**) and after (**G-*ortho*-P-deBr**) end-group de-activation with DP_{ACz} = 45.

Supplementary Note 19 | Operational stability performance of green-emitting self-hosted TADF polymer and ELPR with different device architecture.

Please see **Supplementary Fig. 53.**

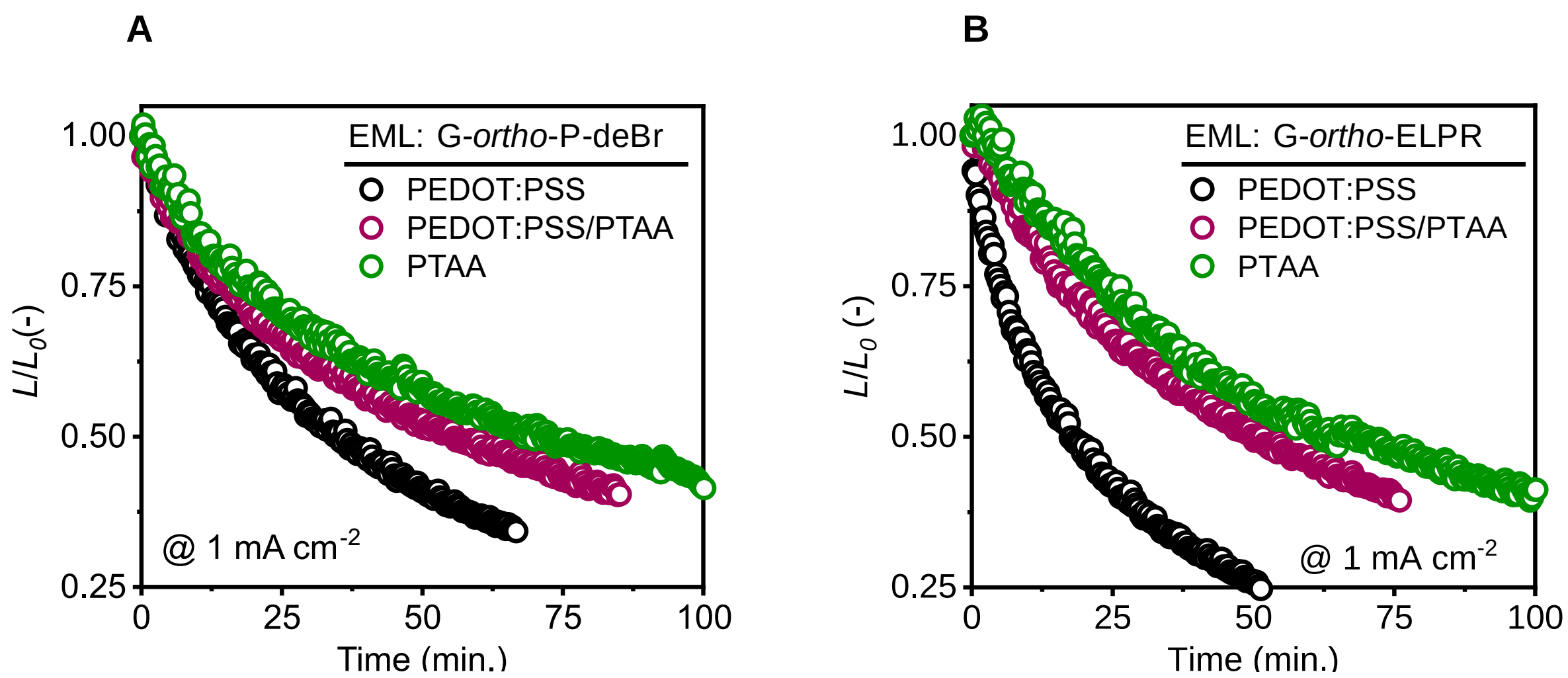

***Supplementary Fig. 53*: OLED operational stability lifetime study of green-emitting self-hosted G-*ortho*-P-deBr and G-*ortho*-ELPR before dialysis treatment at a constant current density of 1 mA cm^{-2}.** To investigate the potential of our ELPR platform, half solution processed and half evaporated devices with the following architecture were fabricated: ITO (50 nm); HTL; polymeric EML; TmPyPB (ETL , 50 nm); Liq (1.5 nm); Al (100 nm), where either (**A**) self-hosted precursor **G-*ortho*-P-deBr** or (**B**) photo-patternable **G-*ortho*-ELPR** (UV crosslinked) as EML was deposited under different HTL layers (PEDOT:PSS only, PEDOT:PSS/PTAA, or PTAA only) in order to improve their LT_{50} lifetime. With the sole PTAA as HTL underneath **G-*ortho*-ELPR**, this device architecture exhibited the longest LT_{50} of ~70 minutes and with merely ~4% drop compared to **G-*ortho*-P-deBr**. These results showed that the lifetime is highly dependent on the device architecture optimization, and the operational lifetime is relatively immune from the crosslinker integration and the subsequent UV crosslinking in our ELPR system.

Supplementary Note 20 | Extraction of photophysical kinetic parameters for green-emitting TADF polymers

Please see **Supplementary Fig.54 and Table 8.**

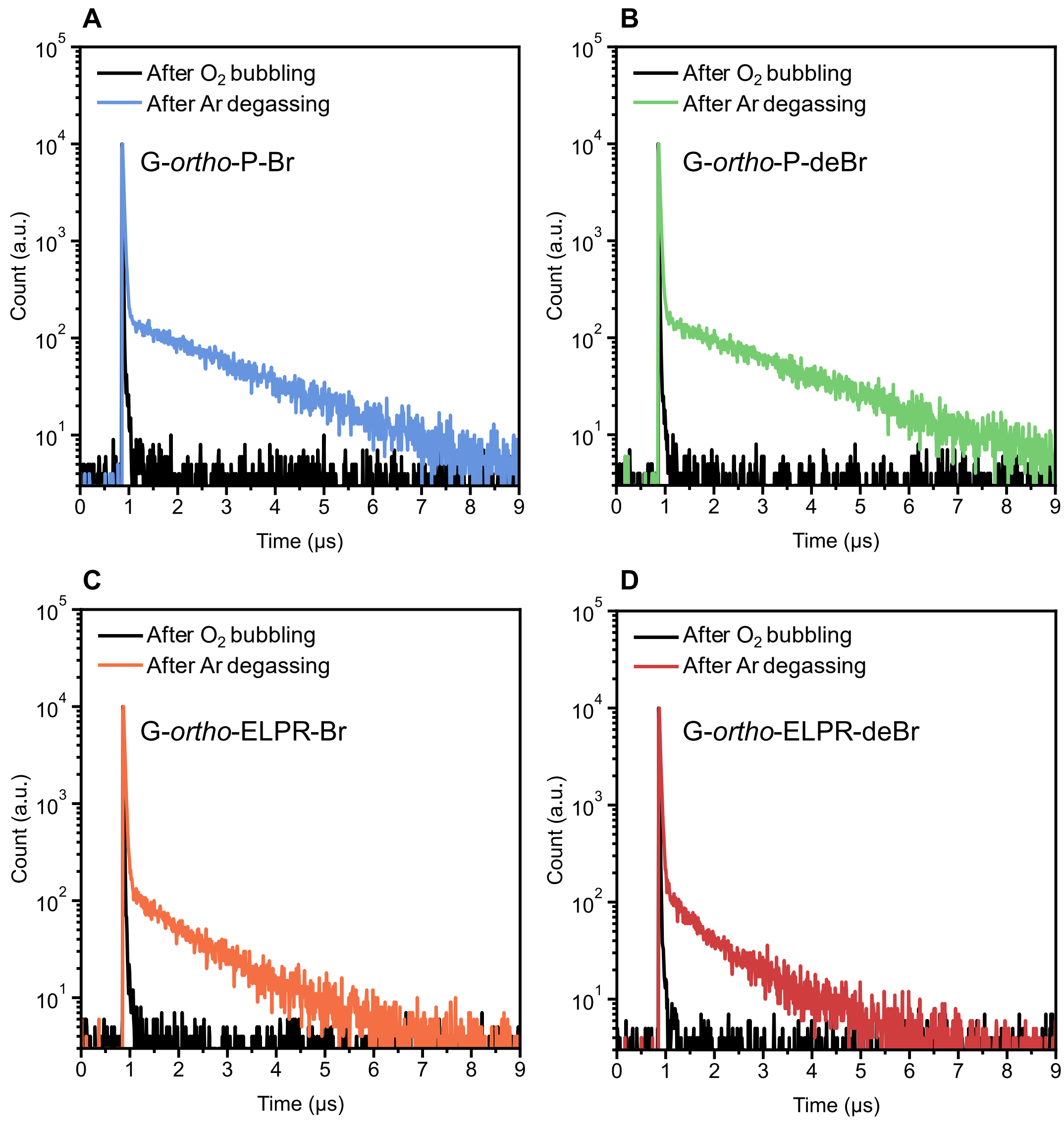

Supplementary Fig. 54: TRPL responses for the synthesized green-emitting TADF polymers dissolved in toluene measured at room temperature. (A) G-ortho-P-Br (DPACz = 45, before end-group de-activation), (B) G-ortho-P-deBr (DPACz = 45, after end-group de-activation), (C) G-ortho-ELPR-Br (DPACz = 45, DPMAc = 12), (D) G-ortho-ELPR-deBr (DPACz = 45, DPMAc = 12).

Supplementary Table 8 | Extracted photophysical characteristics for the green-emitting TADF polymers dissolved toluene solution (0.1 mM)..

**Polymers (DP[a])**	**G-*ortho*-P** (DP_{ACz} = 45)		**G-*ortho*-ELPR** (DP_{ACz} = 45, DP_{MAc} = 12)	
**End group de-activation?**	N	Y	N	Y
$\lambda_{PL}{}^{b}$ **(nm)**	520	521	519	520
$\tau_{PF}{}^{c}$ **(ns)**	27.1	27.6	27.5	28.0
$\tau_{DF}{}^{d}$ **(μs)**	2.04	2.17	1.14	0.98
$\Phi_{tot}{}^{e}$ **(%)**	67	83	50	46
$\Phi_{PF}{}^{f}$ **(%)**	11	12	11	11
$\Phi_{DF}{}^{g}$ **(%)**	56	71	39	35
$k_{PF}{}^{h}$ **(s^{-1})**	3.69×10^{7}	3.62×10^{7}	3.64×10^{7}	3.57×10^{7}
$k_{DF}{}^{h}$ **(s^{-1})**	4.90×10^{5}	4.61×10^{5}	8.75×10^{5}	1.02×10^{6}
$k_{r}^{S\,h}$ **(s^{-1})**	4.06×10^{6}	4.35×10^{6}	4.00×10^{6}	3.93×10^{6}
$k_{nr}^{T\,h}$ **(s^{-1})**	1.82×10^{5}	8.91×10^{4}	4.92×10^{5}	6.18×10^{5}
$k_{RISC}{}^{h}$ **(s^{-1})**	2.80×10^{6}	3.10×10^{6}	3.49×10^{6}	3.64×10^{6}
$k_{ISC}{}^{h}$ **(s^{-1})**	3.28×10^{7}	3.19×10^{7}	3.24×10^{7}	3.18×10^{7}

a. DP was determined by ^{1}H-NMR. *b*. emission peak. *c*. τ_{PF} is prompt fluorescence lifetime measured after oxygen bubbling. *d*. τ_{DF} is delayed fluorescence lifetime measured after argon degassing. *e*. Φ_{tot} is solution PLQY measured after argon degassing. *f*. Φ_{PF} is PLQY prompt component measured after oxygen bubbling. *g*. Φ_{DF} is PLQY delayed component calculated by $\Phi_{DF} = \Phi_{tot} - \Phi_{PF}$. All the lifetimes ($\tau_{PF}$ and τ_{DF}) are determined from dissolved polymer samples in toluene using a Hamamatsu Quantaurus-Tau Fluorescence Lifetime Spectrometer. *h*. various rate constants were estimated using Goushi and Masui method Ref.#15-16.

Supplementary Note 21 | Reliability and reproducibility characterization

Please see **Supplementary Figs. 55-58.**

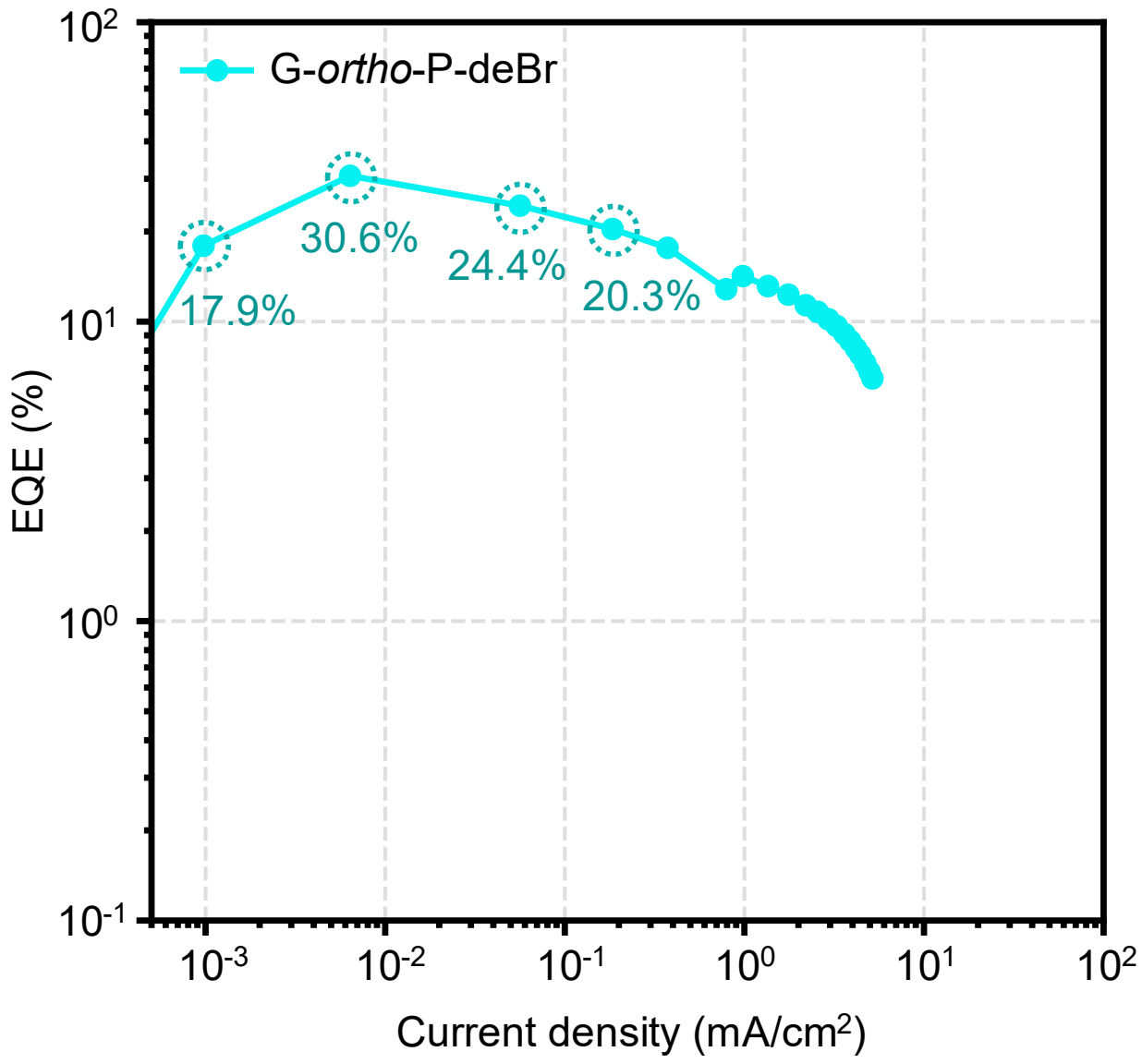

Supplementary Fig. 55: Independent cross-check of the G-*ortho*-P-deBr OLED performance using a calibrated PR655 spectroradiometer. The EQE–current density curve shows a maximum EQE of 30.6% and still exhibit the characteristic double-peak behavior, with multiple EQE data points exceeding 20%.

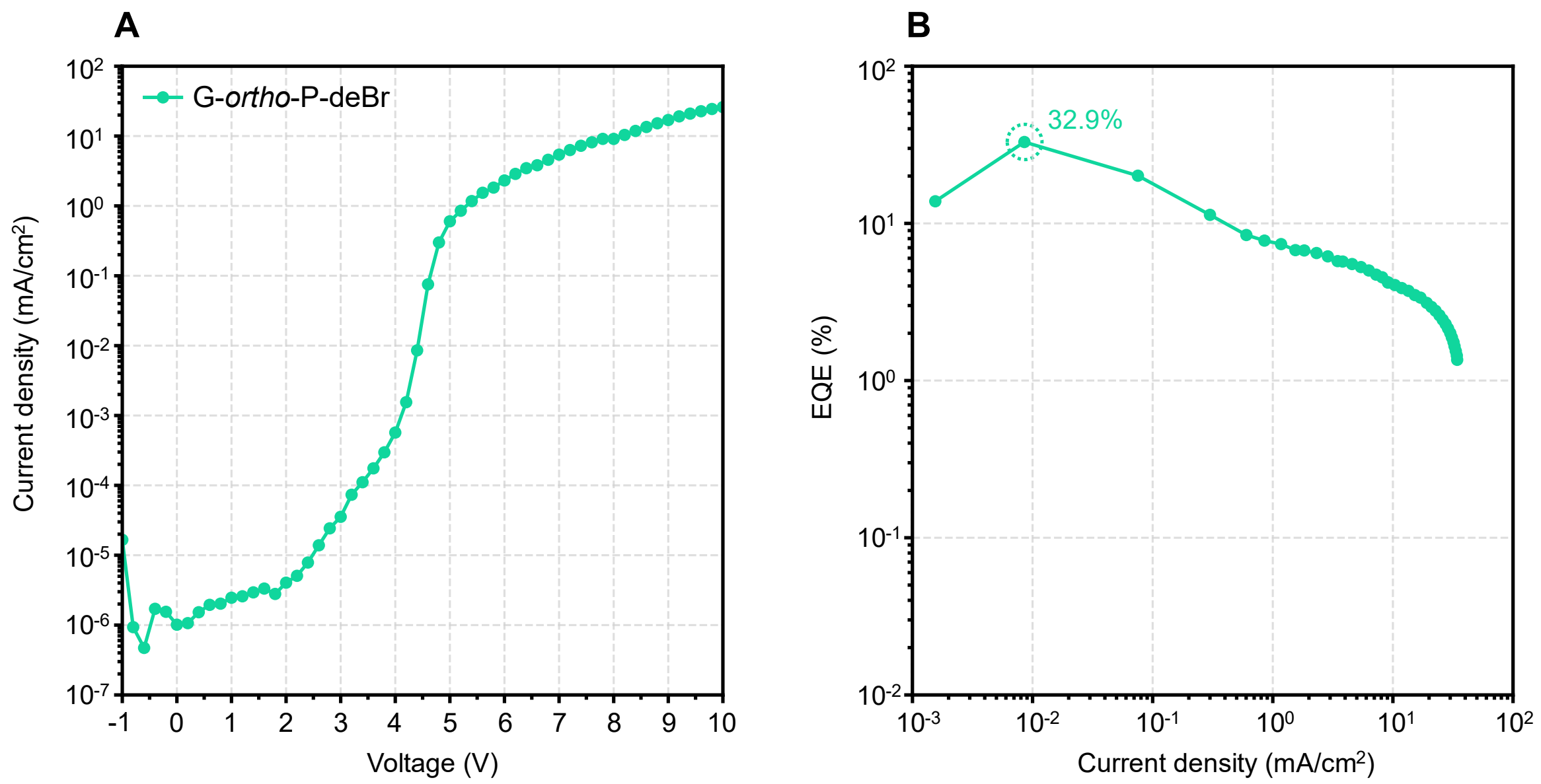

Supplementary Fig. 56: Current density-voltage (A) and EQE-voltage (B) curves of the G-*ortho*-P-deBr OLED measured by sweeping the voltage from −1 V to 10 V to avoid in-built charge accumulation. Before turn-on (below 3 V), the device exhibits a low leakage current density of < 10^{-4} mA cm^{-2}, and a maximum EQE exceeding 30% is still reproducibly recorded.

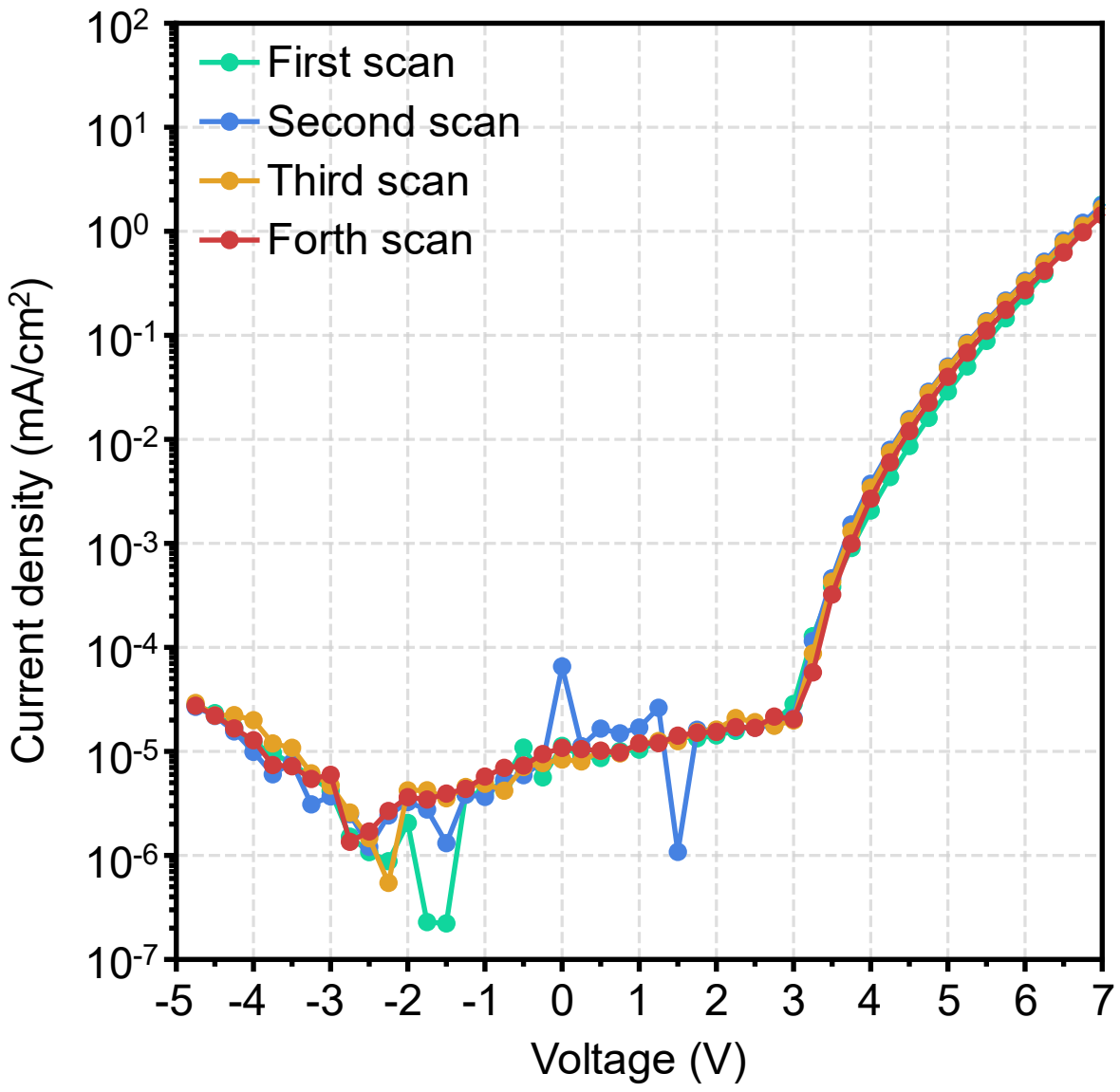

Supplementary Fig. 57: Four consecutive J–V scans of the G-*ortho*-P-deBr OLED measured by sweeping the voltage from −1 V to 10 V. The scans overlap closely over both the negative and positive voltage ranges, with only modest fluctuations between repeats.

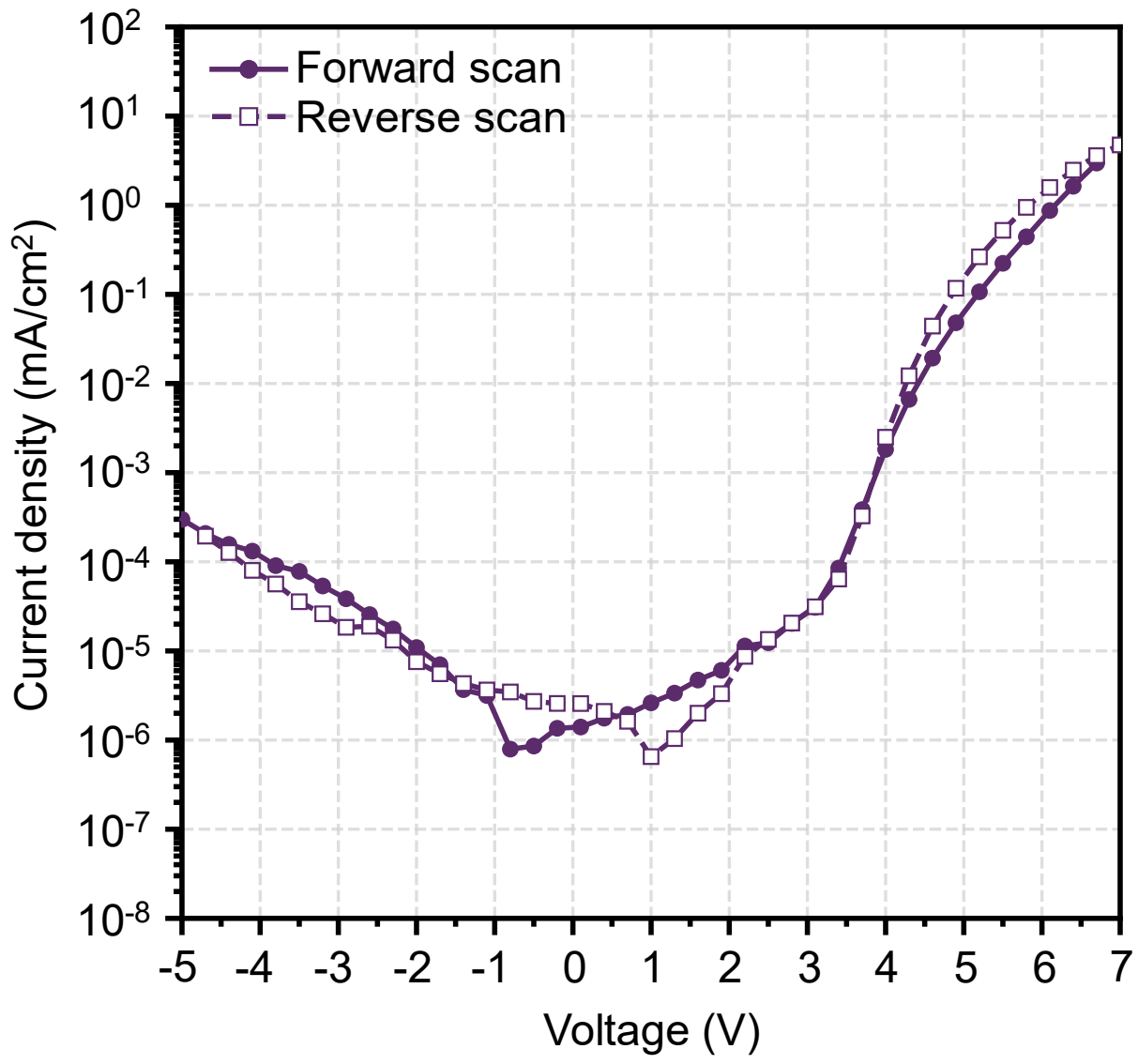

Supplementary Fig. 58: Forward and reverse *J-V* curves of the G-*ortho*-P-deBr OLED measured by sweeping the voltage from −5 V to 7 V for forward scan and from 7 V to -5 V for reverse scan. The nearly overlap behavior in both scan directions confirm stable charge-transport behavior under directional biasing.

Supplementary Note 22 | Microscopic characterization

Optical micrograph please see **Supplementary Fig. 59.** AFM images please see **Supplementary Figs. 60 and 61.**

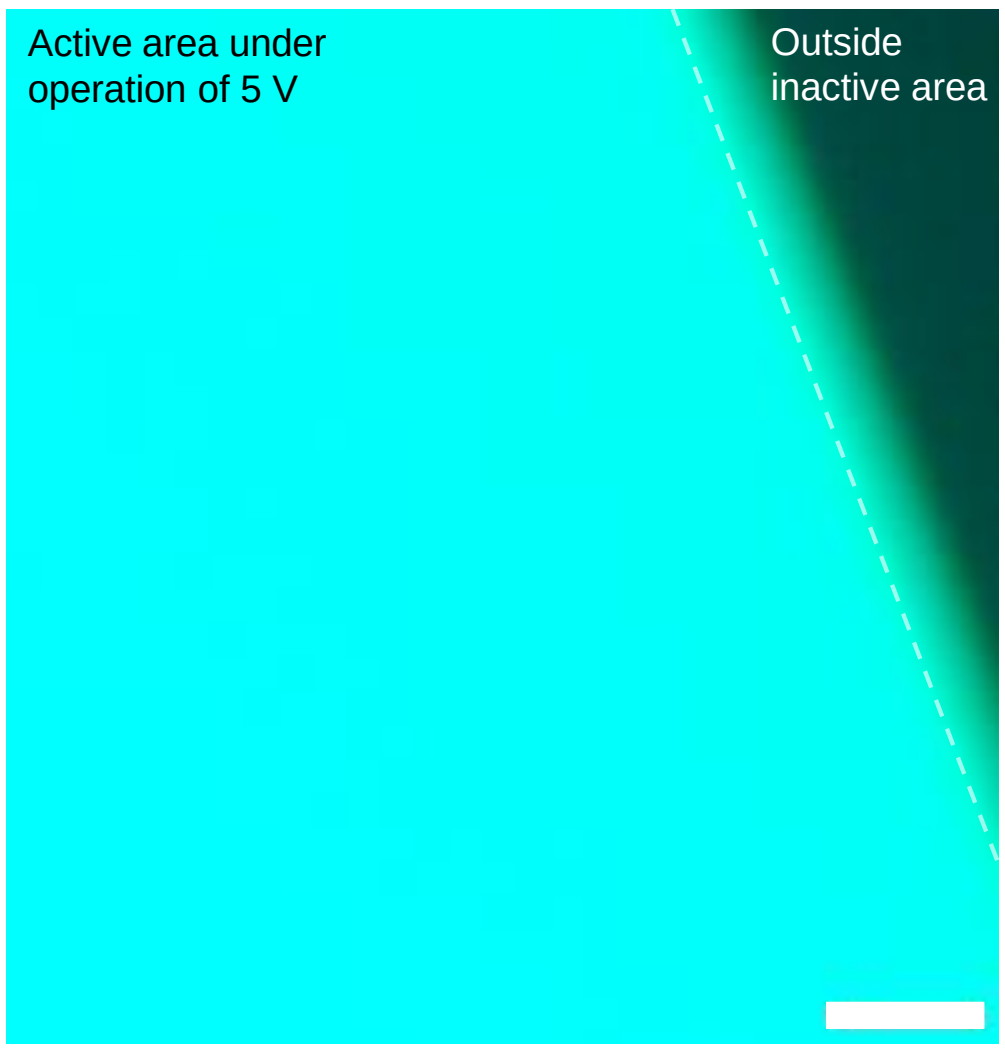

Supplementary Fig. 59: EL image of the G-*ortho*-P-deBr OLED device operated under 5 V. Device architecture: ITO/PEDOT:PSS (CH 8000)/G-*ortho*-P-deBr/TmPyPB/Liq/Al. Scale bar: 25 μm.

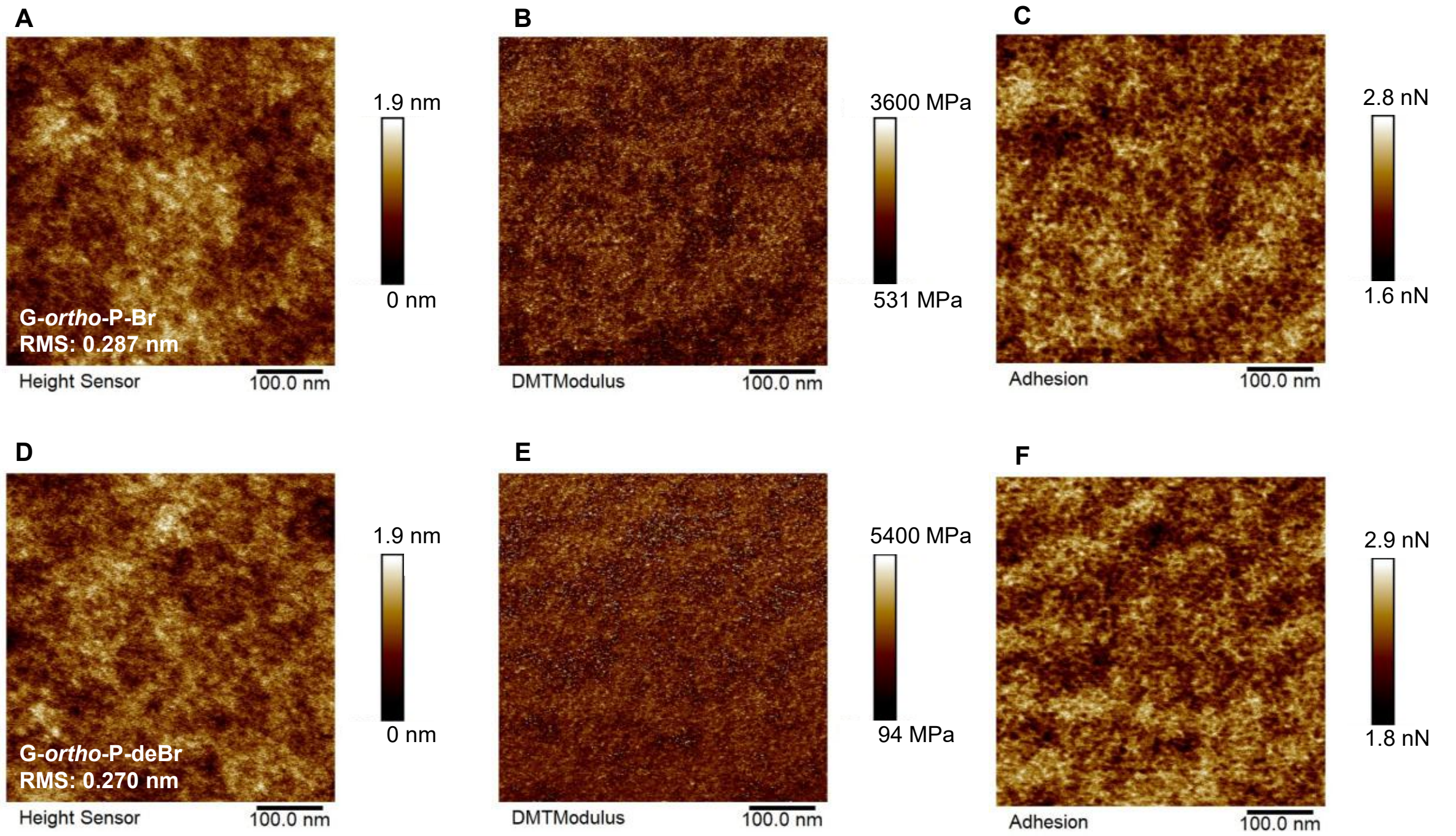

Supplementary Fig. 60: AFM QNM characterization images of G-*ortho*-P-Br (from A to C) and G-*ortho*-P-deBr (from D to F) thin films. (**A**, **D**) Height channel images, (**B**, **E**) DMT modulus images, (**C**, **F**) Adhesion channel images.

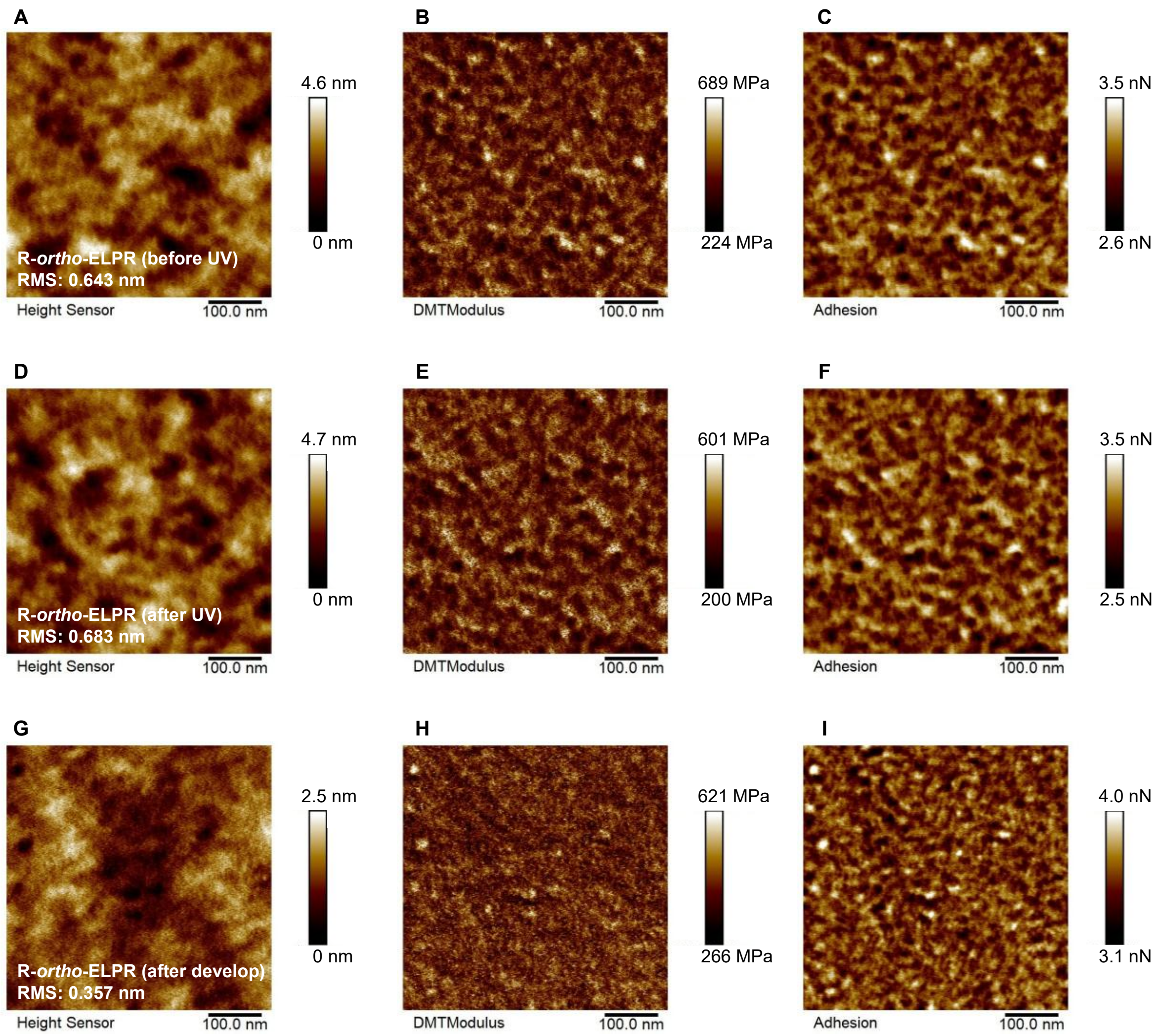

Supplementary Fig. 61: AFM QNM characterization images of R-ortho-ELPR thin films at different photolithography process stages (before UV exposure; from A to C), (after UV exposure; from D to F), (after development; from G to I). (**A**, **D**, **G**) Height channel images, (**B**, **E**, **H**) DMT modulus images, (**C**, **F**) Adhesion channel images.

Supplementary Note 23 | Quantification of copper residue within TADF polymer inherited from ATRP synthesis

Please see **Supplementary Fig.62 and Table 9.**

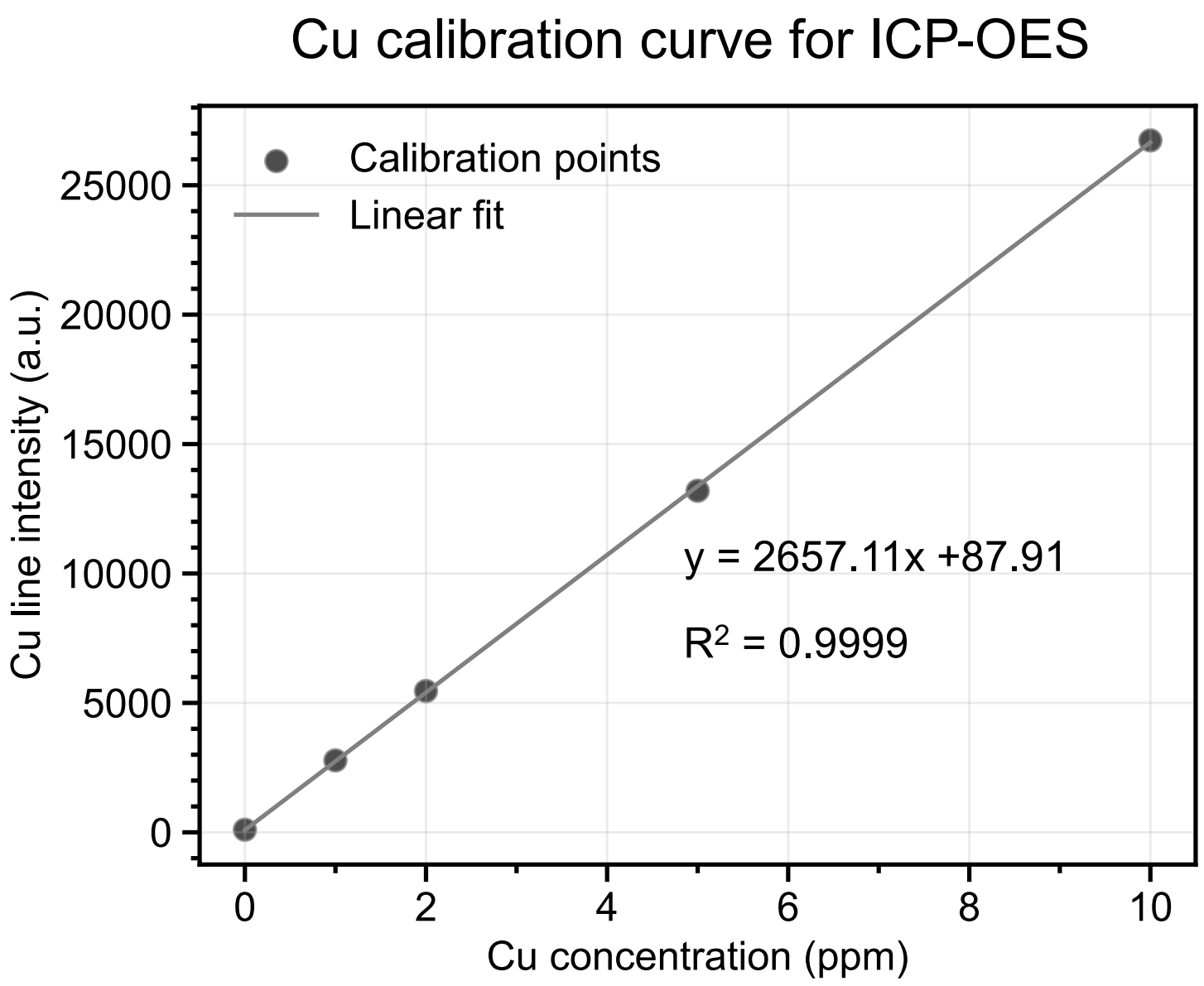

Supplementary Fig. 62: Calibration curve of copper standards used for ICP-OES measurement.

Supplementary Table 9 | Quantification of copper residue concentration in the G-*ortho*-P-deBr polymer sample before and after dialysis.

G-*ortho*-P-deBr	Before dialysis	After dialysis
**Cu line intensity at 327 nm (a.u.)**	407.36	251.96
**Detected Cu conc. (ppm; mg/L)**	0.12	0.06
**Volume of the diluted sample (L)**	0.015	0.015
**Cu mass in the diluted sample (mg)**	0.002	0.001
**Digested polymer mass (mg)**	66.5	64.1
**Cu conc. in polymer sample (ppm)**	**27.12**	**14.45**

Supplementary Note 24 | Optimized operational stability lifetime by dialysis treatment and device optimization

Please see **Supplementary Fig. 63.**

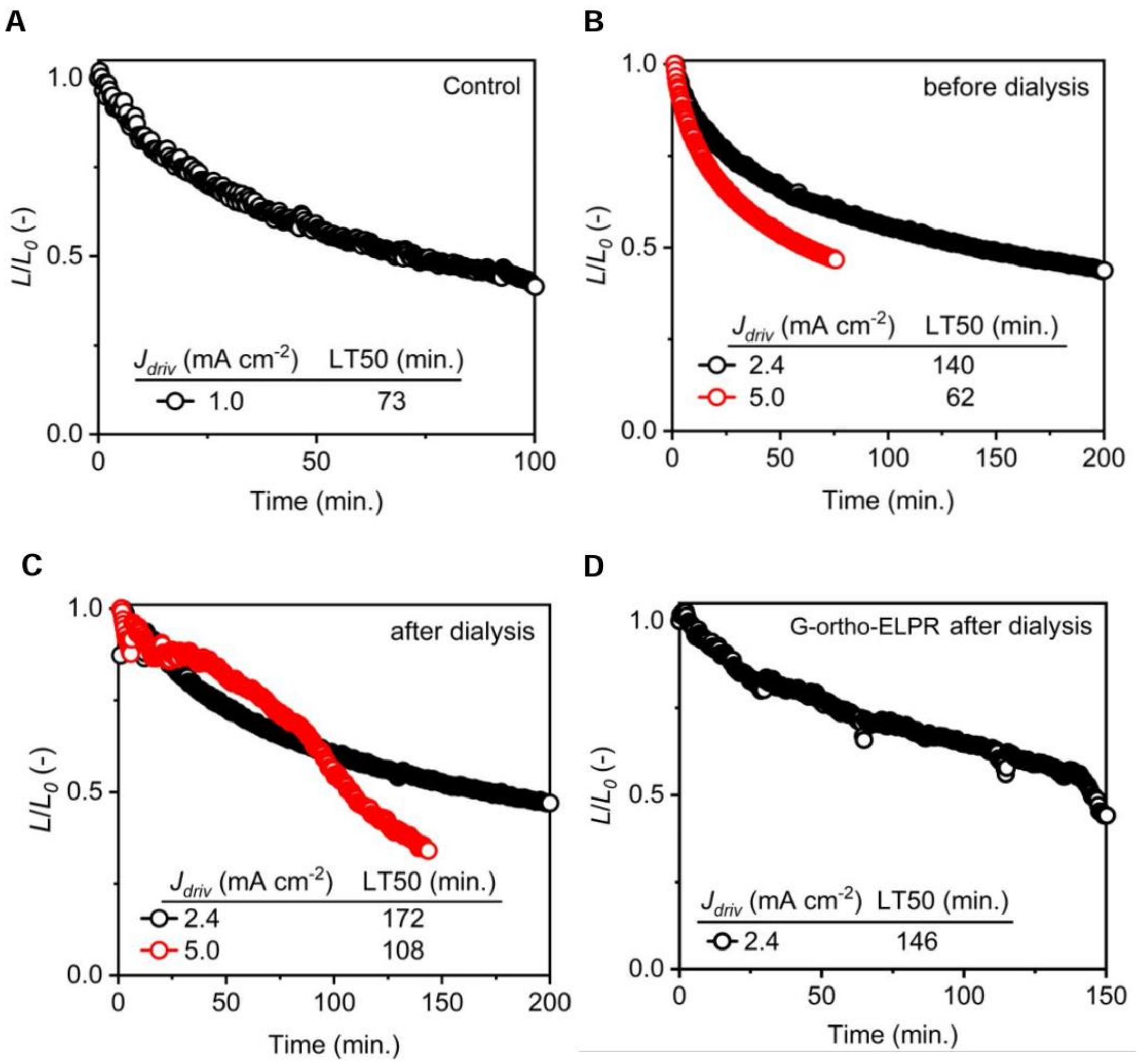

Supplementary Fig. 63: Operational stability of OLED devices. (**A**) Control device architecture of ITO/PTAA/G-*ortho*-P-deBr before dialysis/TmPyPB/Liq/Al. Optimal device with a layer sequence of ITO/v-NPB/G-*ortho*-P-deBr/TPBi/Liq/Al consisting green-emitting self-hosted G-*ortho*-P-deBr TADF polymer emission layer (**B**) before and (**C**) after dialysis purification to reduce copper residue inherited from ATRP synthesis. (**D**) non-crosslinked G-*ortho*-ELPR after dialysis as EML in optimal device.

Supplementary Note 25 | OLED devices optimized for high brightness and high current density

Please see **Supplementary Figs. 64-67.**

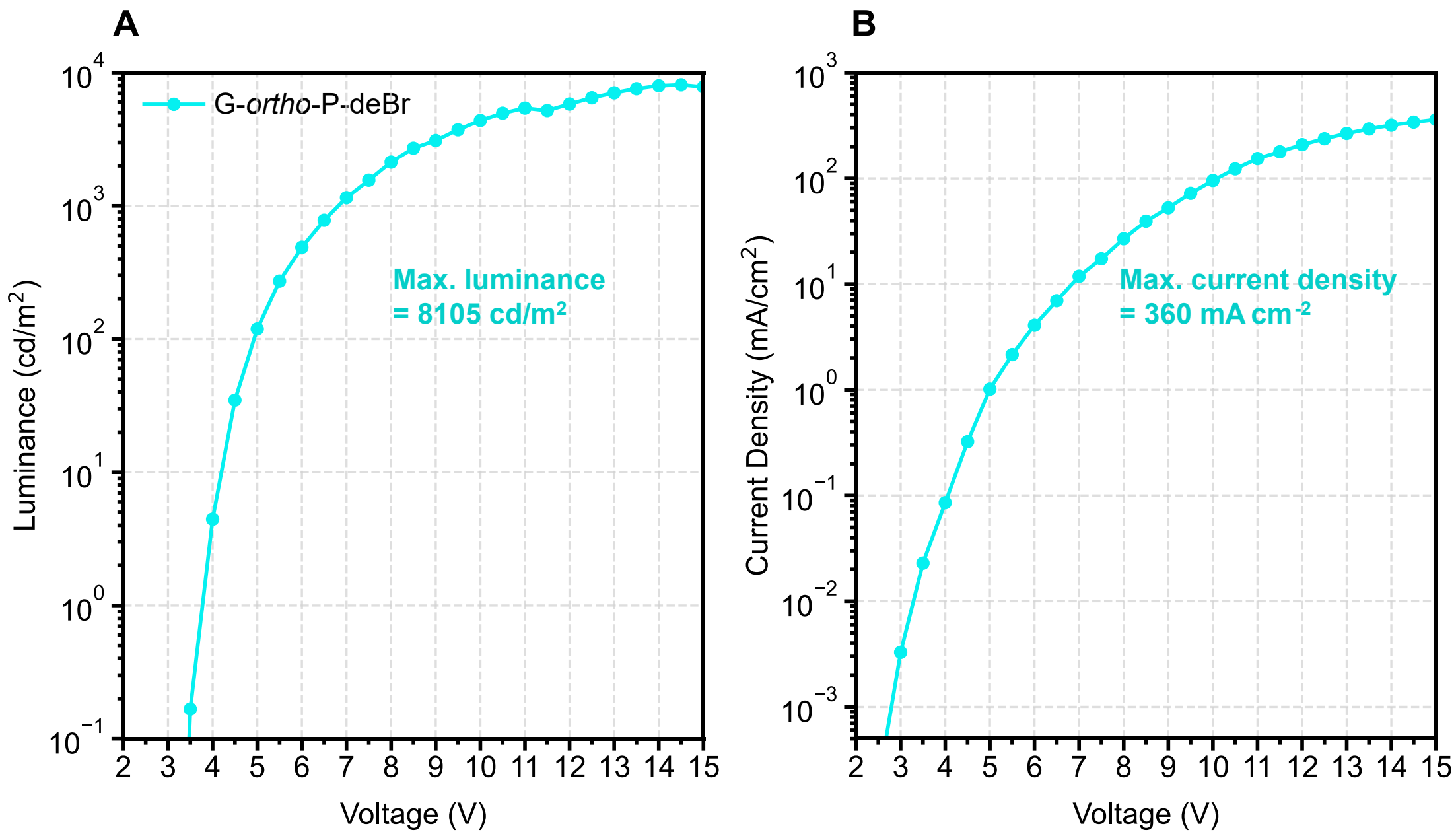

Supplementary Fig. 64: Luminance–voltage (A) and current density-voltage (B) characteristics of the OLED device employing G-*ortho*-P-deBr as EML. Device architecture: ITO/PEDOT:PSS (Al 4083)/v-NPB/G-*ortho*-P-deBr/TPBi/Liq/Al. The device can be operated to the high brightness and high current density regions with the max. luminance of 8105 cd m^{-2} and the max. current density of 360 mA cm^{-2}.

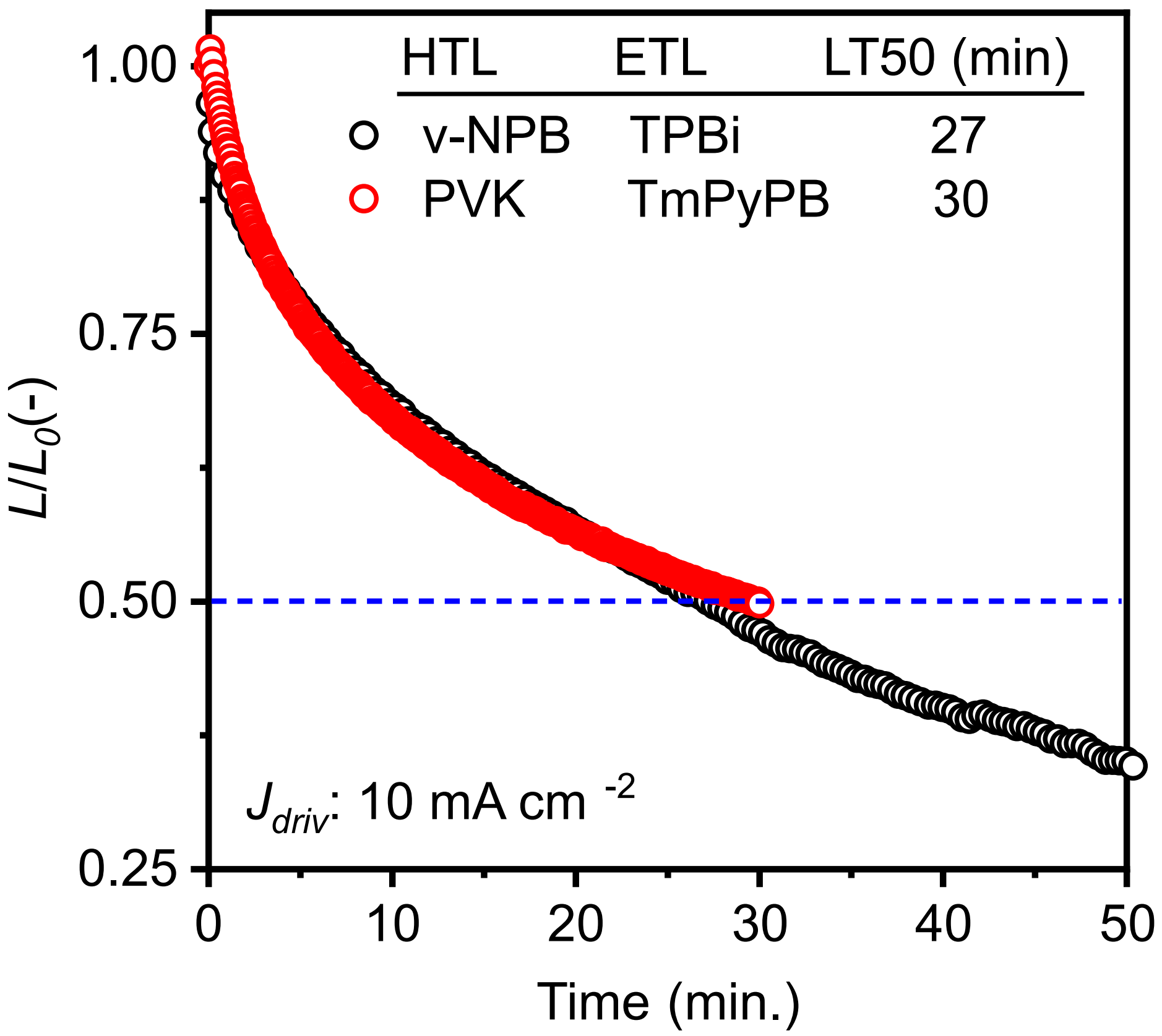

Supplementary Fig. 65: Operational stability of OLED devices consisting green-emitting self-hosted G-*ortho*-P-deBr TADF polymer operated under 10 mA cm^{-2}. Device architectures: ITO/v-NPB/G-ortho-P-deBr/TPBi/Liq/Al (black dots) and ITO/NiOx/PVK/G-ortho-P-deBr/TmPyPB/Liq/Al (red dots).

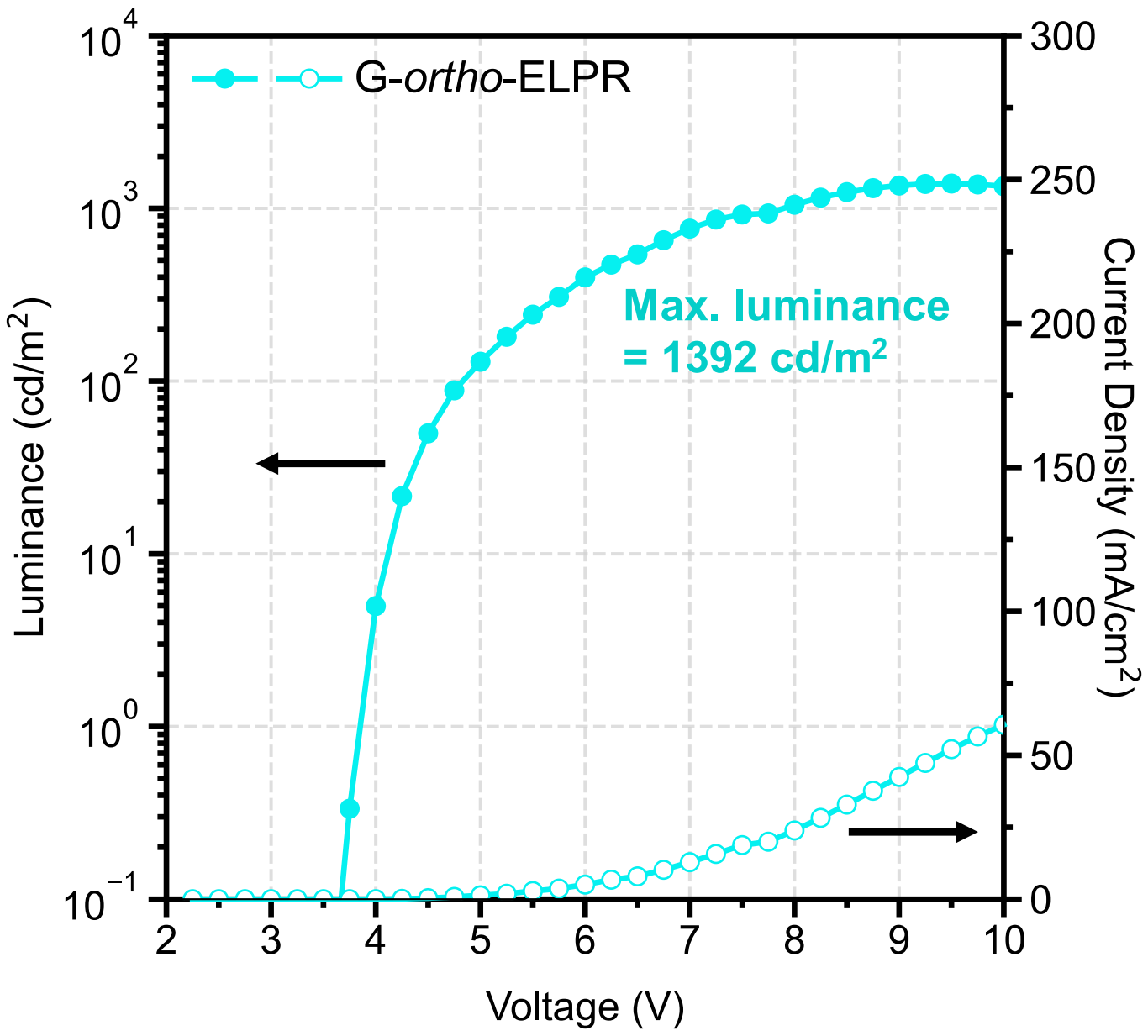

Supplementary Fig. 66: Luminance–voltage characteristic of the OLED device employing G-*ortho*-P-ELPR as EML. Device architecture: ITO/PEDOT:PSS (AI 4083)/PVK/EML/TmPyPB/Liq/Al.

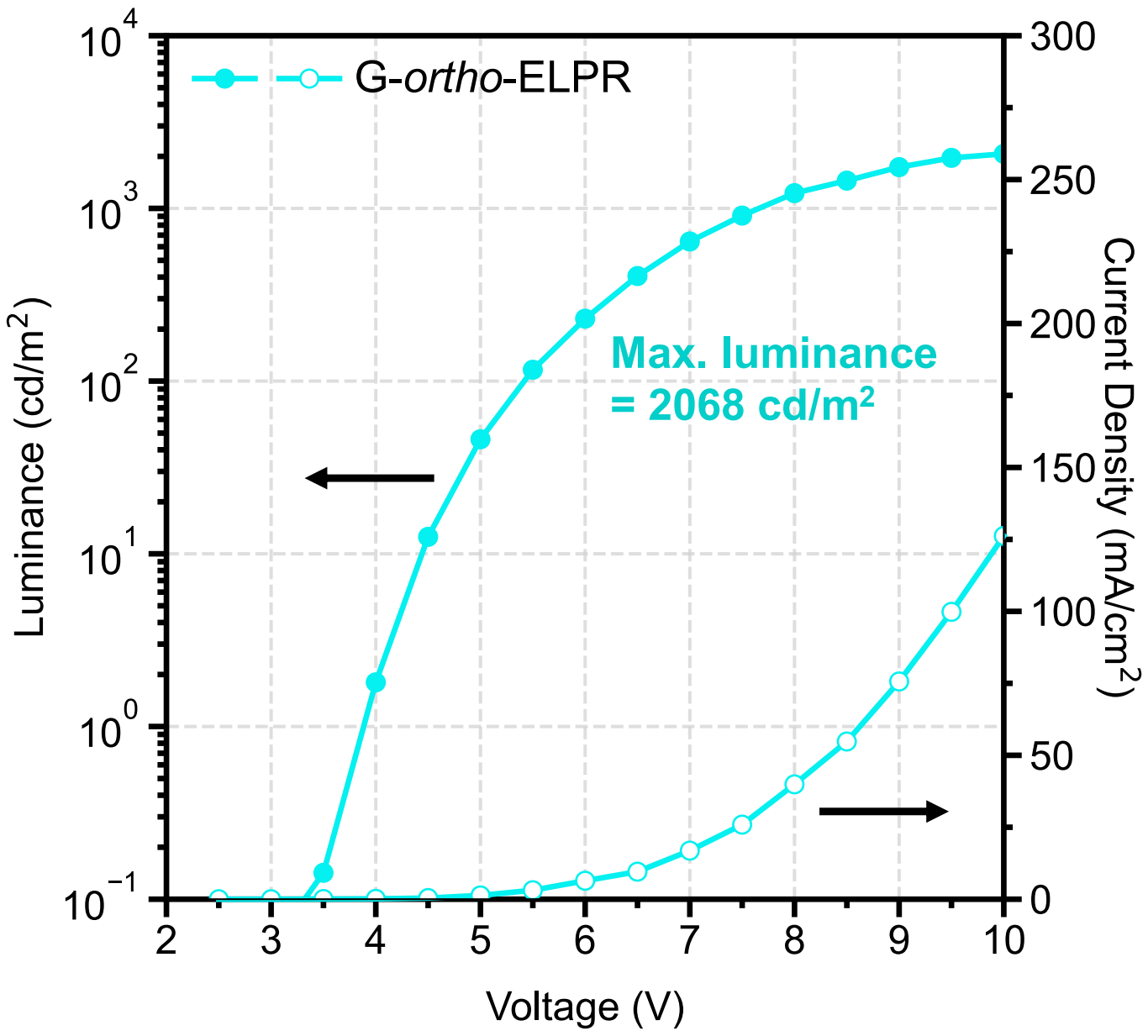

Supplementary Fig. 67: Luminance-Voltage characteristic of the OLED device employing G-*ortho*-P-ELPR as EML. Device architecture: ITO/PEDOT:PSS (AI 4083)/EML/TPBi/Liq/Al.

Supplementary Note 26 | Nyquist plots & frequency response characterization

Please see **Supplementary Figs. 68-70 and Table 10.**

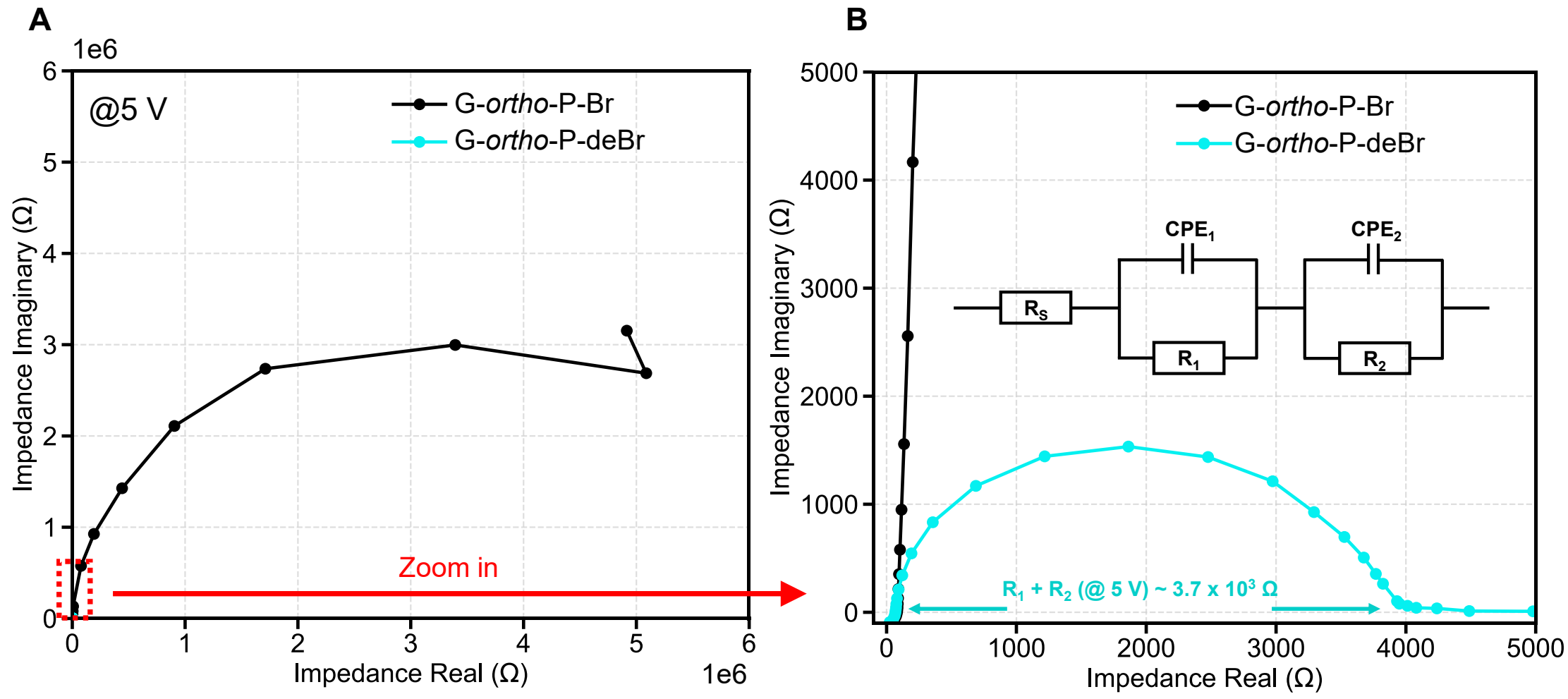

Supplementary Fig. 68: Nyquist plots of the OLED devices employing either G-*ortho*-P-Br or G-*ortho*-P-deBr as EML recorded at 5 V over 1 Hz to 1 MHz frequency range, showing the impact of end-group deactivation on charge-transport resistances. (**A**) The overview Nyquist plot. (**B**) The same Nyquist plot but zoom-in at the high frequency region. Inset of (**B**): The equivalent circuit model used to extract the charge-transport fitting parameters. The brominated device (G-*ortho*-P-Br, black) exhibits an extremely large, non-closing arc, indicative of severely hindered charge transport before end-group deactivation. In contrast, the deactivated/debrominated device (G-*ortho*-P-deBr, cyan) shows a well-defined, closed and smaller-diameter semicircle. Both the injection resistance (R_1) and recombination resistance (R_2) were significantly reduced after end group de-activation and the semicircle diameter has real impedance of $R_1 + R_2 \sim 3.7$ kΩ. Device architecture: ITO/PEDOT:PSS blend (AI 4083/CH 8000 mix)/EML/TmPyPB/Liq/Al.

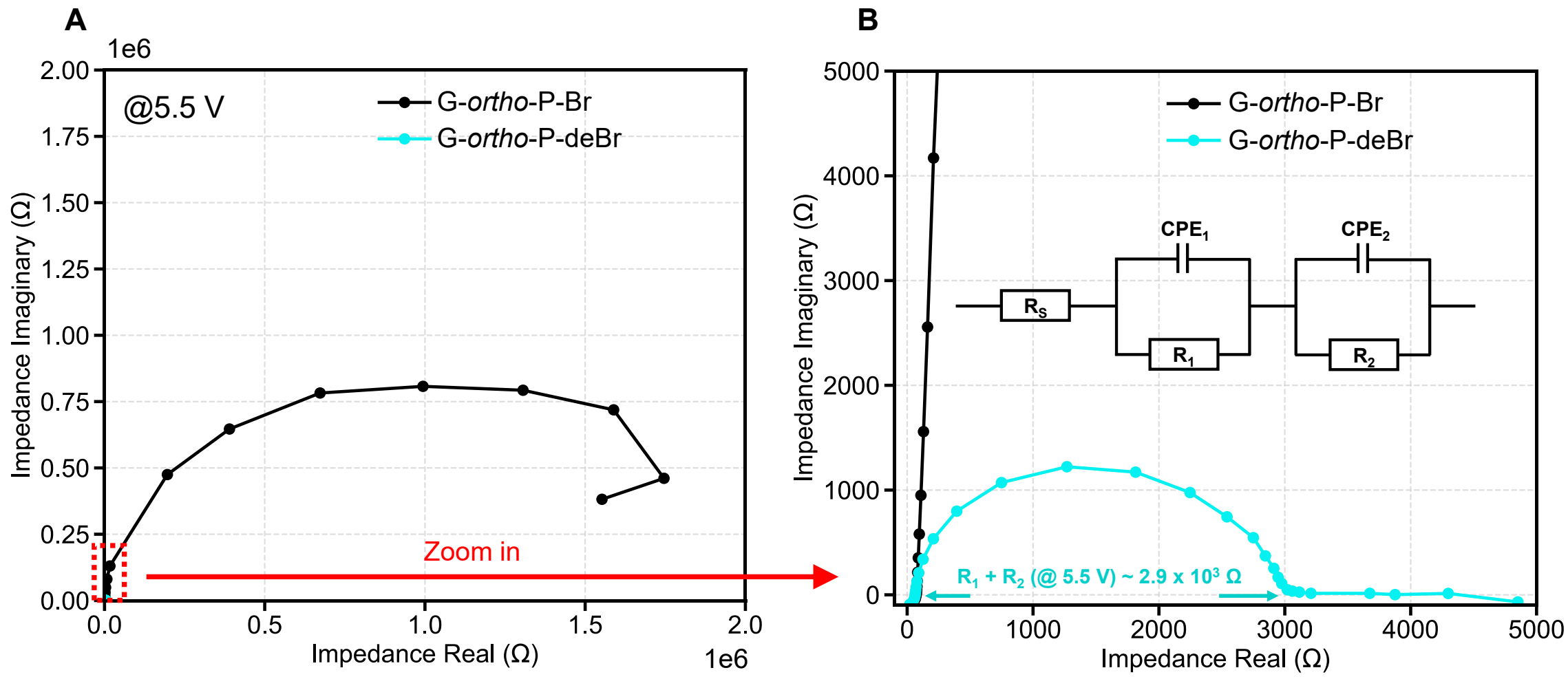

Supplementary Fig. 69: Nyquist plots of the OLED devices employing either G-*ortho*-P-Br or G-*ortho*-P-deBr as EML recorded at 5.5 V over 1 Hz to 1 MHz frequency range, showing the impact of end-group deactivation on charge-transport resistances. (**A**) The overview Nyquist plot. (**B**) The same Nyquist plot but zoom-in at the high frequency region. Inset of (**B**): The equivalent circuit model used to extract the charge-transport fitting parameters. The brominated device (G-*ortho*-P-Br, black) exhibits an extremely large, non-closing arc, indicative of severely hindered charge transport before end-group deactivation. In contrast, the deactivated/debrominated device (G-*ortho*-P-deBr, cyan) shows a well-defined, closed and smaller-diameter semicircle. Both the injection resistance (R_1) and recombination resistance (R_2) were significantly reduced after end group de-activation and the semicircle diameter has real impedance of $R_1 + R_2 \sim 2.9$ kΩ. Device architecture: ITO/PEDOT:PSS blend (AI 4083/CH 8000 mix)/EML/TmPyPB/Liq/Al.

Supplementary Table 10 | Summary of the fitting parameters of the Nyquist plots extracted from the equivalent circuit model.

Measurement condition	DC bias at 5V with 70 mV AC perturbation		DC bias at 5.5V with 70 mV AC perturbation	
**End group de-activation?** [a] **(for G-ortho-P)**	N	Y	N	Y
**R_s** [b] **(Ω)**	82.5	71.0	74.5	67.6
**R_1** [c] **(Ω)**	1.67×10^{7}	1.58×10^{3}	9.96×10^{5}	1.17×10^{3}
**CPE_1** [d] **($s^{\alpha}\ \Omega^{-1}$)**	3.83×10^{-7}	8.23×10^{-8}	1.12×10^{-8}	4.30×10^{-8}
**α_1** [e] **(-)**	0.94	0.95	0.98	0.99
**R_2** [f] **(Ω)**	5.85×10^{6}	2.17×10^{3}	1.14×10^{6}	1.68×10^{3}
**CPE_2** [g] **($s^{\alpha}\ \Omega^{-1}$)**	9.19×10^{-9}	9.18×10^{-9}	4.37×10^{-8}	8.62×10^{-9}
**A_2** [h] **(-)**	0.98	1.00	0.99	1.00

a. Y: End capping C-Br groups are debrominated. N: without end group de-activation. *b*. R_s: contact resistance. *c*. R_1: injection resistance. *d*. CPE_1: constant phase element describing the non-ideal injection capacitance. *e*. α_1: dimensionless non-ideality parameter of CPE_1; where $\alpha_1 < 1$ for non-ideal capacitor case. *f*. R_2: recombination resistance. g. CPE_2: constant phase element describing the non-ideal recombination capacitance. *h*. α_2: dimensionless non-ideality parameter of CPE_2; where $\alpha_2 < 1$ for non-ideal capacitance case.

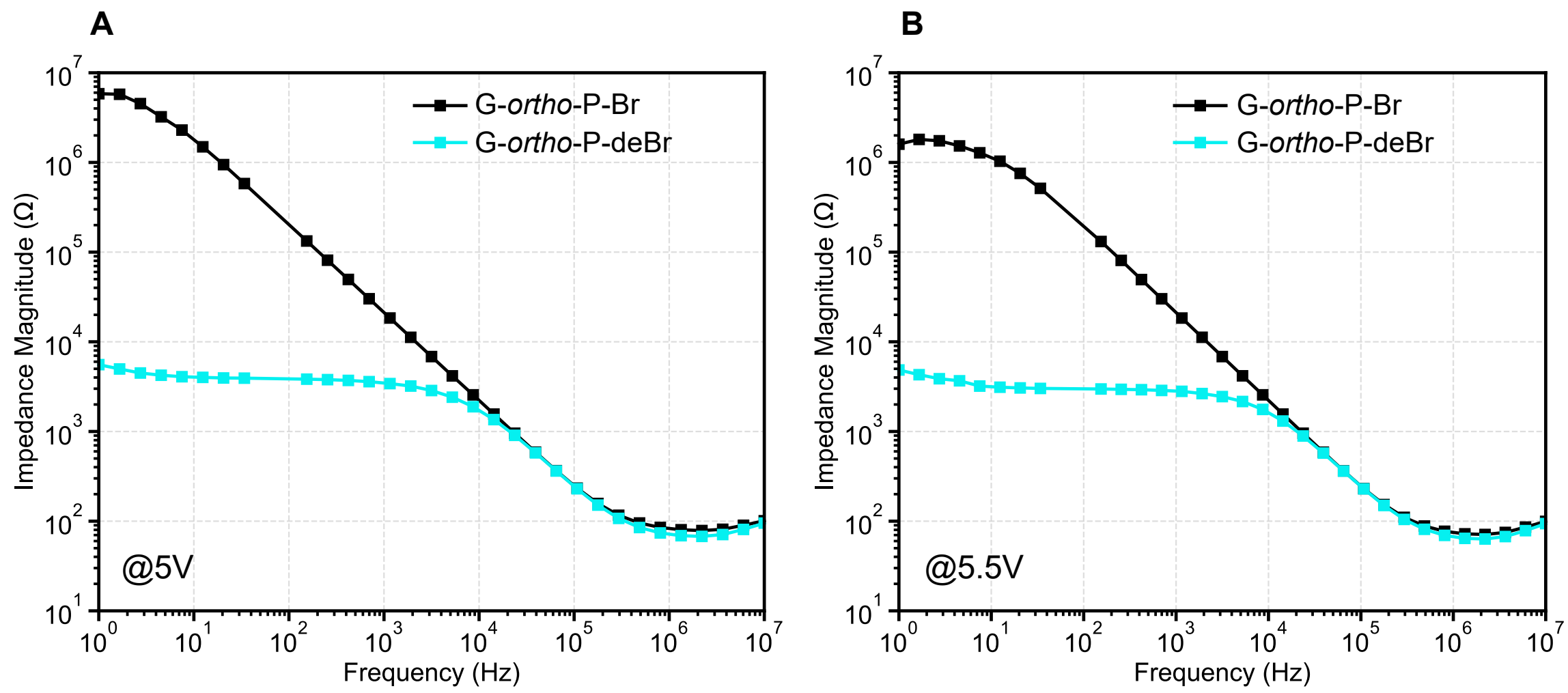

Supplementary Fig. 70: Impedance – frequency response spectra of the OLED devices employing G-*ortho*-P-Br (black squares) or G-*ortho*-P-deBr (cyan squares) as EML under forward DC bias either 5 V (**A**) or 5.5 V (**B**). The pronounced reduction of low-frequency impedance (close to 1 Hz) for G-*ortho*-P-deBr compared to G-*ortho*-P-Br case indicates more efficient charge transport after end group de-activation.

Supplementary Note 27 | Applicability of end group de-activation for red/blue emitting polymers

Please see **Supplementary Figs. 71-76 and Tables 11-12.**

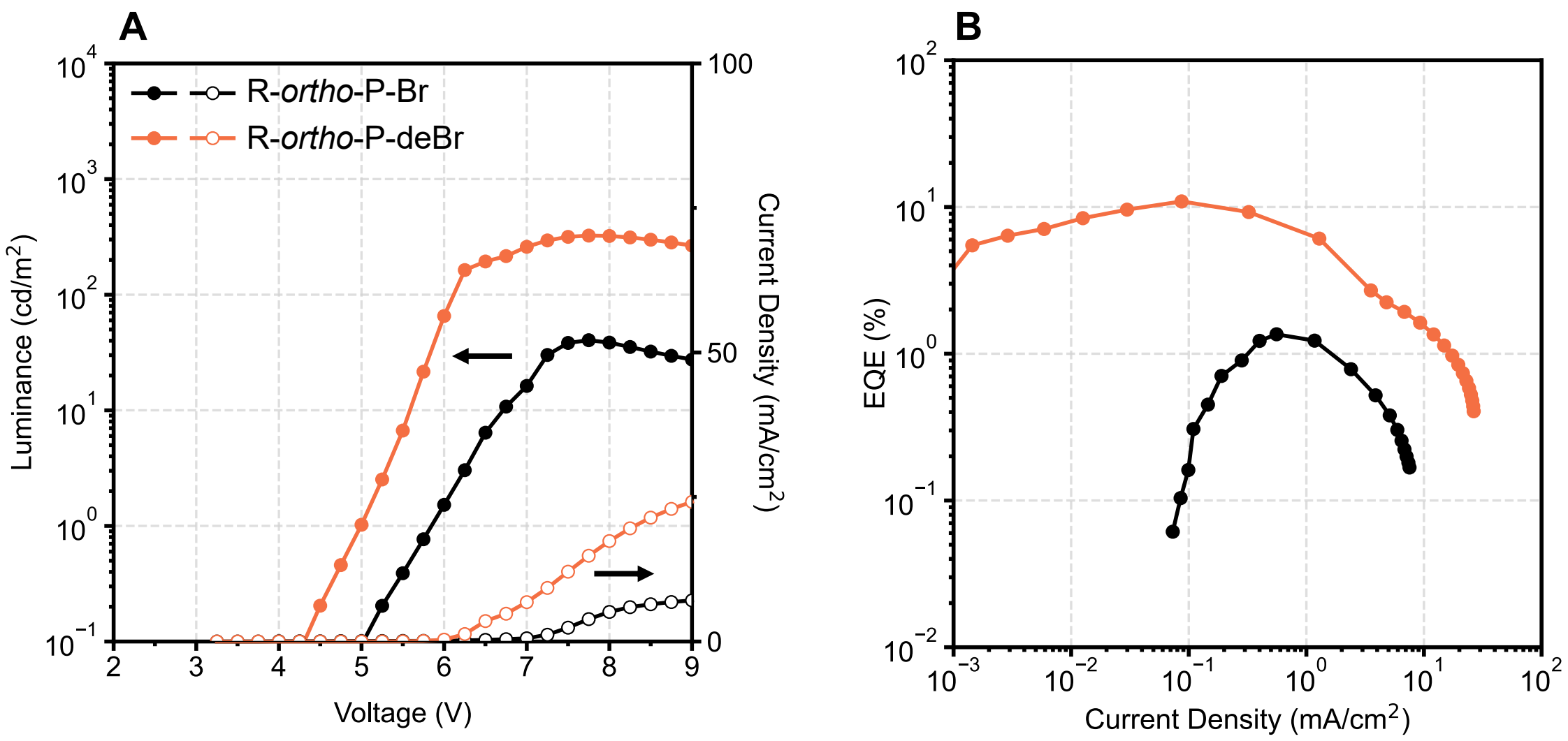

Supplementary Fig. 71: Luminance–voltage (**A**) and EQE-current density (**B**) characteristics of the OLED device employing either R-*ortho*-P-Br (black) or R-*ortho*-P-deBr (orange) as EML. Device architecture: ITO/PEDOT:PSS blend (AI 4083/CH 8000 mix)/EML/TmPyPB/Liq/Al.

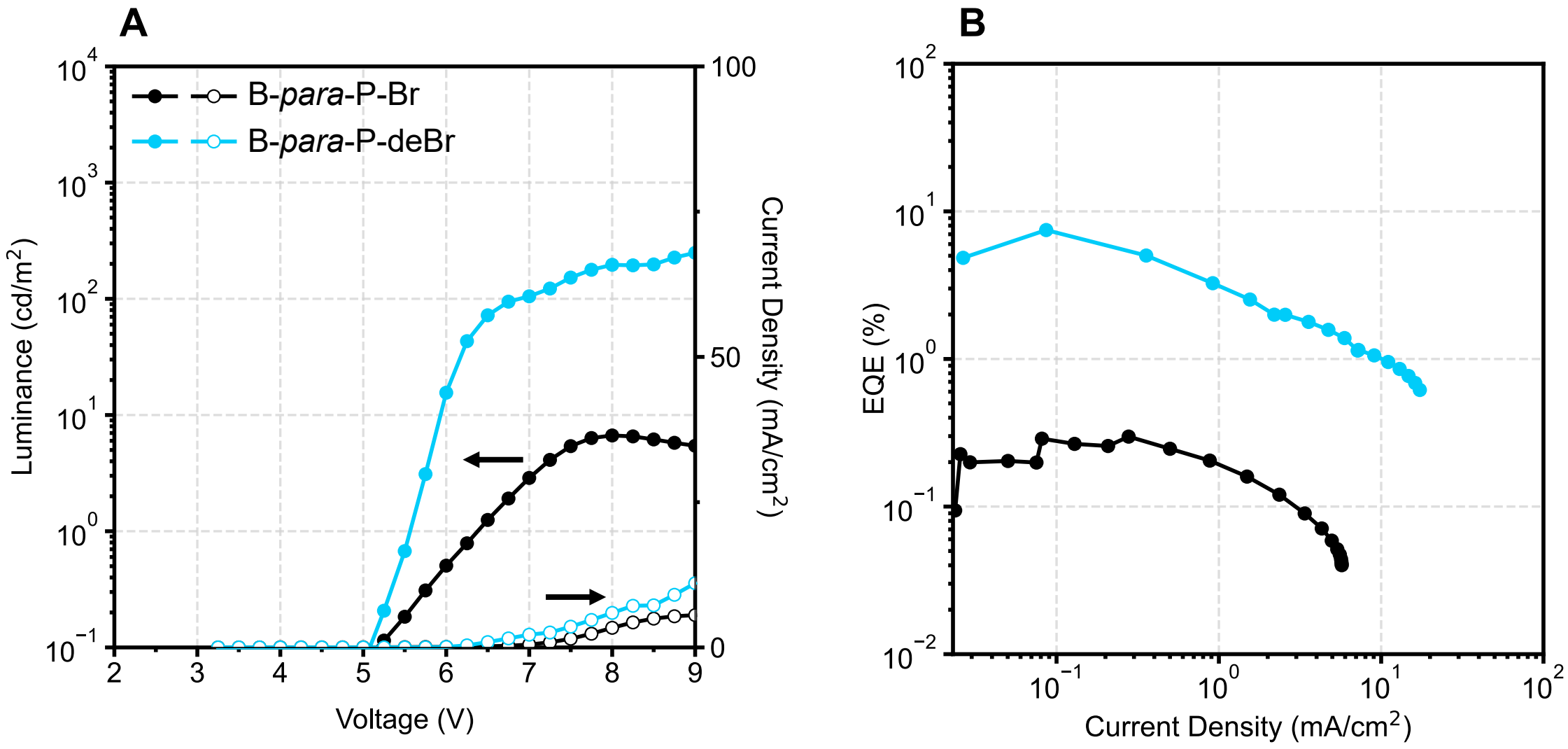

Supplementary Fig. 72: Luminance–voltage (**A**) and EQE-current density (**B**) characteristics of the OLED device employing either B-*para*-P-Br (black) or B-*para*-P-deBr (blue) as EML. Device architecture: ITO/PEDOT:PSS blend (Al 4083/CH 8000 mix)/EML/TmPyPB/Liq/Al.

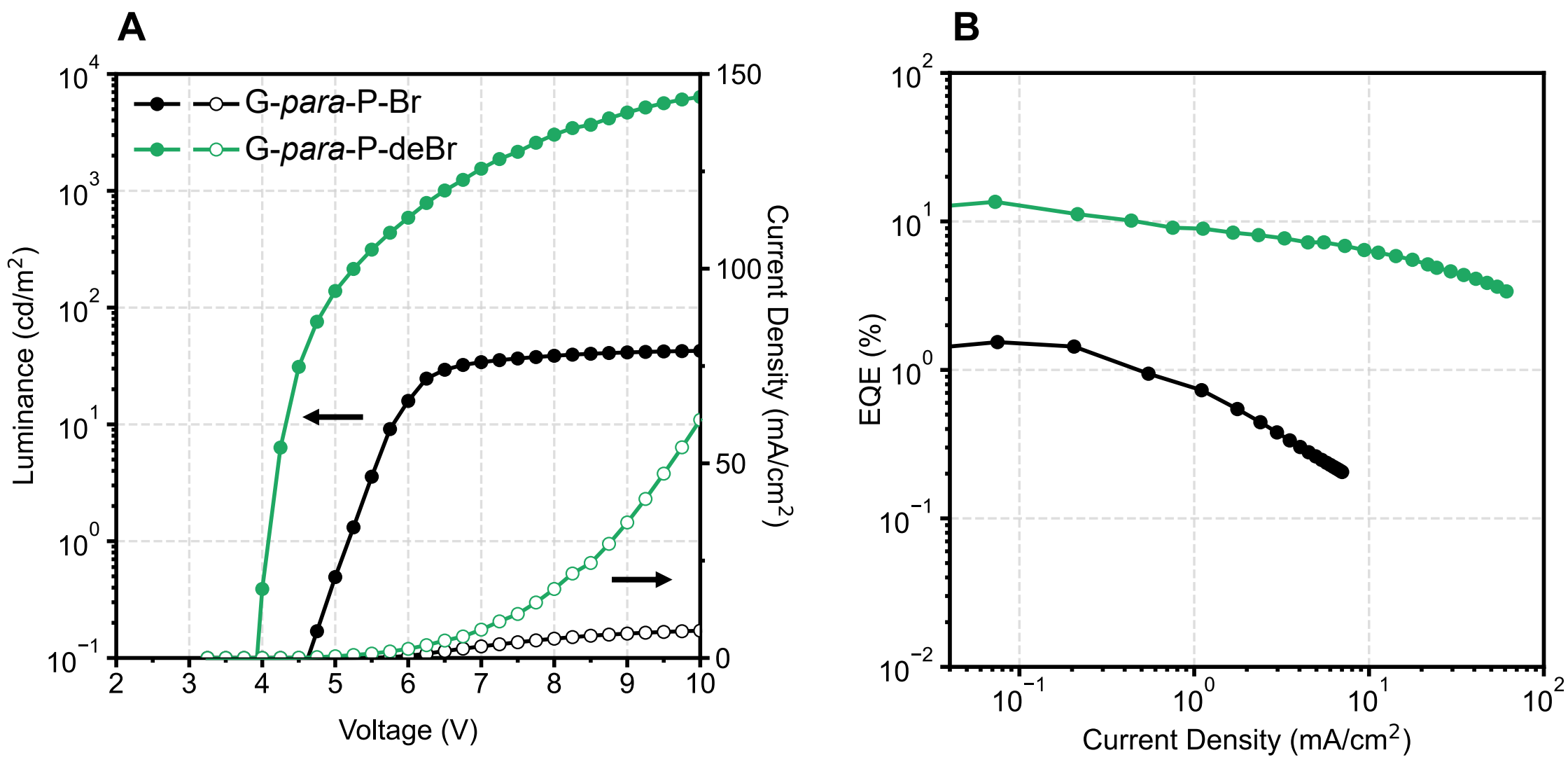

Supplementary Fig. 73: Luminance–voltage (**A**) and EQE-current density (**B**) characteristics of the OLED device employing G-*para*-P-Br (black) or G-*para*-P-deBr (green) as EML. Device architecture: ITO/PEDOT:PSS blend (Al 4083/CH 8000 mix)/EML/TmPyPB/Liq/Al.

Supplementary Table 11 | Quantitative comparison of pixelation strategies for emissive display technologies, including inorganic QD-LEDs, III-V micro-LEDs, perovskite LEDs, and ELPR OLEDs.

LEDs	Pixelization process	Process compatibility	Feature size	Max. *L* (cd/m^2)	$EQE_{max.}$ (%)	FWHM (nm)	Device operational stability	Reference
Inorganic QD-LEDs	Direct photolithography	Solvent orthogonal & Scalable	1 µm	7851	25.6	20~30	LT_{95}:*135 hrs*	Nat. comm. (2025)[4]
	Direct photolithography	Solvent orthogonal & Scalable	0.8 µm	$>10^5$	< 20	20~40	LT_{95}:*~55 hrs*	Nat. Nanotechnol. (2022)[5]
	Transfer printing	Dry process & transfer yield issue	400 nm	$\sim10^5$	23.3	20~30	-	Nat. photonics (2024)[6]
	Lift-off photolithography	Solvent orthogonal & Scalable	0.8 µm	$< 10^5$	16.6	20~30	-	Nat. comm. (2026)[7]
III-V micro-LEDs	Ion implantation pixelization	Suitable for single-color pixelization	0.5 µm	55835	12	4.8	LT_{95}: *> 300 hrs*	Nat. photonics (2021)[8]
	Fluidic assembly	Suitable for sub-100 micron pixelization	45 µm	-	-	25~30	-	Nature (2023)[9]
	Layer transfer + photolithography	Scalability limited to epitaxial substrate size	4 µm	$<10^5$	< 0.5	25~45	-	Nature (2023)[10]
Perovskite LEDs	Direct photolithography	Solvent orthogonal & Scalable	5 µm	> 20000	6.8	24	LT_{50}:*5~7* mins	Sci. Adv. (2022)[11]
	Transfer printing	Dry process & transfer yield issue	400 nm	4159	14.8	18	LT_{50}:*22* mins	Sci. Adv. (2022)[12]
	Inkjet printing	Feature size limited to nozzle/drop size	~100 µm	43883	8.54	20	LT_{50}:*64* mins	Adv. Mater. (2022)[13]
**ELPR OLEDs**	**Direct photolithography E-beam lithography**	**Solvent orthogonal & Scalable**	**110 nm**	**2068**	**13.3**	**85~100**	LT_{50}:***172 mins***	**This work**

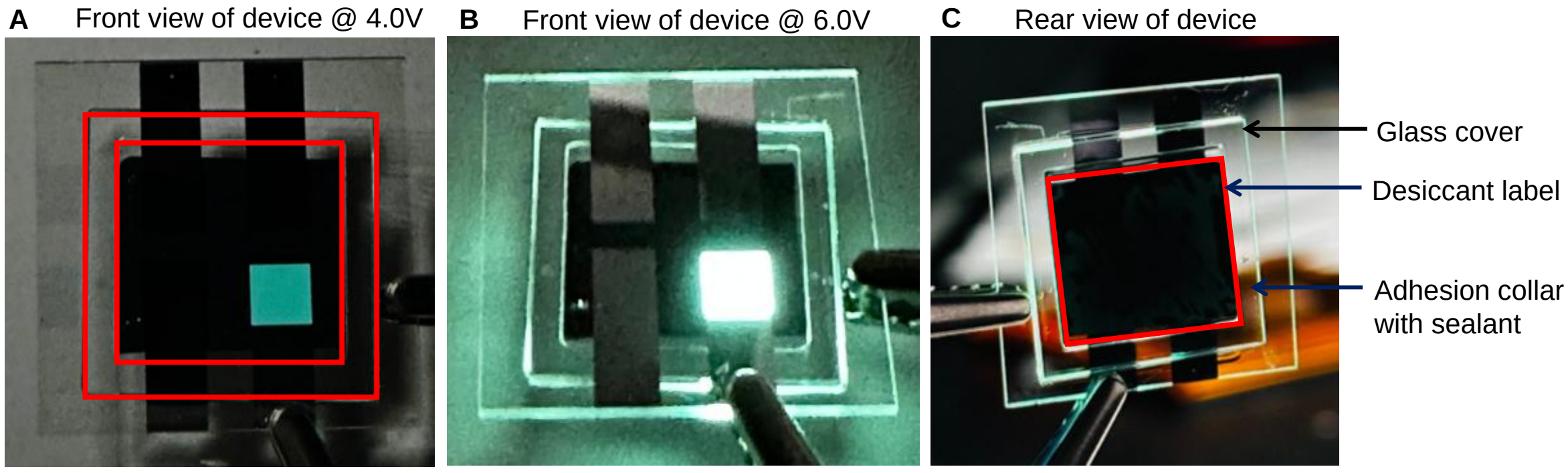

Supplementary Fig. 74: Photographs of encapsulated OLED devices used for the operation lifetime (LT_{50}) measurement. (A) Front view of device driving at 4.0V, (B) Front view of device driving at 6.0V, and (C) Rear view of device driving at 6.0V.

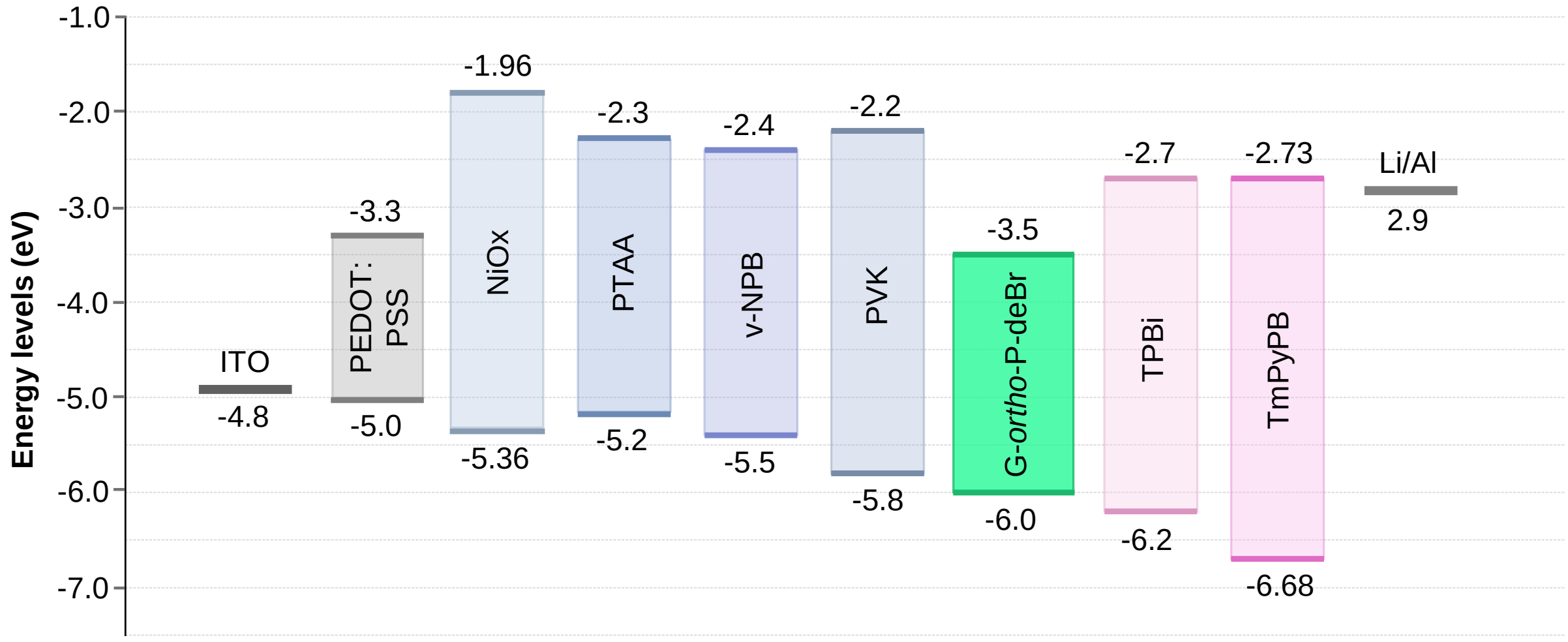

Supplementary Fig. 75: Combined device architecture and energy-level diagram, showing the HOMO and LUMO levels of all transport and injection layers studied during device optimization. G-*ortho*-P-deBr is employed as EML, where the of the various transporting materials studied during the optimization process were displayed. Values indicate the HOMO and LUMO energies in eV relative to the vacuum level.[17-22]

Supplementary Table 12 |. Temperature dependent TRPL lifetimes of green-emitting self-hosted TADF polymer after (G-*ortho*-P-deBr) end group de-activation with DP_{ACz} = 45 in thin fim.

Temperature (K)	τ_{PF} (*ns*)	τ_{DF} (*μs*)	A1	A2
298	27.68	1.52	4503.28	85.31
280	30.82	1.88	3803.97	79.77
260	30.76	2.09	4073.55	75.21
240	27.57	2.35	8911.89	47.61
220	29.37	2.39	4145.12	53.51
200	29.45	2.36	4714.49	42.45
150	29.31	2.20	9222.21	28.27
100	28.45	1.23	10784.7	21.00
77	29.06	0.90	4865.65	19.25

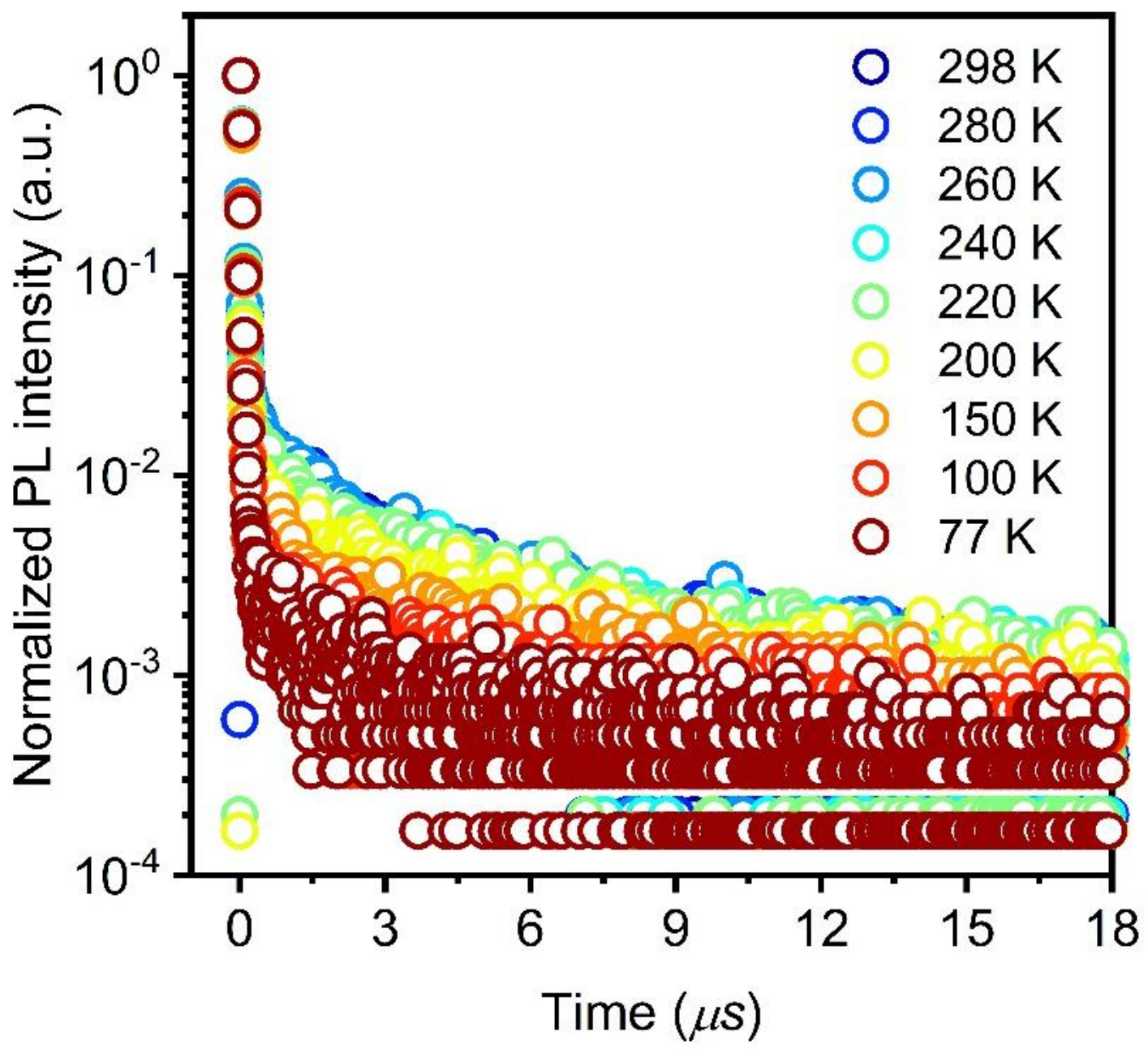

Supplementary Fig. 76: Thin film TRPL spectra of green-emitting self-hosted TADF polymer after (G-*ortho*-P-deBr) end group de-activation with DP_{ACz} = 45 measured at various temperature from 298 to 77 K. First, delay fluorescence lifetime component increased from 298 to 220K, which is typical characteristics of TADF phenomena.

References

1. Tsai, W.-L. et al. A versatile thermally activated delayed fluorescence emitter for both highly efficient doped and non-doped organic light emitting devices. Chem. Commun. **51**, 13662–13665 (2015).
2. Jiang, R. et al. High-Performance Orange–Red Organic Light-Emitting Diodes with External Quantum Efficiencies Reaching 33.5% based on Carbonyl-Containing Delayed Fluorescence Molecules. Adv. Sci. **9**, 2104435 (2022).
3. Pan, L. et al. Hypercrosslinked porous polycarbazoles via one-step oxidative coupling reaction and Friedel–Crafts alkylation. Polym. Chem. **6**, 2478–2487 (2015).
4. Guan, J. et al. Direct ambient photopatterning of RGB quantum dots for light-emitting diodes with EQE exceeding 20%. Nat. Commun. **16**, 9689 (2025).
5. Hahm, D. et al. Direct patterning of colloidal quantum dots with adaptable dual-ligand surface. Nat. Nanotechnol. **17**, 952–958 (2022).
6. Yoo, J. et al. Highly efficient printed quantum dot light-emitting diodes through ultrahigh-definition double-layer transfer printing. Nat. Photonics **18**, 1105–1112 (2024).
7. Kim, H. et al. Photoresist-guided indirect photopatterning of quantum dots via carbene-mediated ligand thermocrosslinking. Nat. Commun. **17**, 4162 (2026).
8. Park, J. et al. Electrically driven mid-submicrometre pixelation of InGaN micro-light-emitting diode displays for augmented-reality glasses. Nat. Photonics **15**, 449–455 (2021).
9. Lee, D. et al. Fluidic self-assembly for MicroLED displays by controlled viscosity. Nature **619**, 755–760 (2023).
10. Shin, J. et al. Vertical full-colour micro-LEDs via 2D materials-based layer transfer. Nature **614**, 81–87 (2023).
11. Liu, D. et al. Direct optical patterning of perovskite nanocrystals with ligand cross-linkers. Sci. Adv. **8**, eabm8433 (2022).
12. Kwon, J. I. et al. Ultrahigh-resolution full-color perovskite nanocrystal patterning for ultrathin skin-attachable displays. Sci. Adv. **8**, eadd0697 (2022).

13. Wei, C. et al. A universal ternary-solvent-ink strategy toward efficient inkjet-printed perovskite quantum dot light-emitting diodes. Adv. Mater. **34**, 2107798 (2022).

14. Serevičius, T. *et al.* TADF parameters in the solid state: an easy way to draw wrong conclusions. *J. Phys. Chem. A* **125**, 1637–1641 (2021).

15. Goushi, K., Yoshida, K., Sato, K. et al. Organic light-emitting diodes employing efficient reverse intersystem crossing for triplet-to-singlet state conversion. Nat. Photonics **6**, 253-258 (2012).

16. Masui, K., Nakanotani, H. & Adachi, C. Analysis of exciton annihilation in high-efficiency sky-blue organic light-emitting diodes with thermally activated delayed fluorescence. *Org. Electron.* **14**, 2721-2726 (2013).

17. Jou, J. H. Kumar, S. Agrawal, A. Li, T. H. & Sahoo, S. Approaches for fabricating high efficiency organic light emitting diodes. *J. Mater. Chem. C*, **3**, 2974-3002 (2015).

18. Qin, J. *et al.* Surfactant-induced hole concentration enhancement for highly efficient perovskite light-emitting diodes. *Nat. Mater.* **24**, 778–784 (2025).

19. Wang, X. *et al.* Co-self-assembled monolayers modified NiO_x/perovskite interface for high-performance wide bandgap perovskite solar cells. *ACS Appl. Nano Mater.* **8** (15) 7787-7795 (2025).

20. Li, Y. et al. Modulation of charge transport layer for perovskite light-emitting diodes. *Adv. Mater.* **37**, 2410535 (2025).

21. Li, W. Zhang, X. Zhang, X. Yao, J. Zhan, C. High-performance solution-processed single-junction polymer solar cell achievable by post-treatment of PEDOT: PSS layer with water-containing methanol, *ACS Appl. Mater. Interfaces* **9** (2) 1446-1452 (2017).

22. Kim, D.H. *et al.* Thermally cross-linkable blended hole transport layer for solution-processed quantum dot light-emitting diodes, *ACS Appl. Nano Mater.* **8**(34) 16727-16735 (2025).
