# Supplementary material for: Electroluminescent photoresists extending lithographic scaling to OLEDs
Source: Nature. 2026 Sep 16;658(8134):78–85. doi: 10.1038/s41586-026-11042-0 (PMC13626961; doi:10.1038/s41586-026-11042-0)
Supplement: Supplementary file 2 [file 41586_2026_11042_MOESM2_ESM.pdf]

nature portfolio

Peer Review File

Electroluminescent Photoresists Extending Lithographic Scaling to OLEDs

Corresponding Author: Professor Chih-Jen Shih

Any redactions in this file are there to maintain patient confidentiality, the confidentiality of unpublished data, or to remove third-party material.

This file contains all reviewer reports in order by version, followed by all author rebuttals in order by version.

Attachments originally included by the reviewers as part of their assessment can be found at the end of this file.

Version 0:

Reviewer comments:

Referee #1

(Remarks to the Author)
This manuscript addresses a key technological bottleneck for next-generation ultra-high-resolution displays and integrated photonics: scalable miniaturization/patterning of OLEDs. The authors develop electroluminescent photoresists (ELPRs) that enable direct UV/EBL patterning while retaining high EL, by adopting a core–shell architecture in which the TADF emitter is protected by a host shell and spatially separated from reactive photo-crosslinking sites. The demonstrated fluorescent nanopatterns down to 110 nm and UV-patterned full-color OLEDs with >13% EQE are compelling and suggest a broadly applicable functional photoresist (FPR) platform. Overall, the work is original and timely and can be suitable for Nature. However, I believe the following revisions are necessary to strengthen the mechanistic conclusions and the reliability of the device claims.
1.Incomplete photophysical characterization as TADF polymers (need kr and kRISC).
While prompt/delayed lifetimes and PLQY are reported for each polymer, key kinetic parameters intrinsic to TADF—most importantly the radiative rate constant (kr) and the reverse intersystem crossing rate (kRISC)—are not evaluated. Without these parameters, the structure–property relationship (e.g., core–shell design, separation from crosslinking sites, compositional variations) remains insufficiently supported. Please extract at least kr and kRISC (and ideally other rates such as non-radiative channels) using a standard kinetic model. Also, temperature-dependent PL measurements (lifetime/PLQY) help in understanding the nonradiative decay processes. This will substantially improve the usefulness and generality of the work.
2.Reliability/reproducibility of EQE at low luminance (<100 cd m^{-2}).
The EQE–luminance data appear reasonably stable above 100 cd m^{-2}, but below this regime, the number of data points is limited, and the behavior looks unstable. In particular, the ~30% EQE in Fig. 5 is a key result; however, the apparent double-peak feature raises concerns about measurement artifacts or device integrity issues (e.g., pinholes/leakage/local shorts, scan-history dependence). Please repeat J–V–L/EQE measurements with multiple devices (statistics) and scan-direction / repeated scans, and provide evidence of reproducibility. If possible, add supporting diagnostics related to leakage/defects (e.g., microscopy, yield, uniformity, leakage current analysis). This will ensure the reported high EQE is robust and not an artifact. PS: EQE-current density characteristics are more fundamental than the EQE-Luminance.

Referee #2

(Remarks to the Author)
The authors address a longstanding challenge in OLED nano-patterning by integrating TADF emitters with photoresist chemistry to directly pattern RGB subpixels. The demonstration of photolithographic patterning of functional OLED emitters represents a novel and technically ambitious approach, and this work offers a meaningful conceptual advance over conventional patterning strategies that have been considered difficult for OLED materials.
However, the technology appears to face significant performance limitations relative to conventional OLED patterning methods. It would be valuable for the authors to more clearly discuss whether ELPR-based OLEDs are realistically compatible with practical display requirements, and to outline how further material or device development could enable

meaningful competition with existing commercial technologies.
Major comments
1. The operational lifetime of the ELPR OLEDs reported in this work is substantially shorter than that reported for many OLED and TADF-based systems in the recent literature. The manuscript attributes the limited lifetime mainly to extrinsic factors such as HTL choice, residual copper, and polymer impurities. However, it remains unclear whether the observed lifetime limitation arises primarily from insufficient materials and process optimization, or from intrinsic constraints imposed by the ELPR molecular and polymer architecture, which is designed to simultaneously support electroluminescence and photolithography.
While the authors attribute the present lifetime limitations primarily to extrinsic factors, the manuscript provides limited evidence to substantiate this interpretation. It remains unclear to what extent these factors dominate the degradation behavior relative to the intrinsic stability of the ELPR materials. Additional experimental data would help clarify the relative contributions of material, process, and device-level effects, and would strengthen the authors' argument regarding the origin of the lifetime limitations.
Also, based on the current materials system, what level of lifetime improvement do the authors consider realistically achievable?
2. The manuscript refers to "full-color" electroluminescence based on the demonstrated RGB ELPR devices. However, the reported EL peaks (approximately 493 nm for blue and 586 nm for red) appear significantly shifted from standard display blue and red primaries. It is therefore unclear whether the present emission characteristics alone justify the use of the term "full color." In addition, the EL spectra shown in the manuscript appear relatively broad, suggesting limited color purity.
Can the authors provide the CIE 1931 (x, y) color coordinates? How do these values compare with commonly accepted display requirements for color purity and gamut coverage?
Furthermore, the emission wavelengths appear to be largely determined by the choice of the emitter core. The authors are encouraged to clarify the rationale behind selecting the current blue and red emitter cores, and to explain why deeper-blue and deeper-red emitters, which are typically required for display-grade color standards, were not explored in this study. In addition, it would be helpful if the authors could discuss the origin of the relatively broad emission bandwidths observed in the EL spectra. Are these linewidths intrinsic to the ELPR material design or processing conditions? A discussion of the spectral broadening mechanisms and potential pathways to improve color purity would significantly strengthen the manuscript.
3. Based on the data presented, the high-brightness operating capability of the ELPR OLEDs does not appear to be experimentally demonstrated. While electroluminescence and uniform emission are shown, the manuscript does not provide clear evidence that the devices can sustain the high current densities required for brightness levels relevant to practical OLED or micro-display applications.
Can the authors clarify whether the present ELPR material architecture is fundamentally capable of stable operation in the high-current, high-brightness regime? The current manuscript does not appear to provide sufficient experimental evidence demonstrating that brightness can be substantially improved through process or device optimization.
4. The J–V and L–V characteristics indicate that current density and luminance do not continue to increase at higher driving voltages, and in some regimes appear to saturate or decrease. The manuscript does not explicitly address the physical origin of this behavior.
Can the authors clarify whether this high-voltage response is associated with irreversible current-induced degradation, charge imbalance and transport limitations, or other exciton-related quenching processes in the ELPR OLEDs? Importantly, do the authors consider this behavior to be an intrinsic limitation of the ELPR material architecture under high current injection, or a reversible effect that can be mitigated through device or materials optimization.
5. End-group deactivation leads to a dramatic improvement in device efficiency and current efficiency, while the emissive core structure remains unchanged. This suggests that the performance gain may arise primarily from reduced trap-assisted losses or improved film quality rather than from intrinsic changes to the emitter.
Can the authors clarify to what extent end-group deactivation also improves tolerance to high current injection, high-brightness operation, and operational stability? Is there experimental evidence that deactivation mitigates current-induced degradation or charge-transport limitations, beyond the observed efficiency enhancement at low to moderate operating conditions.
In addition, while the deactivation effect is prominently demonstrated for the green emitter system, it is less clear how consistently this improvement translates to the red and blue ELPRs. Can the authors clarify whether similar trap-related performance enhancement is observed across all RGB systems, and if not, what governs the color-dependent response to end-group deactivation?
6. Supplementary Fig. 44 shows a strong PLQY disparity among RGB, with green being much higher than red and blue both for self-hosted polymers and for ELPRs after crosslinking. What is your mechanistic explanation for this RGB dependence? Do you think the limitation is mainly intrinsic to the emitter cores, or is it dominated by the polymer or crosslinker environment? What concrete molecular or processing strategies could raise the blue and red PLQYs while retaining photo-patternability?
7. The manuscript would benefit from more explicitly positioning ELPRs relative to conventional OLED patterning approaches, as well as quantum-dot photoresists (QD-PR), micro-LEDs, and perovskite LED technologies. A quantitative comparison across key performance metrics such as patterning resolution, process compatibility, peak luminance, efficiency, spectral purity, and operational stability would help clarify the practical differentiation and potential application space of ELPRs.
Minor comments
1. Please clarify the measurement conditions used for lifetime characterization, including initial luminance, driving mode (constant current or constant voltage), encapsulation conditions, and measurement environment. Including these details in the figure captions would improve clarity and reproducibility.
2. Please provide additional details on PLQY measurements, including film thickness, excitation wavelength, and integrating sphere configuration. This information would facilitate comparison across RGB systems.

3. Including an energy-level diagram of the full device stack (HOMO/LUMO levels with references) would help clarify charge injection and balance, particularly in relation to the reported efficiency improvement after end-group deactivation.

Referee #3

(Remarks to the Author)
I co-reviewed this manuscript with one of the reviewers who provided the listed reports.

Version 1:

Reviewer comments:

Referee #1

(Remarks to the Author)
The authors have satisfactorily addressed all of my major concerns.

The additional photophysical characterization, including extraction of the TADF kinetic parameters and temperature-dependent TRPL analysis, substantially strengthens the mechanistic discussion.

Furthermore, the extensive reproducibility studies, including statistical device analysis, repeated scan measurements, hysteresis tests, leakage-current analysis, EL microscopy, and AFM characterization, convincingly demonstrate that the reported device performance is robust.

I have no further major concerns and recommend acceptance after editorial revision.

Referee #2

(Remarks to the Author)
The authors have made substantial efforts to address my previous comments and have provided extensive additional experimental data. The newly added purification studies, device-stack optimization, high-current characterization, and technology comparison significantly strengthen the manuscript. Overall, most of my previous concerns have been adequately addressed. I have only a few remaining comments that may help further clarify the interpretation and presentation of the results.
1. Interpretation of the operational lifetime limitation
The additional ICP-OES and purification experiments provide useful evidence that residual copper contributes significantly to the observed operational lifetime limitation. In particular, the reduction in residual copper from 27 to 14 ppm and the corresponding improvement in LT50 after dialysis clearly support the influence of metallic impurities on device stability. Similarly, the lifetime improvement achieved through transport-layer optimization suggests that charge balance and interfacial charge accumulation also play important roles in the degradation behavior. These additional results substantially strengthen the discussion of extrinsic factors affecting the operational stability of the devices.
At the same time, it may be helpful to slightly moderate the interpretation that the current stability bottleneck is primarily governed by these extrinsic factors rather than intrinsic aspects of the ELPR molecular or polymer architecture. The present results clearly demonstrate that purification and device-stack optimization can improve operational lifetime. However, such improvements establish the significant contribution of extrinsic degradation pathways rather than directly excluding possible intrinsic stability limitations of the ELPR system. In particular, the relative contributions of the dual-functional polymer architecture, crosslinkable groups, and other material-intrinsic degradation mechanisms have not yet been independently evaluated. Therefore, statements suggesting that the current stability metrics are governed by process-level cleanliness “rather than an intrinsic thermodynamic ceiling,” or that the stability bottleneck arises from extrinsic factors “rather than an intrinsic design flaw,” may be somewhat stronger than can be concluded from the current data. I suggest slightly moderating these statements to reflect that extrinsic factors have been experimentally demonstrated to contribute significantly to the present lifetime limitation, while the possible contribution of intrinsic degradation mechanisms remains to be further clarified.
2. Peak high-current operation and continuous operation
The newly added high-luminance data clearly demonstrate that current density and peak luminance can be substantially improved through device-stack optimization. These results address my previous concern regarding the potential for improving device brightness.
It would nevertheless be helpful to more clearly distinguish between peak high-current operation and stable continuous operation. The J–V–L measurements demonstrate operation at current densities of approximately 126–360 mA cm^{-2}, whereas the continuous lifetime measurements are performed up to 10 mA cm^{-2}. I suggest clarifying this distinction when discussing stable high-current operation and Joule-heating- or breakdown-related limitations.

Overall, the revised manuscript has been significantly strengthened, and the authors have addressed the major concerns raised in my previous review. The remaining comments are primarily intended to improve the clarity and precision of the interpretation of the newly added results. I would support publication after these points are appropriately clarified.

Referee #3

(Remarks to the Author)
I co-reviewed this manuscript with one of the reviewers who provided the listed reports.

Open Access This Peer Review File is licensed under a Creative Commons Attribution 4.0 International License, which permits use, sharing, adaptation, distribution and reproduction in any medium or format, as long as you give appropriate credit to the original author(s) and the source, provide a link to the Creative Commons license, and indicate if changes were made.
In cases where reviewers are anonymous, credit should be given to 'Anonymous Referee' and the source.
The images or other third party material in this Peer Review File are included in the article's Creative Commons license, unless indicated otherwise in a credit line to the material. If material is not included in the article's Creative Commons license and your intended use is not permitted by statutory regulation or exceeds the permitted use, you will need to obtain permission directly from the copyright holder.
To view a copy of this license, visit https://creativecommons.org/licenses/by/4.0/

Response to Reviewers' comments

<u>Referee #1</u>

This manuscript addresses a key technological bottleneck for next-generation ultra-high-resolution displays and integrated photonics: scalable miniaturization/patterning of OLEDs. The authors develop electroluminescent photoresists (ELPRs) that enable direct UV/EBL patterning while retaining high EL, by adopting a core–shell architecture in which the TADF emitter is protected by a host shell and spatially separated from reactive photo-crosslinking sites. The demonstrated fluorescent nanopatterns down to 110 nm and UV-patterned full-color OLEDs with >13% EQE are compelling and suggest a broadly applicable functional photoresist (FPR) platform. Overall, the work is original and timely and can be suitable for Nature. However, I believe the following revisions are necessary to strengthen the mechanistic conclusions and the reliability of the device claims.

We sincerely thank the reviewer for the positive and constructive feedback. To clarify our main objective, **the key novelty of this work lies in a molecular design strategy that integrates two conventionally incompatible functionalities-electroluminescence and nanopatternability-within a single polymer chain. Enabled by this design, we have achieved an unprecedented pattern resolution (110 nm) alongside encouraging device performance (EQE of 13%).** We have carried out new experiments and analysis to address the Reviewer's suggestions. Please find our response as follows.

Comment 1. *Incomplete photophysical characterization as TADF polymers (need k_r and k_{RISC}). While prompt/delayed lifetimes and PLQY are reported for each polymer, key kinetic parameters intrinsic to TADF-most importantly the radiative rate constant (k_r) and the reverse intersystem crossing rate (k_{RISC})-are not evaluated. Without these parameters, the structure–property relationship (e.g. core-shell design, separation from crosslinking sites, compositional variations) remains insufficiently supported. Please extract at least* ***k_r and k_{RISC}*** *(and ideally other rates such as non-radiative channels) using a standard kinetic model. Also, temperature-dependent PL measurements (lifetime/PLQY) help in understanding the nonradiative decay processes. This will substantially improve the usefulness and generality of the work.*

We thank the Reviewer for the constructive suggestions. We have thoroughly examined photophysical properties based on the time-resolved photoluminescence (TRPL) responses, including the prompt/delayed lifetimes (τ_{PF}/τ_{DF}) and prompt/total PLQYs (Φ_{PF}/Φ_{tot}), including the evaluation of kinetic parameters using a well-established TADF kinetic model reported by the Adachi group.[1,2] The kinetic parameters, including prompt rate constant (k_p), delay rate constant (k_d), radiative decay rate constant (k_r^s), reverse intersystem crossing rate constant (k_{RISC}), intersystem crossing rate constant (k_{ISC}), are summarized in the **Fig. R1.1 and Table R1.1** (**Supplementary Fig. 54** and **Supplementary Table 8**) as follows.

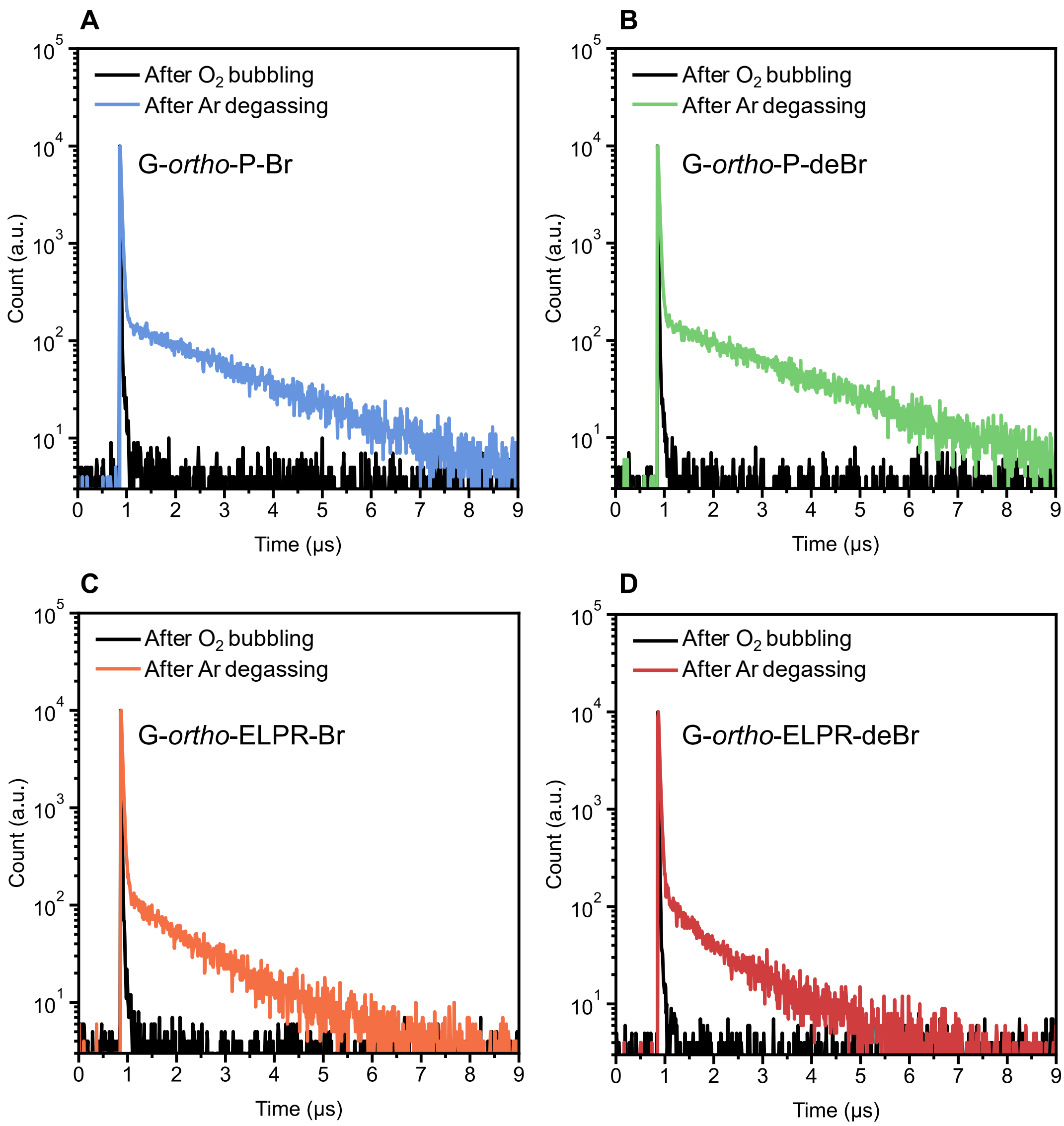

Fig. R1.1 (***Supplementary Fig. 54***) | TRPL responses for the synthesized green-emitting TADF polymers dissolved in toluene measured at room temperature. (**A**) **G-*ortho*-P-Br** (DP_{ACz} = 45, before end-group de-activation), (**B**) **G-*ortho*-P-deBr** (DP_{ACz} = 45, after end-group de-activation), (**C**) **G-*ortho*-ELPR-Br** (DP_{ACz} = 45, DP_{MAc} = 12), (**D**) **G-*ortho*-ELPR-deBr** (DP_{ACz} = 45, DP_{MAc} = 12).

Table R1.1 (*Supplementary Table 8*) | Extracted photophysical characteristics for the green-emitting TADF polymers dissolved toluene solution (0.1 mM).

**Polymers (DP[a])**	**G-*ortho*-P** (DP_{ACz} = 45)		**G-*ortho*-ELPR** (DP_{ACz} = 45, DP_{MAc} = 12)	
**End group de-activation?**	N	Y	N	Y
$\lambda_{PL}{}^{b}$ **(nm)**	520	521	519	520
$\tau_{PF}{}^{c}$ **(ns)**	27.1	27.6	27.5	28.0
$\tau_{DF}{}^{d}$ **(μs)**	2.04	2.17	1.14	0.98
$\Phi_{tot}{}^{e}$ **(%)**	67	83	50	46
$\Phi_{PF}{}^{f}$ **(%)**	11	12	11	11
$\Phi_{DF}{}^{g}$ **(%)**	56	71	39	35
$k_{PF}{}^{h}$ **(s^{-1})**	3.69×10^{7}	3.62×10^{7}	3.64×10^{7}	3.57×10^{7}
$k_{DF}{}^{h}$ **(s^{-1})**	4.90×10^{5}	4.61×10^{5}	8.75×10^{5}	1.02×10^{6}
$k_{r}^{S\,h}$ **(s^{-1})**	4.06×10^{6}	4.35×10^{6}	4.00×10^{6}	3.93×10^{6}
$k_{nr}^{T\,h}$ **(s^{-1})**	1.82×10^{5}	8.91×10^{4}	4.92×10^{5}	6.18×10^{5}
$k_{RISC}{}^{h}$ **(s^{-1})**	2.80×10^{6}	3.10×10^{6}	3.49×10^{6}	3.64×10^{6}
$k_{ISC}{}^{h}$ **(s^{-1})**	3.28×10^{7}	3.19×10^{7}	3.24×10^{7}	3.18×10^{7}

a. DP was determined by ^{1}H-NMR. *b*. emission peak. *c*. τ_{PF} is prompt fluorescence lifetime measured after oxygen bubbling. *d*. τ_{DF} is delayed fluorescence lifetime measured after argon degassing. *e*. Φ_{tot} is solution PLQY measured after argon degassing. *f*. Φ_{PF} is PLQY prompt component measured after oxygen bubbling. *g*. Φ_{DF} is PLQY delayed component calculated by $\Phi_{DF} = \Phi_{tot} - \Phi_{PF}$. All the lifetimes ($\tau_{PF}$ and τ_{DF}) are determined from dissolved polymer samples in toluene using a Hamamatsu Quantaurus-Tau Fluorescence Lifetime Spectrometer. *h*. various rate constants were estimated using Goushi and Masui method Ref.#15-16.

We have also performed temperature-dependent TRPL measurements on both the self-hosted TADF polymers and various ELPRs to elucidate their nonradiative decay processes. However, we were unable to determine the absolute PLQYs at low temperatures, because we do not have instruments to measure the absorption and integrating-sphere-integrated emission in cryostats.

The following sentences have been added to the revised manuscript on **page 5**.

[Moreover, the TADF characteristic of G-ortho-ELPR is further confirmed by a high total photoluminescence quantum yield (Φ_{tot}) of 50%, which is dominated by the delayed fluorescence quantum yield (Φ_{DF}) of 39%. The favorable exciton dynamics are attributed to the fact that the non-radiative rate constant of the triplet state (k_{nr}^{T} = 6.18×10^{5} s^{-1}) is outpaced by the rapid reverse intersystem crossing rate (k_{RISC} = 3.64 ×10^{6} s^{-1}) and radiative rate constant (k_r^{s} = 3.93×10^{6} s^{-1}). The photophysical and kinetic characteristics of the self-hosted TADF polymers and ELPRs are summarized in **Supplementary Table 8**.

In addition, the temperature-dependent TRPL responses of the thin-film sample of G-ortho-deBr reveal that τ_{DF} increases with decreasing temperature from 298 K to 220 K, exhibiting typical TADF characteristics (**Supplementary Fig. 76** and **Supplementary Table 12**).[51]]

The following sentences have been also added to the Supplementary Information in the **Supplementary Note 1**, **Measurement** section on page 4-5.

[To isolate and quantify the prompt and delayed emission components, the photoluminescence quantum yields (PLQYs) and time-resolved photoluminescence (TRPL) spectra of the TADF polymers were recorded in 0.1 mM dilute toluene solutions, utilizing oxygen bubbling to determine the prompt PLQY (Φ_{PF}).[14] The efficient quenching of triplet states (T_1) by oxygen ensures that the emission occurs exclusively via the prompt singlet pathway.

The total PLQY (Φ_{tot}) is measured after argon degassing the TADF polymer solution for 30 minutes to entirely remove dissolved oxygen, allowing both singlet and triplet pathways to contribute for PL emission. This yielded a Φ_{tot} of TADF polymer in the toluene solution.

The delayed PLQY (Φ_{DF}), in **Supplementary Table 8**, is calculated directly by subtracting the Φ_{PF} from Φ_{tot}.

$$\Phi_{DF} = \Phi_{tot} - \Phi_{PF}$$

This photophysical assignment is explicitly corroborated by the TRPL decay spectra provided in **Supplementary Fig. 54**. In the oxygen-bubbled toluene solution, the triplet excited states are fully quenched, resulting in a clean, monoexponential prompt decay profile devoid of any delayed features.[3] In stark contrast, the argon-degassed solution exhibits a pronounced long-lived delayed emission component with a prompt component. Both prompt and delayed exciton lifetimes (τ_{PF} and τ_{DF}) were estimated by fitting the TRPL decay curves with an Arrhenius biexponential function. This clearly confirms that the triplet excited states are preserved under inert conditions and successfully undergo thermally activated reverse intersystem crossing (RISC) back to the singlet manifold ($T_1 \rightarrow S_1$) to

generate delayed fluorescence. Note that the high k_{RISC} in these TADF polymers facilitates rapid triplet upconversion, a process intrinsically kinetically coupled to their high k_{ISC}.

By extracting the τ_{PF}, τ_{DF}, k_{PF} and k_{DF} from these TRPL curves (**Supplementary Fig. 54**) alongside the PLQY data, we evaluated the key kinetic rate constant of the self-hosted TADF polymers and ELPRs. We then derived the key kinetic characteristics, such as radiative decay rate constant (k_r^S), reverse intersystem crossing rate constant (k_{RISC}), intersystem crossing rate constant (k_{ISC}), and non-radiative rate constant from triplet state (k_{nr}^T) using a well-established TADF kinetic model reported by Adachi group.[15,16] All photophysical characteristics, and derived rate constants are summarized in **Supplementary Table 8**.]

References in SI

14. Serevičius, T. et al. TADF parameters in the solid state: an easy way to draw wrong conclusions. J. Phys. Chem. A **125**, 1637–1641 (2021).

15. Goushi, K., Yoshida, K., Sato, K. et al. Organic light-emitting diodes employing efficient reverse intersystem crossing for triplet-to-singlet state conversion. Nat. Photonics **6**, 253-258 (2012).

16. Masui, K., Nakanotani, H. & Adachi, C. Analysis of exciton annihilation in high-efficiency sky-blue organic light-emitting diodes with thermally activated delayed fluorescence. Org. Electron. **14**, 2721-2726 (2013).

51. Tsuchiya, Y., Mizukoshi, K., Saigo, M. et al. Temperature dependency of energy shift of excitonic states in a donor-acceptor type TADF molecule. Nat Commun. **16**, 4815 (2025).

We have added Supplementary Fig. 54 and 76 on pages 75 and 101 and **Supplementary Tables 8** and **12** on pages 76 and 101 to the Supplementary information.

Finally, we have carried out temperature-dependent TRPL measurements (**Fig. R1.2 attached below**) to extract the energy gap between singlet and triplet states, ΔE_{ST} of our representative TADF polymer G-o-P-deBr. The value was determined to be 40.4 meV.

Supplementary Table 12 | Temperature dependent TRPL lifetimes of green-emitting self-hosted TADF polymer after (G-*ortho*-P-deBr) end group de-activation with DP_{ACz} = 45 in thin fim.

Temperature (K)	τ_{PF} (*ns*)	τ_{DF} (*μs*)	A1	A2
298	27.68	1.52	4503.28	85.31
280	30.82	1.88	3803.97	79.77
260	30.76	2.09	4073.55	75.21
240	27.57	2.35	8911.89	47.61
220	29.37	2.39	4145.12	53.51
200	29.45	2.36	4714.49	42.45
150	29.31	2.20	9222.21	28.27
100	28.45	1.23	10784.7	21.00
77	29.06	0.90	4865.65	19.25

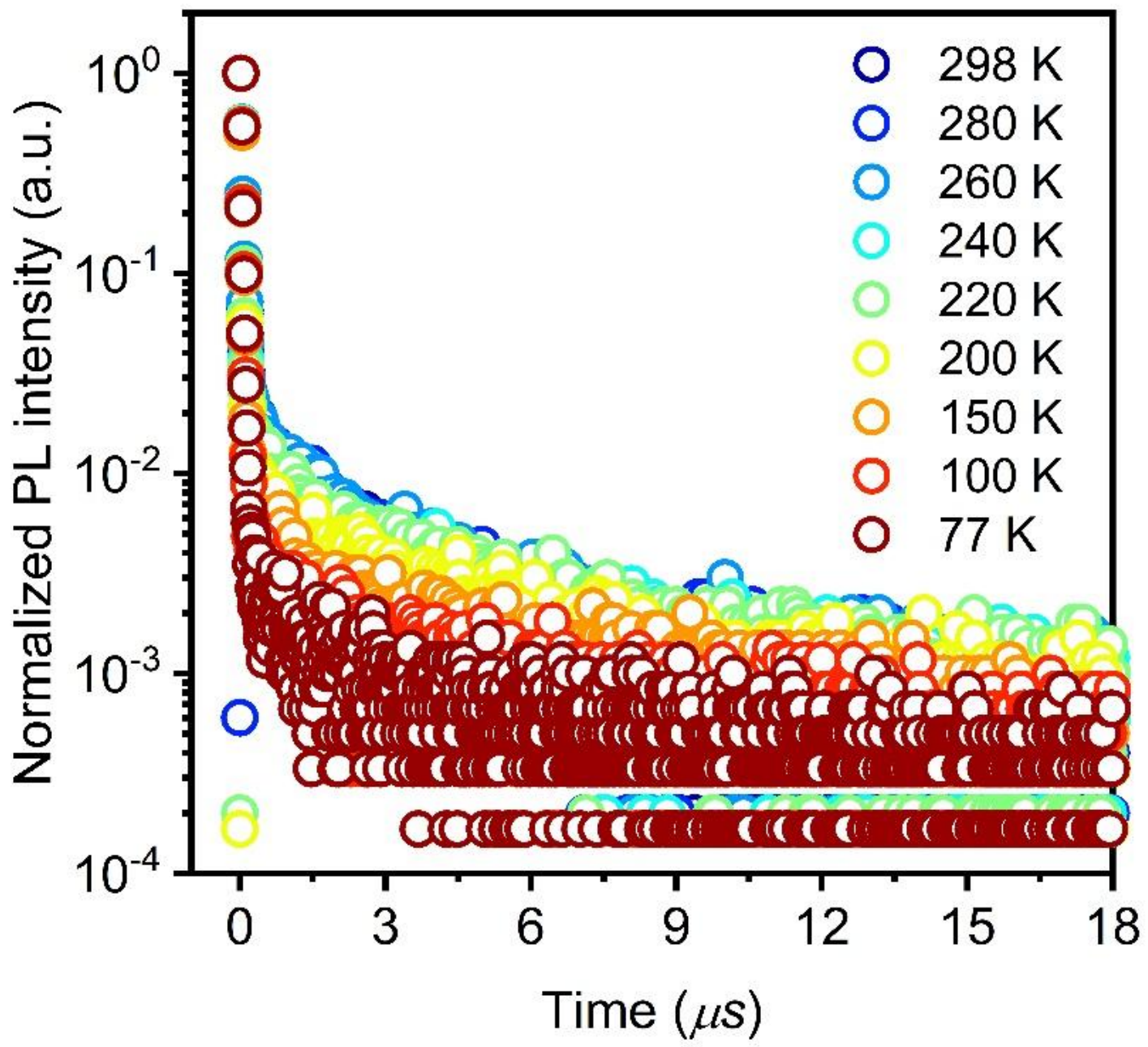

Supplementary Fig. 76: Thin film TRPL spectra of green-emitting self-hosted TADF polymer after (G-*ortho*-P-deBr) end group de-activation with DP_{ACz} = 45 measured at various temperature from 298 to 77K. First, delay fluorescence lifetime component increased from 298 to 220K, which is typical characteristics of TADF phenomena.

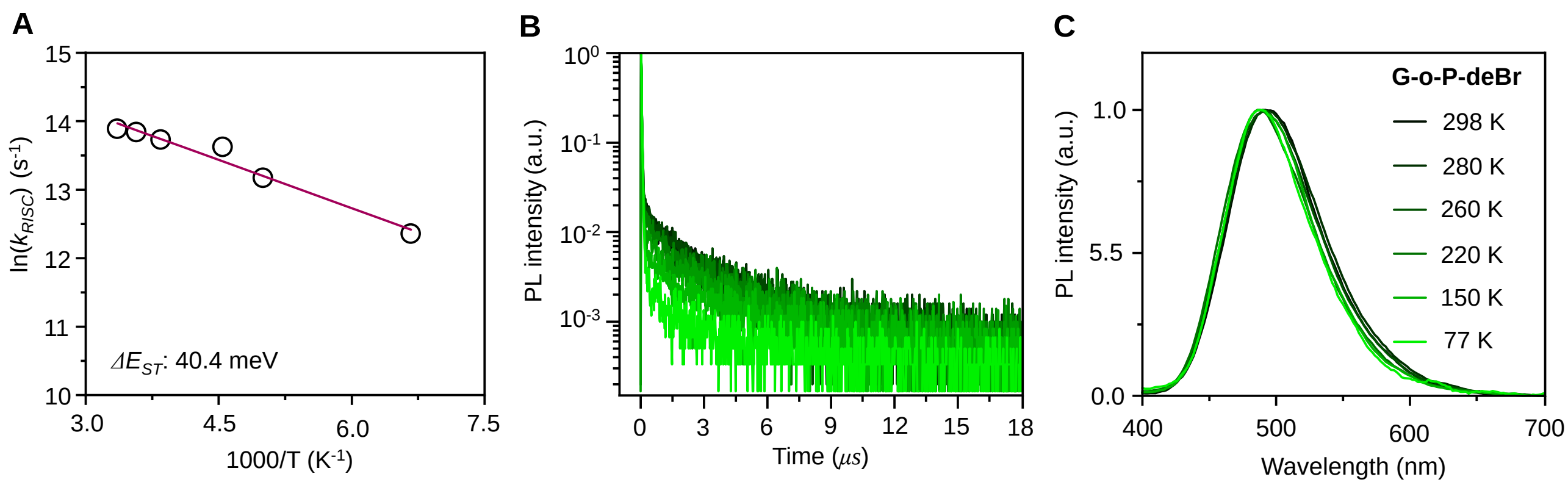

Fig. R1.2 | Determination of the energy gap between singlet and triplet states, ΔE_{ST} of our representative TADF polymer G-ortho-P-deBr using (A) the Arrhenius law fitting of k_{RISC}, extracted from the temperature dependent TRPL measurements (B). (C) PL spectra at various temperatures.

Comment 2. *Reliability/reproducibility of EQE at low luminance (<100 cd m $^{-2}$).*

The EQE–luminance data appear reasonably stable above 100 cd m $^{-2}$, but below this regime, the number of data points is limited, and the behavior looks unstable. In particular, the ~30% EQE in Fig. 5 is a key result; however, the apparent double-peak feature raises concerns about measurement artifacts or device integrity issues (e.g., pinholes/leakage/local shorts, scan-history dependence). Please repeat J–V–L/EQE measurements with multiple devices (statistics) and scan-direction / repeated scans, and provide evidence of reproducibility. If possible, add supporting diagnostics related to leakage/defects (e.g., microscopy, yield, uniformity, leakage current analysis). This will ensure the reported high EQE is robust and not an artifact. PS: EQE-current density characteristics are more fundamental than the EQE-Luminance.

We thank the Reviewer for his/her critical observation and constructive suggestion. In response to the concerns, we have fabricated new batches of devices to carefully examine their reliability and reproducibility. We have implemented the new results in the updated manuscript and SI. Please find our detailed response as follows.

Robustness and independent cross-check of the maximum EQE

To examine the characterized EQE behavior, we fabricated new batches of devices and carefully cross-checked the EL responses. First, we tested the devices using the absolute EQE measurement setup equipped with a 3.3 inches integrated sphere. The OLED device employing G-*ortho*-P-deBr as the emissive layer (EML), with the device architecture of ITO/PEDOT:PSS blend(AI 4083/CH 8000 mix)/EML/TmPyPB/Liq/Al, again demonstrated a maximum EQE of 30.9% with consistent EQE-current behavior (**Fig. 5a** and **5c** in the revised manuscript and **Fig. R1.3** attached below). In particular, in response to the Reviewer's suggestion, panels (a) and (c) of **Fig. 5** (**Fig. R1.3**) have been revised to EQE as a function of current density instead of luminance, as this relationship is more fundamental to device physics during operation.

Next, we cross-checked the EL characteristics using a calibrated PR655 Spectroradiometer. As shown in **Fig. R1.4 below,** the spectroradiometer-based measurement reproduced an EQE exceeding 30%, and the EQE-J response and its overall trend are also consistent, with at least three data points above 20%. We are therefore confident that the measured EL performance is intrinsic and robust. The characteristics have been included in the updated SI (**Supplementary Fig. 55**; attached below).

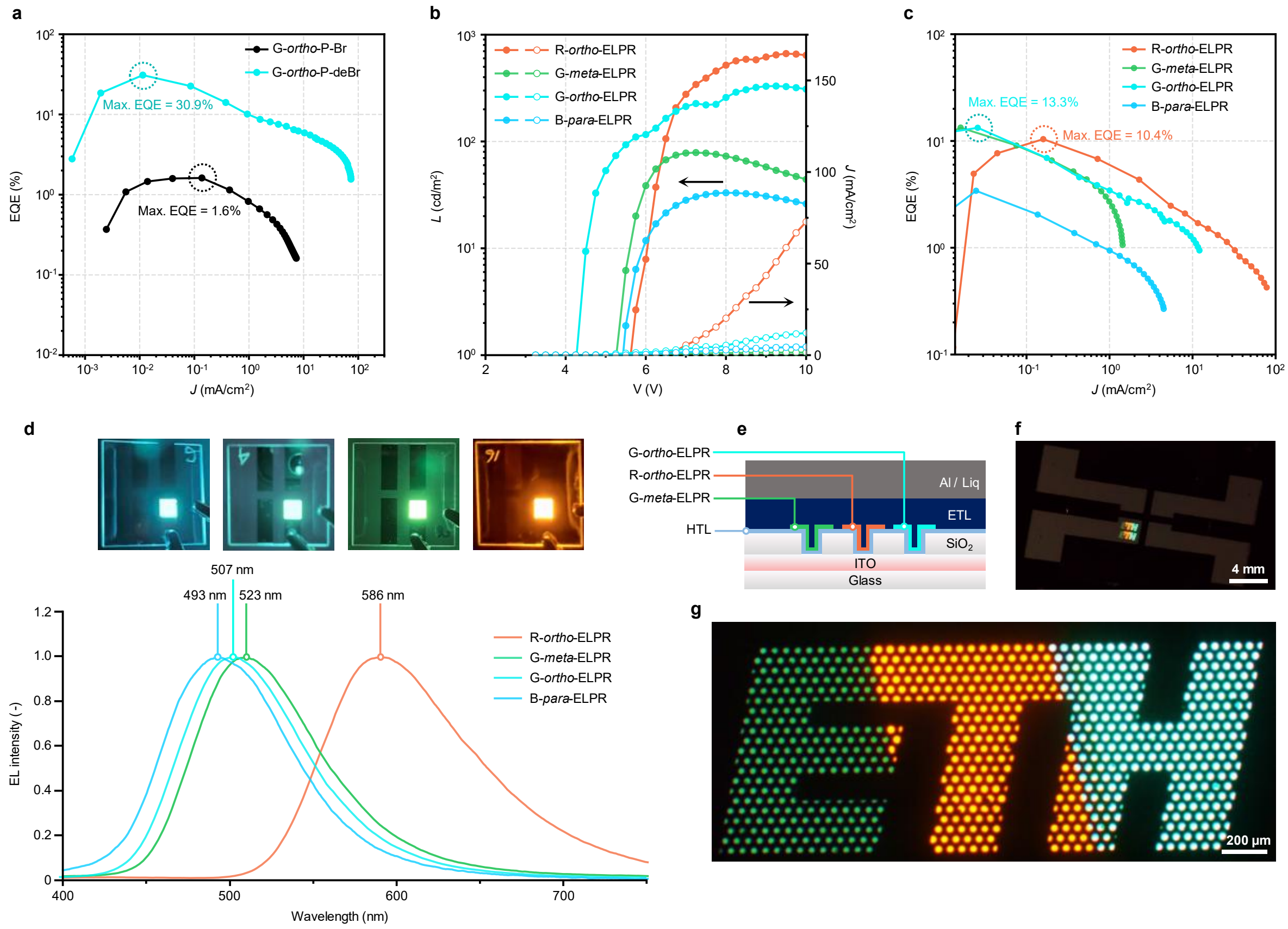

Fig. R1.3 ***(Main text Fig. 5a & 5c)*** **|** Self-hosted G-*ortho*-P and ELPR OLED performance and multicolour integration.

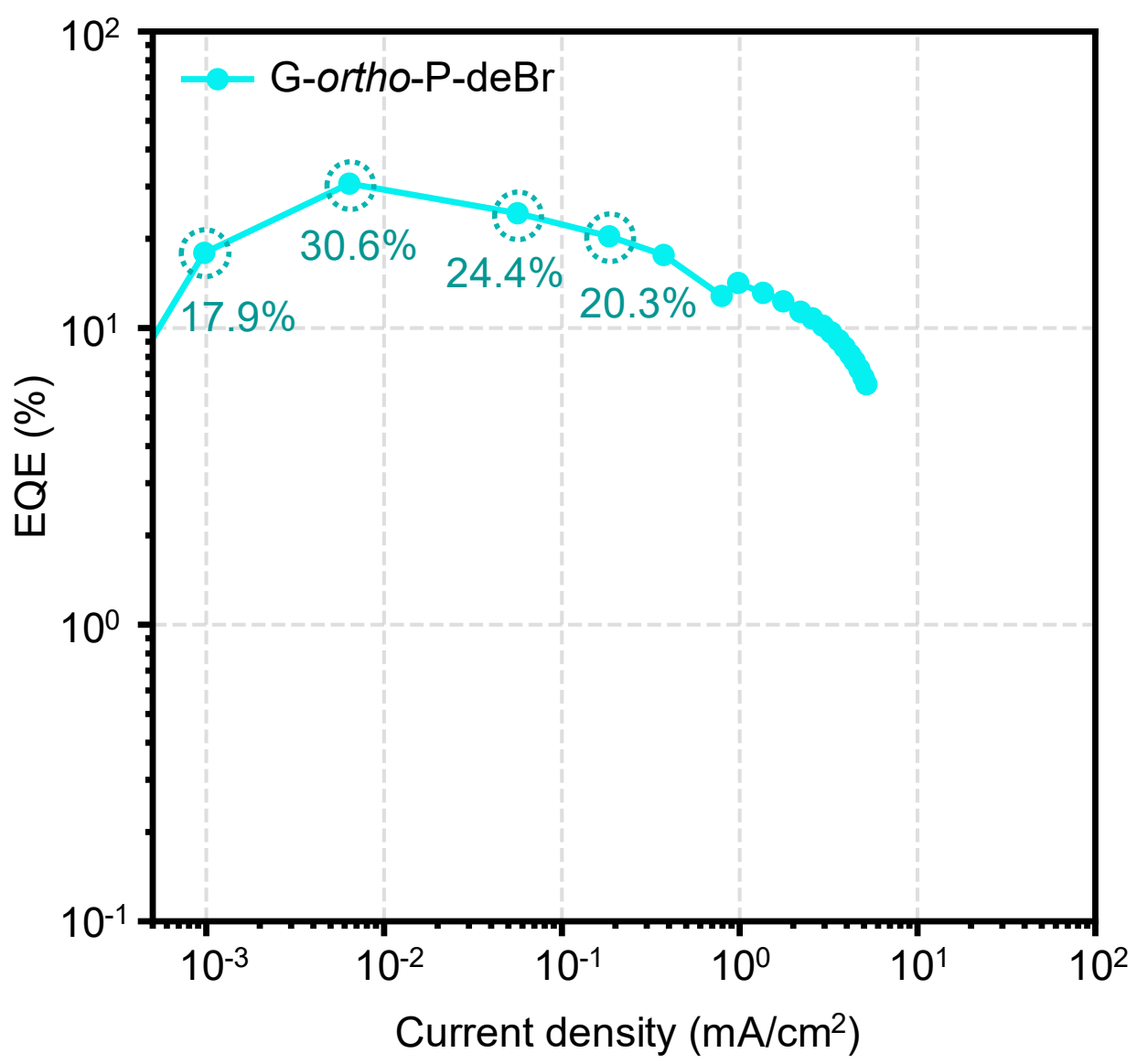

Fig. R1.4 ***(Supplementary Fig. 55)*** **|** Independent cross-check of the G-*ortho*-P-deBr OLED performance using a calibrated PR655 spectroradiometer. The EQE-current density curve shows a maximum EQE of 30.6% and still exhibit the characteristic double-peak behavior, with multiple EQE data points exceeding 20%.

Statistics of the maximum EQE

Next, to address the concern about measurement robustness and reproducibility, we have fabricated and characterized 33 OLED devices based on G-*ortho*-P-deBr; the statistical histogram please see **Fig. R1.5 below** (revised **Extended Data Fig. 4f**). The maximum EQE distribution is well described by a normal fit with a mean EQE of 31.3% and a standard deviation of 1.7%, demonstrating that the ~30% EQE reported in the main text is robust and statistically representative. In the revised manuscript (**Extended Data Fig. 4**), panels (e) and (f) have been replaced. Panel (e) of *J*-*V*-*L* curve was revised, showing the *J*-*V*-*L* characteristics up to 10 V. Panel (f) was revised by increasing device population for statistical histogram analysis.

We have updated **Extended Data Fig. 4e and 4f** on page 27 to the revised manuscript.

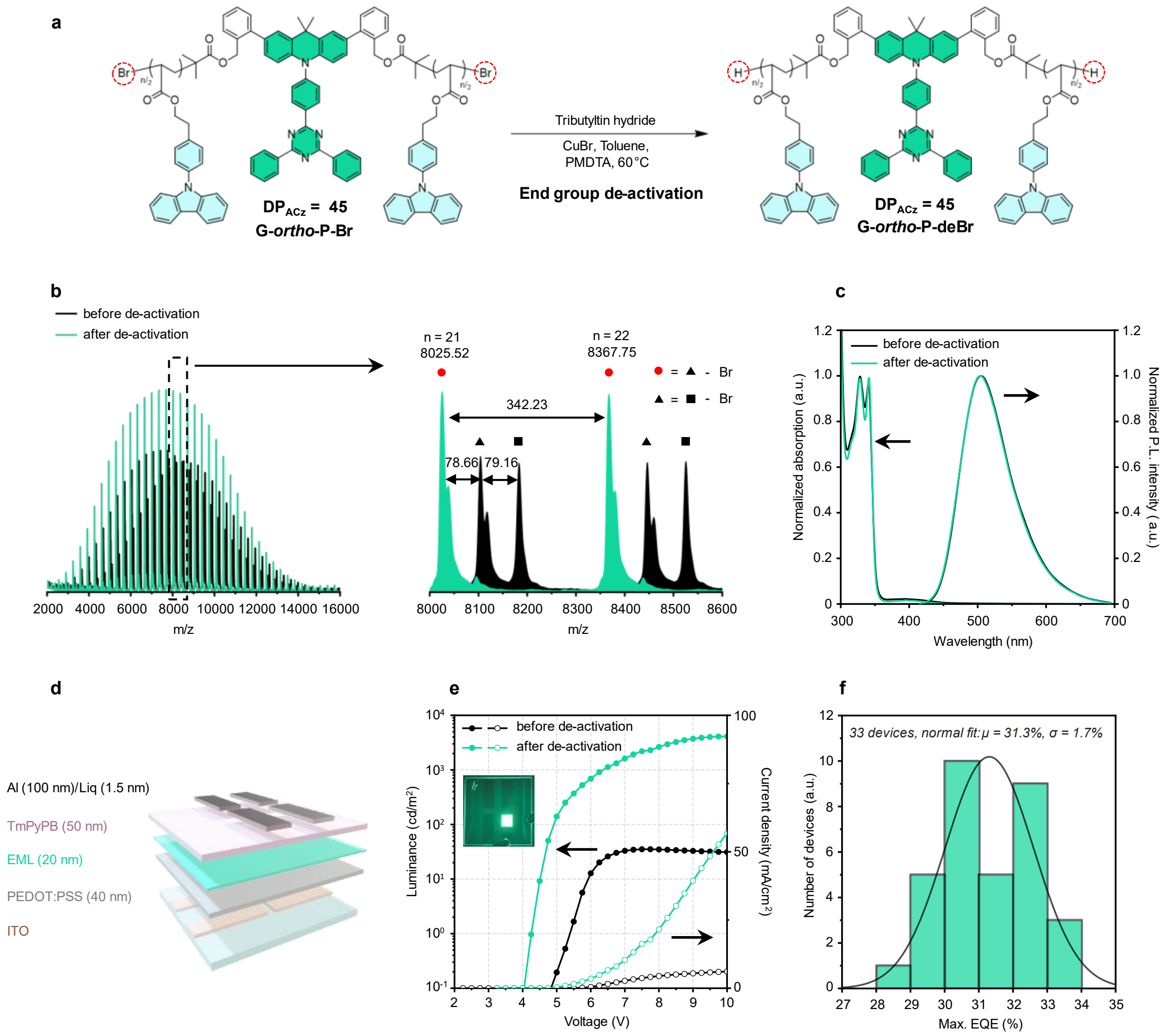

Fig. R1.5 ***(Extended Data Fig. 4)*** **|** EL enhancement of green-emitting self-hosted TADF polymer before (G-*ortho*-P-Br) and after (G-*ortho*-P-deBr) end-group deactivation.

Repeated-scan and scan-direction of J-V & EQE-J measurements

To further examine the effects of voltage scan direction and possible charge accumulation or leakage, we first characterized the G-*ortho*-P-deBr OLEDs by increasing the voltage linearly from −1 to 10 V (**Fig. R1.6 below**). For V < 3V, the devices showed low leakage current (J < 10^{-4} mA cm^{-2}), without abnormal leakage. After OLED turned on (J > 10^{-3} mA cm^{-2}), the EQE increased rapidly and exhibited maximum EQE greater than above 30%. We therefore confirm that the high EQE values are not artifacts or resulting from leakage paths.

Next, with the same OLED pixel, we performed up to four repeated *J-V* scans from −5 to 7 V; all scans overlapped closely in both negative and positive voltage regions, demonstrating robust electrical characteristics under repeated biasing (**Fig. R1.7 below**).

Finally, to examine electrical hysteresis, the *J-V* characteristics were measured with both forward and reverse voltage sweeping (**Fig R1.8 below**). The forward and reverse scans overlapped nicely across a wide current range spanning several orders of magnitude, demonstrating negligible hysteresis.

All in all, these results indicate that the ~30% maximum EQE is reproducible and not dominated by scan direction or device instability. We have added these figures in the revised SI (**Supplementary Fig. 56-58**).

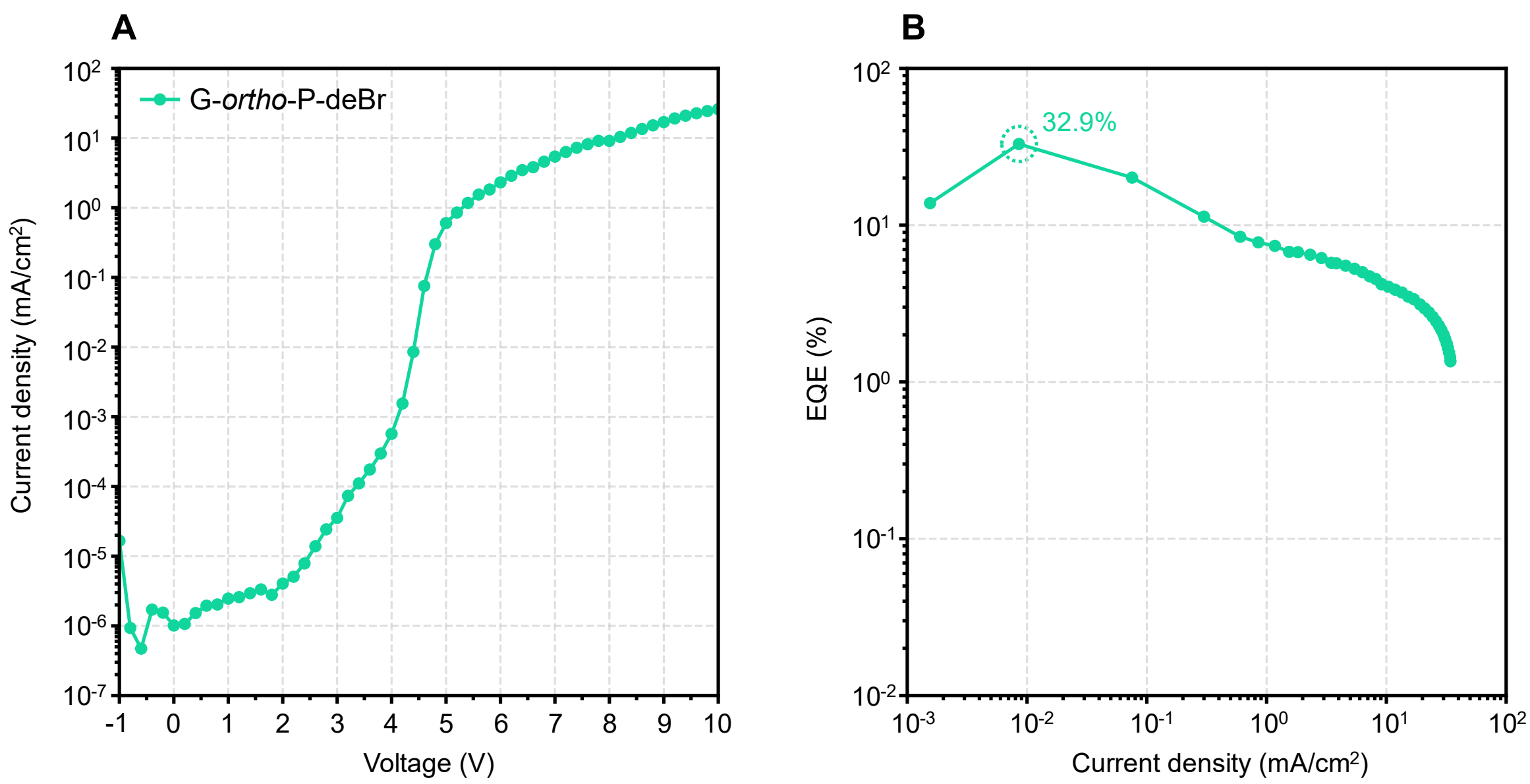

Fig. R1.6 ***(Supplementary Fig. 56)*** **|** Current density-voltage (**A**) and EQE-voltage (**B**) curves of the G-*ortho*-P-deBr OLED measured by sweeping the voltage from −1 V to 10 V to avoid built-in charge accumulation. Before turn-on (below 3 V), the device exhibits a low leakage current density of < 10^{-4} mA cm^{-2}, and a maximum EQE exceeding 30% is still reproducibly recorded.

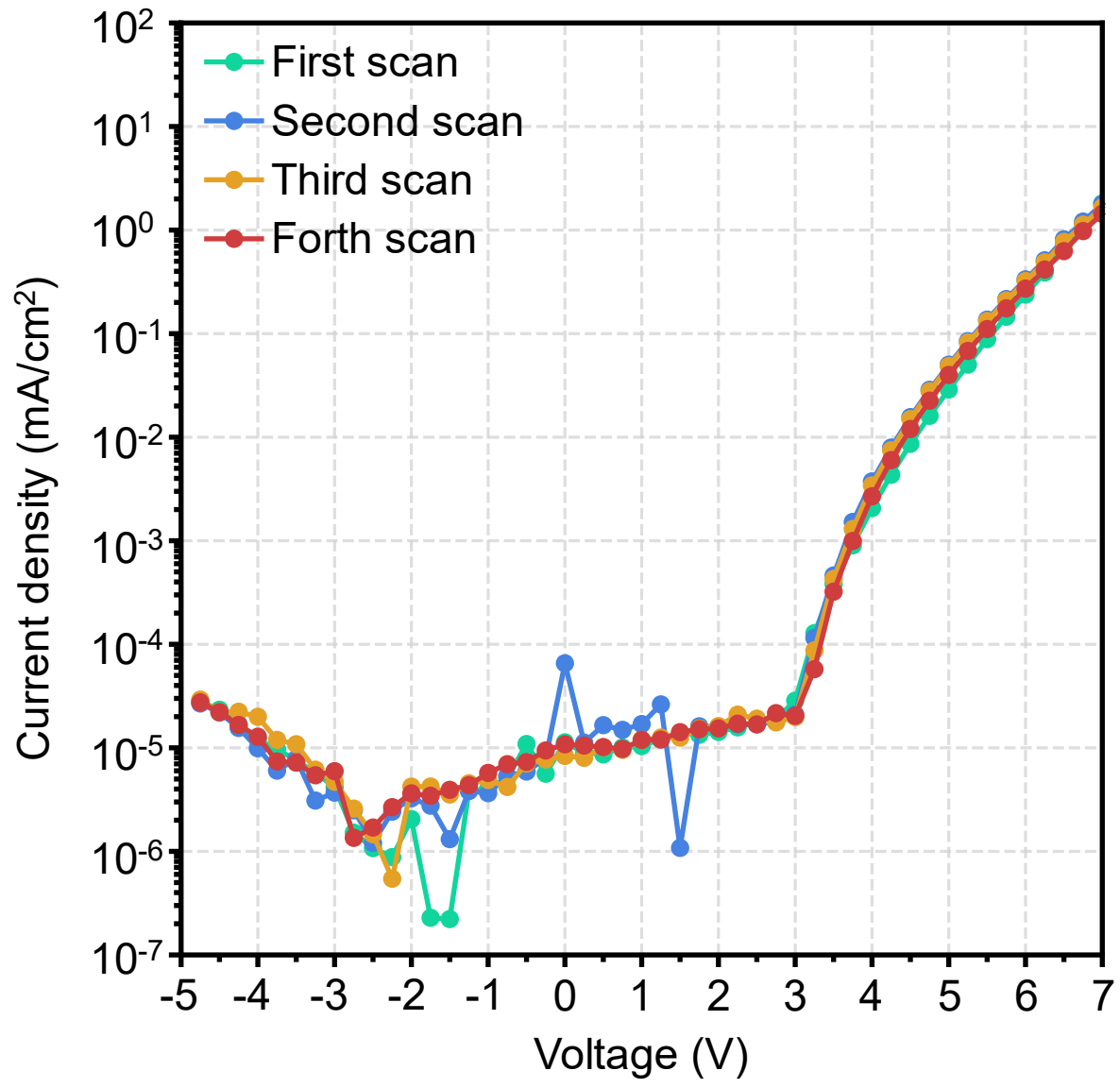

Fig. R1.7 (***Supplementary Fig. 57***) **|** Four consecutive *J*-*V* scans of the G-*ortho*-P-deBr OLED measured by sweeping the voltage from −1 V to 10 V. The scans overlap closely over both the negative and positive voltage ranges, with only modest fluctuations between repeats.

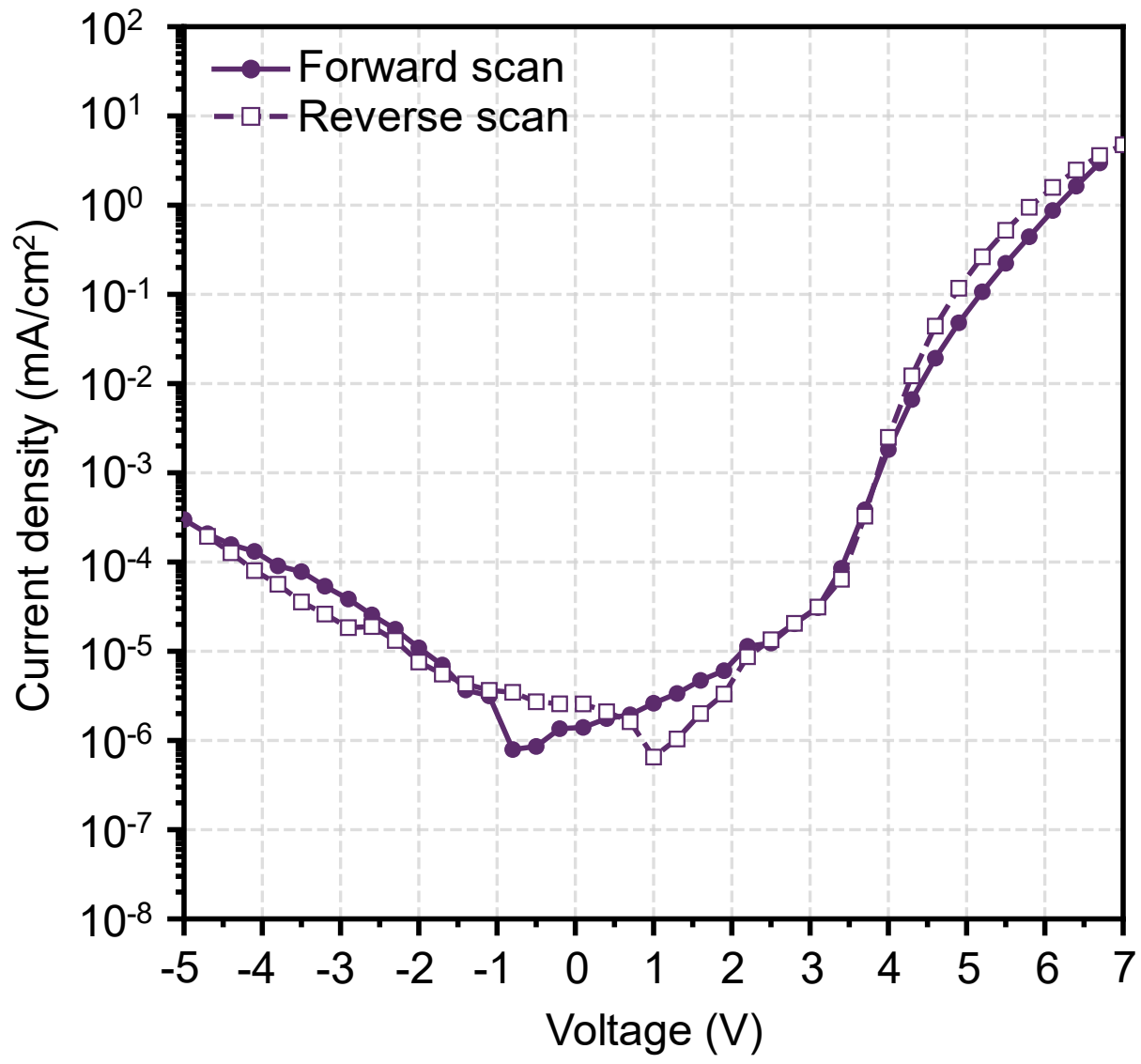

Fig. R1.8 (***Supplementary Fig. 58***) **|** Forward and reverse scans of *J*-*V* curves for the G-*ortho*-P-deBr OLED.

Examination of morphological defects

To directly examine possible presence of pinhole-induced emissive defects in OLEDs, we have fabricated and tested the EL characteristic of the OLED device employing G-*ortho*-P-deBr as EML with the representative architecture ITO/PEDOT:PSS blend (AI 4083/CH 8000 mix)/EML/TmPyPB/Liq/Al. **Fig. R1.9** below presents its EL microscope image operating at 5 V. Within the microscopic diffraction limit, as far as we can tell, the emission is spatially uniform across the entire active area; we did not observe localized bright/dark spots. This observation offers a first evidence that the morphological defects are negligible to the overall device performance.

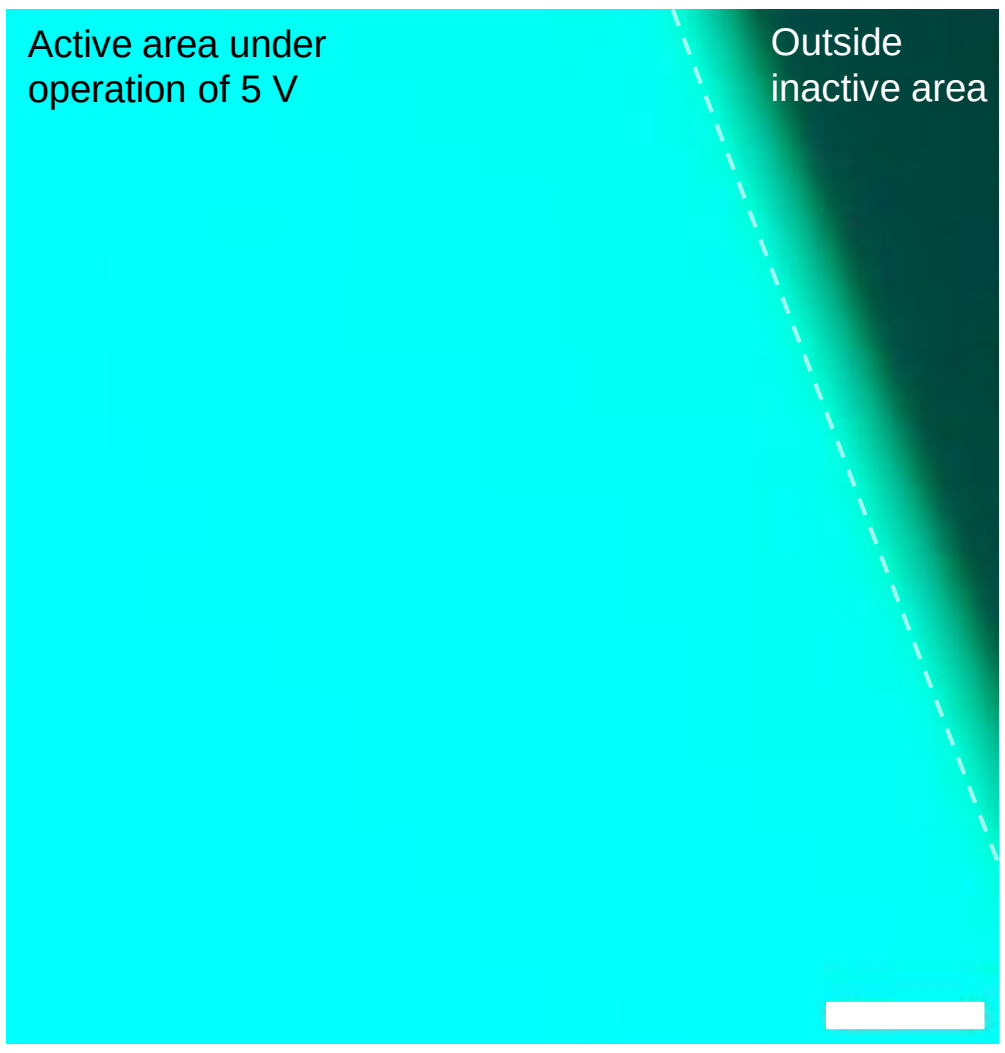

Fig. R1.9 (***Supplementary Fig. 59***) **|** EL microscopic image of the G-*ortho*-P-deBr OLED device operated under 5 V. Scale bar: 25 μm.

To further examine the surface morphologies of the EML films, we performed atomic force microscopy (AFM) on both the self-hosted polymers (G-*ortho*-P-Br and G-*ortho*-P-deBr) (**Fig. R1.10 below**) and the ELPR films (R-*ortho*-ELPRs; before UV exposure, after UV exposure, and after development) (**Fig. R1.11 below**). The AFM measurements were carried out on a Bruker system in PeakForce Quantitative Nanomechanics (QNM) mode in air using a HiRes-C19/Cr-Au probe with a tip radius < 1 nm, enabling simultaneous acquisition of height, Derjaguin–Muller-Toporov (DMT) modulus, and adhesion maps. Across all samples and processing conditions, the AFM height images reveal smooth and continuous surface morphologies for both the spin-coated self-hosted TADF polymer and ELPR films, with Root Mean Square roughness (RMS) of approximately 2 nm. No visible pinholes, cracks, or other defects were observed. We are therefore confident that the high EQE values as well as the electrical characteristics do not result from film morphology defects, but rather reflect the intrinsic performance of the OLED architecture.

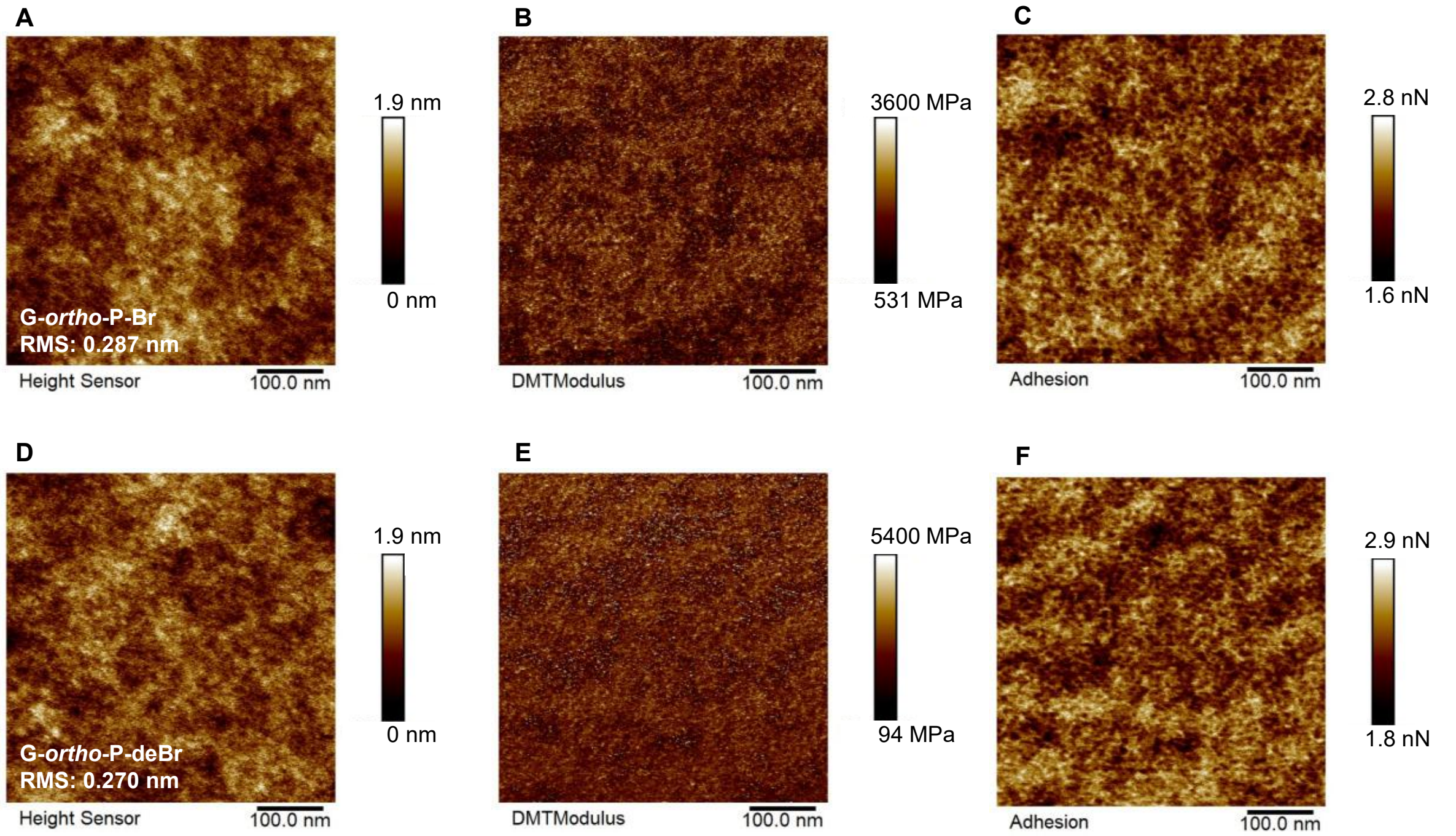

Fig. R1.10 (***Supplementary Fig. 60***) **|** AFM QNM images of G-*ortho*-P-Br (from **A** to **C**) and G-*ortho*-P-deBr (from **D** to **F**) thin films. (**A**, **D**) Height channel images, (**B**, **E**) DMT modulus images, (**C**, **F**) Adhesion channel images.

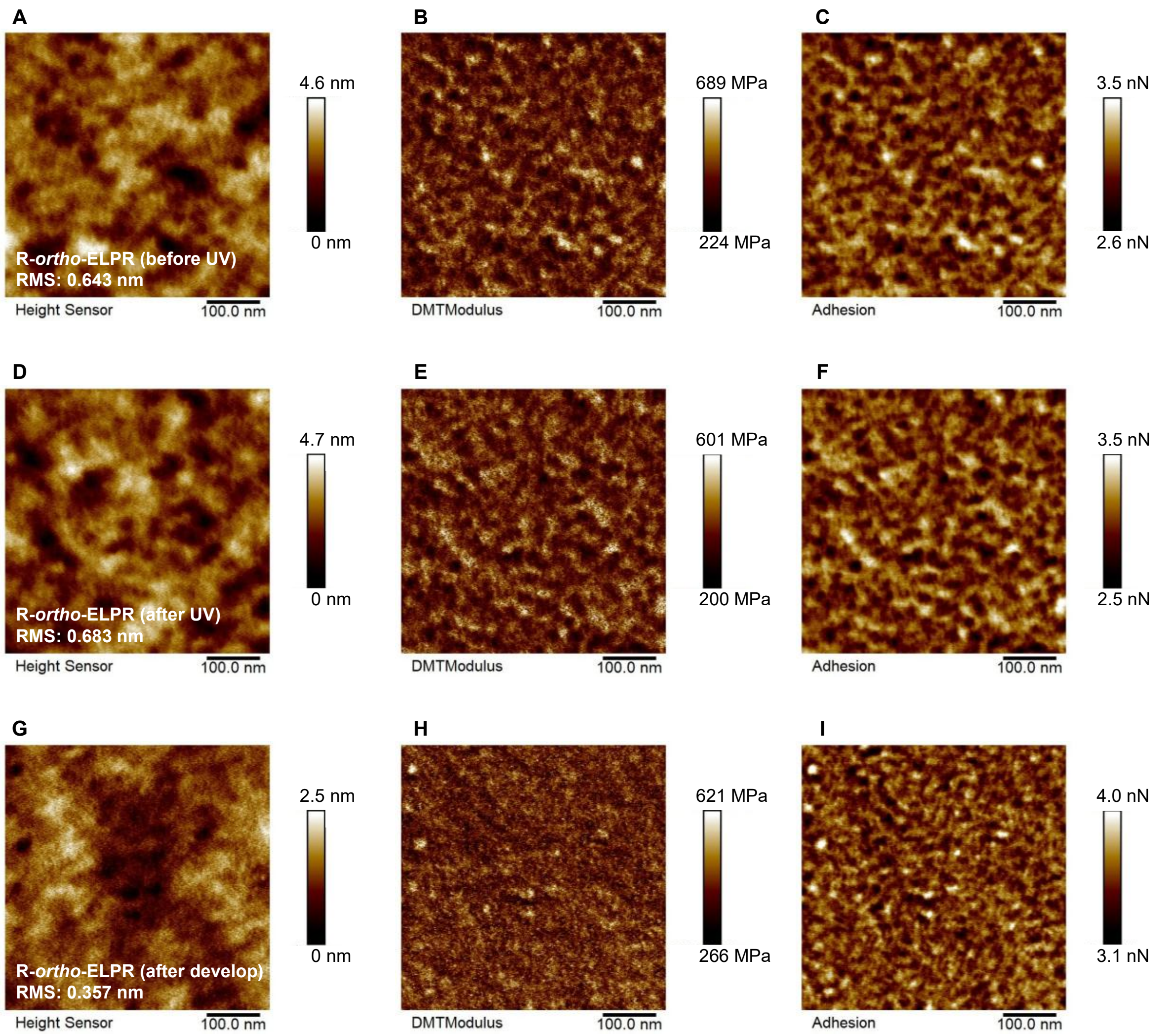

Fig. R1.11 (***Supplementary Fig. 61***) **|** AFM QNM characterization images of R-ortho-ELPR thin films at different photolithography process stages (before UV exposure; from **A** to **C**), (after UV exposure; from **D** to **F**), (after development; from **G** to **I**). (**A**, **D, G**) Height channel images, (**B**, **E, H**) DMT modulus images, (**C**, **F**) Adhesion channel images.

Interestingly, the corresponding DMT modulus and adhesion maps of the two self-hosted polymer films (G-*ortho*-P-Br and G-*ortho*-P-deBr) are topographically homogeneous, with only modest fluctuations in the apparent stiffness and adhesion interaction (see **Fig. R1.10 B & E above**). In contrast, the R-*ortho*-ELPR films exhibit nanoscale phase separations with mechanically distinct stiffness in the modulus channel (**Fig. R1.11 B, E, and H above**). We believe this is a direct observation for the segregation of crosslinker-rich (crosslinker-to-crosslinker) regions and host-rich (host-to-host) regions formed upon spin-coating.

Specifically, the adhesion map shows a similar domain pattern behavior as the modulus map (**Fig. R1.11 C, F, & I above**). After development, the characteristic domain size of R-*ortho*-ELPR becomes slightly smaller and the room mean square average (RMS) roughness is reduced, indicating that the formation of a three-dimensional crosslinked network decreases the interchain distance and leads to a more compact, laterally refined morphology (**Fig. R1.11 G, H, & I**). The in-situ AFM findings presented here nicely adhere to our molecular picture illustrated in **Fig. 3**.

With the above microscopic and nanoscale characterization, we conclude that the observed high EQE values for the self-hosted G-*ortho*-P-deBr devices do not result from film inhomogeneity or defects. They reflect the intrinsic performance of the OLED architecture. In addition, these observations support our argument in the main text that the TADF emitting core is spatially isolated from the outer-shell crosslinking moieties, which take advantage of segregation of host-to-host and crosslinker-to-crosslinker domains, as revealed in the modulus maps of R-*ortho*-ELPR films.

We have included **Supplementary Fig. 59-61** on page 81-83 in the Supplementary information.

At the end, we would like to express our sincere appreciation to the Reviewer's constructive and insightful suggestions.

Referee #2 & #3

The authors address a longstanding challenge in OLED nano-patterning by integrating TADF emitters with photoresist chemistry to directly pattern RGB subpixels. The demonstration of photolithographic patterning of functional OLED emitters represents a novel and technically ambitious approach, and this work offers a meaningful conceptual advance over conventional patterning strategies that have been considered difficult for OLED materials.

However, the technology appears to face significant performance limitations relative to conventional OLED patterning methods. It would be valuable for the authors to more clearly discuss whether ELPR-based OLEDs are realistically compatible with practical display requirements, and to outline how further material or device development could enable meaningful competition with existing commercial technologies.

We appreciate the Reviewer's constructive and insightful comments. To clarify our main objective, **the key novelty of this work lies in a molecular design strategy that integrates two conventionally incompatible functionalities—electroluminescence and nanopatternability—within a single polymer chain. Enabled by this design, we have achieved an unprecedented pattern resolution (110 nm) alongside encouraging device performance (EQE of 13%).**

We acknowledge the performance gap between our ELPRs and state-of-the-art OLEDs, particularly regarding chromaticity and operational stability, which is an issue we now explicitly discuss in the revised manuscript. To demonstrate the optimization potential of this system, we have further purified the synthesized polymers, successfully extending the device lifetime (LT_{50}) by 2.5-fold (from ~60 to ~170 min). We believe the concept established here lays a solid foundation for future molecular engineering and purification protocols to ultimately bridge this gap.

Please find our detailed response as follows.

Comment 1. *The operational lifetime of the ELPR OLEDs reported in this work is substantially shorter than that reported for many OLED and TADF-based systems in the recent literature. The manuscript attributes the limited lifetime mainly to extrinsic factors such as HTL choice, residual copper, and polymer impurities. However, it remains unclear whether the observed lifetime limitation arises primarily from insufficient materials and process optimization, or from intrinsic constraints imposed by the ELPR molecular and polymer architecture, which is designed to simultaneously support electroluminescence and photolithography.*

While the authors attribute the present lifetime limitations primarily to extrinsic factors, the manuscript provides limited evidence to substantiate this interpretation. It remains unclear to what extent these factors dominate the degradation behavior relative to the intrinsic stability of the ELPR materials. Additional experimental data would help clarify the relative contributions of material, process, and device-level effects, and would strengthen the authors' argument regarding the origin of the lifetime limitations.

We thank the Reviewer for this insightful point. We agree that distinguishing between extrinsic degradation pathways and the intrinsic stability limits of the ELPR architecture is crucial. To clarify this, we have conducted systematic purification and lifetime studies focusing on residual copper catalysts.

Specifically, regarding the impact of residual copper from ATRP, we quantified trace metal concentrations in our representative G-ortho-P-deBr polymer *via* inductively coupled plasma-optical emission spectroscopy (ICP-OES). (Methodology: 50–70 mg of polymer was digested in a 2:1 v/v nitric acid/hydrogen peroxide mixture using a microwave digestion system, diluted with ultrapure water, and analyzed on a Horiba Ultra 2 instrument calibrated against copper standards; **Fig. R2.12 below**).

As summarized in **Table R2.1 below**, standard precipitation (1× hexane, 2× methanol) left 27 ppm of residual copper. Following an intensive 48-hour dialysis against THF, this concentration was nearly halved to 14 ppm.

We subsequently evaluated the operational stability of OLEDs fabricated with the dialyzed versus non-dialyzed polymers (ITO/v-NPB/polymer/TPBi/Liq/Al; encapsulated using a chemically etched aluminosilicate cover glass integrated with a getter sheet, **Fig. R2.13 below**). Under constant current driving at 5 mA cm^{-2} (initial luminance of ~360 cd m^{-2}), the LT_{50} surged from 62 to 108 min, a 74% improvement. At 2.4 mA cm^{-2} (~175 cd m^{-2}), the LT_{50} increased from 140 to 172 min.

These results provide direct experimental proof that extrinsic ionic metal residues act as a major degradation driver. Importantly, the steep upward trajectory of device lifetime scaling with polymer purity indicates that our current stability metrics are governed by process-level cleanliness, rather than an intrinsic thermodynamic ceiling of the dual-functional polymer chain.

Beyond material purity, we also find the interfacial charge balance plays a comparably critical role. By pairing the dialyzed G-ortho-P-deBr emitter with rationally selected transport layers (v-NPB as HTL and TPBi as ETL), the optimized architecture (ITO/v-NPB/polymer/TPBi/Liq/Al) achieved the aforementioned LT_{50} of 172 min at 2.4 mA cm^{-2} (**Fig. R2.13 below**), representing a ~2.4-fold enhancement over our unoptimized baseline reported in the original manuscript.

Collectively, these results substantiate that the current stability bottleneck is governed by extrinsic, addressable factors (i.e., trace metallic impurities and carrier charge accumulation), rather than an intrinsic design flaw. While the absolute lifetime remains modest compared to commercial OLED benchmarks, clear synthetic and technological pathways exist to unlock further gains:

1. **Process-wise:** applying chelating-resin treatments to suppress residual copper <5 ppm; and
2. **Synthetically:** transitioning to metal-free controlled radical polymerizations, such as reversible addition–fragmentation chain transfer (RAFT) or nitroxide-mediated polymerization (NMP), to fundamentally eliminate transition-metal contamination.

The following sentences have been added to the revised Manuscript on page 13.

"By halving the residual copper content *via* intensive dialysis (**Supplementary Fig. 62** and **Supplementary Table 9**) and replacing the baseline PTAA/TmPyPB transport layers with cross-linkable N4,N4'-di(naphthalen-1-yl)-N4,N4'-bis(4-vinylphenyl)biphenyl-4,4′-diamine (v-NPB) and 1,3,5-Tris(1-phenyl-1H-benzimidazol-2-yl)benzene (TPBi), respectively, the optimized G-ortho-P-deBr device exhibited an extended LT_{50} of 172 min at a constant current of 2.4 mA cm^{-2} (**Supplementary Fig. 63**). This represents a 135% lifetime improvement over the control device, notably achieved under a 2.4-fold higher operating current. Furthermore, under elevated driving currents of 5.0 and 10 mA cm^{-2}, the devices recorded solid LT_{50} values of 108 and 30 min, respectively (**Supplementary Figs. 63 and 65**). A parallel stability enhancement was observed for the sister system, where the dialyzed G-ortho-ELPR device achieved an LT_{50} of 146 min (**Supplementary Fig. 63d**)."

We have included **Supplementary Fig. 62 and 63** on page 84 and 86. Supplementary Table 9 has been added on page 85 to Supplementary information.

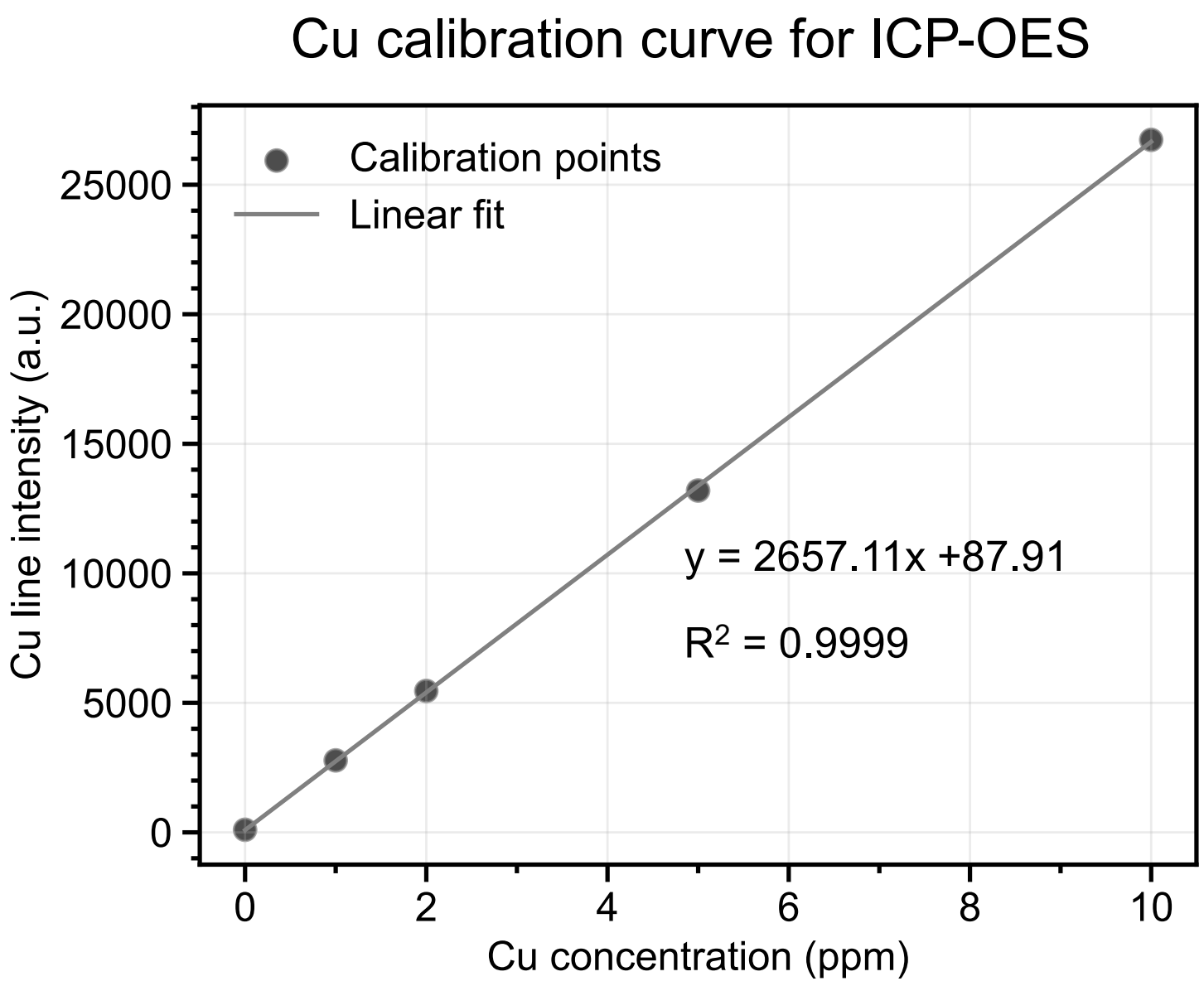

Fig. R2.12 (***Supplementary Fig. 62***) **|** Calibration curve of copper standards used for ICP-OES measurement.

Table R2.1 (*Supplementary Table 9*) | Quantification of copper residue concentration in the G-*ortho*-P-deBr polymer sample before and after dialysis, where the dialysis was performed against THF with multiple solvent exchanges in order to reduce the copper residue inherited from ATPR synthesis.

**G-*ortho*-P-deBr**	**Before dialysis**	**After dialysis**
**Cu line intensity at 327 nm (a.u.)**	407.36	251.96
**Detected Cu conc. (ppm; mg/L)**	0.12	0.06
**Volume of the diluted sample (L)**	0.015	0.015
**Cu mass in the diluted sample (mg)**	0.002	0.001
**Digested polymer mass (mg)**	66.5	64.1
**Cu conc. in polymer sample (ppm)**	**27.12**	**14.45**

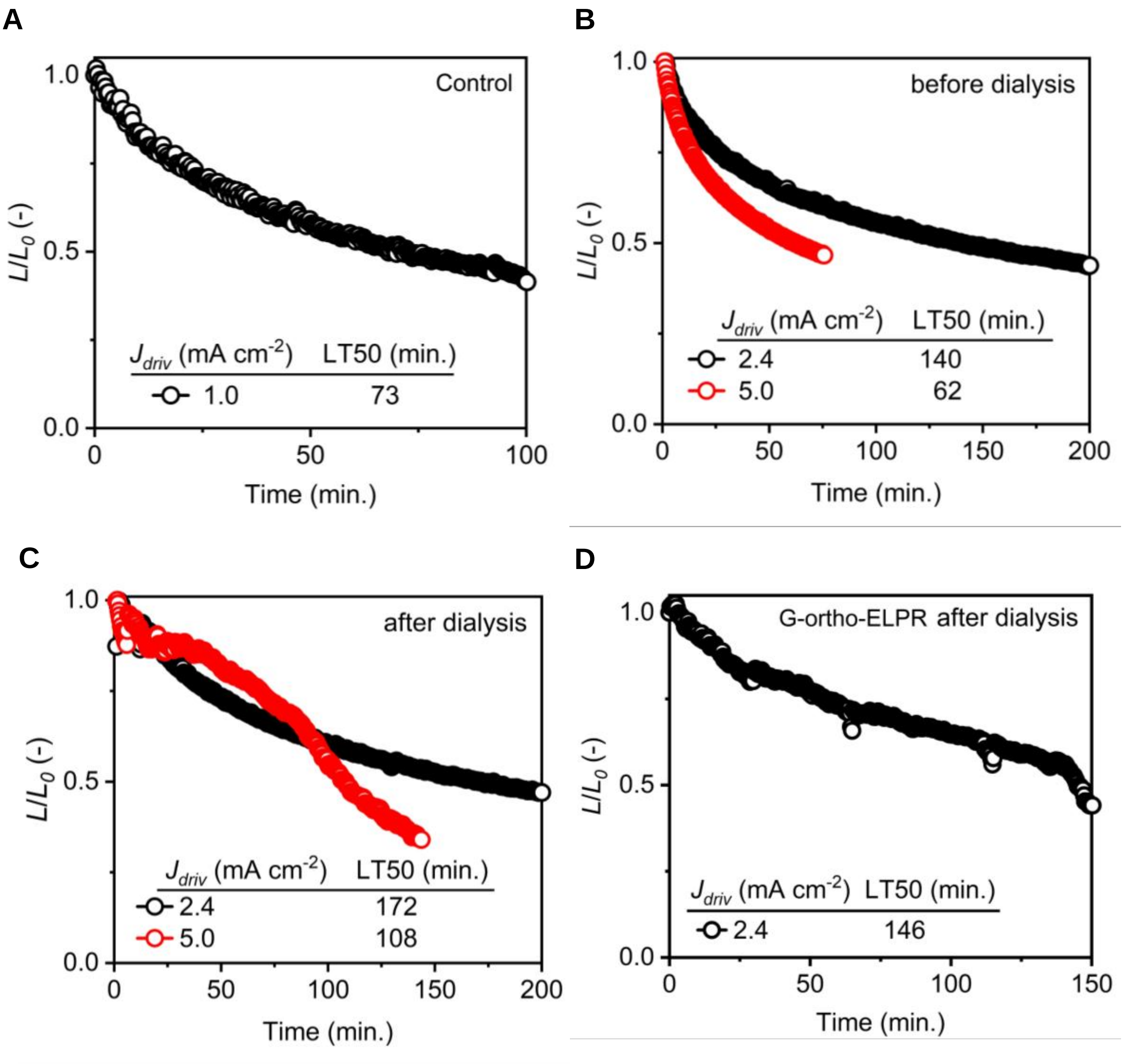

Fig. R2.13 (***Supplementary Fig. 63***) **|** Operational stability of OLED devices. (**A**) Control device architecture of ITO/PTAA/G-*ortho*-P-deBr before dialysis/TmPyPB/Liq/Al. Optimal device with a layer sequence of ITO/v-NPB/G-*ortho*-P-deBr/TPBi/Liq/Al consisting green-emitting self-hosted G-*ortho*-P-deBr TADF polymer emission layer (**B**) before and (**C**) after dialysis purification to reduce copper residue inherited from ATRP synthesis. (**D**) non-crosslinked G-*ortho*-ELPR after dialysis as EML in optimal device.

Comment 2. *The manuscript refers to "full-color" electroluminescence based on the demonstrated RGB ELPR devices. However, the reported EL peaks (approximately 493 nm for blue and 586 nm for red) appear significantly shifted from standard display blue and red primaries. It is therefore unclear whether the present emission characteristics alone justify the use of the term "full color." In addition, the EL spectra shown in the manuscript appear relatively broad, suggesting limited color purity.*

Can the authors provide the CIE 1931 (x, y) color coordinates? How do these values compare with commonly accepted display requirements for color purity and gamut coverage?

Furthermore, the emission wavelengths appear to be largely determined by the choice of the emitter core. The authors are encouraged to clarify the rationale behind selecting the current blue and red emitter cores, and to explain why deeper-blue and deeper-red emitters, which are typically required for display-grade color standards, were not explored in this study. In addition, it would be helpful if the authors could discuss the origin of the relatively broad emission bandwidths observed in the EL spectra. Are these linewidths intrinsic to the ELPR material design or processing conditions? A discussion of the spectral broadening mechanisms and potential pathways to improve color purity would significantly strengthen the manuscript.

We thank the Reviewer for their constructive comment regarding display-grade color standards, as well as their insightful questions on color purity, emitter selection, and spectral broadening. Please find our response as follows.

1. Chromaticity Evaluation and Terminology Revision

The chromaticity coordinates of our demonstrated ELPR thin films are summarized in **Table R2.2 below**: R-ortho-ELPR = (0.51, 0.42), G-ortho-ELPR = (0.21, 0.40), and B-para-ELPR = (0.18, 0.32). For reference, the Digital Cinema Initiatives (DCI-P3) color space has primary coordinates of (0.680, 0.320), (0.265, 0.690), and (0.150, 0.060) for RGB, respectively. Because our ELPR emission coordinates sit closer to the achromatic center, they correspond to ~15% gamut area coverage relative to the DCI-P3 space. We fully agree with the Reviewer that these initial demonstration devices do not yet satisfy commercial display-grade color purity.

Accordingly, we have modified our claim by replacing the term "full-color" with **"multicolor"** throughout the revised manuscript. This change accurately reflects our primary objective: demonstrating the monolithic photolithographic patterning of spectrally distinct red, green, and blue ELPR pixels on a single substrate, rather than claiming a commercial display product.

2. Rationale Behind Chromaticity and Spectral Broadening

The modest gamut coverage reported here is a direct consequence of emitter core selection. Our molecular design prioritized establishing a synthetic baseline for CMOS-compatible OLED miniaturization. To systematically isolate the effects of photolithographic cross-linking from electronic retuning, we utilized a unified donor moiety, 9,9-dimethyl-9,10-dihydroacridine (DMAC), across all three RGB cores, varying solely the acceptor units. Furthermore, DMAC undergoes regioselective bromination at the 2,7-positions via *N*-bromosuccinimide, providing a highly reliable synthetic handle for subsequent Suzuki cross-coupling to install the *ortho*-, *meta*-, and *para*-phenyl linkers. This modular strategy allowed us to preserve a consistent core–shell architecture across the palette, which was vital for validating the universal patternability of the ELPR concept.

The observed spectral broadening arises primarily from the strong charge-transfer (CT) character inherent to traditional donor–acceptor TADF emitters. This intrinsic broadening is further amplified by inhomogeneous matrix effects (e.g., local polarity variations and low-frequency torsional vibrations of the polymer backbone), which enhance vibronic coupling.

Table R2.2 (*Supplementary Table 6*) | Summary of photophysical data of selected polymer thin films with CIE (x, y) color coordinates.

**Polymers (DP[a])**	**End group de-activation?[b]**	**Measured under air or nitrogen?**	**UV crosslinking?[c]**	**λ_{film} (nm)**	**Φ_{film} (%)**	**FWHM (nm)**	**CIE (x, y)[d]**
**G-*ortho*-P** (DP_{ACz} = 45)	N	Air	-	500	72	87	(0.22, 0.43)
	Y	Air	-	498	72	86	(0.21, 0.42)
	Y	Nitrogen	-	500	81	87	(0.21, 0.42)
**G-*ortho*-ELPR** (DP_{ACz} = 45, DP_{MAc} = 12)	N	Air	N	491	36	86	(0.21, 0.38)
	N	Air	Y	491	38	88	(0.21, 0.39)
	N	Nitrogen	N	490	39	86	(0.20, 0.37)
	Y	Air	N	494	34	85	(0.21, 0.40)
	Y	Air	Y	493	37	89	(0.21, 0.40)
	Y	Nitrogen	N	489	38	87	(0.20, 0.36)
	Y	Nitrogen	Y	490	44	87	(0.20, 0.37)
**R-*ortho*-P** (DP_{ACz} = 42)	Y	Nitrogen	-	600	31	113	(0.53, 0.43)
**R-*ortho*-ELPR** (DP_{ACz} = 42, DP_{MAc} = 9)	N	Nitrogen	N	598	24	111	(0.50, 0.41)
	Y	Nitrogen	N	592	25	114	(0.50, 0.41)
	Y	Nitrogen	Y	595	26	112	(0.51, 0.42)
**B-*para*-P** (DP_{ACz} = 50)	Y	Nitrogen	-	482	30	82	(0.18, 0.32)
**B-*para*-ELPR** (DP_{ACz} = 50, DP_{MAc} = 7)	Y	Nitrogen	N	480	14	80	(0.18, 0.30)
	Y	Nitrogen	Y	479	17	85	(0.19, 0.32)

a. DP was determined by ^{1}H-NMR. *b*. Y: End capping C-Br groups are debrominated. N: without end group de-activation *c*. 7000 mJ cm^{-2} of 365 nm UV is exposed to film sample for enough UV crosslinking. *d*. CIE coordinates

3. Solutions Toward High Color Purity and Narrow-Band Emission

While fully decoupling these matrix interactions remains an ongoing effort, we strongly agree that narrowing the emission bandwidth is the most critical next step. To demonstrate that our ELPR platform is fundamentally compatible with high-purity, narrow-band emission, we recently extended this chemistry to multi-resonance TADF (MR-TADF) cores.

As shown in **Figures R2.14 and R2.15 below**, we synthesized two self-hosted MR-TADF polymers (incorporating DtCzB blue and DABNA green cores decorated with our standard ACz host monomers). Remarkably, both systems exhibit sharp electroluminescence with FWHM values of 33 nm (DtCzB) and 29 nm (DABNA). Preliminary gamut projections indicate that substituting our classical TADF blue emitter with this MR-TADF counterpart alone nearly triples the DCI-P3 coverage to 44%. While a full RGB MR-TADF demonstration falls outside the scope of the present study, these preliminary results confirm that the relatively broad spectra reported in this manuscript are a function of first-generation emitter selection, rather than an optical limitation of the photopatternable polymer backbone.

We have updated **Supplementary Table 6** (page 65) and revised the terminology across the text.

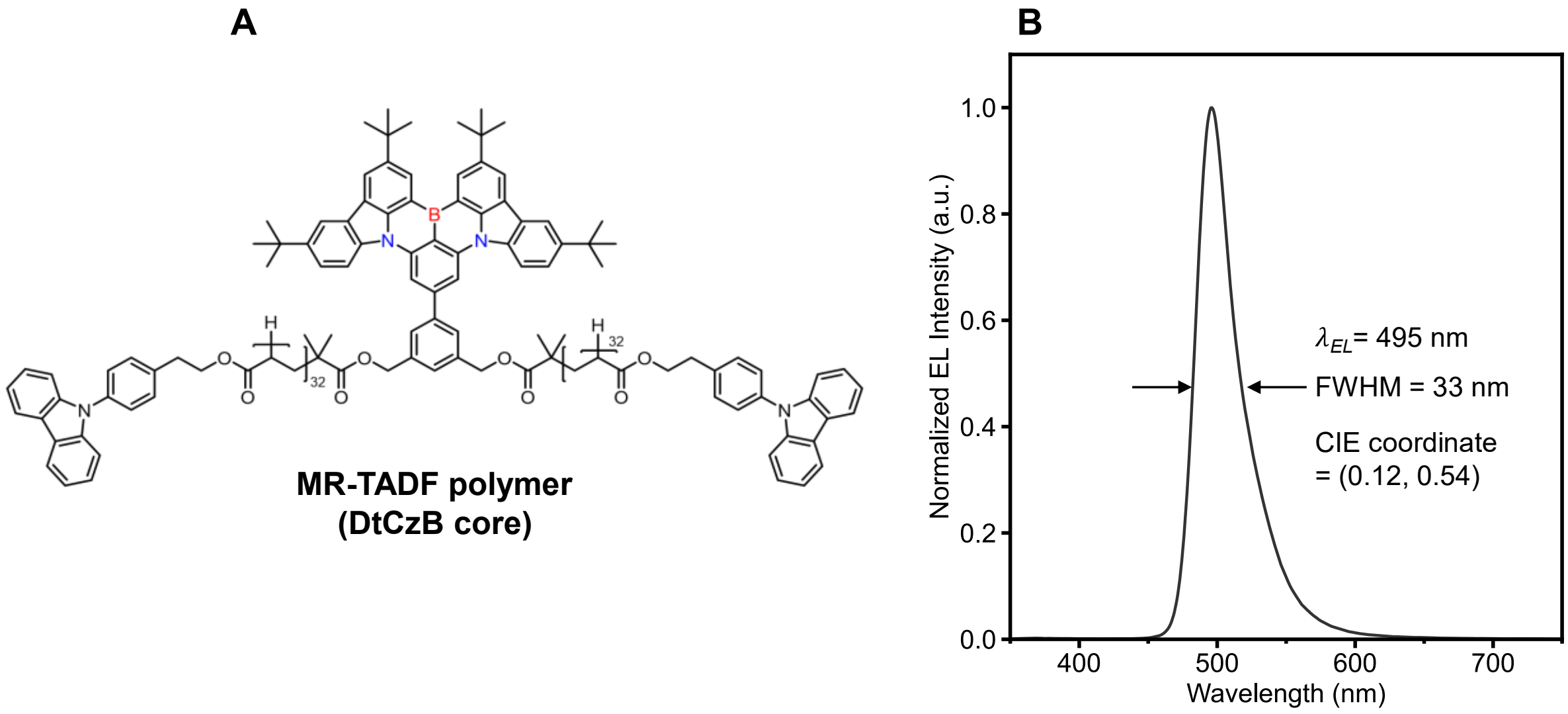

Fig. R2.14 (For revision purposes only) | The self-hosted MR-TADF polymer with narrow band EL recorded from an OLED device. (**A**) The chemical structure of the MR-TADF polymer, where the MR-TADF emitting core (DtCzB) is surrounding by ACz hosts. (**B**) EL spectrum of the OLED device employing this MR-TADF polymer (end group de-activated) as EML with narrow band emission.

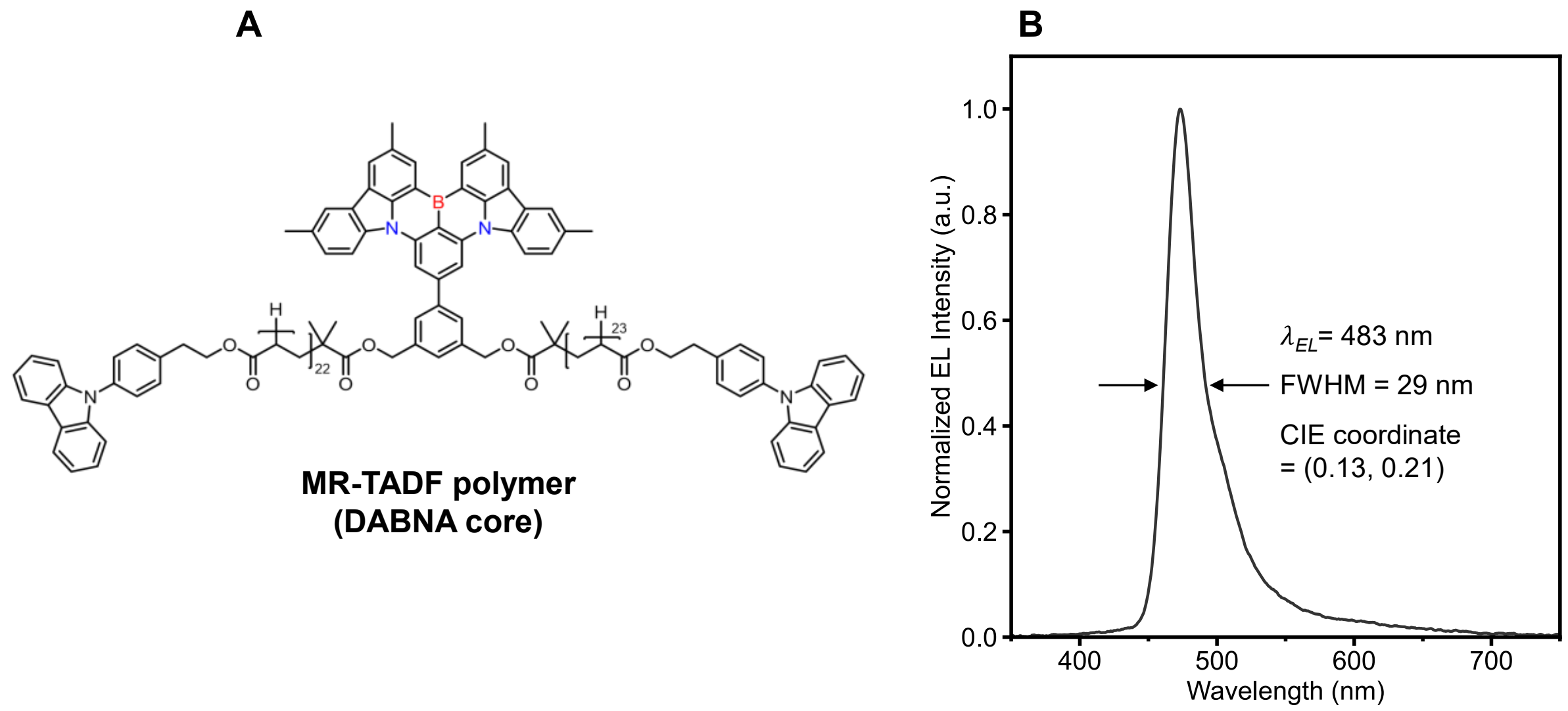

Fig. R2.15 (For revision purposes only) | The self-hosted MR-TADF polymer with narrow band EL recorded from an OLED device. (**A**) The chemical structure of the MR-TADF polymer, where the MR-TADF emitting core (DABNA) is surrounding by ACz hosts. (**B**) EL spectrum of the OLED device employing this MR-TADF polymer (end group de-activated) as EML with narrow band emission.

Comment 3. *Based on the data presented, the high-brightness operating capability of the ELPR OLEDs does not appear to be experimentally demonstrated. While electroluminescence and uniform emission are shown, the manuscript does not provide clear evidence that the devices can sustain the high current densities required for brightness levels relevant to practical OLED or micro-display applications. Can the authors clarify whether the present ELPR material architecture is fundamentally capable of stable operation in the high-current, high-brightness regime? The current manuscript does not appear to provide sufficient experimental evidence demonstrating that brightness can be substantially improved through process or device optimization.*

We thank the Reviewer for pushing us to explore the high-brightness operating regime of our ELPR platform.

To address this point, we first would like to clarify the device design trade-off regarding our original manuscript. The baseline device architecture reported in the first draft (ITO / PEDOT:PSS blend / EML / TmPyPB / Liq / Al) was configured for maximum exciton confinement to benchmark peak EQEs (achieving record values of >30% and 13% for G-ortho-P-deBr and G-ortho-ELPR, respectively). While highly efficient for harvesting singlet/triplet excitons at low drive currents, such high-confinement transport layers inherently introduce carrier injection bottlenecks under high electrical stress.

To verify that the ELPR polymer backbone itself is fundamentally capable of high-luminance operation, we re-engineered the interfacial transport layers to favor carrier throughput over absolute confinement. Remarkably, a re-engineered self-hosted G-ortho-P-deBr device employing PEDOT:PSS (AI 4083) as the HIL and v-NPB as the HTL safely sustained a high current operation up to 360 mA cm^{-2}, yielding an unprecedented peak luminance of 8,105 cd m^{-2} (**Fig. R2.16 below**). We also confirm that our designs of dual-functional polymer chains do not suffer from Joule-heating-induced thermal meltdown or intrinsic dielectric breakdown in continuous operation at a high current of 10 mA cm^{-2} (**Fig. R2.17 below**).

A parallel study was carried out for the G-ortho-ELPR system. By incorporating a PEDOT:PSS/PVK bilayer HTL, the maximum luminance was enhanced to 1,392 cd m^{-2} at 60.6 mA cm^{-2} (**Fig. R2.18 below**). Furthermore, an alternative stack (PEDOT:PSS AI 4083 + TPBi ETL) pushed the peak luminance to 2,068 cd m^{-2} at 126.2 mA cm^{-2} (**Fig. R2.19 below**).

In summary, these data provide strong evidence that the perceived low-brightness ceilings in our original text result from transport-layer selection. Once freed from injection barriers, the ELPR polymer architecture robustly supports the high-current, high-brightness regimes, thereby confirming the relevance of ELPRs to practical display technologies.

We have added **Supplementary Fig. 64-67** on page 87-90 to the Supplementary information in the **Supplementary note 22**.

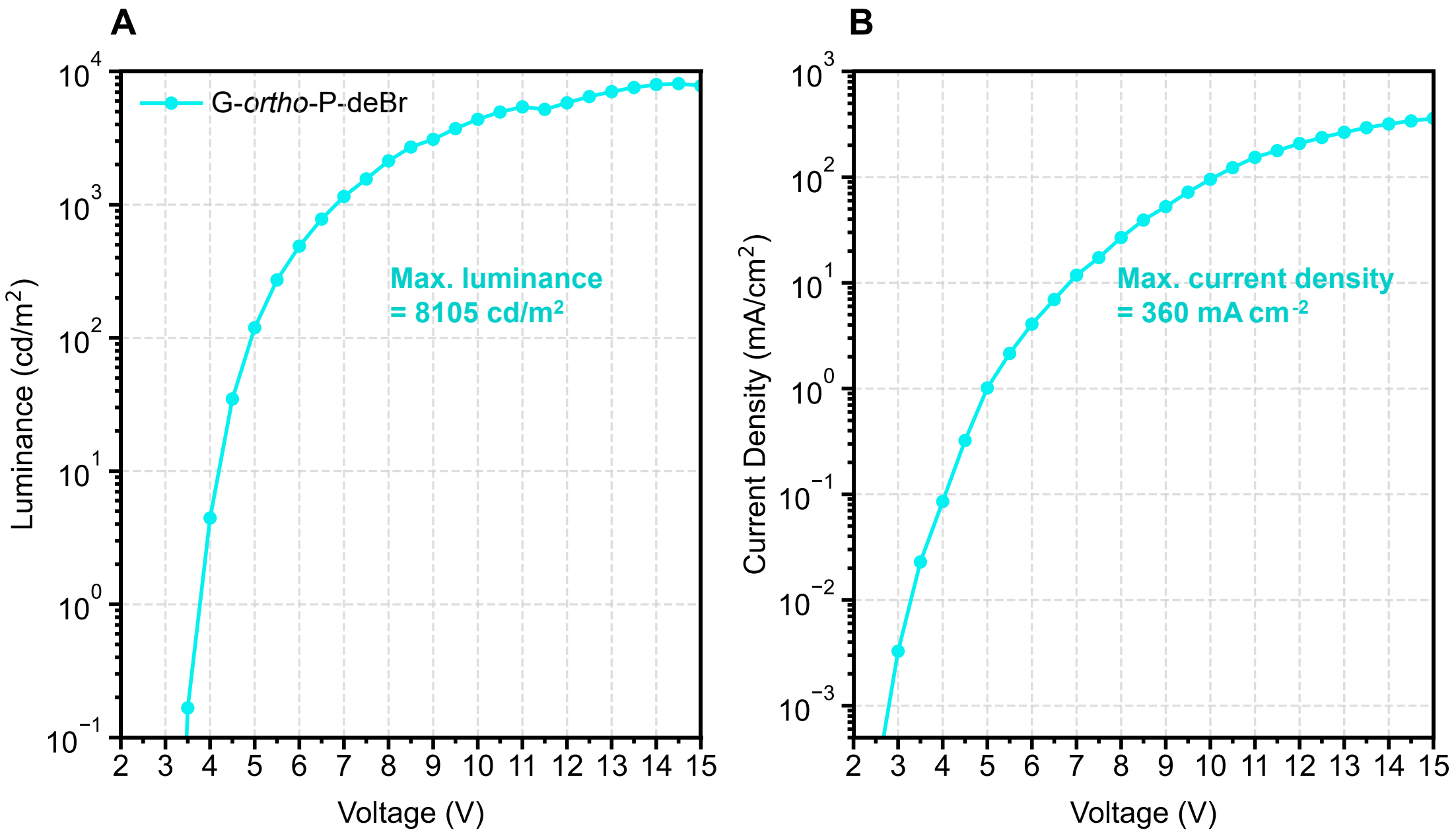

Fig. R2.16 (*Supplementary Fig. 64*) | Luminance–voltage (**A**) and current density-voltage (**B**) characteristics of the OLED device employing G-*ortho*-P-deBr as EML. Device architecture: ITO/PEDOT:PSS (Al 4083)/v-NPB/G-*ortho*-P-deBr/TPBi/Liq/Al. The device exhibits a maximum brightness of 8,105 cd m^{-2} and a peak current density of 360 mA cm^{-2} at a driving voltage of 15 V.

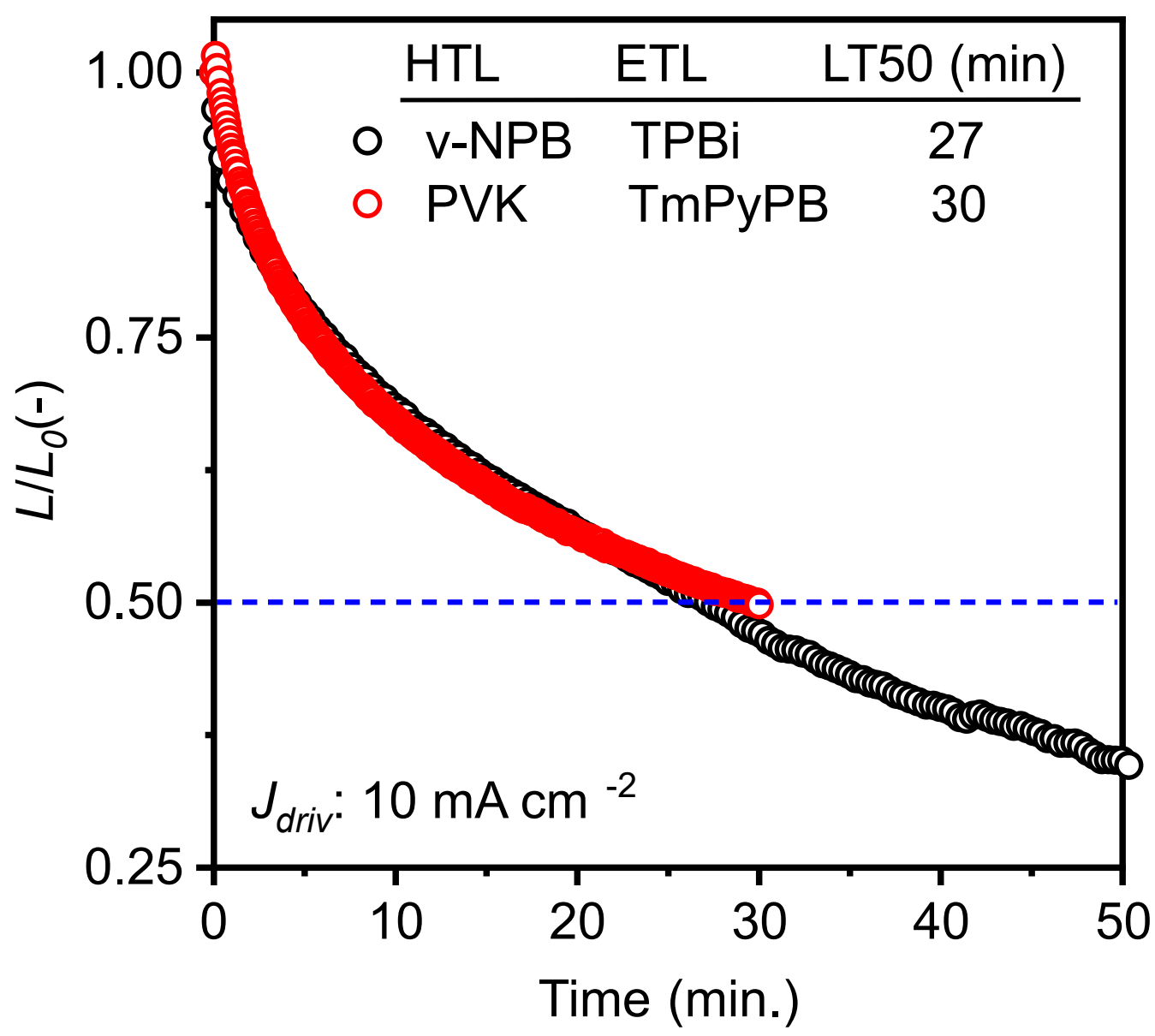

Fig. R2.17 (*Supplementary Fig. 65*) | Operational lifetime of OLED devices consisting green-emitting self-hosted G-*ortho*-P-deBr TADF polymer operated at a constant J_{driv} of 10 mA cm^{-2}. Device architectures: ITO/v-NPB/G-*ortho*-P-deBr/TPBi/Liq/Al (black dots) and ITO/NiOx/PVK/G-*ortho*-P-deBr/TmPyPB/Liq/Al (red dots).

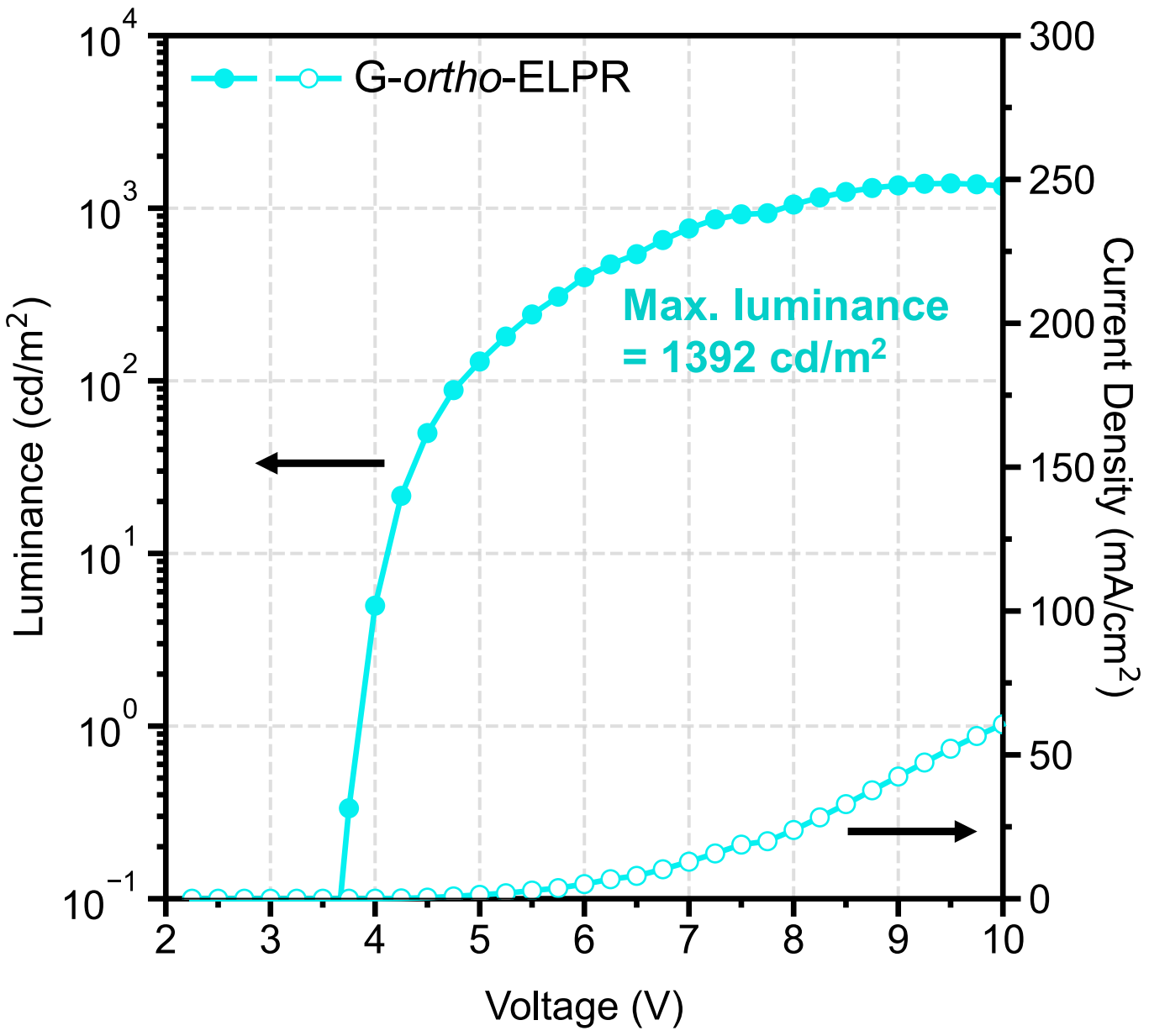

Fig. R2.18 (*Supplementary Fig. 66*) | Luminance–voltage characteristic of the OLED device employing G-*ortho*-P-ELPR as EML. Device architecture: ITO/PEDOT:PSS (AI 4083)/PVK/EML/TmPyPB/Liq/Al.

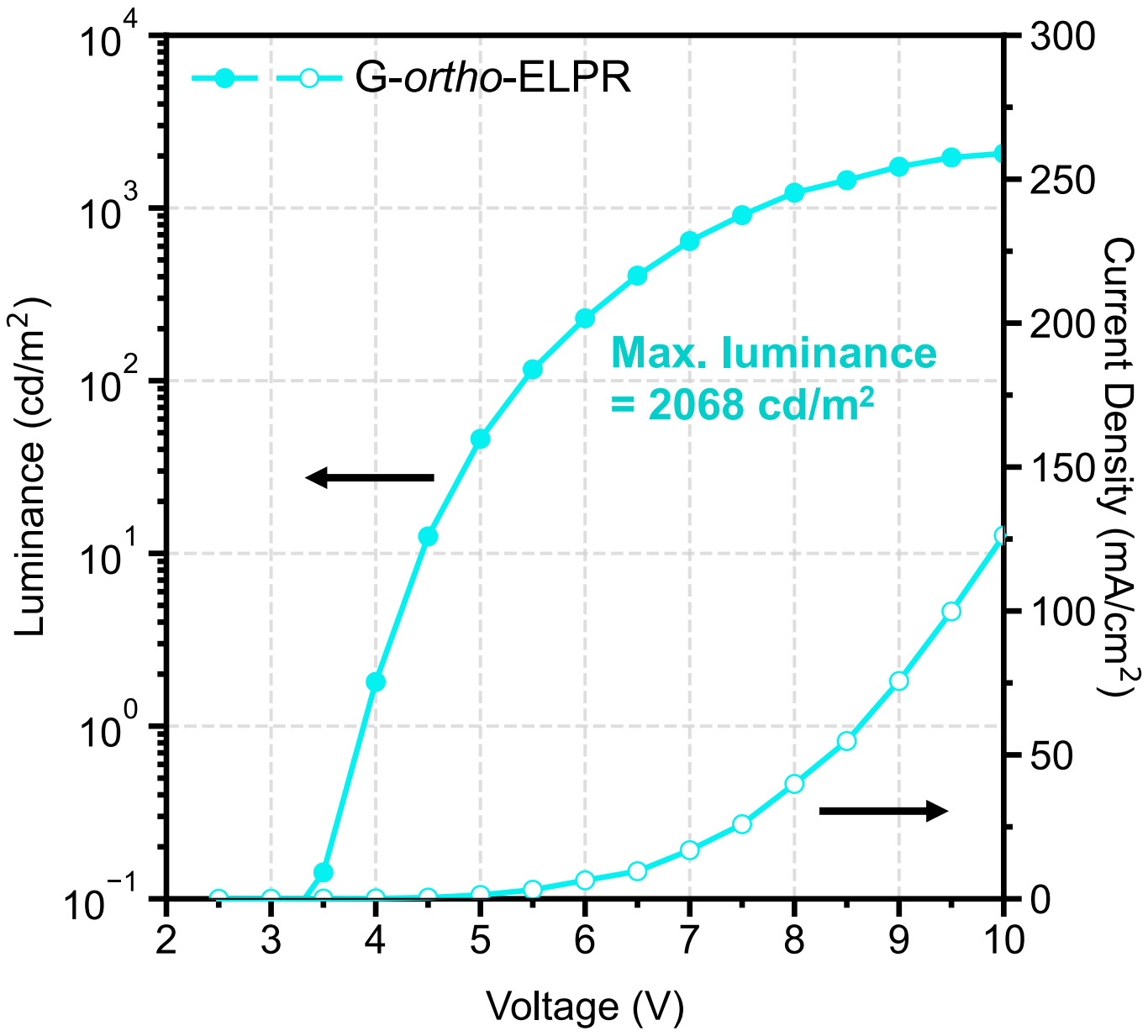

Fig. R2.19 (***Supplementary Fig. 67***) **|** Luminance–voltage characteristic of the OLED device employing G-*ortho*-P-ELPR as EML. Device architecture: ITO/PEDOT:PSS (AI 4083)/EML/TPBi/Liq/Al.

Comment 4. *The J–V and L–V characteristics indicate that current density and luminance do not continue to increase at higher driving voltages, and in some regimes appear to saturate or decrease. The manuscript does not explicitly address the physical origin of this behavior.*

Can the authors clarify whether this high-voltage response is associated with irreversible current-induced degradation, charge imbalance and transport limitations, or other exciton-related quenching processes in the ELPR OLEDs? Importantly, do the authors consider this behavior to be an intrinsic limitation of the ELPR material architecture under high current injection, or a reversible effect that can be mitigated through device or materials optimization.

We thank the Reviewer for the insightful question. As will be detailed later, our findings suggest that the high-voltage saturation observed in our original draft originates predominantly from reversible charge-injection and interfacial transport, rather than irreversible current-induced degradation or an intrinsic thermodynamic limit of the ELPR polymer architecture.

Specifically, our experimental evidence can be summarized as follows.

1. Mitigation of Current Saturation

As demonstrated in our earlier response to the Reviewer's **Comment 3**, replacing the baseline high-confinement stack with an injection-optimized architecture allowed the representative G-ortho-P-deBr device to sustain a monotonic upward driving trajectory up to 360 mA cm^{-2} (8,105 cd m^{-2}) without early saturation.

Regarding the **G-ortho-P-ELPR** system discussed here, when the baseline stack (ITO/PEDOT:PSS blend/EML/TmPyPB) was re-engineered by introducing either PVK as the HTL or TPBi as the ETL (ITO/PEDOT:PSS/PVK/EML/TmPyPB and ITO/PEDOT:PSS/EML/TPBi), the devices not only delivered substantially enhanced J and L, but also maintained a monotonic increase across the entire driving-voltage range, showing no sign of high-voltage roll-over **(Figs. R2.18 and R2.19 above).**

2. Direct Evidence of Intrinsic Dielectric Robustness

To explicitly rule out irreversible Joule-heating or current-induced chemical degradation, we subjected the ELPR pixels to rigorous electrical stress protocols:

- **Repeated Bias Stressing:** We performed four consecutive J-V scans on the exact same OLED pixel between –5 V and +7 V. All four sweep profiles overlapped virtually identically (**Fig. R2.20 below**), confirming the robust electrochemical stability of the cross-linked polymer network under repeated high-field injection.

- **Hysteresis Evaluation:** J-V characteristics recorded under forward and reverse voltage sweeps exhibited near-perfect congruence across several orders of magnitude (**Fig. R2.21 below**). The negligible hysteresis confirms the absence of severe bias-induced ion migration or deep, permanent carrier trapping within the emissive layer.

The observed saturation in our first draft can be attributed to the large injection barriers of the baseline stack. These barriers induced severe space-charge build-up at elevated biases, which partially screened the external electric field (limiting J) and promoted field-induced exciton quenching (limiting L).

In summary, these data confirm that the high-voltage behavior reported in the original text was a direct consequence of our first-generation transport-layer selection. Once interfacial charge balance is established, the ELPR polymer backbone robustly supports stable high-bias operation. We have incorporated this discussion into the revised manuscript.

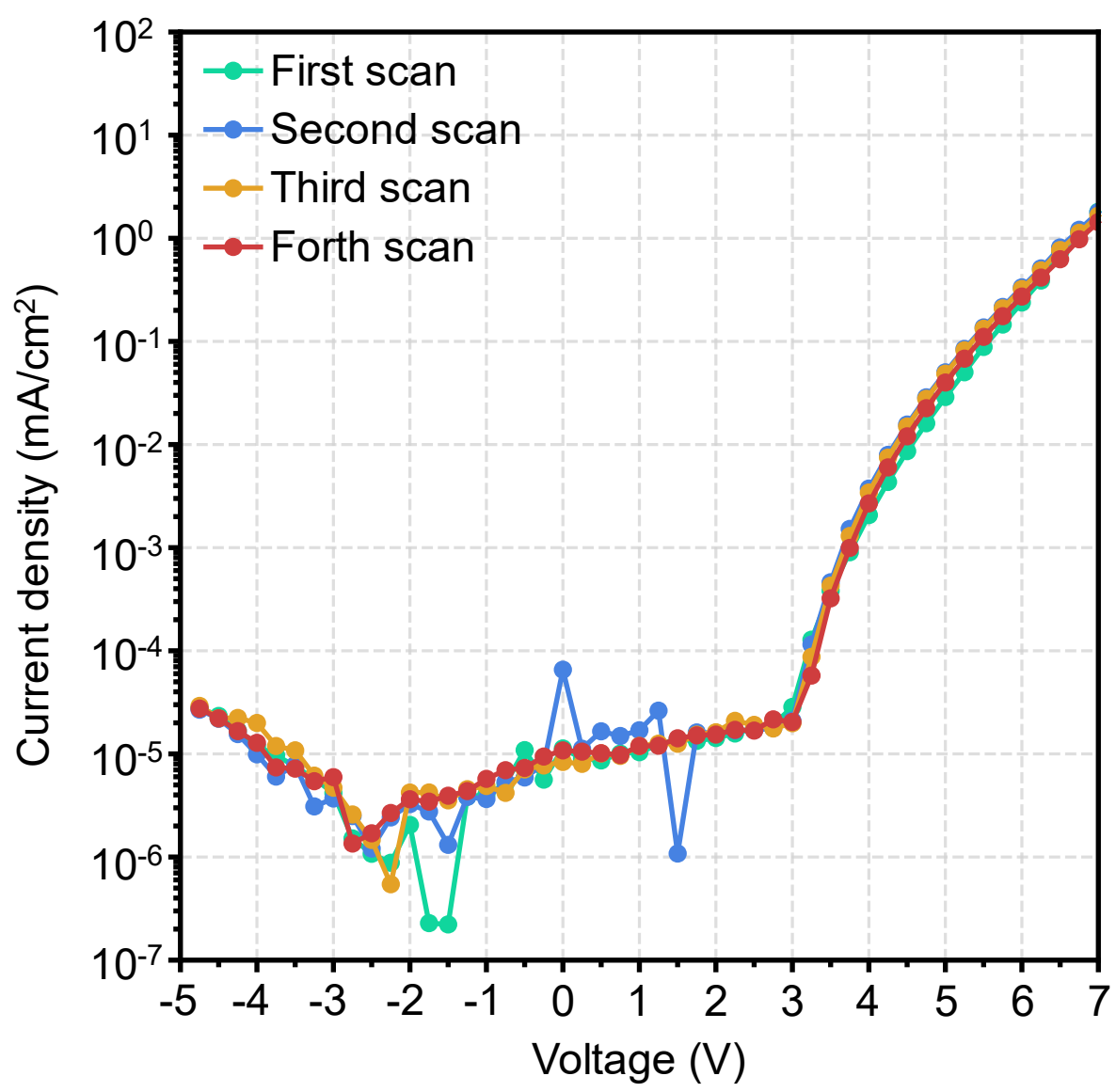

Fig. R2.20 (***Supplementary Fig. 57***) **|** Four consecutive *J-V* scans of the G-*ortho*-P-deBr OLED measured by sweeping the voltage from −1 V to 10 V. The scans overlap closely over both the negative and positive voltage ranges, with only modest fluctuations between repeats.

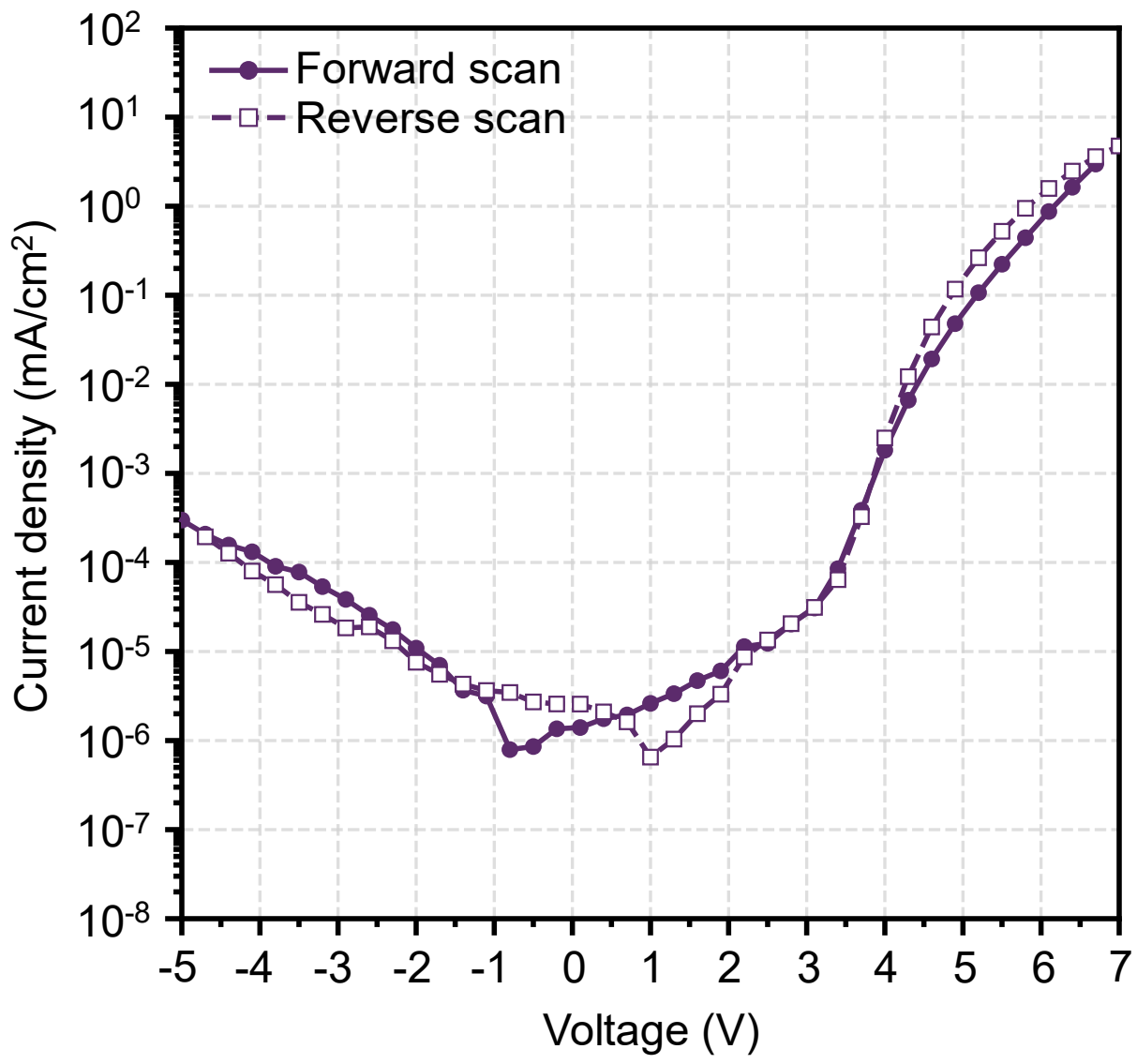

Fig. R2.21 (***Supplementary Fig. 58***) **|** Forward and reverse scans of *J*-*V* curves for the G-*ortho*-P-deBr OLED.

Comment 5. *End-group deactivation leads to a dramatic improvement in device efficiency and current efficiency, while the emissive core structure remains unchanged. This suggests that the performance gain may arise primarily from reduced trap-assisted losses or improved film quality rather than from intrinsic changes to the emitter.*

Can the authors clarify to what extent end-group deactivation also improves tolerance to high current injection, high-brightness operation, and operational stability? Is there experimental evidence that deactivation mitigates current-induced degradation or charge-transport limitations, beyond the observed efficiency enhancement at low to moderate operating conditions.

In addition, while the deactivation effect is prominently demonstrated for the green emitter system, it is less clear how consistently this improvement translates to the red and blue ELPRs. Can the authors clarify whether similar trap-related performance enhancement is observed across all RGB systems, and if not, what governs the color-dependent response to end-group deactivation?

We thank the Reviewer for this thoughtful and constructive comment, which prompted us to examine the fundamental impact of end-group deactivation on high-current operation, as well as the universality of this strategy across different ELPR colors. We have performed new experiments and analysis to fully address the Reviewer's concerns. Please find the details as follows.

1. Physical Mechanism of End-Group Deactivation (Impedance Spectroscopy)

To explicitly clarify how end-group deactivation mitigates charge-transport limitations, we carried out impedance spectroscopy (IS) on the OLEDs of identical device architecture, only differing in their emissive layers (G-ortho-P-Br vs. deactivated G-ortho-P-deBr). Measurements were conducted under forward DC biases of 5.0 V and 5.5 V with a 70 mV AC perturbation over a 1 Hz to 1 MHz frequency range.

As shown in the Nyquist plots recorded at 5.0 V (**Figs. R2.22 and R2.23 below**), the brominated G-ortho-P-Br device exhibits an extremely large, non-closing arc with imaginary impedance values exceeding 10^5 Ω toward the low-frequency region, indicating severely hindered, trap-limited charge transport. In contrast, the deactivated G-ortho-P-deBr device exhibits a well-defined, fully closed semicircle with a drastically reduced diameter.

The Nyquist data were fitted using the equivalent circuit model, R_s – (R_1||CPE_1) – (R_2||CPE_2). In this framework, R_s reflects the ohmic losses (electrodes and fast transport, not directly related to trap states); R_1 and CPE_1 represent the injection resistance and its associated non-ideal capacitance; and R_2 and CPE_2 describe the recombination-related resistance and capacitance. Lower R_1 and R_2 values correspond to more efficient carrier injection and radiative recombination, respectively.

The extracted fitting parameters (**Table R2.3**) reveal a clear trend: the end-group deactivation reduces the injection resistance R_1 by over four orders of magnitude, from 1.67 x 10^7 Ω (G-*ortho*-P-Br) to 1.58 x 10^3 Ω (G-*ortho*-P-deBr). Simultaneously, the recombination resistance (R_2) is reduced by a comparable factor, from 5.85 × 10^6 Ω to 2.17 x 10^3 Ω. Furthermore, the Bode phase analysis (**Fig. R2.24**) confirms that the deactivated device exhibits at least a two-order-of-magnitude reduction in low-frequency (1 Hz) impedance.

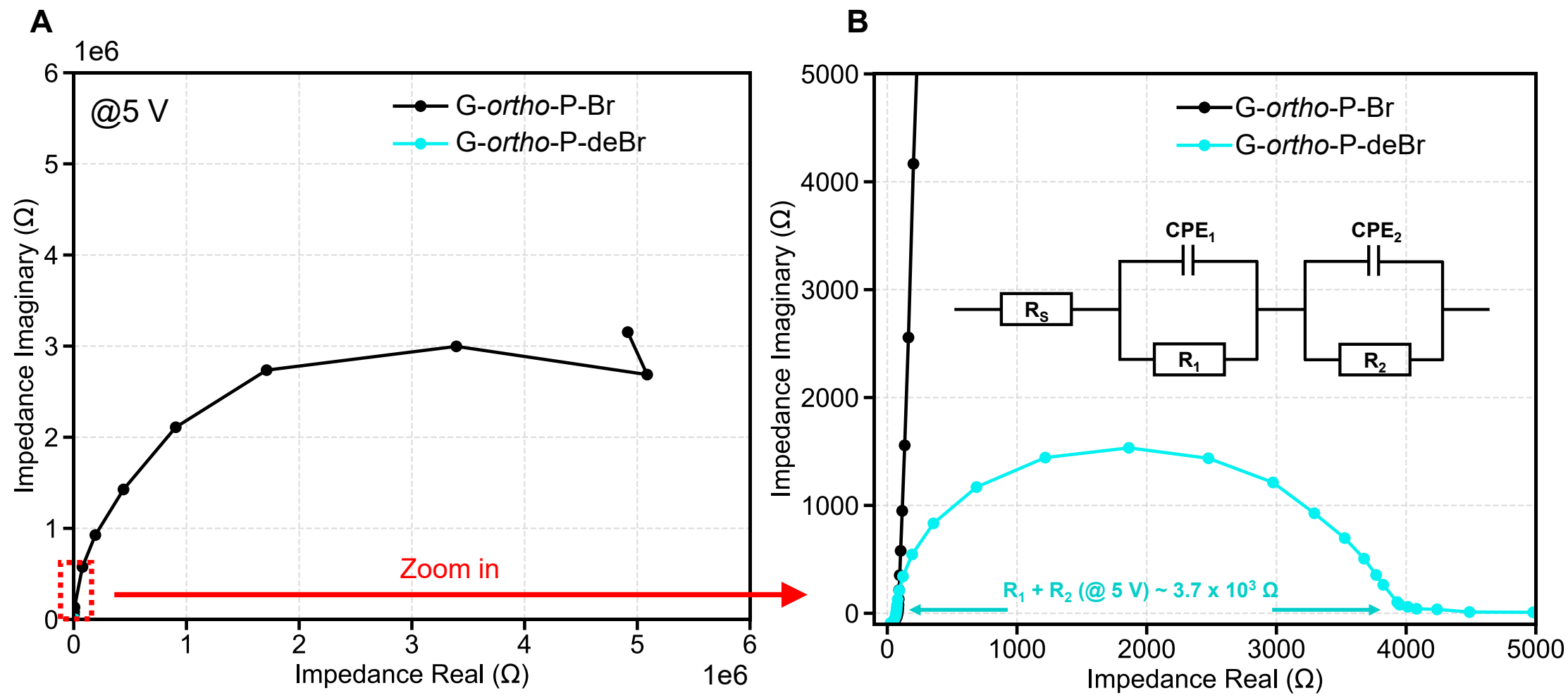

Fig. R2.22 (***Supplementary Fig. 68***) **|** Nyquist plots of the OLED devices employing either G-*ortho*-P-Br or G-*ortho*-P-deBr as EML recorded at 5 V over 1 Hz to 1 MHz frequency range, showing the impact of end-group deactivation on charge-transport resistances. (**A**) The overview Nyquist plot. (**B**) The same Nyquist plot but zoom-in at the high frequency region. Inset of (**B**): The equivalent circuit model used to extract the charge-transport fitting parameters. The brominated device (G-*ortho*-P-Br, black) exhibits an extremely large, non-closing arc, indicative of severely hindered charge transport before end-group deactivation. In contrast, the deactivated/debrominated device (G-*ortho*-P-deBr, cyan) shows a well-defined, closed and smaller-diameter semicircle. Both the injection resistance (R_1) and recombination resistance (R_2) were significantly reduced after end group de-activation and the semicircle diameter has real impedance of $R_1 + R_2 \sim 3.7$ kΩ. Device architecture: ITO/PEDOT:PSS blend (Al 4083/CH 8000 mix)/EML/TmPyPB/Liq/Al.

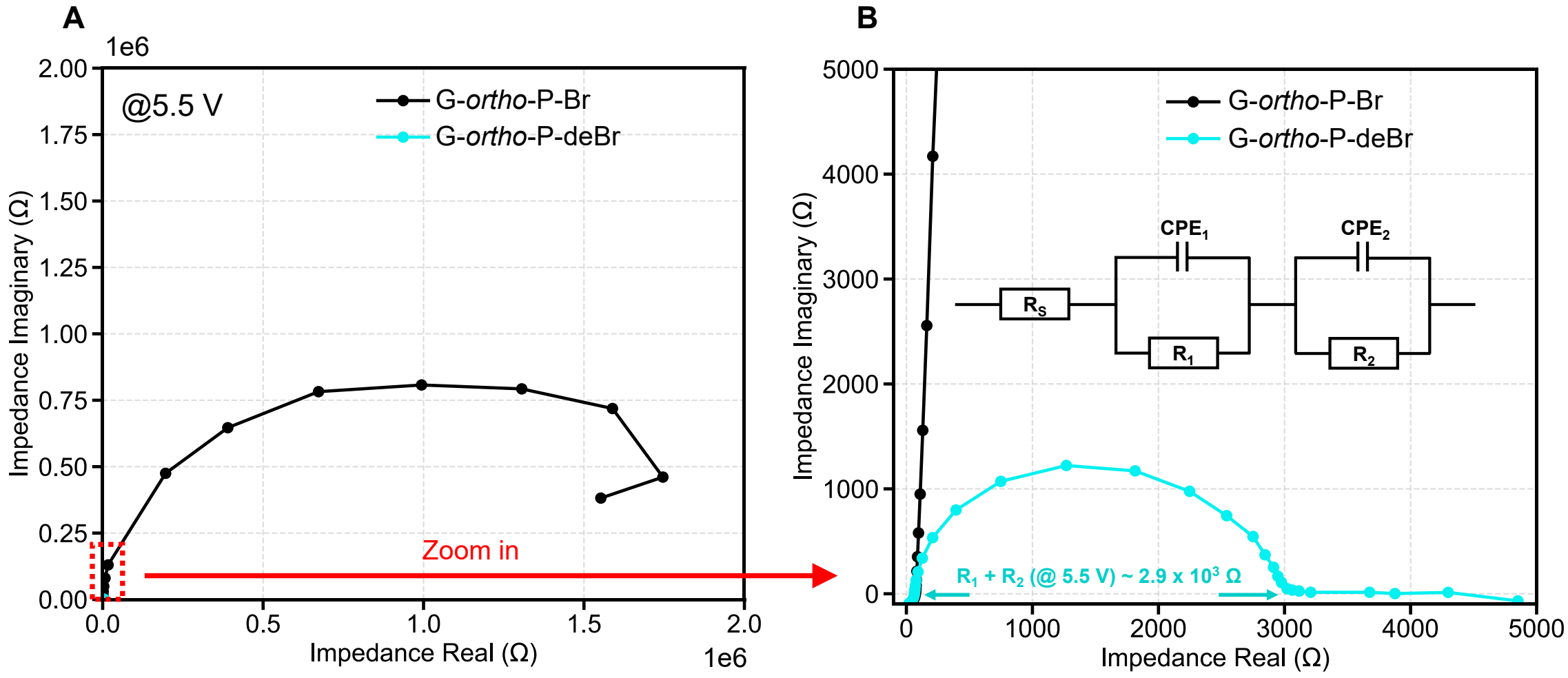

Fig. R2.23 (***Supplementary Fig. 69***) **|** Nyquist plots of the OLED devices employing either G-*ortho*-P-Br or G-*ortho*-P-deBr as EML recorded at 5.5 V over 1 Hz to 1 MHz frequency range, showing the impact of end-group deactivation on charge-transport resistances. (**A**) The overview Nyquist plot. (**B**) The same Nyquist plot but zoom-in at the high frequency region. Inset of (**B**): The equivalent circuit model used to extract the charge-transport fitting parameters. The brominated device (G-*ortho*-P-Br, black) exhibits an extremely large, non-closing arc, indicative of severely hindered charge transport before end-group deactivation. In contrast, the deactivated/debrominated device (G-*ortho*-P-deBr, cyan) shows a well-defined, closed and smaller-diameter semicircle. Both the injection resistance (R_1) and recombination resistance (R_2) were significantly reduced after end group de-activation and the semicircle diameter has real impedance of $R_1 + R_2 \sim 2.9$ kΩ. Device architecture: ITO/PEDOT:PSS blend (Al 4083/CH 8000 mix)/EML/TmPyPB/Liq/Al.

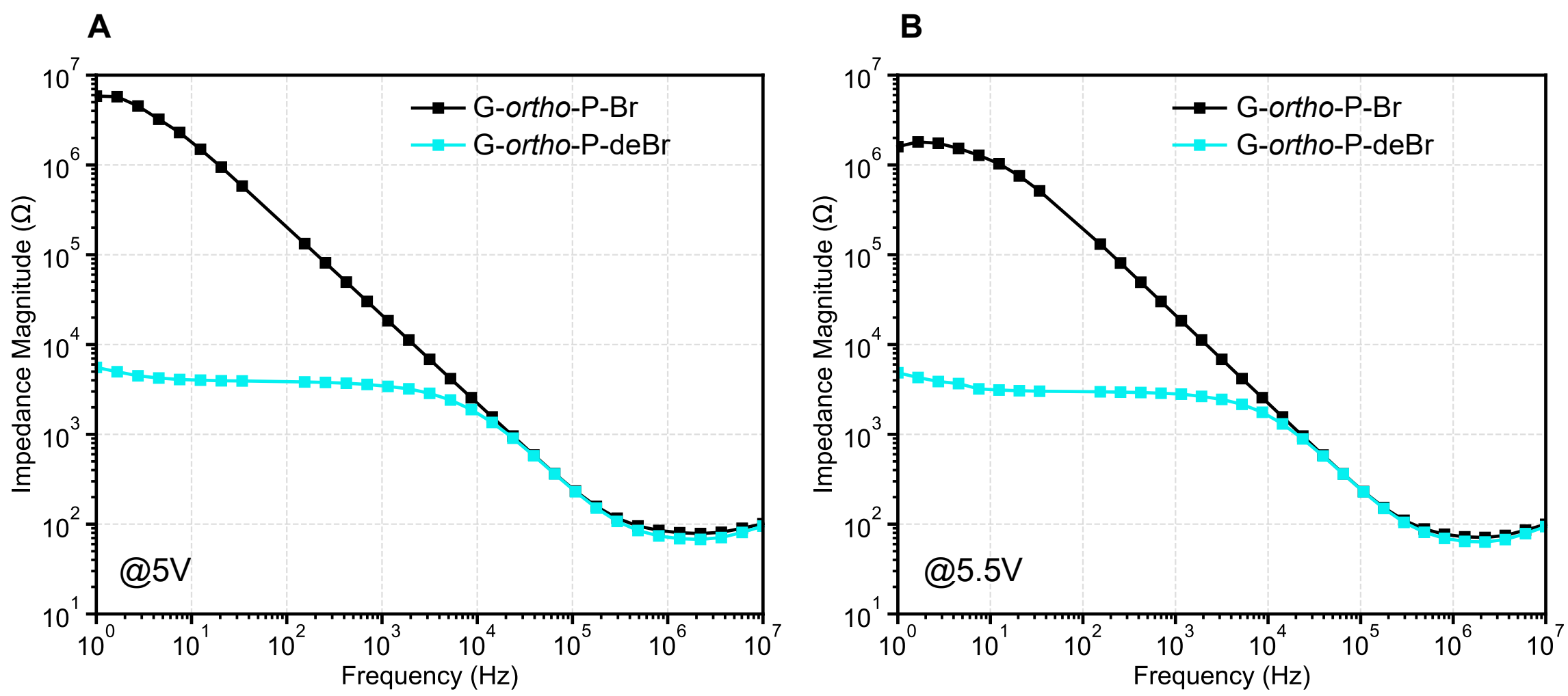

Fig. R2.24 (***Supplementary Fig. 70***) **|** Impedance – frequency response spectra of the OLED devices employing G-*ortho*-P-Br (black squares) or G-*ortho*-P-deBr (cyan squares) as EML under forward DC bias either 5 V (**A**) or 5.5 V (**B**). The pronounced reduction of low-frequency impedance (close to 1 Hz) for G-*ortho*-P-deBr compared to G-*ortho*-P-Br case indicates more efficient charge transport after end group de-activation.

Table R2.3 (***Supplementary Table 10***) | Summary of the fitting parameters of the Nyquist plots extracted from the equivalent circuit model for the OLED devices employing either G-*ortho*-P-Br or G-*ortho*-P-deBr as EML. Both the injection resistance and recombination resistance were significantly reduced after end group de-activation.

**Measurement condition**	**DC bias at 5V with 70 mV AC perturbation**		**DC bias at 5.5V with 70 mV AC perturbation**	
**End group de-activation?** [a] **(for G-ortho-P)**	N	Y	N	Y
**R_s**[b] **(Ω)**	82.5	71.0	74.5	67.6
**R_1**[c] **(Ω)**	1.67×10^7	1.58×10^3	9.96×10^5	1.17×10^3
**CPE_1**[d] **($s^{\alpha}\ \Omega^{-1}$)**	3.83×10^{-7}	8.23×10^{-8}	1.12×10^{-8}	4.30×10^{-8}
**α_1**[e] **(-)**	0.94	0.95	0.98	0.99
**R_2**[f] **(Ω)**	5.85×10^6	2.17×10^3	1.14×10^6	1.68×10^3
**CPE_2**[g] **($s^{\alpha}\ \Omega^{-1}$)**	9.19×10^{-9}	9.18×10^{-9}	4.37×10^{-8}	8.62×10^{-9}
**A_2**[h] **(-)**	0.98	1.00	0.99	1.00

a. Y: End capping C-Br groups are debrominated. N: without end group de-activation. *b*. R_s: contact resistance. *c*. R_1: injection resistance. *d*. CPE_1: constant phase element describing the non-ideal injection capacitance. *e*. α_1: dimensionless non-ideality parameter of CPE_1; where $\alpha_1 < 1$ for non-ideal capacitor case. *f*. R_2: recombination resistance. g. CPE_2: constant phase element describing the non-ideal recombination capacitance. *h*. α_2: dimensionless non-ideality parameter of CPE_2; where $\alpha_2 < 1$ for non-ideal capacitance case.

2. Macroscopic Translation to High-Brightness Tolerance and Operational Stability

This profound reduction in impedance provides direct physical evidence that end-group deactivation effectively passivates charge-trapping sites, thereby mitigating current-induced space-charge accumulation. Indeed, this fundamental suppression of trap-assisted losses directly translates to the following aspects in terms of the macroscopic device robustness:

- **Tolerance to Extreme Current Injection:** The new discussion and analysis addressing the Reviewer's Comment 3 precisely correspond to deactivated G-ortho-P-deBr polymers that safely sustained extreme electrical driving up to 360 mA cm^{-2}, yielding an unprecedented peak luminance of 8,105 cd m^{-2} **(Fig. R2.16 above)**.

- **Operational Robustness Under High Stress:** Under continuous DC driving, the deactivated OLED achieved an LT_{50} of 108 min at 5 mA cm^{-2}, which scaled robustly to 27–30 min under 10 mA cm^{-2} (**Figs. R2.13 and R2.17**).

Accordingly, these results verify that end-group deactivation does not only boost low-current radiative efficiency, but also fundamentally passivates the dual-functional polymer backbone against field-induced thermal meltdown and trap-assisted chemical degradation under display-relevant operating conditions.

3. Universality Across the Full RGB Polymers

Finally, to explicitly verify the general applicability of this debromination strategy, we extended the end-group deactivation protocol beyond our baseline green **G-*ortho*-P** system to the red (**R-*ortho*-P**), blue (**B-*para*-P**), and another isomeric green (**G-*para*-P**) self-hosted polymers (**Figs. R2.25-R2.27 below**).

Across all systems considered here, the end-group deactivation revealed consistent and unambiguous device enhancements, as reflected in the substantially enhanced peak luminances, current efficiencies, and maximum EQEs. These results confirm that the performance gains unlocked by passivating trap-limited conduction are fundamentally intrinsic to our dual-functional polymer design, rather than an idiosyncratic artifact of a specific emissive core. All of these figures have been included in the revised SI.

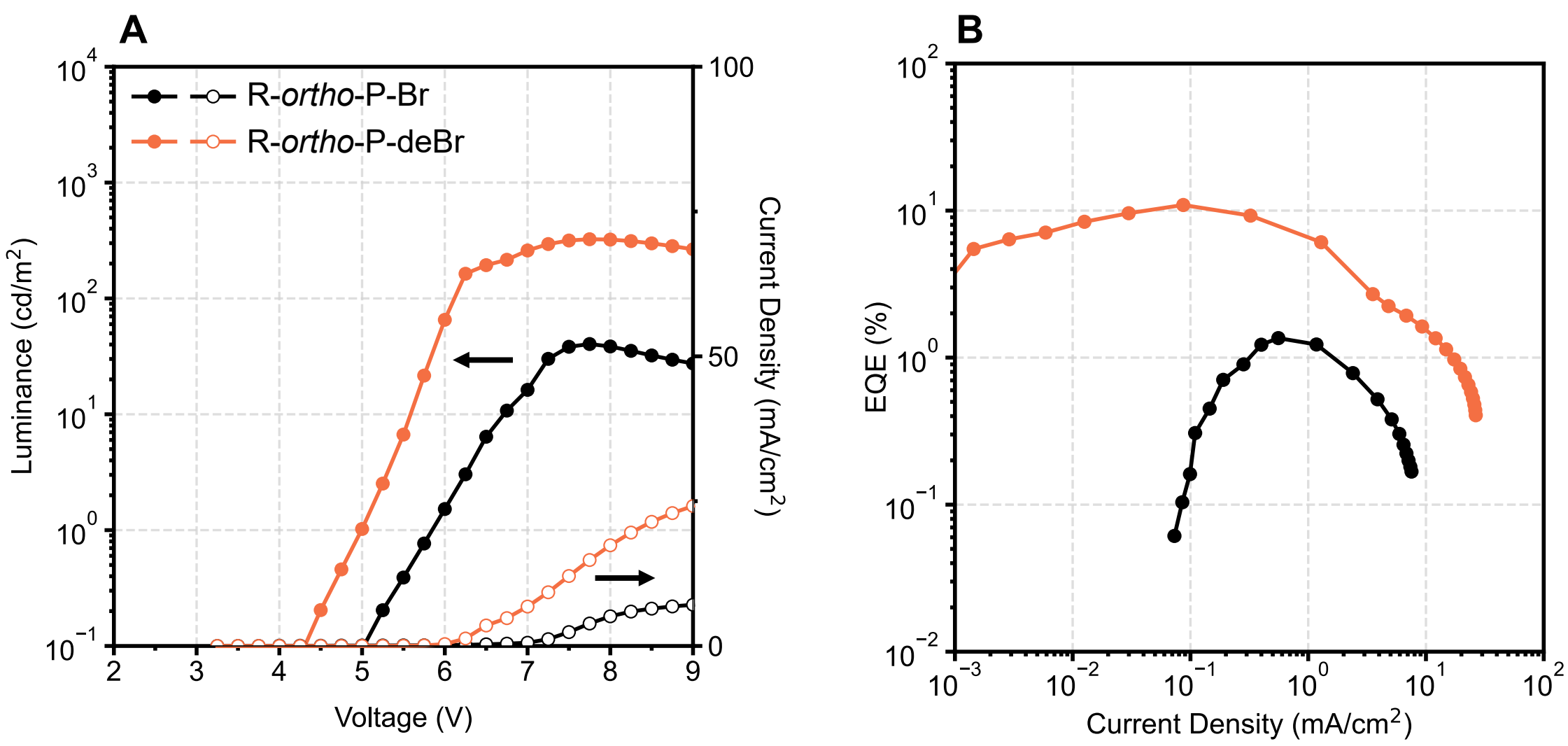

Fig. R2.25 (*Supplementary Fig. 71*) | Luminance–voltage (A) and EQE-current density (B) characteristics of the OLED device employing either R-*ortho*-P-Br (black) or R-*ortho*-P-deBr (orange) as EML. Device architecture: ITO/PEDOT:PSS blend (AI 4083/CH 8000 mix)/EML/TmPyPB/Liq/Al.

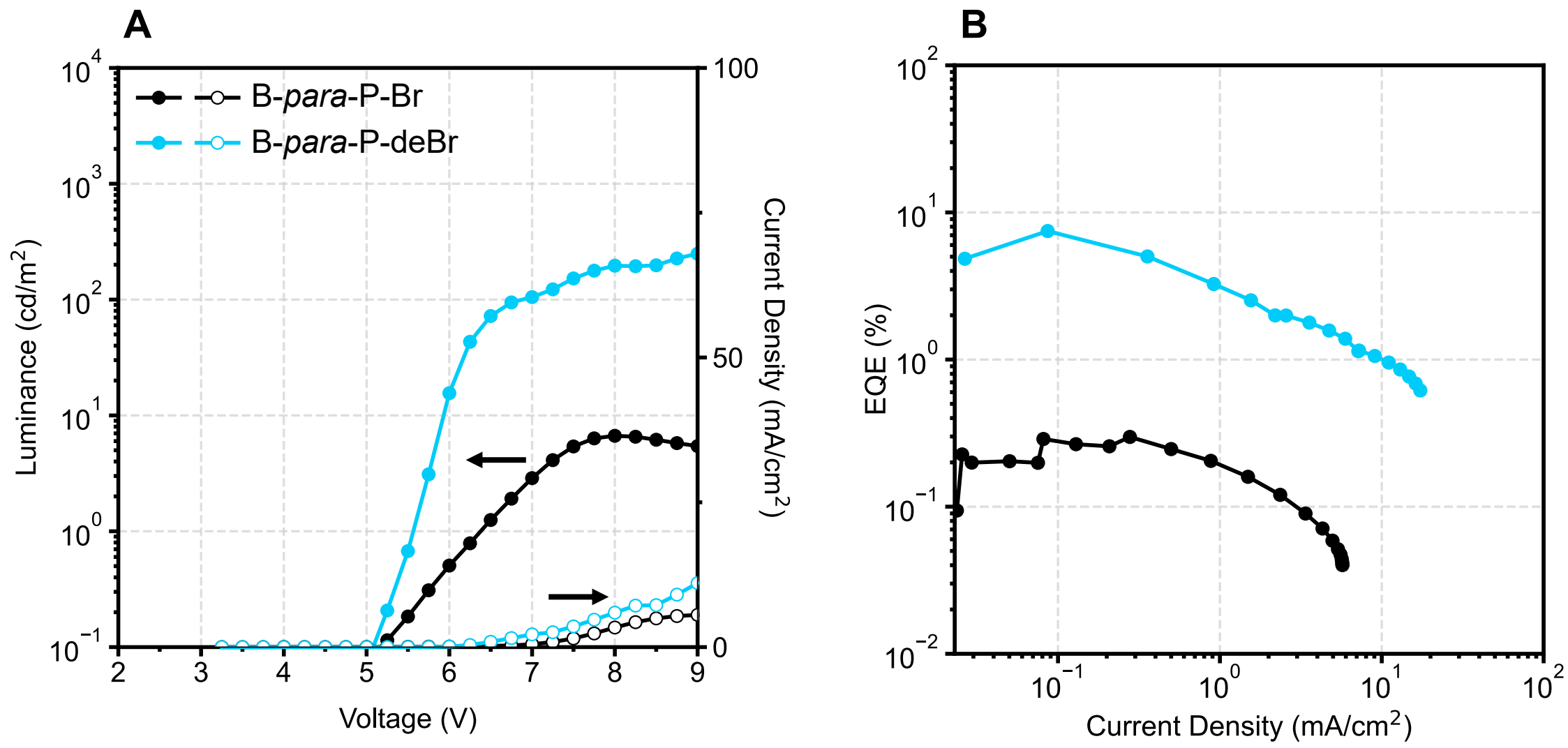

Fig. R2.26 (***Supplementary Fig. 72***) **|** Luminance–voltage (**A**) and EQE-current density (**B**) characteristics of the OLED device employing either B-*para*-P-Br (black) or B-*para*-P-deBr (blue) as EML. Device architecture: ITO/PEDOT:PSS blend (AI 4083/CH 8000 mix)/EML/TmPyPB/Liq/Al.

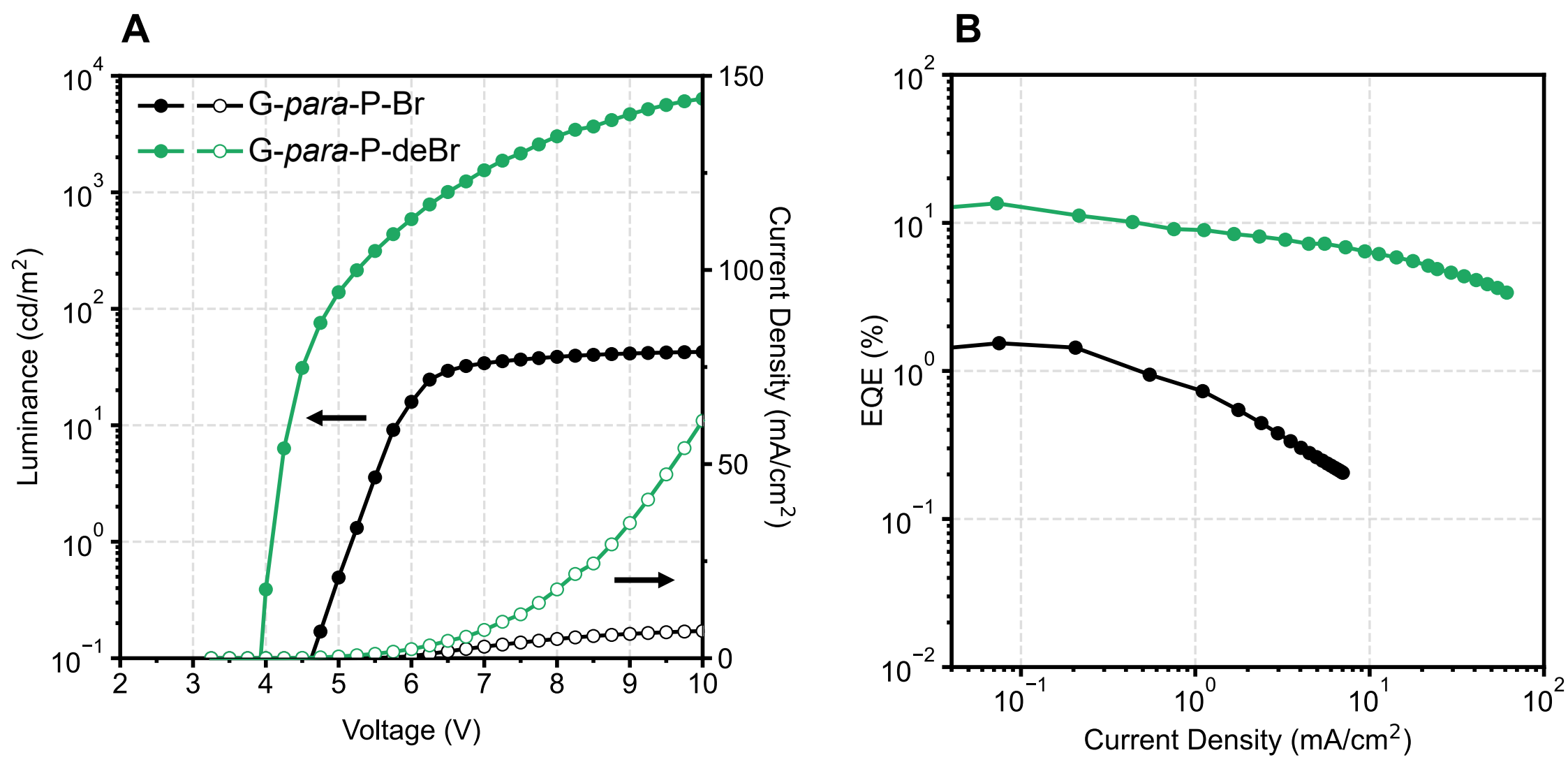

Fig. R2.27 (***Supplementary Fig. 73***) **|** Luminance–voltage (**A**) and EQE-current density (**B**) characteristics of the OLED device employing G-*para*-P-Br (black) or G-*para*-P-deBr (green) as EML. Device architecture: ITO/PEDOT:PSS blend (AI 4083/CH 8000 mix)/EML/TmPyPB/Liq/Al.

Comment 6. *Supplementary Fig. 44 shows a strong PLQY disparity among RGB, with green being much higher than red and blue both for self-hosted polymers and for ELPRs after crosslinking. What is your mechanistic explanation for this RGB dependence? Do you think the limitation is mainly intrinsic to the emitter cores, or is it dominated by the polymer or crosslinker environment? What concrete molecular or processing strategies could raise the blue and red PLQYs while retaining photo-patternability?*

We thank the Reviewer for this insightful photophysical question. As will be detailed later, the observed PLQY disparity among the RGB ELPRs does not stem from a fundamentally destructive crosslinking environment, but rather arises from a synergistic interplay between the intrinsic energetic limitations of the parent emitters and the "universal" dielectric environment, which was developed and optimized for the green emitters of our first-generation polymer matrix.

To clearly address the Reviewer's concern, we outline the mechanistic origins and concrete optimization strategies as follows:

1. Mechanistic Origin: Intrinsic Energetics vs. Polymer Environment:

The inherent radiative efficiencies of the core emitters are intrinsically disparate. DMAC-TRZ (green) is a benchmark TADF core capable of PLQY > 90%. In contrast, the red core (DMAC-NAI) is fundamentally limited by the Energy Gap Law, where the smaller energy gap exponentially accelerates non-radiative decay (PLQY ~ 60% even in optimized hosts).[4] On the other hand, although the blue core (DMAC-DPS) possesses high baseline efficiency (PLQY > 80%) but is unfortunately sensitive to local matrix polarity.[5,6] Therefore, a significant baseline disparity exists independent of the crosslinking chemistry.

Second, to cleanly isolate the variables of photolithography and validate monolithic patternability, we utilized a universal host matrix, namely ACz-based acrylate/methacrylate scaffold, for all three colors. The intrinsic ester functionalities of the acrylate backbone introduce a moderate, fixed baseline polarity. This specific dielectric environment was optimized to energetically match to the charge-transfer (CT) characteristics of the green DMAC-TRZ. However, the fixed polarity is suboptimal for the blue emitter, thereby: (i) triggering polarity-induced stabilization of the CT state that opens non-radiative pathways, and (ii) failing to provide the stringent triplet confinement required for the red and blue excitons. In other words, the polymer environment does not actively "quench" the excitons via defective crosslinkers, but rather acts as a mismatched energetic host for the red and blue cores, as illustrated in **Fig. R2.28 below.**

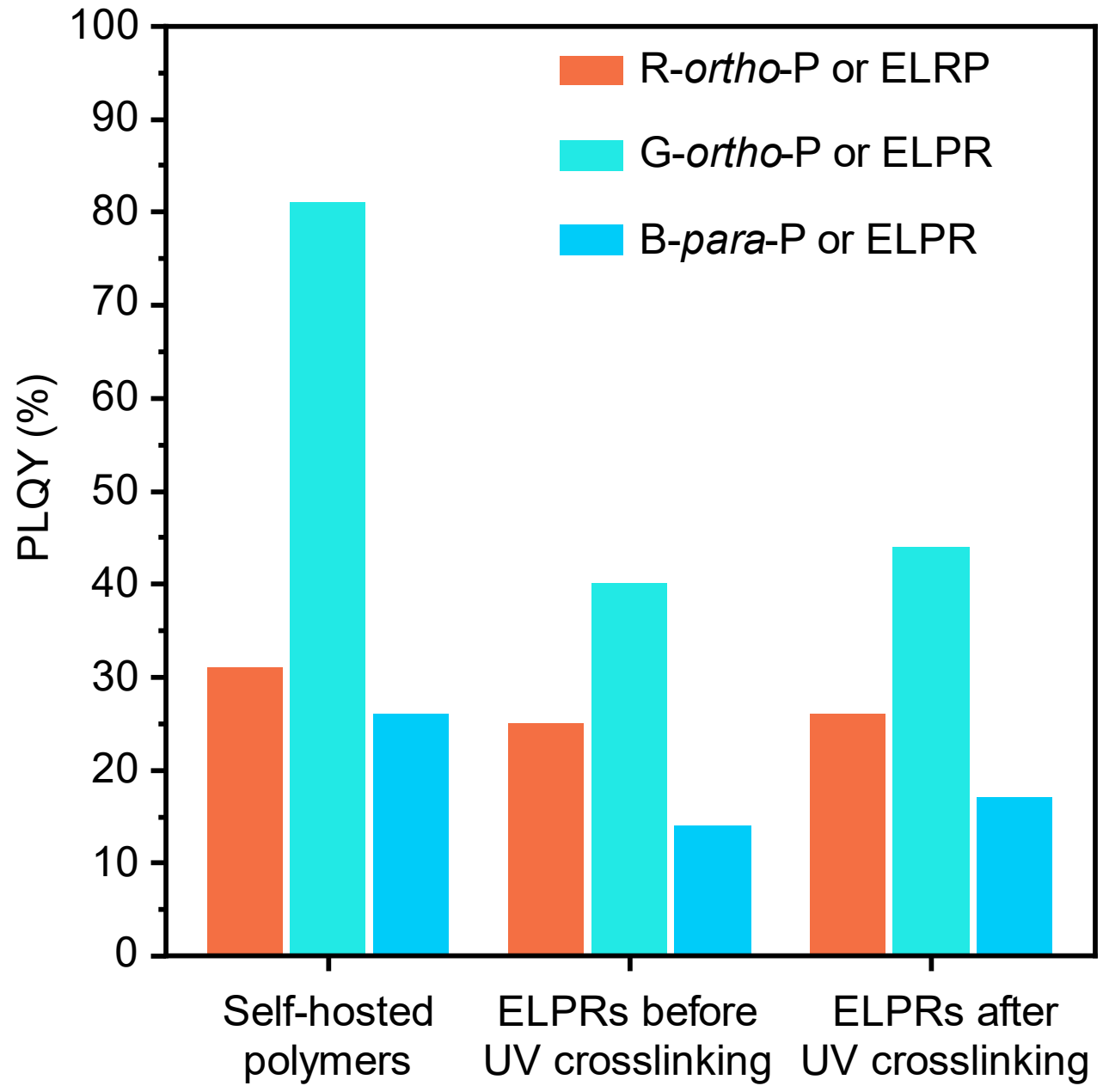

Fig. R2.28 | (*Supplementary Fig. 44*): Absolute thin-film PLQYs histograms of self-hosted TADF polymers and ELPRs before and after crosslinking under nitrogen atmosphere. Self-hosted TADF polymers after end-group de-activation: R-ortho-P (DP_{ACz} = 42), G-*ortho*-P (DP_{ACz} = 45), and B-*para*-P (DP_{ACz} = 50); ELPRs: R-*ortho*-ELPR (DP_{ACz} = 42, DP_{MAc} = 9), G-*ortho*-ELPR (DP_{ACz} = 45, DP_{MAc} = 12), and B-*para*-ELPR (DP_{ACz} = 50, DP_{MAc} = 12)

2. Future Molecular Strategies for Next-Generation ELPRs:

Moving forward, raising the red and blue PLQYs while preserving high-resolution photo-patternability can be achieved through targeted molecular engineering:

- **For Blue ELPRs (Polarity & Confinement Control):** The polar methacrylate crosslinkers can be substituted with low-polarity, crosslinkable vinyl-styrene derivatives. The integration of high-triplet-energy monomeric hosts (e.g., phosphine oxide derivatives, with T_1 > 3.0 eV) into the backbone will severely suppress Dexter energy back-transfer, securely confining blue excitons.

- **For Red ELPRs (Rigidification):** Suppressing the Energy Gap Law requires suppressing vibronic coupling. We can integrate highly rigidified, multi-resonant or fused-ring red cores that are far less susceptible to the low-frequency torsional vibrations of the surrounding polymer matrix.

- **The Ultimate Bypass (MR-TADF Emitters):** As demonstrated in our response to **Comment 2**, transitioning from conventional CT-based TADF to Multi-Resonance TADF (MR-TADF) cores fundamentally solves this issue. Because MR-TADF emitters possess localized excited states, they are highly shielded from the surrounding polymer's dielectric environment. Our successful integration of the DABNA and DtCzB cores (yielding 29–33 nm FWHM) proves that the ELPR

photolithography platform is highly compatible with these advanced, environment-insensitive emitters.

In summary, our primary objective in this proof-of-concept study was to establish the scalability and ultrahigh-resolution patternability of our proposed ELPR molecular design strategy. The development of color-specific, energetically tailored host-crosslinker palettes represents an exciting, clearly defined roadmap for subsequent optimization.

Comment 7. *The manuscript would benefit from more explicitly positioning ELPRs relative to conventional OLED patterning approaches, as well as quantum-dot photoresists (QD-PR), micro-LEDs, and perovskite LED technologies. A quantitative comparison across key performance metrics such as patterning resolution, process compatibility, peak luminance, efficiency, spectral purity, and operational stability would help clarify the practical differentiation and potential application space of ELPRs.*

We thank the Reviewer for providing this strategic opportunity to position our ELPR platform within the broader landscape of modern LED technologies. We have compiled **Table R2.4** (incorporated into revised **Supplementary Table 11**), which carefully benchmarks our fully organic ELPR platform against existing technologies, including Fine Metal Mask (FMM) OLEDs, Quantum-Dot Photoresists (QD-PRs), Perovskite photoresists (Pe-PRs), and inorganic III–V micro-LEDs across all requested metrics.

In addition to the outlined comparisons, we would like to highlight the following remarks.

1. Breaking the Resolution Limits and Process Orthogonality of Conventional OLEDs:

Compared to the vacuum-evaporated OLEDs relying on FMM that hit a severe mechanical shadow-mask resolution limit (pixel size down to ~15 - 30 μm), ELPRs clearly outperformed in resolution and process orthogonality. With ELPRs, one can not only pattern OLEDs down to the diffraction limit of visible light (L/S = 110/150 nm; see **Fig. 4** in main text), but also perform multiple photographic process to define overlaying layers after OLED deposition (**Extended Data Fig. 2**). We believe ELPRs will open the door towards ultrahigh-density near-eye microdisplays.

2. Superior Monolithic Process Robustness and Resolution Limits over QD-PRs and Pe-PRs:

We would like to point out that in the literature, QD-PR and Pe-PR systems are usually termed as "photocrosslinkable QDs and Perovskites", because they are not fully polymeric systems, despite technical relevance. Although recent QD-PR and Pe-PRs have demonstrated remarkable progress, their practical manufacturing is considerably hindered by ligand detachment, ion migration, and chemical degradation during standard developer and strip cycles. Our ELPRs are fully organic, covalently cross-linked networks, so they exhibit absolute solvent-orthogonality and structural integrity throughout harsh, multi-step photolithographic sequences. Most importantly, because of the nature of hybrid materials, the QD-PRs and Pe-PRs can only demonstrate pattern resolution down to ~ 1 μm (see Table R2.4). From the viewpoint of "extending the Moore's law to multicolor LEDs", our ELPRs would certainly stand out.

3. Circumventing the Mass-Transfer Bottleneck of Micro-LEDs:

While inorganic III–V micro-LEDs deliver unmatched absolute brightness and thermodynamic lifetime, their mass manufacturability remains strictly bottlenecked by high-temperature epitaxial growth and yield-sensitive, heterogeneous pick-and-place transfer schemes. Solution-processed ELPRs completely circumvent these mechanical assembly hurdles via direct, low-temperature additive/subtractive patterning on arbitrary backplanes, including CMOS chips.

All in all, as illustrated in **Table R2.4**, ELPRs place itself at a unique technoeconomic sweet spot: **they combine the extreme resolution and monolithic scalability with CMOS photolithography, with the low-temperature orthogonal solution-processability of organic semiconductors.** While inorganic platforms will continue to dominate high-luminance outdoor signage, ELPRs establish a highly competitive framework for next-generation, ultrahigh-pixel-density integrated optoelectronics.

Table R2.4 (***Supplementary Table 11***) **|** Quantitative comparison of patterning strategies for various LED technologies, including inorganic QD-LEDs, III-V micro-LEDs, perovskite LEDs, and ELPR OLEDs.

LEDs	Pixelization process	Process compatibility	Feature size	Peak L (cd/m^2)	EQE$_{max}$ (%)	FWHM (nm)	Device operational stability	Reference
Inorganic QD-LEDs	Direct photolithography	Solvent orthogonal & Scalable	1 µm	7851	25.6	20~30	*LT_{95}:135 hrs*	Nat. comm. (2025)[7]
	Direct photolithography	Solvent orthogonal & Scalable	0.8 µm	>10^5	< 20	20~40	*LT_{95}:~55 hrs*	Nat. Nanotechnol. (2022)[8]
	Transfer printing	Dry process & transfer yield issue	400 nm	~10^5	23.3	20~30	-	Nat. photonics (2024)[9]
	Lift-off photolithography	Solvent orthogonal & Scalable	0.8 µm	< 10^5	16.6	20~30	-	Nat. comm. (2026)[10]
III-V micro-LEDs	Ion implantation pixelization	Suitable for single-color pixelization	0.5 µm	55835	12	4.8	*LT_{95}: > 300 hrs*	Nat. photonics (2021)[11]
	Fluidic assembly	Suitable for sub-100 micron pixelization	45 µm	-	-	25~30	-	Nature (2023)[12]
	Layer transfer + photolithography	Scalability limited to epitaxial substrate size	4 µm	<10^5	< 0.5	25~45	-	Nature (2023)[13]
Perovskite LEDs	Direct photolithography	Solvent orthogonal & Scalable	5 µm	> 20000	6.8	24	*LT_{50}:5~7* mins	Sci. Adv. (2022)[14]
	Transfer printing	Dry process & transfer yield issue	400 nm	4159	14.8	18	*LT_{50}:22* mins	Sci. Adv. (2022)[15]
	Inkjet printing	Feature size limited to nozzle/drop size	~100 µm	43883	8.54	20	*LT_{50}:64* mins	Adv. Mater. (2022)[16]
**ELPR OLEDs**	**Direct photolithography E-beam lithography**	**Solvent orthogonal & Scalable**	**110 nm**	**2068**	**13.3**	**85~100**	***LT_{50}:172 mins***	**This work**

Minor comments

1. Please clarify the measurement conditions used for lifetime characterization, including initial luminance, driving mode (constant current or constant voltage), encapsulation conditions, and measurement environment. Including these details in the figure captions would improve clarity and reproducibility.

We thank the reviewer for this valuable feedback. Please find our detailed response as follows.

The operational lifetime (LT_{50}) of the devices in the original manuscript was characterized under a constant driving current density (J_{driv}) of 1 mA cm^{-2}, corresponding to initial luminance of 100 cd m^{-2}. During the revision process, we further optimized the device architecture by systematically varying the carrier-transporting materials and significantly reducing the residual copper complex within the TADF polymer emission layer via dialysis. To evaluate the LT_{50}, the resulting optimal devices comprising a v-NPB as HTL, a TPBi as ETL, and the purified TADF polymer emission layer were subjected to electrical stress at varying driving current densities (J_{driv}) of 2.4, 5.0, and 10 mA cm^{-2}. These J_{driv} yielded corresponding initial luminances of 175, 360, and 635 cd m^{-2}, respectively.

We have added following sentences on page 5-6 to the Supplementary information in the Measurement section. We have also added the driving current densities in the revised figure panels.

[*The operational lifetime (*LT_{50}*) of the encapsulated devices were evaluated at room temperature under ambient relative humidity conditions. Long-term stability characterization was executed using a commercial, 16 parallel stressing channels in 4 different chambers, Litos LED Lifetime System (Fluxim AG) equipped with calibrated silicon photodiodes to track luminance decay. The devices were operated in a constant direct-current (DC) driving mode at varied current densities of 1 mA* cm^{-2}*, 2.4 mA* cm^{-2}*, 5 mA* cm^{-2}*, and 10 mA* cm^{-2}*.*]

Device Encapsulation

Following the thermal deposition of the aluminum electrode, the fabricated devices were immediately transferred without atmospheric exposure into an integrated, dry nitrogen-filled glovebox (O_2 < 0.1 ppm, H_2O < 0.1 ppm) for hermetic encapsulation. The devices were sealed using a chemically etched aluminosilicate cavity cover glass integrated with a pre-conditioned desiccant label to scavenge residual moisture (**Fig. R2.29**). A UV-curable NOA 61 epoxy resin sealant was precisely dispensed along the perimeter of the cover glass. The encapsulation architecture was then pressed securely against the device substrate and cured under ultraviolet radiation (λ = 365 nm) for 30 seconds. A pixel-aligned shadow mask was utilized during exposure to completely shield the active polymer emissive area from UV-induced degradation.

We have added following sentences on page 16 of the revised manuscript in the Methods section. We have also added **Fig. R2.29 below** (**Supplementary Fig. 74)** on page 99 in the Supplementary information.

[**Device encapsulation**

Following the thermal deposition of the aluminum electrode, the fabricated devices were immediately transferred without atmospheric exposure into an integrated, dry nitrogen-filled glovebox (O_2 < 0.1 ppm, H_2O < 0.1 ppm) for hermetic encapsulation. The devices were sealed using a chemically etched aluminosilicate cavity cover glass integrated with a pre-conditioned desiccant label to scavenge residual moisture (**Supplementary Fig. 74**). A UV-curable NOA 61 epoxy resin sealant was precisely dispensed along the perimeter of the cover glass. The encapsulation architecture was then pressed securely against the device substrate and cured under ultraviolet radiation (λ = 365 nm) for 30 seconds. A pixel-aligned shadow mask was utilized during exposure to completely shield the active polymer emissive area from UV-induced degradation.]

Operational Lifetime Measurements

The LT_{50} of the encapsulated devices were evaluated at room temperature under ambient relative humidity conditions. Long-term stability characterization was executed using a commercial, 16-channels Litos Lifetime System (Fluxim AG) equipped with calibrated silicon photodiodes to track luminance decay. The devices were operated in a constant direct-current (DC) driving mode at varied current densities (J_{driv}) of 2.4, 5.0, and 10 mA cm^{-2}, which corresponded to initial luminances (L_0) of 175, 360, and 635 cd m^{-2}, respectively. The LT_{50} value was defined precisely as the elapsed operational timeframe required for the device luminance to decay to 50% of its baseline value (L/L_0 = 0.5).

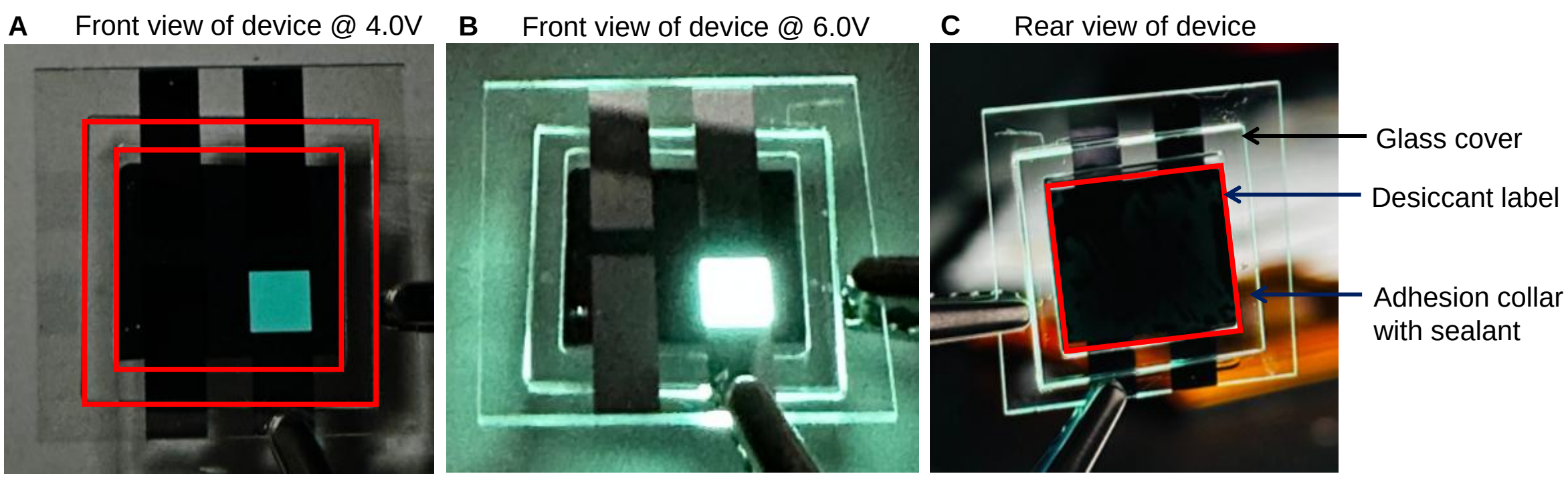

Fig. R2.29 ***(Supplementary Fig. 74)*** **|** Photographs of encapsulated OLED devices used for the operation lifetime (LT_{50}) measurement. (A) Front view of device driving at 4.0V, (B) Front view of device driving at 6.0V, and (C) Rear view of device driving at 6.0V.

2. *Please provide additional details on PLQY measurements, including film thickness, excitation wavelength, and integrating sphere configuration. This information would facilitate comparison across RGB systems.*

We thank the Reviewer for raising this point. The absolute Φ_{PL} values of emission layer solid-state thin films and 0.1 mM solutions in the toluene were determined using the Quantaurus QY (C11347-11) from Hamamatsu equipped with 150 W Xenon light source and 3.3 inches integrating sphere, which is coated with highly reflective Spectralon and calibrated with standard samples. The Φ_{PL} values of both solution and thin film samples were characterized at an excitation wavelength (λ_{ext}) of 340 nm by using a software-controlled monochromator. Solution-state Φ_{PL} values were characterized using 0.1 mM solutions in toluene. Thin-film samples of all polymers and ELPRs were prepared by spin-coating 5 mg mL^{-1} toluene solutions at 2,000 rpm for 40 s, yielding film thicknesses of 23 ± 2 nm for solid-state Φ_{PL} values measurements. The toluene solutions were bubbled with oxygen for 10 minutes to quench triplet-mediated delayed emission and thereby determine the prompt fluorescence Φ_{PF} values. For solution-state Φ_{PL} measurements, the samples were purged with argon for 30 min to remove dissolved oxygen, enabling determination of the total Φ_{PL} comprising both prompt and delayed fluorescence components. For thin-film Φ_{PL} measurements, the integrating sphere was purged with dry nitrogen for 20 minutes before measurement, and a continuous low flow of nitrogen was maintained throughout the measurement.

3. *Including an energy-level diagram of the full device stack (HOMO/LUMO levels with references) would help clarify charge injection and balance, particularly in relation to the reported efficiency improvement after end-group deactivation.*

We thank the reviewers for the suggestion. The energy-levels of HTLs, self-hosting G-*ortho*-P-deBr as EML, ETLs, electron injection and Al cathode layers for our OLED devices are shown in **Fig. R2.30** (**Supplementary Fig. 75**) to illustrate charge injection, transport, and confinement, including all transport and injection layers used during device optimization.

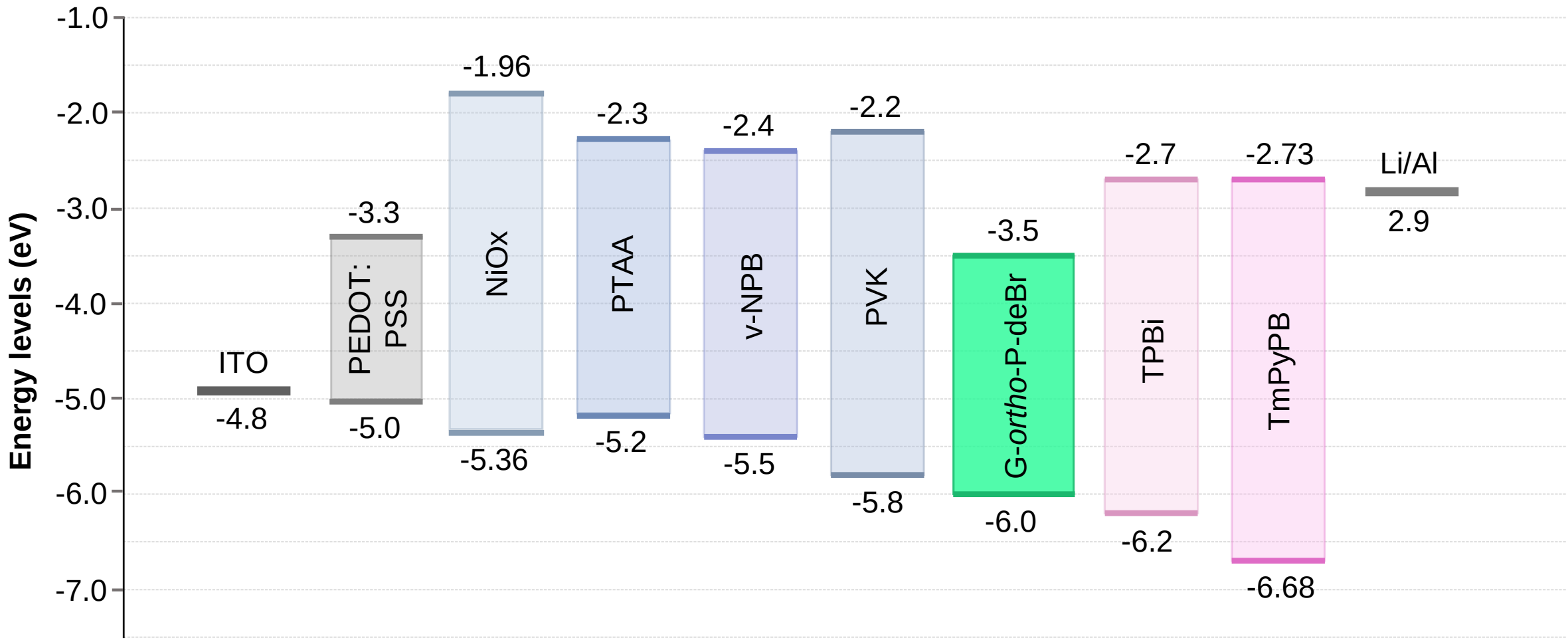

Fig. R2.30 (***Supplementary Fig. 75***) **|** Combined device architecture and energy-level diagram, showing the HOMO and LUMO levels of all transport and injection layers studied during device optimization. G-*ortho*-P-deBr is employed as EML, where the of the various transporting materials studied during the optimization process were displayed. Values indicate the HOMO and LUMO energies in eV relative to the vacuum level.[17-22]

At the end, we would like to express our sincere appreciation to the Reviewer's constructive and insightful suggestions.

Referee #3*:*

I co-reviewed this manuscript with one of the reviewers who provided the listed reports.

We sincerely thank Reviewers 2 and 3 for their insightful assessment and constructive feedback. Within the scope of this manuscript, we have done our best to address all Reviewers' comments. We hope the Reviewers would appreciate our effort and scientific discussions presented here.

Reference

1. Goushi, K., Yoshida, K., Sato, K. & Adachi, C. Organic light-emitting diodes employing efficient reverse intersystem crossing for triplet-to-singlet state conversion. *Nat. Photonics* **6**, 253–258 (2012).

2. Masui, K., Nakanotani, H. & Adachi, C. Analysis of exciton annihilation in high-efficiency sky-blue organic light-emitting diodes with thermally activated delayed fluorescence. *Org. Electron.* **14**, 2721–2726 (2013).

3. Serevičius, T. *et al.* TADF parameters in the solid state: an easy way to draw wrong conclusions. *J. Phys. Chem. A* **125**, 1637–1641 (2021).

4. Zeng, W. *et al.* Achieving nearly 30% external quantum efficiency for orange–red organic light emitting diodes by employing thermally activated delayed fluorescence emitters composed of 1,8-naphthalimide-acridine hybrids. *Adv. Mater.* **30**, 1704961 (2018).

5. Zhang, Q. *et al.* Efficient blue organic light-emitting diodes employing thermally activated delayed fluorescence. *Nat. Photonics* **8**, 326–332 (2014).

6. Tsai, W.-L. *et al.* A versatile thermally activated delayed fluorescence emitter for both highly efficient doped and non-doped organic light emitting devices. *Chem. Commun.* **51**, 13662-13665 (2015).

7. Guan, J. *et al.* Direct ambient photopatterning of RGB quantum dots for light-emitting diodes with EQE exceeding 20%. *Nat. Commun.* **16**, 9689 (2025).

8. Hahm, D. *et al.* Direct patterning of colloidal quantum dots with adaptable dual-ligand surface. *Nat. Nanotechnol.* **17**, 952–958 (2022).

9. Yoo, J. *et al.* Highly efficient printed quantum dot light-emitting diodes through ultrahigh-definition double-layer transfer printing. *Nat. Photonics* **18**, 1105-1112 (2024).

10. Kim, H. *et al.* Photoresist-guided indirect photopatterning of quantum dots via carbene-mediated ligand thermocrosslinking. *Nat. Commun.* **17**, 4162 (2026).

11. Park, J. *et al.* Electrically driven mid-submicrometre pixelation of ingan micro-light-emitting diode displays for augmented-reality glasses. *Nat. Photonics* **15**, 449–455 (2021).

12. Lee, D. *et al.* Fluidic self-assembly for microLED displays by controlled viscosity. *Nature* **619**, 755-760 (2023).

13. Shin, J. *et al.* Vertical full-colour micro-leds via 2d materials-based layer transfer. *Nature* **614**, 81-87 (2023).

14. Liu, D. *et al.* Direct optical patterning of perovskite nanocrystals with ligand cross-linkers. *Sci. Adv.* **8**, eabm8433 (2022).

15. Kwon, J. I. *et al.* Ultrahigh-resolution full-color perovskite nanocrystal patterning for ultrathin skin-attachable displays. *Sci. Adv.* **8**, eadd0697 (2022).

16. Wei, C. *et al.* A universal ternary-solvent-ink strategy toward efficient inkjet-printed perovskite quantum dot light-emitting diodes. *Adv. Mater.* **34**, 2107798 (2022).

17. Jou, J. H. Kumar, S. Agrawal, A. Li, T. H. & Sahoo, S. Approaches for fabricating high efficiency organic light emitting diodes. *J. Mater. Chem. C*, **3**, 2974-3002 (2015).

18. Qin, J. *et al.* Surfactant-induced hole concentration enhancement for highly efficient perovskite light-emitting diodes. *Nat. Mater.* **24**, 778–784 (2025).

19. Wang, X. *et al.* Co-self-assembled monolayers modified NiO_x/perovskite interface for high-performance wide bandgap perovskite solar cells. *ACS Appl. Nano Mater.* **8** (15) 7787-7795 (2025).

20. Li, Y. *et al.* Modulation of charge transport layer for perovskite light-emitting diodes. *Adv. Mater.* **37**, 2410535 (2025).

21. Kim, D. H. *et al.* Thermally cross-linkable blended hole transport layer for solution-processed quantum dot light-emitting diodes. *ACS Appl. Nano Mater.* **8** (34) 16727-16735 (2025).

22. Li, W. Zhang, X. Zhang, X. Yao, J. & Zhan, C. High-performance solution-processed single-junction polymer solar cell achievable by post-treatment of PEDOT:PSS layer with water-containing methanol. ACS Appl. Mater. Interfaces, **9** (2) 1446-1452 (2017).

Response to Reviewers' comments

Reviewer #1

The authors have satisfactorily addressed all of my major concerns. The additional photophysical characterization, including extraction of the TADF kinetic parameters and temperature-dependent TRPL analysis, substantially strengthens the mechanistic discussion. Furthermore, the extensive reproducibility studies, including statistical device analysis, repeated scan measurements, hysteresis tests, leakage-current analysis, EL microscopy, and AFM characterization, convincingly demonstrate that the reported device performance is robust.

I have no further major concerns and recommend acceptance after editorial revision.

We sincerely thank Reviewer 1 for the positive and constructive feedback. We are very pleased that both Reviewers recognize our effort and support publication of our manuscript.

Reviewers #2 and #3

The authors have made substantial efforts to address my previous comments and have provided extensive additional experimental data. The newly added purification studies, device-stack optimization, high-current characterization, and technology comparison significantly strengthen the manuscript. Overall, most of my previous concerns have been adequately addressed. I have only a few remaining comments that may help further clarify the interpretation and presentation of the results.

We sincerely thank Reviewers 2 and 3 for the positive and constructive feedback. We are very pleased that both Reviewers recognize our effort and support publication of our manuscript. We have addressed the Reviewers' final comments as follows.

1. Interpretation of the operational lifetime limitation: The additional ICP-OES and purification experiments provide useful evidence that residual copper contributes significantly to the observed operational lifetime limitation. In particular, the reduction in residual copper from 27 to 14 ppm and the corresponding improvement in LT50 after dialysis clearly support the influence of metallic impurities on device stability. Similarly, the lifetime improvement achieved through transport-layer optimization suggests that charge balance and interfacial charge accumulation also play important roles in the degradation behavior. These additional results substantially strengthen the discussion of extrinsic factors affecting the operational stability of the devices.

At the same time, it may be helpful to slightly moderate the interpretation that the current stability bottleneck is primarily governed by these extrinsic factors rather than intrinsic aspects of the ELPR molecular or polymer architecture. The present results clearly demonstrate that purification and device-stack optimization can improve operational lifetime. However, such improvements establish the significant contribution of extrinsic degradation pathways rather than directly excluding possible intrinsic stability limitations of the ELPR system. In particular, the relative contributions of the dual functional polymer architecture, crosslinkable groups, and other material-intrinsic degradation mechanisms have not yet been independently evaluated. Therefore, statements suggesting that the current stability metrics are governed by process-level cleanliness "rather than an intrinsic thermodynamic ceiling," or that the stability bottleneck arises from extrinsic factors "rather than an intrinsic design flaw," may be somewhat stronger than can be concluded from the current data. I suggest slightly moderating these statements to reflect that extrinsic factors have been experimentally demonstrated to contribute significantly to the present lifetime limitation, while the possible contribution of intrinsic degradation mechanisms remains to be further clarified.

We thank the Reviewer for raising the question. We agree with the Reviewer that we cannot exclude all intrinsic scenarios resulting in the reduced device operational lifetimes. To address the Reviewer's concern, on Page 13, we have modified the following sentence.

Original: "These results underscore that device operational stability is mainly governed by the nature of the underlying HTLs, while the effects of crosslinker loading and UV crosslinking in ELPRs are negligible."

New: "While these findings highlight that the extrinsic factors, including HTLs and metal impurities, significantly limit current device operational stability, the potential contributions of intrinsic degradation mechanisms, such as those related to the dual-functional polymer architecture and crosslinkable groups, remain to be independently evaluated."

2. Peak high-current operation and continuous operation: The newly added high-luminance data clearly demonstrate that current density and peak luminance can be substantially improved through device-stack optimization. These results address my previous concern regarding the potential for improving device brightness.

It would nevertheless be helpful to more clearly distinguish between peak high-current operation and stable continuous operation. The J–V–L measurements demonstrate operation at current densities of approximately 126–360 mA cm^{-2}, whereas the continuous lifetime measurements are performed up to 10 mA cm^{-2}. I suggest clarifying this distinction when discussing stable high-current operation and Joule-heating- or breakdown-related limitations.

We thank the Reviewer for raising the question. We agree with the Reviewer that the stable operation and high-current operation belong to two distinct regimes. To address the Reviewer's question, we have added the following sentence on Page 13:

"It should be noted that a distinction must be made between peak high-current operation and continuous stable operation. While J-V-L measurements demonstrate that the devices can sustain transient current densities well over 100 mA cm^{-2}, continuous long-term operation is currently restricted to 10 mA cm^{-2} or below to mitigate excessive Joule heating and prevent premature electrical breakdown."

Overall, the revised manuscript has been significantly strengthened, and the authors have addressed the major concerns raised in my previous review. The remaining comments are primarily intended to improve the clarity and precision of the interpretation of the newly added results. I would support publication after these points are appropriately clarified.

We again thank the Reviewers for their constructive and supportive comments. We have addressed the Reviewers' final questions and hope the Reviewers would appreciate our additions.

The authors address a longstanding challenge in OLED nano-patterning by integrating TADF emitters with photoresist chemistry to directly pattern RGB subpixels. The demonstration of photolithographic patterning of functional OLED emitters represents a novel and technically ambitious approach, and this work offers a meaningful conceptual advance over conventional patterning strategies that have been considered difficult for OLED materials.

However, the technology appears to face significant performance limitations relative to conventional OLED patterning methods. It would be valuable for the authors to more clearly discuss whether ELPR-based OLEDs are realistically compatible with practical display requirements, and to outline how further material or device development could enable meaningful competition with existing commercial technologies.

Major comments

1. The operational lifetime of the ELPR OLEDs reported in this work is substantially shorter than that reported for many OLED and TADF-based systems in the recent literature. The manuscript attributes the limited lifetime mainly to extrinsic factors such as HTL choice, residual copper, and polymer impurities. However, it remains unclear whether the observed lifetime limitation arises primarily from insufficient materials and process optimization, or from intrinsic constraints imposed by the ELPR molecular and polymer architecture, which is designed to simultaneously support electroluminescence and photolithography.
   While the authors attribute the present lifetime limitations primarily to extrinsic factors, the manuscript provides limited evidence to substantiate this interpretation. It remains unclear to what extent these factors dominate the degradation behavior relative to the intrinsic stability of the ELPR materials. Additional experimental data would help clarify the relative contributions of material, process, and device-level effects, and would strengthen the authors' argument regarding the origin of the lifetime limitations.
   Also, based on the current materials system, what level of lifetime improvement do the authors consider realistically achievable?

2. The manuscript refers to "full-color" electroluminescence based on the demonstrated RGB ELPR devices. However, the reported EL peaks (approximately 493 nm for blue and 586 nm for red) appear significantly shifted from standard display blue and red primaries. It is therefore unclear whether the present emission characteristics alone justify the use of the term "full color." In addition, the EL spectra shown in the manuscript appear relatively broad, suggesting limited color purity.
   Can the authors provide the CIE 1931 (x, y) color coordinates? How do these values compare with commonly accepted display requirements for color purity and gamut coverage?
   Furthermore, the emission wavelengths appear to be largely determined by the choice of

the emitter core. The authors are encouraged to clarify the rationale behind selecting the current blue and red emitter cores, and to explain why deeper-blue and deeper-red emitters, which are typically required for display-grade color standards, were not explored in this study. In addition, it would be helpful if the authors could discuss the origin of the relatively broad emission bandwidths observed in the EL spectra. Are these linewidths intrinsic to the ELPR material design or processing conditions? A discussion of the spectral broadening mechanisms and potential pathways to improve color purity would significantly strengthen the manuscript.

3. Based on the data presented, the high-brightness operating capability of the ELPR OLEDs does not appear to be experimentally demonstrated. While electroluminescence and uniform emission are shown, the manuscript does not provide clear evidence that the devices can sustain the high current densities required for brightness levels relevant to practical OLED or micro-display applications.
   Can the authors clarify whether the present ELPR material architecture is fundamentally capable of stable operation in the high-current, high-brightness regime? The current manuscript does not appear to provide sufficient experimental evidence demonstrating that brightness can be substantially improved through process or device optimization.

4. The J–V and L–V characteristics indicate that current density and luminance do not continue to increase at higher driving voltages, and in some regimes appear to saturate or decrease. The manuscript does not explicitly address the physical origin of this behavior.
   Can the authors clarify whether this high-voltage response is associated with irreversible current-induced degradation, charge imbalance and transport limitations, or other exciton-related quenching processes in the ELPR OLEDs? Importantly, do the authors consider this behavior to be an intrinsic limitation of the ELPR material architecture under high current injection, or a reversible effect that can be mitigated through device or materials optimization.

5. End-group deactivation leads to a dramatic improvement in device efficiency and current efficiency, while the emissive core structure remains unchanged. This suggests that the performance gain may arise primarily from reduced trap-assisted losses or improved film quality rather than from intrinsic changes to the emitter.
   Can the authors clarify to what extent end-group deactivation also improves tolerance to high current injection, high-brightness operation, and operational stability? Is there experimental evidence that deactivation mitigates current-induced degradation or charge-transport limitations, beyond the observed efficiency enhancement at low to moderate operating conditions.

In addition, while the deactivation effect is prominently demonstrated for the green emitter system, it is less clear how consistently this improvement translates to the red and blue ELPRs. Can the authors clarify whether similar trap-related performance enhancement is observed across all RGB systems, and if not, what governs the color-dependent response to end-group deactivation?

6. Supplementary Fig. 44 shows a strong PLQY disparity among RGB, with green being much higher than red and blue both for self-hosted polymers and for ELPRs after crosslinking. What is your mechanistic explanation for this RGB dependence? Do you think the limitation is mainly intrinsic to the emitter cores, or is it dominated by the polymer or crosslinker environment? What concrete molecular or processing strategies could raise the blue and red PLQYs while retaining photo-patternability?

7. The manuscript would benefit from more explicitly positioning ELPRs relative to conventional OLED patterning approaches, as well as quantum-dot photoresists (QD-PR), micro-LEDs, and perovskite LED technologies. A quantitative comparison across key performance metrics such as patterning resolution, process compatibility, peak luminance, efficiency, spectral purity, and operational stability would help clarify the practical differentiation and potential application space of ELPRs.

Minor comments

1. Please clarify the measurement conditions used for lifetime characterization, including initial luminance, driving mode (constant current or constant voltage), encapsulation conditions, and measurement environment. Including these details in the figure captions would improve clarity and reproducibility.

2. Please provide additional details on PLQY measurements, including film thickness, excitation wavelength, and integrating sphere configuration. This information would facilitate comparison across RGB systems.

3. Including an energy-level diagram of the full device stack (HOMO/LUMO levels with references) would help clarify charge injection and balance, particularly in relation to the reported efficiency improvement after end-group deactivation.

The authors address a longstanding challenge in OLED nano-patterning by integrating TADF emitters with photoresist chemistry to directly pattern RGB subpixels. The demonstration of photolithographic patterning of functional OLED emitters represents a novel and technically ambitious approach, and this work offers a meaningful conceptual advance over conventional patterning strategies that have been considered difficult for OLED materials.

However, the technology appears to face significant performance limitations relative to conventional OLED patterning methods. It would be valuable for the authors to more clearly discuss whether ELPR-based OLEDs are realistically compatible with practical display requirements, and to outline how further material or device development could enable meaningful competition with existing commercial technologies.

Major comments

1. The operational lifetime of the ELPR OLEDs reported in this work is substantially shorter than that reported for many OLED and TADF-based systems in the recent literature. The manuscript attributes the limited lifetime mainly to extrinsic factors such as HTL choice, residual copper, and polymer impurities. However, it remains unclear whether the observed lifetime limitation arises primarily from insufficient materials and process optimization, or from intrinsic constraints imposed by the ELPR molecular and polymer architecture, which is designed to simultaneously support electroluminescence and photolithography.
   While the authors attribute the present lifetime limitations primarily to extrinsic factors, the manuscript provides limited evidence to substantiate this interpretation. It remains unclear to what extent these factors dominate the degradation behavior relative to the intrinsic stability of the ELPR materials. Additional experimental data would help clarify the relative contributions of material, process, and device-level effects, and would strengthen the authors' argument regarding the origin of the lifetime limitations.
   Also, based on the current materials system, what level of lifetime improvement do the authors consider realistically achievable?

2. The manuscript refers to "full-color" electroluminescence based on the demonstrated RGB ELPR devices. However, the reported EL peaks (approximately 493 nm for blue and 586 nm for red) appear significantly shifted from standard display blue and red primaries. It is therefore unclear whether the present emission characteristics alone justify the use of the term "full color." In addition, the EL spectra shown in the manuscript appear relatively broad, suggesting limited color purity.
   Can the authors provide the CIE 1931 (x, y) color coordinates? How do these values compare with commonly accepted display requirements for color purity and gamut coverage?
   Furthermore, the emission wavelengths appear to be largely determined by the choice of

the emitter core. The authors are encouraged to clarify the rationale behind selecting the current blue and red emitter cores, and to explain why deeper-blue and deeper-red emitters, which are typically required for display-grade color standards, were not explored in this study. In addition, it would be helpful if the authors could discuss the origin of the relatively broad emission bandwidths observed in the EL spectra. Are these linewidths intrinsic to the ELPR material design or processing conditions? A discussion of the spectral broadening mechanisms and potential pathways to improve color purity would significantly strengthen the manuscript.

3. Based on the data presented, the high-brightness operating capability of the ELPR OLEDs does not appear to be experimentally demonstrated. While electroluminescence and uniform emission are shown, the manuscript does not provide clear evidence that the devices can sustain the high current densities required for brightness levels relevant to practical OLED or micro-display applications.

   Can the authors clarify whether the present ELPR material architecture is fundamentally capable of stable operation in the high-current, high-brightness regime? The current manuscript does not appear to provide sufficient experimental evidence demonstrating that brightness can be substantially improved through process or device optimization.

4. The J–V and L–V characteristics indicate that current density and luminance do not continue to increase at higher driving voltages, and in some regimes appear to saturate or decrease. The manuscript does not explicitly address the physical origin of this behavior.

   Can the authors clarify whether this high-voltage response is associated with irreversible current-induced degradation, charge imbalance and transport limitations, or other exciton-related quenching processes in the ELPR OLEDs? Importantly, do the authors consider this behavior to be an intrinsic limitation of the ELPR material architecture under high current injection, or a reversible effect that can be mitigated through device or materials optimization.

5. End-group deactivation leads to a dramatic improvement in device efficiency and current efficiency, while the emissive core structure remains unchanged. This suggests that the performance gain may arise primarily from reduced trap-assisted losses or improved film quality rather than from intrinsic changes to the emitter.

   Can the authors clarify to what extent end-group deactivation also improves tolerance to high current injection, high-brightness operation, and operational stability? Is there experimental evidence that deactivation mitigates current-induced degradation or charge-transport limitations, beyond the observed efficiency enhancement at low to moderate operating conditions.

In addition, while the deactivation effect is prominently demonstrated for the green emitter system, it is less clear how consistently this improvement translates to the red and blue ELPRs. Can the authors clarify whether similar trap-related performance enhancement is observed across all RGB systems, and if not, what governs the color-dependent response to end-group deactivation?

6. Supplementary Fig. 44 shows a strong PLQY disparity among RGB, with green being much higher than red and blue both for self-hosted polymers and for ELPRs after crosslinking. What is your mechanistic explanation for this RGB dependence? Do you think the limitation is mainly intrinsic to the emitter cores, or is it dominated by the polymer or crosslinker environment? What concrete molecular or processing strategies could raise the blue and red PLQYs while retaining photo-patternability?

7. The manuscript would benefit from more explicitly positioning ELPRs relative to conventional OLED patterning approaches, as well as quantum-dot photoresists (QD-PR), micro-LEDs, and perovskite LED technologies. A quantitative comparison across key performance metrics such as patterning resolution, process compatibility, peak luminance, efficiency, spectral purity, and operational stability would help clarify the practical differentiation and potential application space of ELPRs.

Minor comments

1. Please clarify the measurement conditions used for lifetime characterization, including initial luminance, driving mode (constant current or constant voltage), encapsulation conditions, and measurement environment. Including these details in the figure captions would improve clarity and reproducibility.
2. Please provide additional details on PLQY measurements, including film thickness, excitation wavelength, and integrating sphere configuration. This information would facilitate comparison across RGB systems.
3. Including an energy-level diagram of the full device stack (HOMO/LUMO levels with references) would help clarify charge injection and balance, particularly in relation to the reported efficiency improvement after end-group deactivation.
